# Supplementary material for: Ultra-High-Throughput Clinical Proteomics Reveals Classifiers of COVID-19 Infection
Source: Cell Syst. 2020 Jul 22;11(1):11–24.e4. doi: 10.1016/j.cels.2020.05.012 (PMC7264033; doi:10.1016/j.cels.2020.05.012)
Supplement: Document S1. Figures S1–S8 and Tables S1–S6 [file mmc1.pdf]

Cell Systems, Volume *11*

Supplemental Information

Ultra-High-Throughput Clinical Proteomics

Reveals Classifiers of COVID-19 Infection

Christoph B. Messner, Vadim Demichev, Daniel Wendisch, Laura Michalick, Matthew White, Anja Freiwald, Kathrin Textoris-Taube, Spyros I. Vernardis, Anna-Sophia Egger, Marco Kreidl, Daniela Ludwig, Christiane Kilian, Federica Agostini, Aleksej Zelezniak, Charlotte Thibeault, Moritz Pfeiffer, Stefan Hippenstiel, Andreas Hocke, Christof von Kalle, Archie Campbell, Caroline Hayward, David J. Porteous, Riccardo E. Marioni, Claudia Langenberg, Kathryn S. Lilley, Wolfgang M. Kuebler, Michael Mülleder, Christian Drosten, Norbert Suttorp, Martin Witzenrath, Florian Kurth, Leif Erik Sander, and Markus Ralser

Table S1. **Scoring of exploratory and validation COVID-19 cohorts**.

**Exploratory Cohort**						
Patient	WHO score	WHO category	Clinical assessment (Charité)	Age	Sex	Outcome
1	3	mild	mild	21	m	discharged
2	3	mild	mild	31	f	discharged
3	3	mild	mild	45	m	discharged
10	3	mild	mild	50	f	discharged
18	3	mild	mild	60	f	discharged
19	3	mild	mild	52	m	discharged
23	3	mild	mild	44	f	discharged
27	3	mild	mild	40	f	discharged
28	3	mild	mild	64	m	discharged
34	3	mild	mild	47	m	discharged
35	3	mild	mild	37	f	discharged
37	3	mild	mild	78	m	discharged
4*			severe*	37	m	
6	3	mild	severe	24	m	discharged
5	4	mild	severe	71	m	discharged
7	4	mild	severe	32	m	discharged
24	4	mild	severe	64	m	discharged
29	4	mild	severe	53	m	discharged
11	5	severe	severe	61	m	discharged
16	5	severe	severe	78	m	still hospitalized
22	5	severe	severe	62	m	discharged
26	5	severe	severe	56	f	discharged
12	6	severe	critical	75	m	discharged
20	6	severe	critical	54	f	discharged
21	6	severe	critical	52	m	discharged
8	7	severe	critical	63	m	still hospitalized
9	7	severe	critical	80	f	still hospitalized
13	7	severe	critical	71	m	death
14	7	severe	critical	81	m	death
15	7	severe	critical	54	m	death
25	7	severe	critical	62	f	still hospitalized
32	7	severe	critical	50	f	death

Validation Cohort						
Individual/ Patient	WHO score	WHO category	Clinical category	Age	Sex	Outcome
HD-1	0	uninfected	healthy	27	f	
HD-2	0	uninfected	healthy	23	m	
HD-3	0	uninfected	healthy	34	f	
HD-4	0	uninfected	healthy	31	f	
HD-5	0	uninfected	healthy	30	m	
HD-6	0	uninfected	healthy	41	f	
HD-7	0	uninfected	healthy	24	f	
HD-8	0	uninfected	healthy	27	m	
HD-9	0	uninfected	healthy	30	f	
HD-10	0	uninfected	healthy	34	f	
HD-11	0	uninfected	healthy	42	m	
HD-12	0	uninfected	healthy	30	f	
HD-13	0	uninfected	healthy	33	f	
HD-14	0	uninfected	healthy	29	f	
HD-15	0	uninfected	healthy	36	f	
49	3	mild	mild	72	f	discharged
56	3	mild	mild	58	f	discharged
65	3	mild	mild	22	m	discharged
66	3	mild	mild	75	f	discharged
75	3	mild	mild	84	m	discharged
38	4	mild	severe	70	m	discharged
48	4	mild	severe	48	m	discharged
50	4	mild	severe	78	m	discharged
63	5	severe	severe	61	m	discharged
57	6	severe	critical	55	f	discharged
60	6	severe	critical	65	m	discharged
62	6	severe	critical	72	m	discharged
33	7	severe	critical	35	f	Still hospitalized
58	7	severe	critical	26	m	Still hospitalized
59	7	severe	critical	86	m	death
61	7	severe	critical	54	m	Still hospitalized
64	7	severe	critical	57	m	Still hospitalized

*A secondary assessment triggered by blind proteome clustering (Figure S3) revealed an Influenza Type B rather than a COVID-19 infection.

Table S2. **WHO scoring for COVID-19 cases used in the study (World Health Organisation 2020).**

Patient state	Descriptor	Score
Uninfected	No clinical or virological evidence of infection	0
Ambulatory	No limitation of activities	1
	Limitation of activities	2
Hospitalised - mild disease	No oxygen therapy	3
	Oxygen by mask or nasal prongs	4
Hospitalised - severe disease	Non-invasive ventilation or high-flow oxygen	5
	Intubation and mechanical ventilation	6
	Ventilation + additional organ support (pressors, renal replacement therapy (RRT), extracorporeal membrane oygenation (ECMO))	7

Table S3. **Proteins differentially expressed depending on COVID-19 severity**.

Gene symbols	Names	Possible relevance to SARS-CoV-2 infection (speculative)
**Upregulated**		
A1BG	Alpha-1B-Glycoprotein	Function poorly understood, maybe related to hypoxia as indicated from a study in cattle (Kong et al., 2019).
ACTB;ACTG1	Actin Beta and Gamma-1	F-actin is released at the site of tissue injury and scavenged by plasma Gelsolin (DiNubile, 2008)
C1R	Complement C1r	Initiates complement activation (Hajishengallis et al., 2017; Ricklin et al., 2010, 2016).
C1S	Complement C1s	Initiates complement activation, activated by C1R (Hajishengallis et al., 2017; Ricklin et al., 2010, 2016).
C8A	Complement C8 Alpha Chain	Part of the complement system (Hajishengallis et al., 2017; Ricklin et al., 2010, 2016).
CD14	Monocyte Differentiation Antigen CD14	CD14 is primarily displayed by monocytes and macrophages and can be released in a soluble form; soluble CD14 is also produced by the liver. Primarily involved in the recognition of bacterial LPS, its upregulation might be indicative in immune response dysregulation by SARS-CoV-2. CD14 has also been implicated in a broader spectrum antigen response and lung inflammation (Anas et al., 2010). Upregulation of CD14 expression levels in monocytes was previously reported upon SARS-Cov infection (Hu et al., 2012). Cytokines can induce the release of soluble CD14 (Shive et al., 2015) and production of soluble CD14 in the liver is induced by IL-6 (Bas et al., 2004).
CFB	Complement Factor B	Part of the alternative complement pathway (Hajishengallis et al., 2017; Ricklin et al., 2010, 2016).
CFH	Complement Factor H	Modulates complement activation mainly via inhibition of the alternative complement pathway (Hajishengallis et al., 2017; Ricklin et al., 2010,

		2016).
CFI	Complement Factor I	Modulates complement activity via degradation of C3b and C4b (Hajishengallis et al., 2017; Ricklin et al., 2010, 2016).
CRP	C-Reactive Protein	Acute phase response protein, strongly upregulated in COVID-19 (Shi et al., 2020).
FGA, FGB, FGG	Fibrinogen Alpha, Beta and Gamma Chains	Fibrinogen levels are elevated in acute-phase response (Jain et al., 2011), upregulated in COVID-19 (Shi et al., 2020).
HP	Haptoglobin	Elevated in acute-phase response (Jain et al., 2011). Of note, HP expression is known to be promoted by IL-6 (Li et al., 2020), which is elevated in severe COVID-19 (Ruan et al., 2020).
ITIH3	Inter-Alpha-Trypsin Inhibitor Heavy Chain 3	Involved in inflammatory response to trauma (Hamm et al., 2008)
ITIH4	Inter-Alpha-Trypsin Inhibitor Heavy Chain 4	Involved in inflammatory response to trauma (Hamm et al., 2008). Production of ITIH4 in the liver is upregulated by IL-6 (Bhanumathy et al., 2002).
LBP	Lipopolysaccharide Binding Protein	Recognises bacterial LPS, upregulation might be indicative in immune response dysregulation by SARS-CoV-2.
LGALS3BP	Galectin 3 Binding Protein	Upregulated in viral infections, promotes inflammation (Xu et al., 2019), has been shown to induce IL-6 expression (Silverman et al., 2012).
LRG1	Leucine-Rich Alpha-2-Glycoprotein	LRG1 expression is induced by IL-6 (Shirai et al., 2009), which is elevated in severe COVID-19 (Ruan et al., 2020). LRG1 promotes angiogenesis (Wang et al., 2013) and has been reported to be associated with local inflammation (Naka and Fujimoto, 2018). LRG1 is known to promote lung fibrosis (Honda et al., 2017). Secretion of LRG1 by neutrophils upon activation has been reported (Druhan et al., 2017). Of note, neutrophil-lymphocyte-ratio is elevated in COVID-19 (Qin et al., 2020).
SAA1 and SAA1;SAA2	Serum Amyloid A1 and A2	SAA1 and SAA2 are markers of inflammatory response and tissue injury (Sack, 2018).

		Expression is known to be induced by IL-6 (Hagihara et al., 2004).
SERPINA10	Protein Z-Dependent Protease Inhibitor	Acts in complex with Protein Z to inhibit the F10a coagulation factor (Almawi et al., 2013), while coagulation has been observed to be increased in severe COVID-19 (Zhou et al., 2020).
**Downregulated**		
ALB	Albumin	Low levels associated with acute-phase response (Soeters et al., 2019) and observed in critical COVID-19 (Zhou et al., 2020).
APOA1	Apolipoprotein A1	Major component of the High-Density Lipoprotein (HDL) complex, which is a modulator of innate immune response and inflammation (Fotakis et al., 2019; Gordon et al., 2011; Macpherson et al., 2019; White et al., 2017). Although it might be that APOA1 levels drop upon the infection, we can also speculate that low APOA1 levels are a risk factor for COVID-19. Notably, We observe clear correlation of APOA1 with HDL cholesterol and only weak negative correlation with age (Figure S6). Of note, decreased APOA1 levels have been associated with poor prognosis in systemic inflammatory response (Kumaraswamy et al., 2012; Sirniö et al., 2017; Tani et al., 2016).
APOC1	Apolipoprotein C1	Part of multiple apolipoprotein complexes, has links to immune response and inflammation (Fuior and Gafencu, 2019).
GSN	Gelsolin	Low levels of plasma gelsolin are associated with inflammation. One of the primary mechanisms leading to this is believed to be the recruitment of gelsolin, which has actin recycling function, to the sites of tissue injury, depleting its plasma levels. Of note, we observed increased serum levels of the ACTB;ACTG1 protein group in severe and critical COVID-19 cases. Importantly, plasma gelsolin is a modulator of inflammation, which carries a protective function (DiNubile, 2008; Li et al., 2012). Low plasma gelsolin is a marker of poor prognosis in various pathological conditions, including diabetes (Khatri et al., 2014), cancers (Asare-Werehene et al., 2019; Stock et al., 2015) and sepsis (Lee et al., 2007), leading to

		suggestions and animal tests for its therapeutic use. Of note, treatment with gelsolin has been observed to decrease IL-6 levels in mice (Cheng et al., 2017). Gelsolin treatment has been suggested to promote epithelial repair (Wittmann et al., 2018).
TF	Transferrin	Iron carrier, observed to decrease in acute phase response (Jain et al., 2011).

Table S4. **Date of symptom onset and blood sampling for the patients from the exploratory cohort.**

Patient	Onset of symptoms	Admission to hospital	blood sampling
1	15/02/2020	01/03/2020	02/03,04/03,06/03,07/03,09/03,10/03,11/03,12/03,13/03,16/03,18/03,20/03,23/03,23/03,25/03,27/03
2	25/02/2020	02/03/2020	04/03,06/03,07/03,08/03,09/03
3	29/02/2020	11/03/2020	11/03,12/03,13/03,16/03,18/03,20/03,23/03,23/03,25/03
5	28/02/2020	11/03/2020	12/03,13/03,16/03,18/03
6	11/03/2020	12/03/2020	13/03,16/03,18/03
7	08/03/2020	14/03/2020	16/03,19/03,20/03,23/03,25/03
8	21/02/2020	15/03/20	20/03,23/03,25/03,27/03
9	12/03/2020	16/03/2020	17/03,17/03,20/03,23/03,25/03,27/03
10	09/03/2020	17/03/2020	19/03,20/03
11	08/03/2020	17/03/2020	20/03,23/03,25/03,27/03
12	21/02/2020	18/03/2020	17/03,20/03,23/03,25/03,27/03
13	13/03/2020	19/03/2020	20/03,23/03,25/03,27/03
14	15/03/2020	21/03/2020	23/03
15	NA	17/03/2020	23/03,25/03
16	19/03/2020	22/03/2020	23/03,25/03,27/03

18	04/03/2020	10/30/2020	25/03
19	14/03/2020	19/03/2020	23/03,25/03
20	12/03/2020	20/30/2020	23/03,25/03,27/03
21	12/03/2020	20/03/2020	23/03,25/03,27/03
22	09/03/2020	18/03/2020	23/03,25/03,27/03
23	NA	23/03/2020	23/03,25/03,27/03
24	06/03/2020	21/03/2020	25/03,27/03
25	NA	23/03/2020	25/03,27/03
26	15/03/2020	24/03/2020	25/03,27/03
27	12/03/2020	12/03/2020	25/03
28	20/03/2020	23/03/2020	25/03
29	13/03/2020	23/03/2020	25/03,27/03
32	NA	21/03/2020	27/03
34	21/03/2020	26/03/2020	27/03
35	15/03/2020	25/03/2020	27/03
37	10/03/2020	24/03/2020	27/03

Table S5. **List of FDA approved biomarkers, quantified with ultra-high-throughput serum proteomics.**

**Protein Name**
Albumin<br>α-1-acid glycoprotein<br>α-1-antitrypsin<br>α-2-antiplasmin<br>α-2-HS-glycoprotein<br>α-2-macroglobulin<br>Antithrombin III<br>Apolipoprotein B<br>β-2-microglobulin

β-Thromboglobulin
Biotinidase
Ceruloplasmin
Cholinesterase
Complement C1
Complement C1Q
Complement C3
Complement C4
Complement C5
CRP
Cystatin C
Factor IX antigen
Factor X
Factor XIII
Fibrinogen
Fibronectin
Haptoglobin
Hemopexin
IgG
IgM
Insulin-like growth factor II
IGFBP-3
κ light chains
λ light chains
Lipoprotein (a)
Lysozyme
Plasminogen
Prealbumin / transthyretin
Properdin factor B
Protein S
Retinol binding protein
Sex hormone-binding globulin
Thyroxine binding globulin
Transferrin receptor
Von Willebrand factor

Table S6. **Lower and upper m/z limits of the SWATH precursor selection windows used.**

Lower m/z limit	Upper m/z limit	CE Spread
449.5	468.9	5
467.9	486.4	5
485.4	503.3	5
502.3	519.8	5
518.8	535.7	5
534.7	550.9	5
549.9	565.5	5
564.5	579.7	5
578.7	594	5
593	607.9	5
606.9	621.9	5
620.9	635.8	5
634.8	649.7	5
648.7	663.3	5
662.3	676.3	5
675.3	689.6	5
688.6	702.9	5
701.9	716.8	5
715.8	732.3	5
731.3	748.9	5
747.9	766.4	5
765.4	784.2	5
783.2	803	5

802	824.7	5
823.7	849.6	5

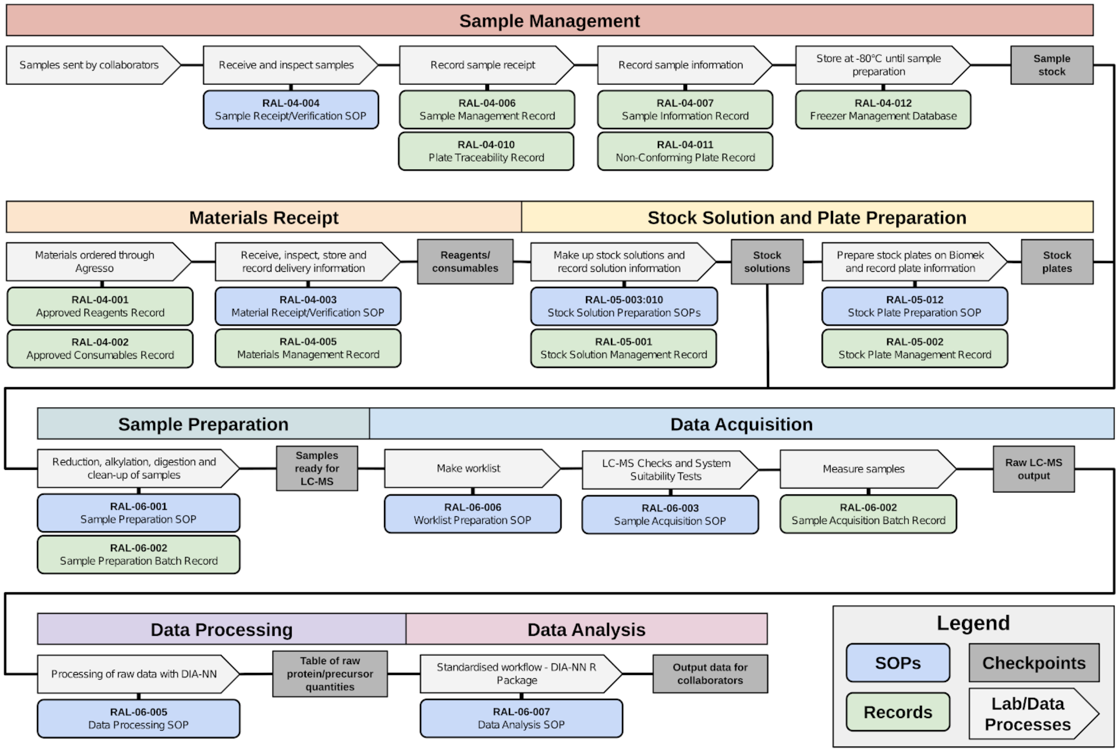

Figure S1, Related to Figure 1. **Detailed overview of the high-throughput proteomics workflow.** This figure details the documentation and management tasks orienting on ISO13485 standardisation for medical devices as reference.

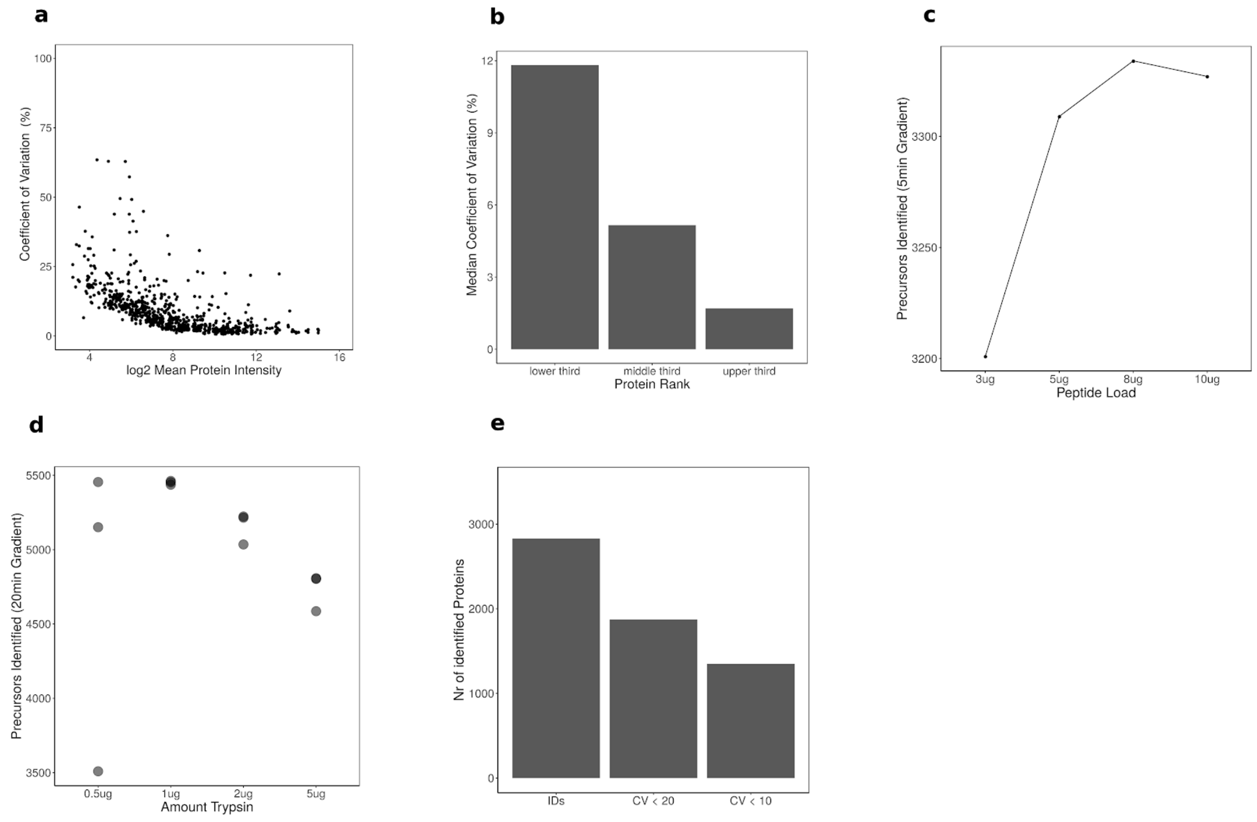

Figure S2. **Quality parameters of protein quantification in ultra-high-throughput proteomics. a**. Coefficient of Variation (%) as a function of log2 mean protein intensity for 39 repeated injections of a pooled whole-serum tryptic digest that were run over a period of > 4 days, to test for instrument stability.. **b.** Median CV value for low, middle, and high-intensity proteins in the 39 repeated injections. **c.** Number of precursors identified in 5-min high-flow SWATH runs with different amounts of serum proteins injected. **d.** Number of precursors identified in a plasma sample with different amounts of trypsin used for digestion, run with a 20-min water to acetonitrile gradient. **e.** Proteins identified in triplicate injections of a mammalian (K562) cell lysate using the 5-minute high-flow SWATH runs presented in this study. Total number of unique Proteins (1% FDR), number of unique Proteins quantified with less than 20% coefficient of variation (CV) and less than 10% CV are shown.

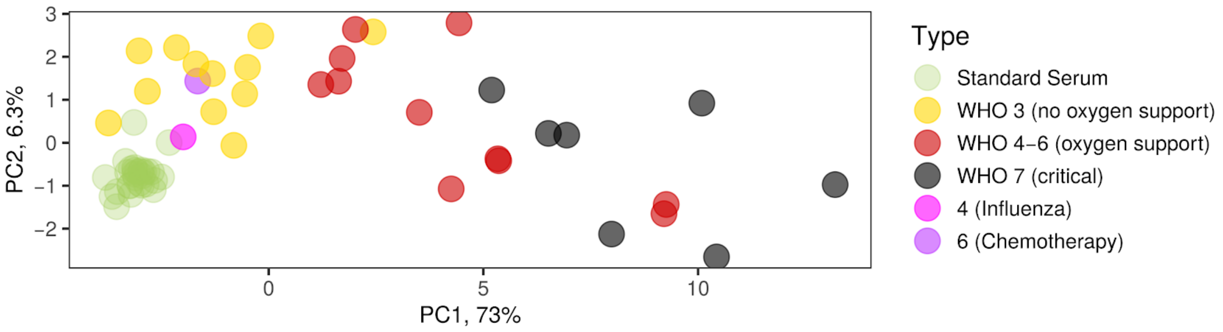

Figure S3: **Clustering of untargeted plasma proteomes led to the reclassification of two study participants.** Principal component analysis based on proteins differentially expressed depending on COVID-19 severity grading according to WHO. Patients 4 and 6 were initially clinically assessed as “severe” (Braun et al, 2020) but clustered with patients that suffered from a mild form of COVID-19. A retrospective assessment revealed that patient 6 had received R-CHOP chemotherapy 10 days prior to study inclusion and patient 4 was in fact suffering from type B influenza rather than SARS-CoV-2 infection. Patient 6 was categorized as mild by using the WHO ordinal outcome scale of clinical improvement (World Health Organisation, 2020) and Patient 4 was removed from the analysis. Please note that the PCA transformation is slightly different from that in Figure 4b, as the addition of two extra samples for patient 4 led to slightly different protein quantities being calculated for all the samples by the MaxLFQ algorithm (Cox et al., 2014).

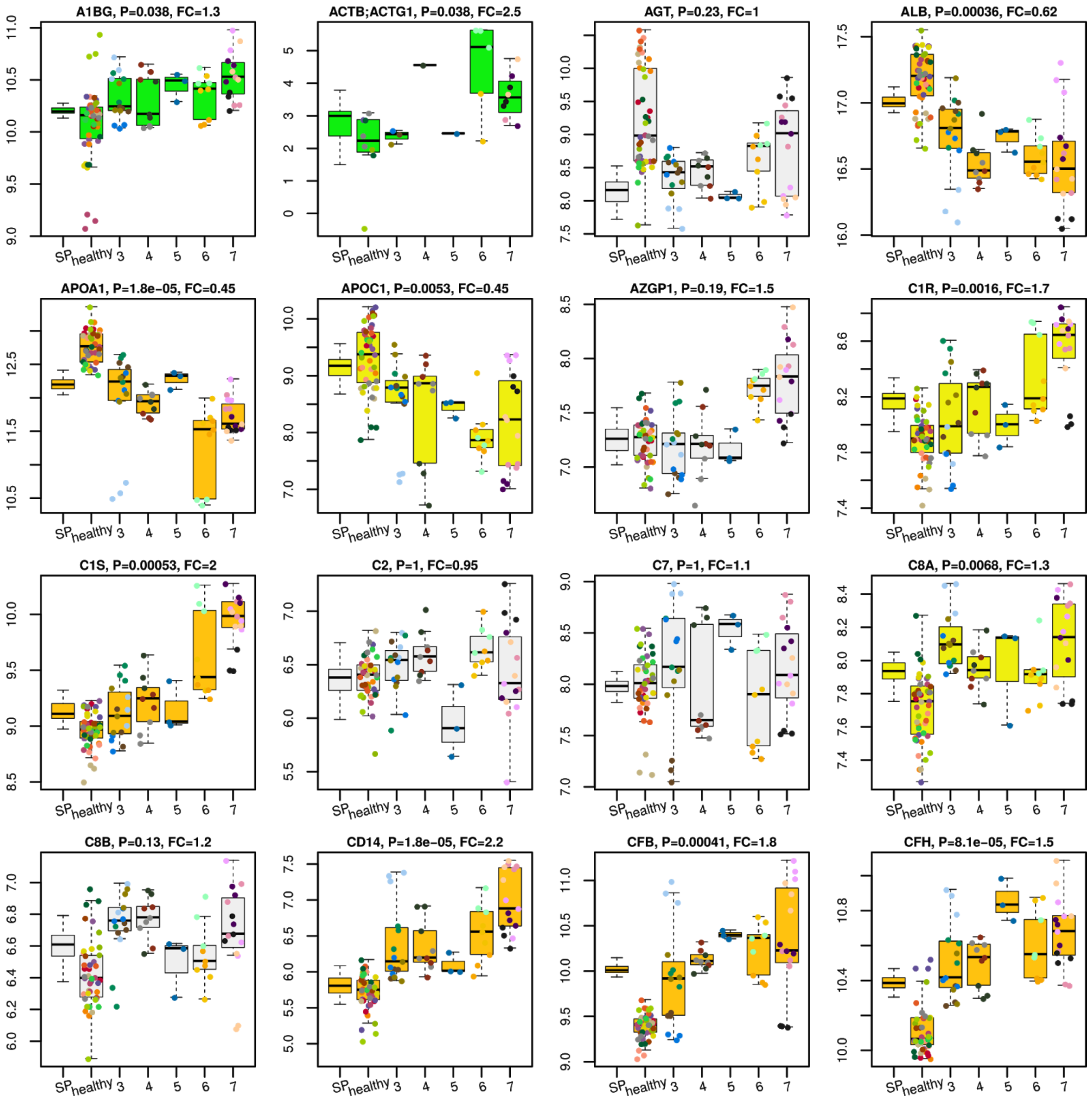
A1BG, P=0.038, FC=1.3
ACTB;ACTG1, P=0.038, FC=2.5
AGT, P=0.23, FC=1
ALB, P=0.00036, FC=0.62
APOA1, P=1.8e-05, FC=0.45
APOC1, P=0.0053, FC=0.45
AZGP1, P=0.19, FC=1.5
C1R, P=0.0016, FC=1.7
C1S, P=0.00053, FC=2
C2, P=1, FC=0.95
C7, P=1, FC=1.1
C8A, P=0.0068, FC=1.3
C8B, P=0.13, FC=1.2
CD14, P=1.8e-05, FC=2.2
CFB, P=0.00041, FC=1.8
CFH, P=8.1e-05, FC=1.5
SP
healthy
3
4
5
6
7

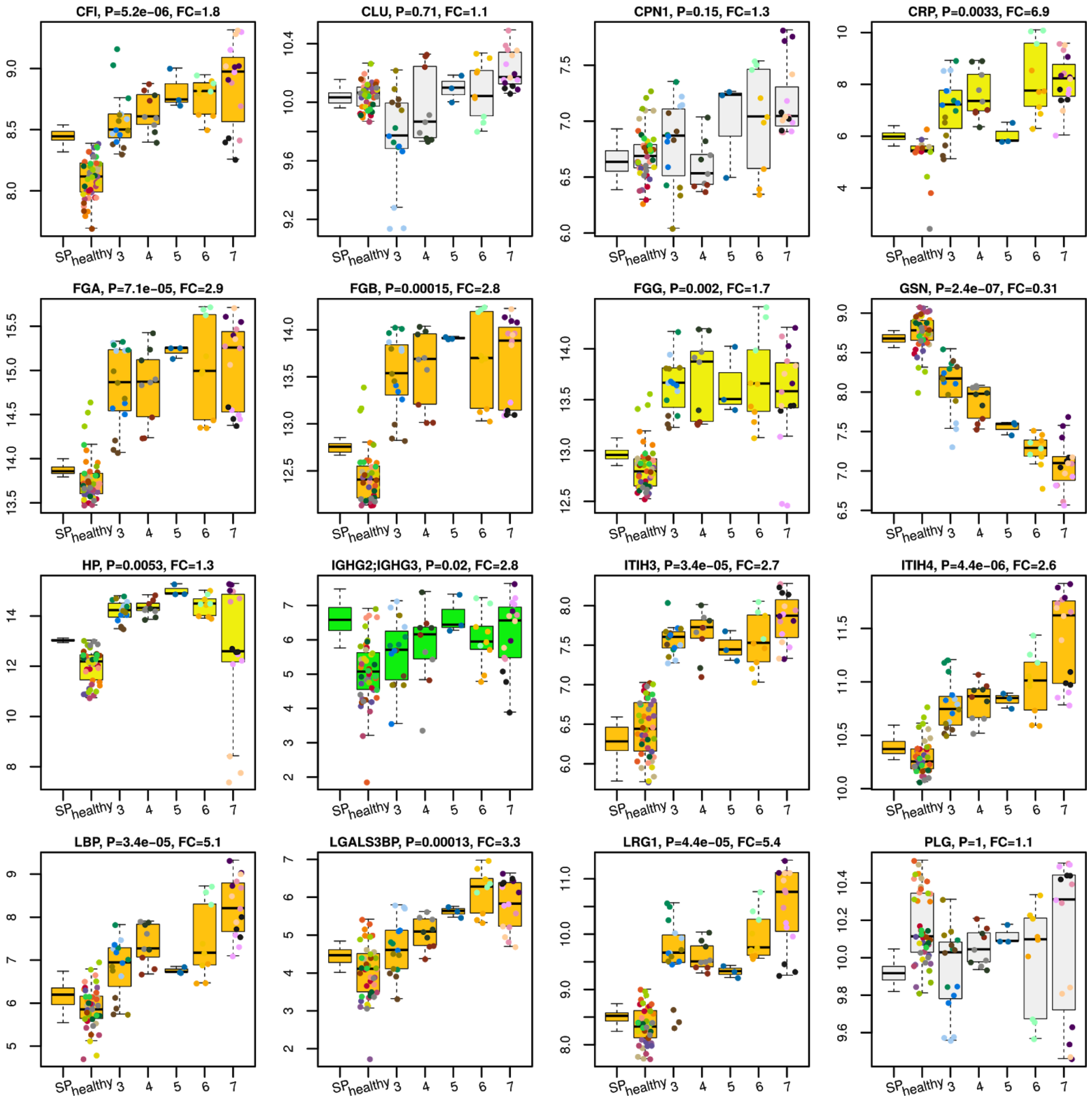
CFI, P=5.2e−06, FC=1.8
CLU, P=0.71, FC=1.1
CPN1, P=0.15, FC=1.3
CRP, P=0.0033, FC=6.9
FGA, P=7.1e−05, FC=2.9
FGB, P=0.00015, FC=2.8
FGG, P=0.002, FC=1.7
GSN, P=2.4e−07, FC=0.31
HP, P=0.0053, FC=1.3
IGHG2;IGHG3, P=0.02, FC=2.8
ITIH3, P=3.4e−05, FC=2.7
ITIH4, P=4.4e−06, FC=2.6
LBP, P=3.4e−05, FC=5.1
LGALS3BP, P=0.00013, FC=3.3
LRG1, P=4.4e−05, FC=5.4
PLG, P=1, FC=1.1
SP
healthy
3
4
5
6
7

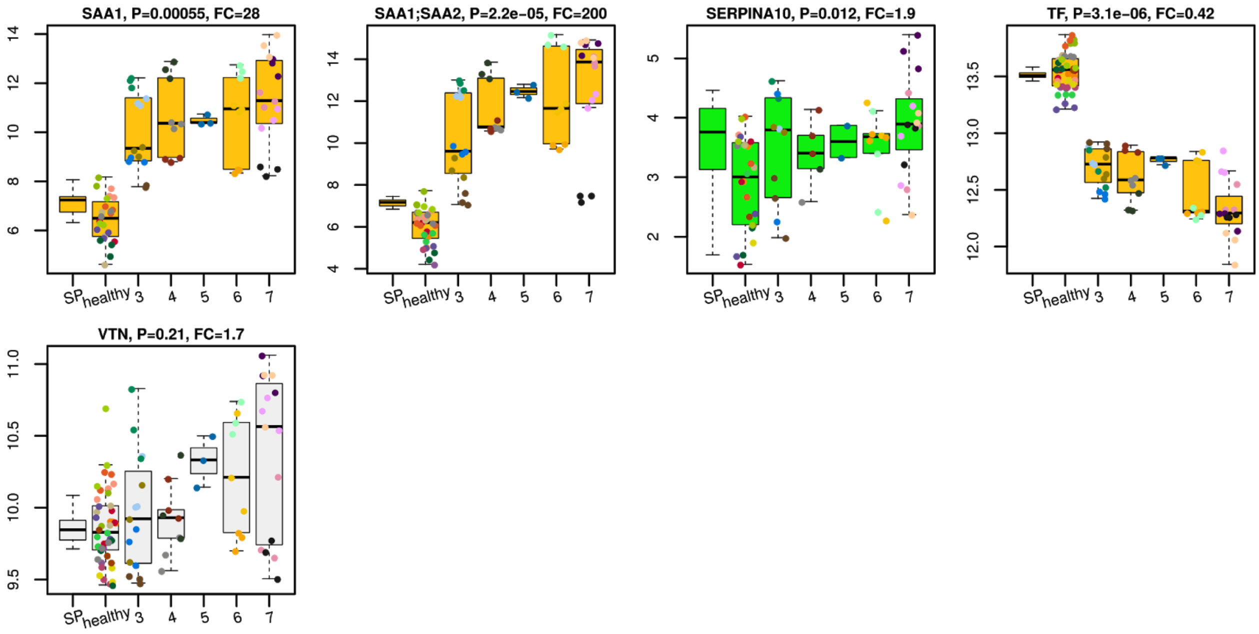

Figure S4. **Proteomes in mild, severe and critical SARS-CoV-2 infection for patients in the validation cohort**. Differentially expressed proteins are highlighted with colours depending on the multiple testing-corrected p-values: green: P < 0.05, yellow: P < 0.01, orange: P < 0.001.

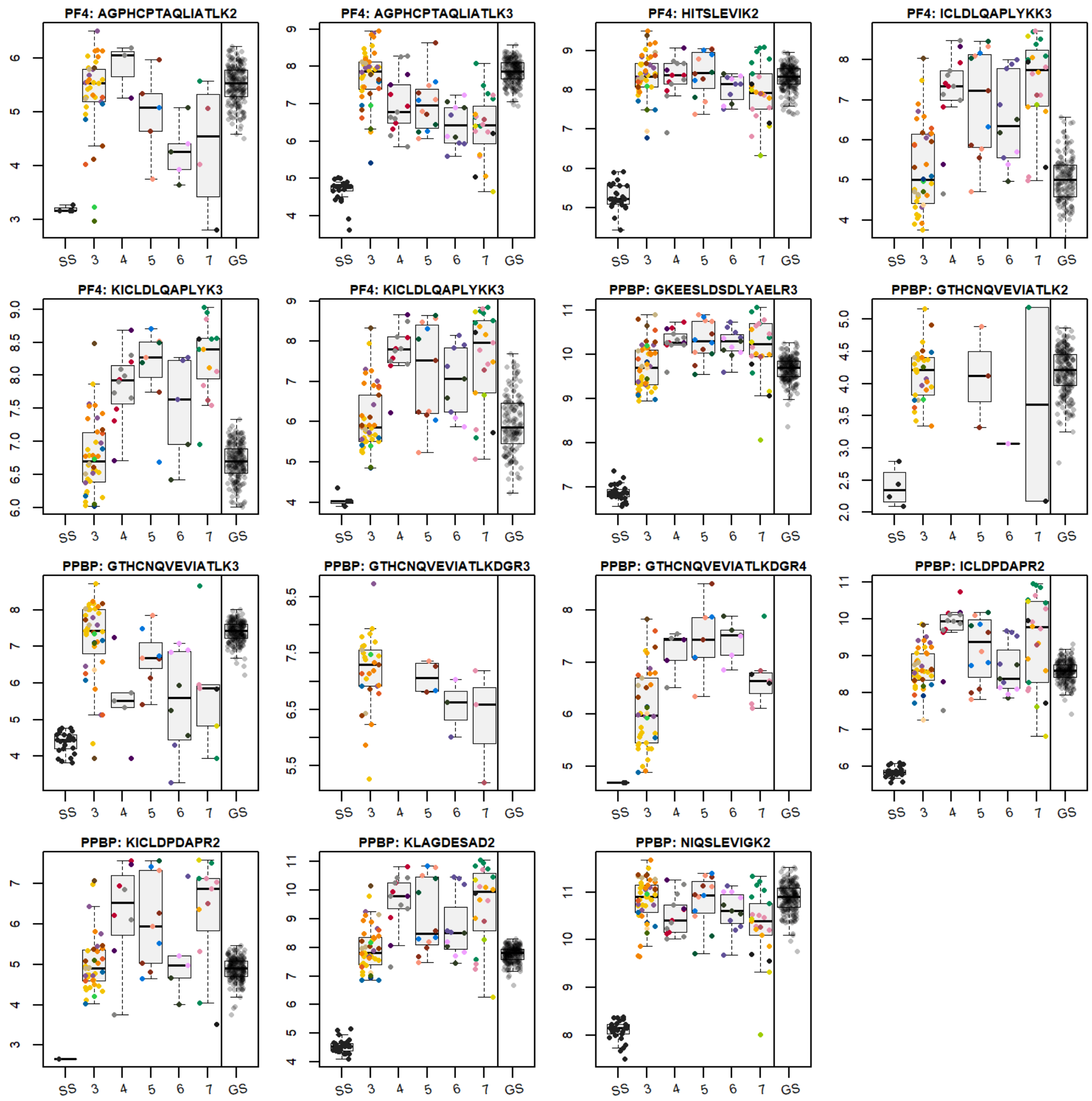

Figure S5. **Differential response of peptide precursors mapping to PF4 and PPBP**. Boxplots are presented for log_2-transformed peptide precursor quantities. The peptide precursor charge is indicated with a number after the amino acid sequence. All cysteines were considered carbamidomethylated. Boxplots are drawn based on individual measurements (multiple timepoints per patient), which are indicated with points (different colours for different patients, ordered from left to right by the time taken). Standard Serum sample preparation controls are indicated with “SS” and represent the technical variability (sample preparation + LC-MS). The last box represents the variability seen in the GS study; as the absolute quantities from the two studies cannot be compared directly (the data was acquired on different LC-MS setups), to

simplify the visual assessment of the variability, the median of GS quantities was matched to the median of WHO grade 3 (no oxygen support) COVID-19 cases.

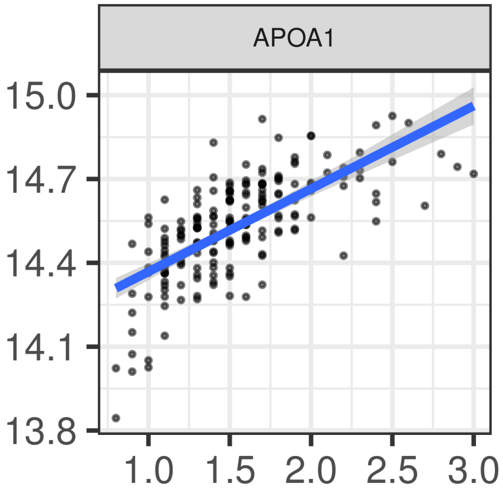

Figure S6. **Log_2-transformed serum levels of Apolipoprotein A1 (APOA1; arbitrary units) plotted against the HDL-Cholesterol concentration (mmol/L) in the GS study**.

Figure S7 (following 18 pages). **Proteomes in mild, severe and critical SARS-CoV-2 infection (complete list)**. Differentially expressed proteins are highlighted with colours depending on the multiple testing-corrected p-values: green: $P < 0.05$, yellow: $P < 0.01$, orange: $P < 0.001$.

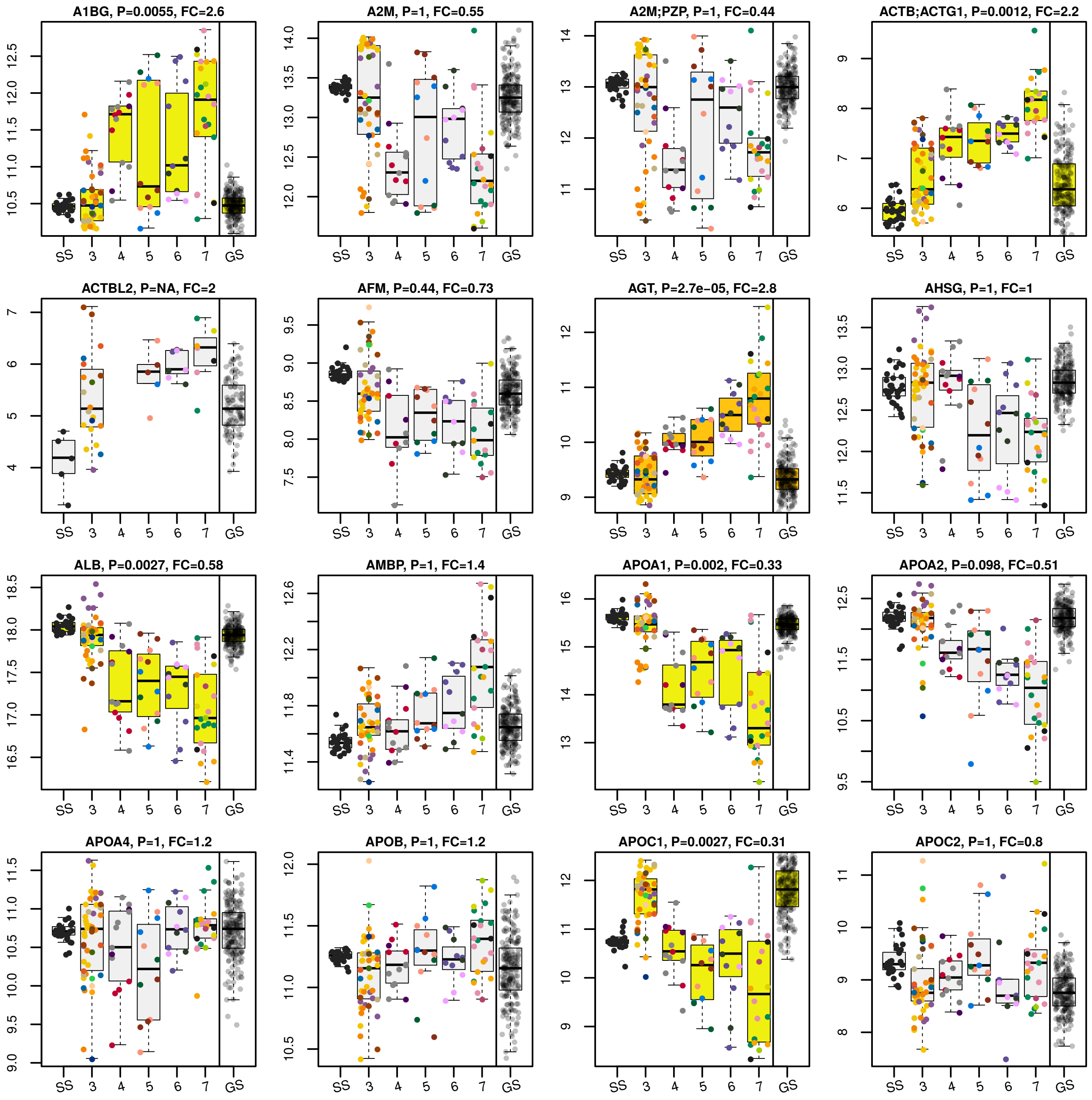
A1BG, P=0.0055, FC=2.6
A2M, P=1, FC=0.55
A2M;PZP, P=1, FC=0.44
ACTB;ACTG1, P=0.0012, FC=2.2
ACTBL2, P=NA, FC=2
AFM, P=0.44, FC=0.73
AGT, P=2.7e-05, FC=2.8
AHSG, P=1, FC=1
ALB, P=0.0027, FC=0.58
AMBP, P=1, FC=1.4
APOA1, P=0.002, FC=0.33
APOA2, P=0.098, FC=0.51
APOA4, P=1, FC=1.2
APOB, P=1, FC=1.2
APOC1, P=0.0027, FC=0.31
APOC2, P=1, FC=0.8
SS
3
4
5
6
7
GS

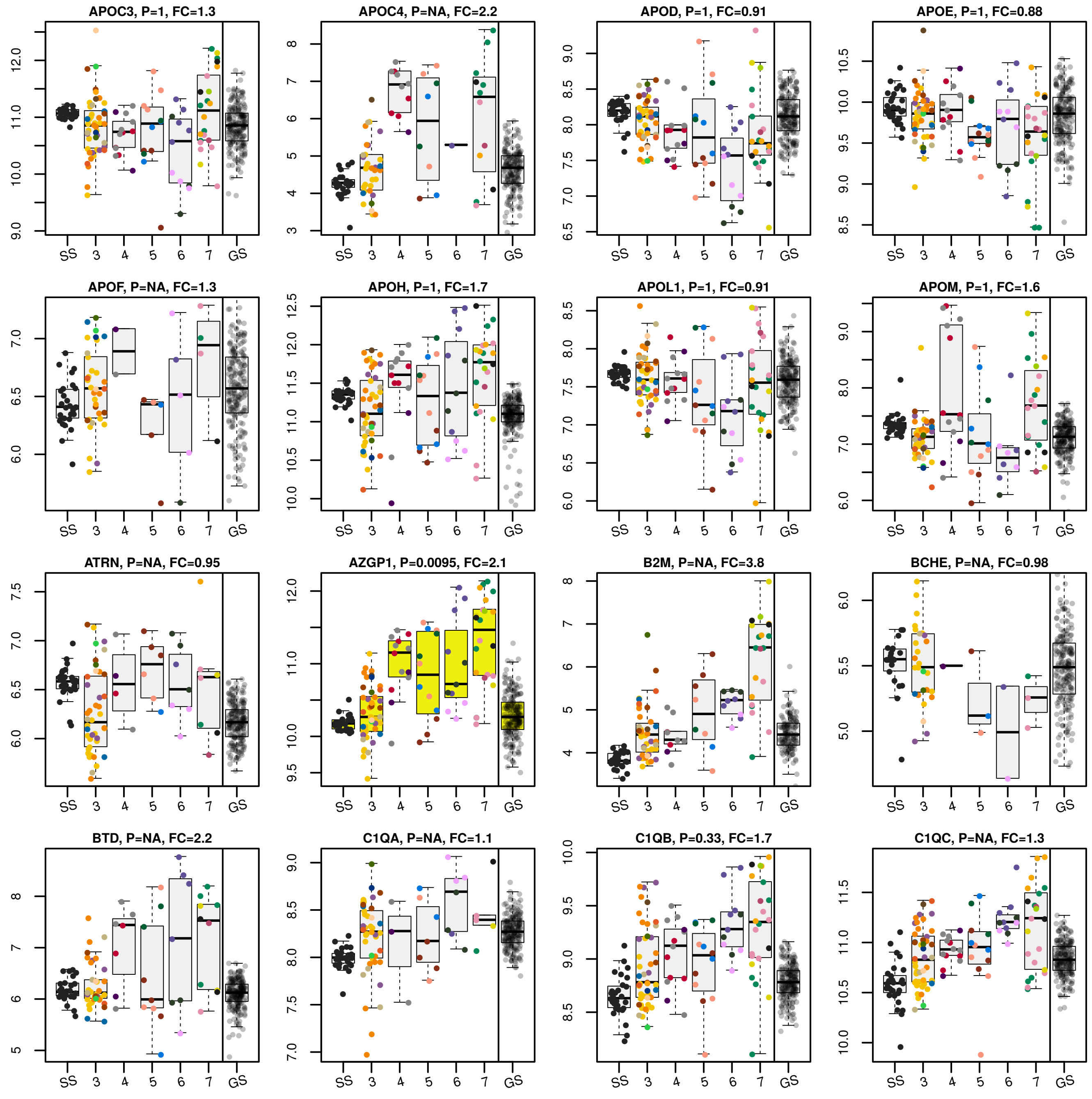
APOC3, P=1, FC=1.3
APOC4, P=NA, FC=2.2
APOD, P=1, FC=0.91
APOE, P=1, FC=0.88
APOF, P=NA, FC=1.3
APOH, P=1, FC=1.7
APOL1, P=1, FC=0.91
APOM, P=1, FC=1.6
ATRN, P=NA, FC=0.95
AZGP1, P=0.0095, FC=2.1
B2M, P=NA, FC=3.8
BCHE, P=NA, FC=0.98
BTD, P=NA, FC=2.2
C1QA, P=NA, FC=1.1
C1QB, P=0.33, FC=1.7
C1QC, P=NA, FC=1.3
SS
3
4
5
6
7
GS

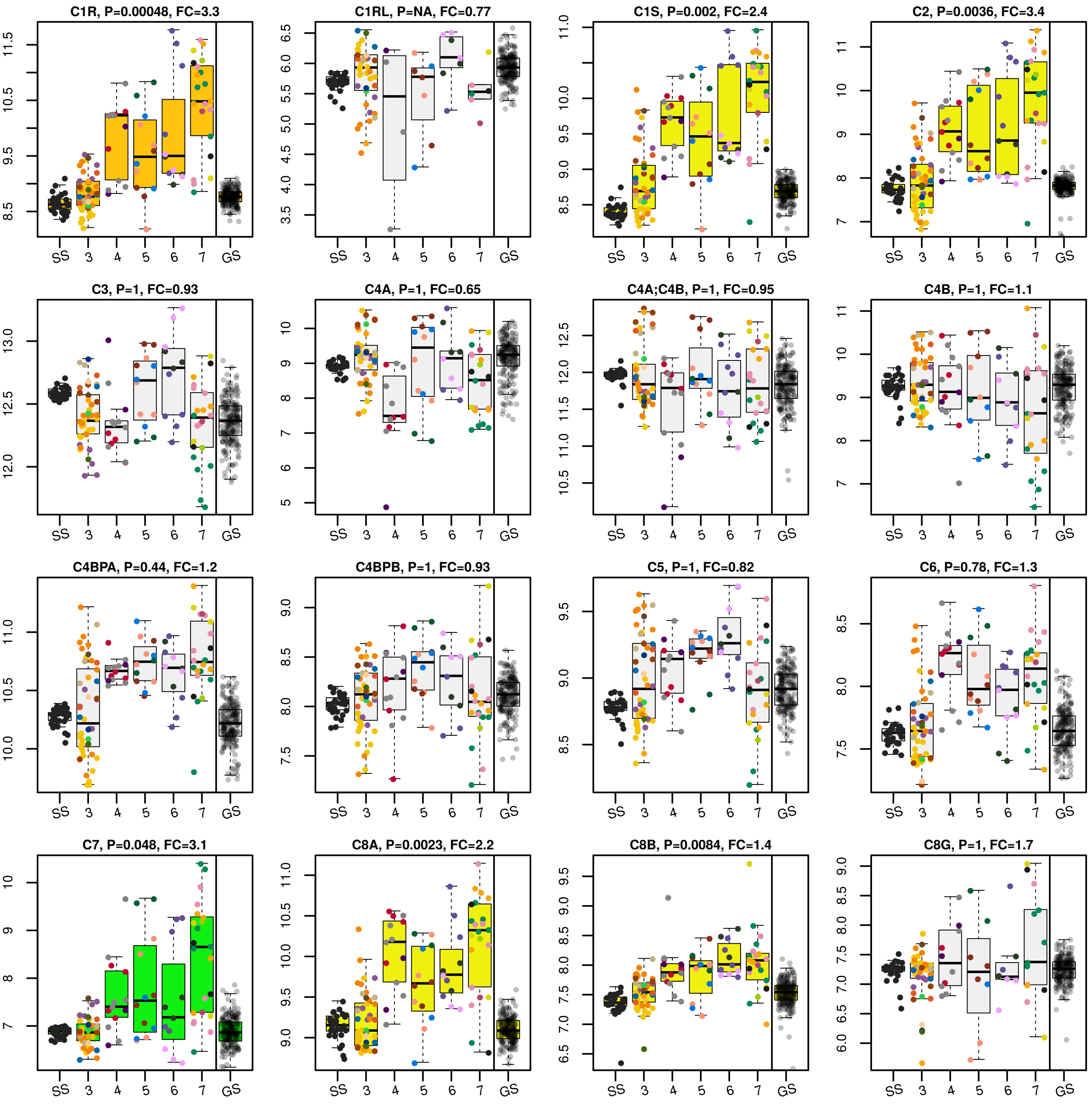

C1R, P=0.00048, FC=3.3
C1RL, P=NA, FC=0.77
C1S, P=0.002, FC=2.4
C2, P=0.0036, FC=3.4
C3, P=1, FC=0.93
C4A, P=1, FC=0.65
C4A;C4B, P=1, FC=0.95
C4B, P=1, FC=1.1
C4BPA, P=0.44, FC=1.2
C4BPB, P=1, FC=0.93
C5, P=1, FC=0.82
C6, P=0.78, FC=1.3
C7, P=0.048, FC=3.1
C8A, P=0.0023, FC=2.2
C8B, P=0.0084, FC=1.4
C8G, P=1, FC=1.7
SS
3
4
5
6
7
GS

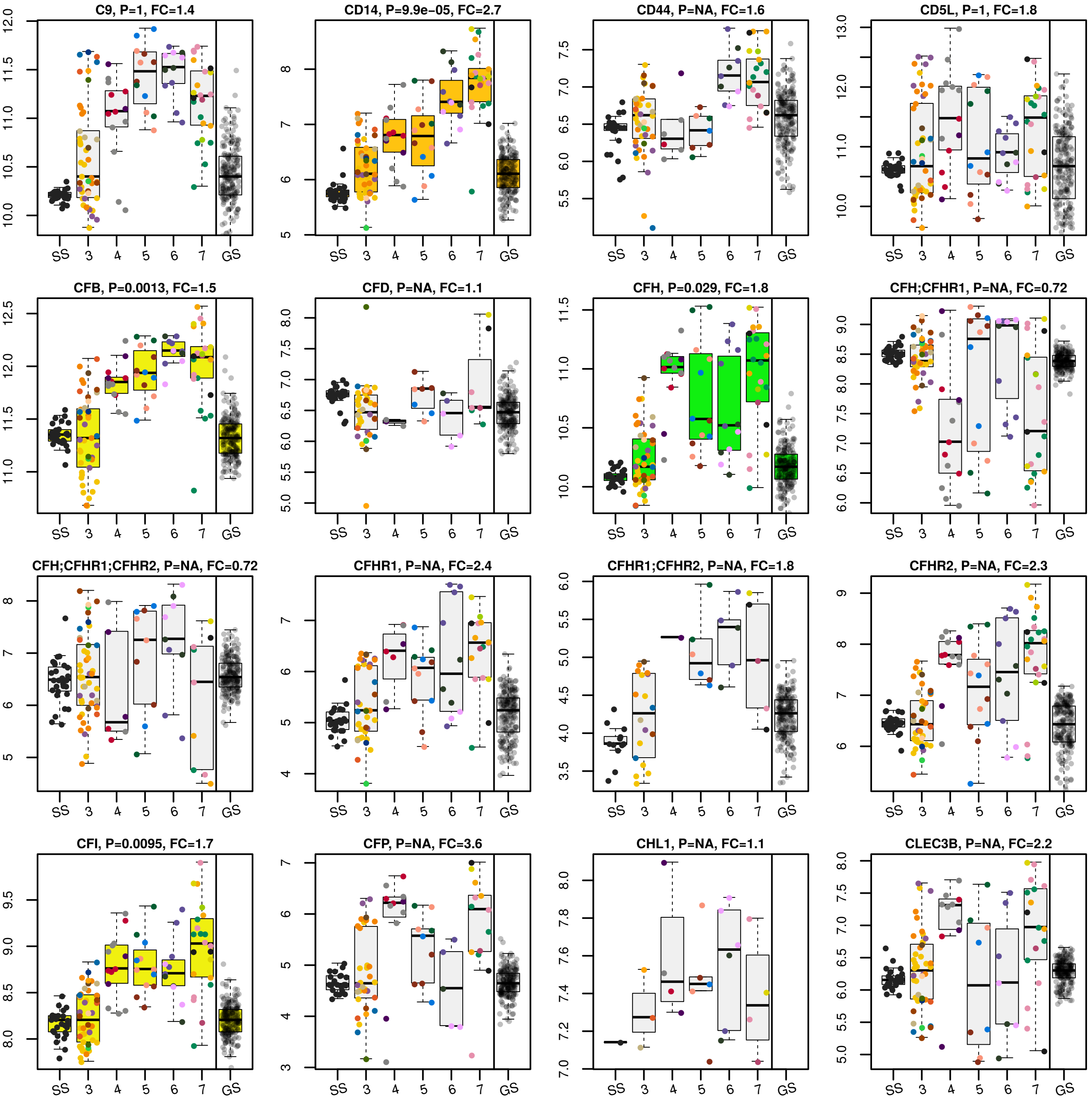
C9, P=1, FC=1.4
CD14, P=9.9e−05, FC=2.7
CD44, P=NA, FC=1.6
CD5L, P=1, FC=1.8
CFB, P=0.0013, FC=1.5
CFD, P=NA, FC=1.1
CFH, P=0.029, FC=1.8
CFH;CFHR1, P=NA, FC=0.72
CFH;CFHR1;CFHR2, P=NA, FC=0.72
CFHR1, P=NA, FC=2.4
CFHR1;CFHR2, P=NA, FC=1.8
CFHR2, P=NA, FC=2.3
CFI, P=0.0095, FC=1.7
CFP, P=NA, FC=3.6
CHL1, P=NA, FC=1.1
CLEC3B, P=NA, FC=2.2
SS
3
4
5
6
7
GS

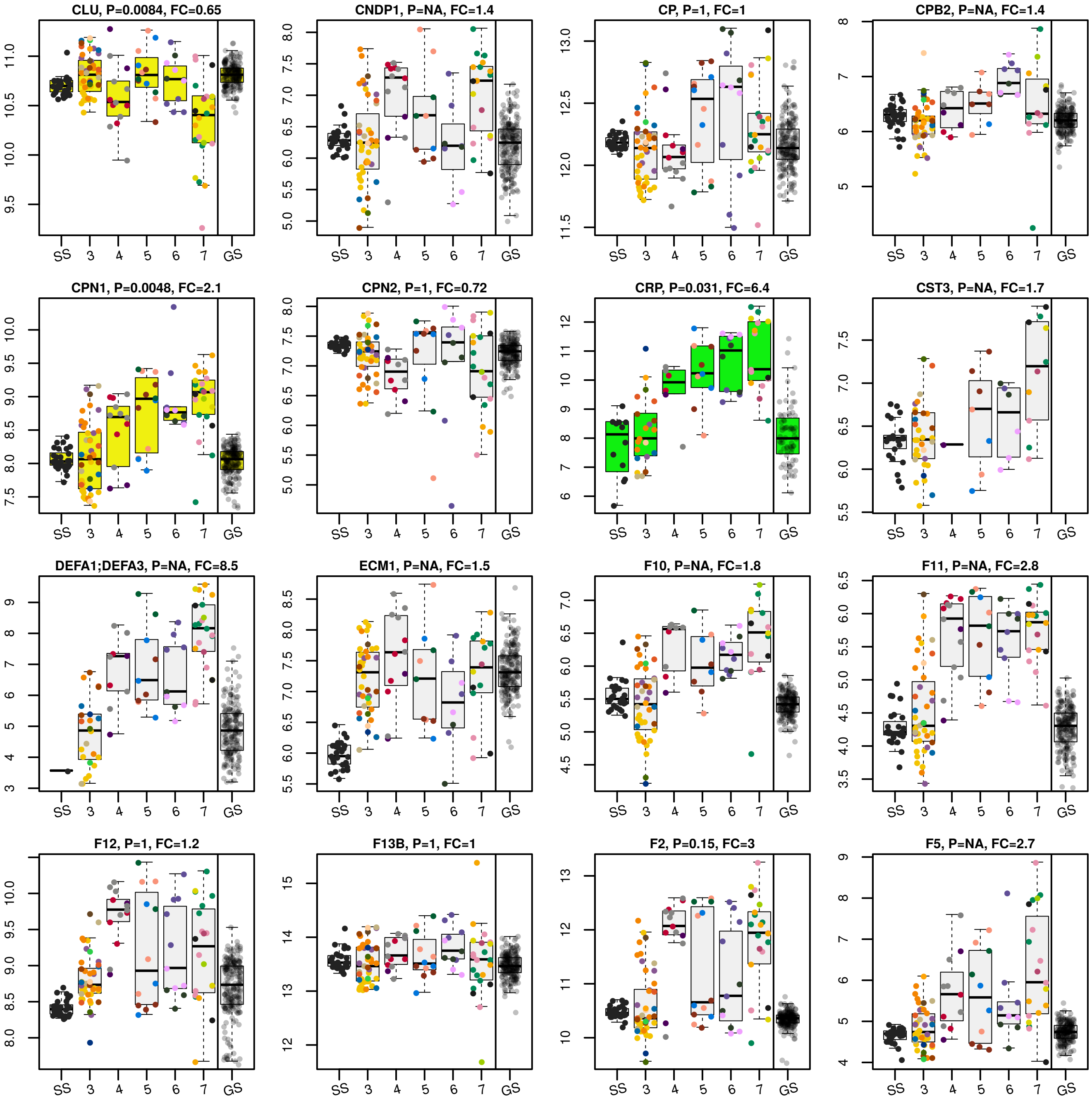
CLU, P=0.0084, FC=0.65
CNDP1, P=NA, FC=1.4
CP, P=1, FC=1
CPB2, P=NA, FC=1.4
CPN1, P=0.0048, FC=2.1
CPN2, P=1, FC=0.72
CRP, P=0.031, FC=6.4
CST3, P=NA, FC=1.7
DEFA1;DEFA3, P=NA, FC=8.5
ECM1, P=NA, FC=1.5
F10, P=NA, FC=1.8
F11, P=NA, FC=2.8
F12, P=1, FC=1.2
F13B, P=1, FC=1
F2, P=0.15, FC=3
F5, P=NA, FC=2.7
SS 3 4 5 6 7 GS

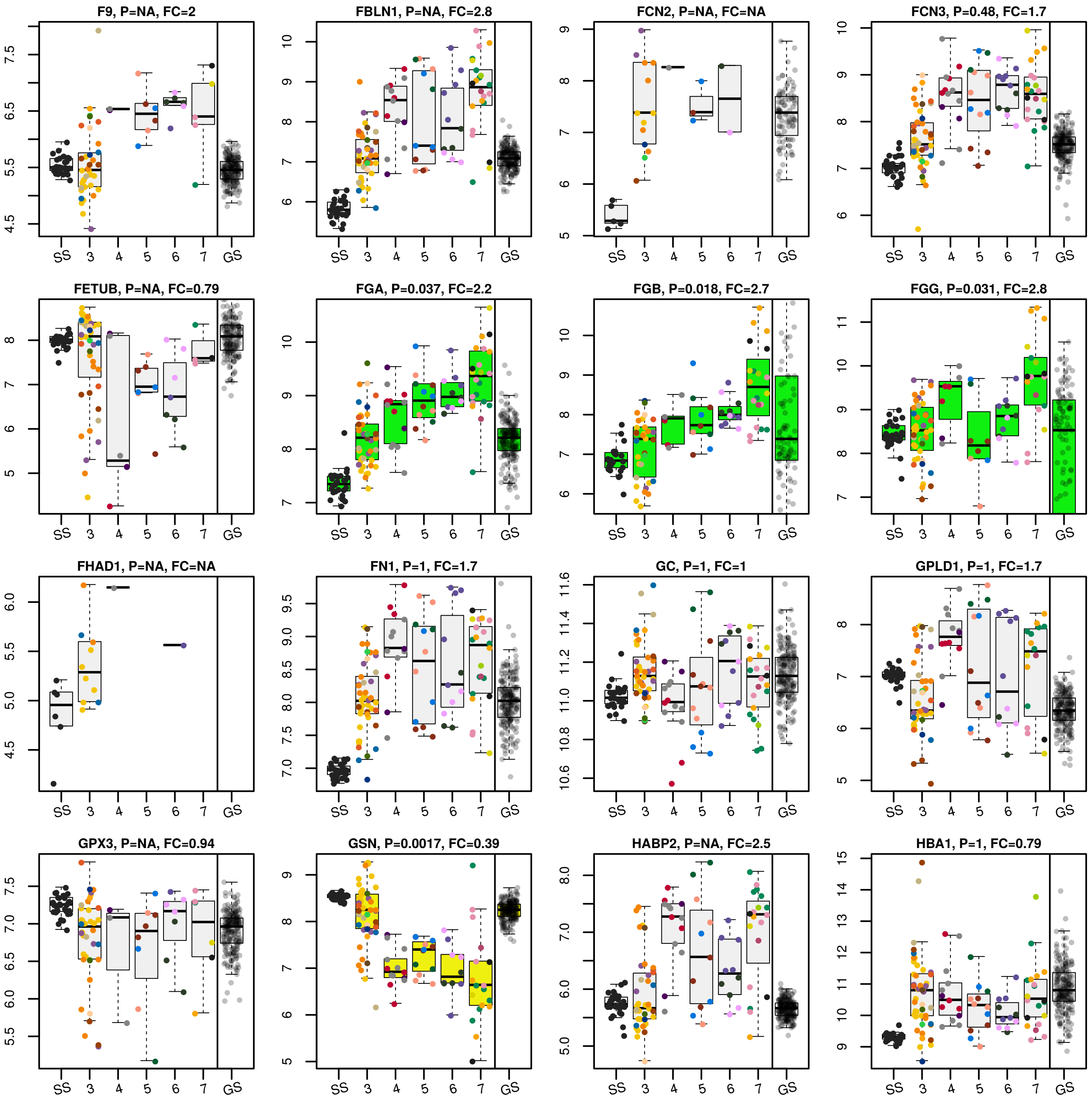
F9, P=NA, FC=2
FBLN1, P=NA, FC=2.8
FCN2, P=NA, FC=NA
FCN3, P=0.48, FC=1.7
FETUB, P=NA, FC=0.79
FGA, P=0.037, FC=2.2
FGB, P=0.018, FC=2.7
FGG, P=0.031, FC=2.8
FHAD1, P=NA, FC=NA
FN1, P=1, FC=1.7
GC, P=1, FC=1
GPLD1, P=1, FC=1.7
GPX3, P=NA, FC=0.94
GSN, P=0.0017, FC=0.39
HABP2, P=NA, FC=2.5
HBA1, P=1, FC=0.79
SS
3
4
5
6
7
GS

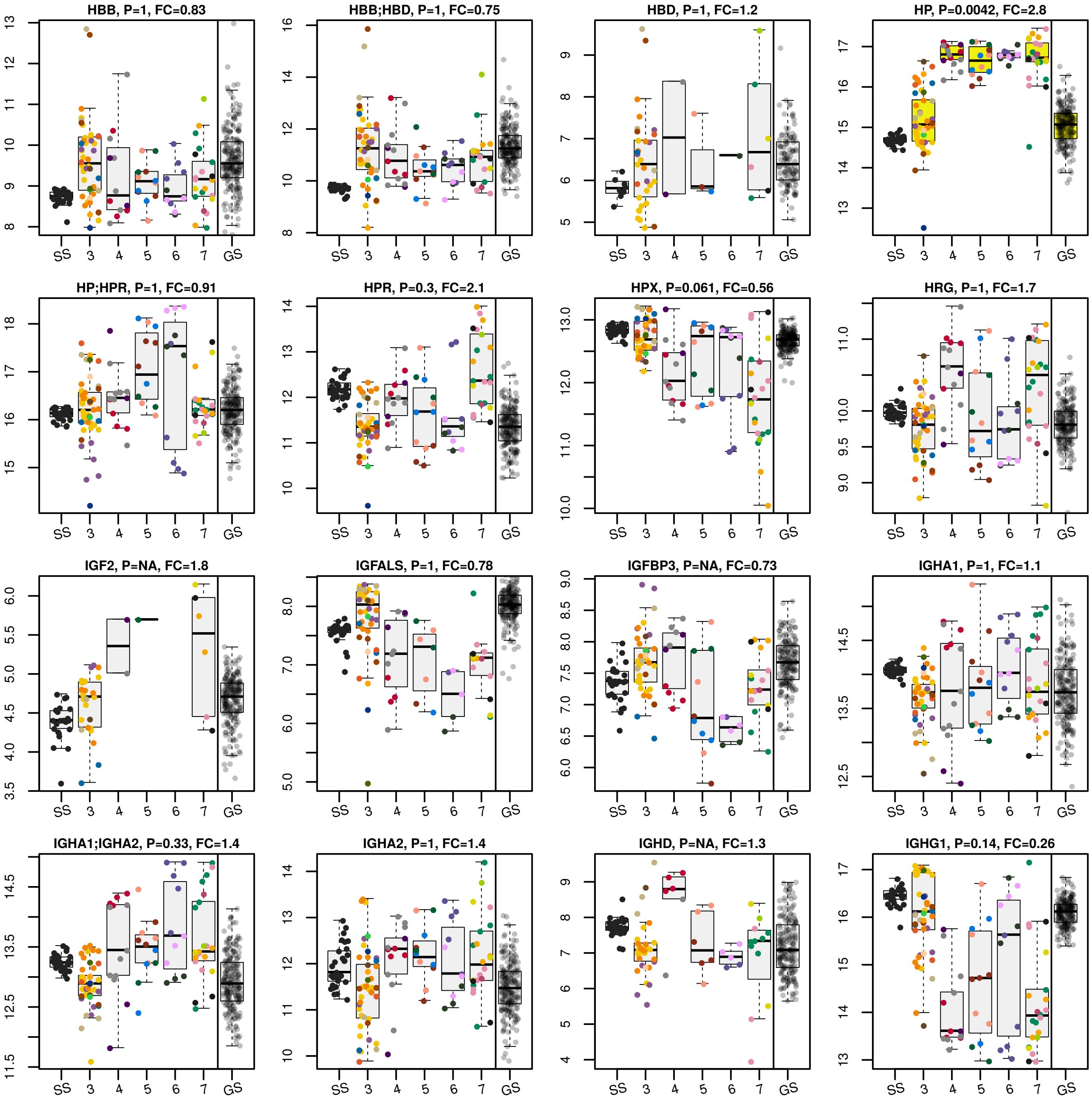
HBB, P=1, FC=0.83
HBB;HBD, P=1, FC=0.75
HBD, P=1, FC=1.2
HP, P=0.0042, FC=2.8
HP;HPR, P=1, FC=0.91
HPR, P=0.3, FC=2.1
HPX, P=0.061, FC=0.56
HRG, P=1, FC=1.7
IGF2, P=NA, FC=1.8
IGFALS, P=1, FC=0.78
IGFBP3, P=NA, FC=0.73
IGHA1, P=1, FC=1.1
IGHA1;IGHA2, P=0.33, FC=1.4
IGHA2, P=1, FC=1.4
IGHD, P=NA, FC=1.3
IGHG1, P=0.14, FC=0.26
SS 3 4 5 6 7 GS

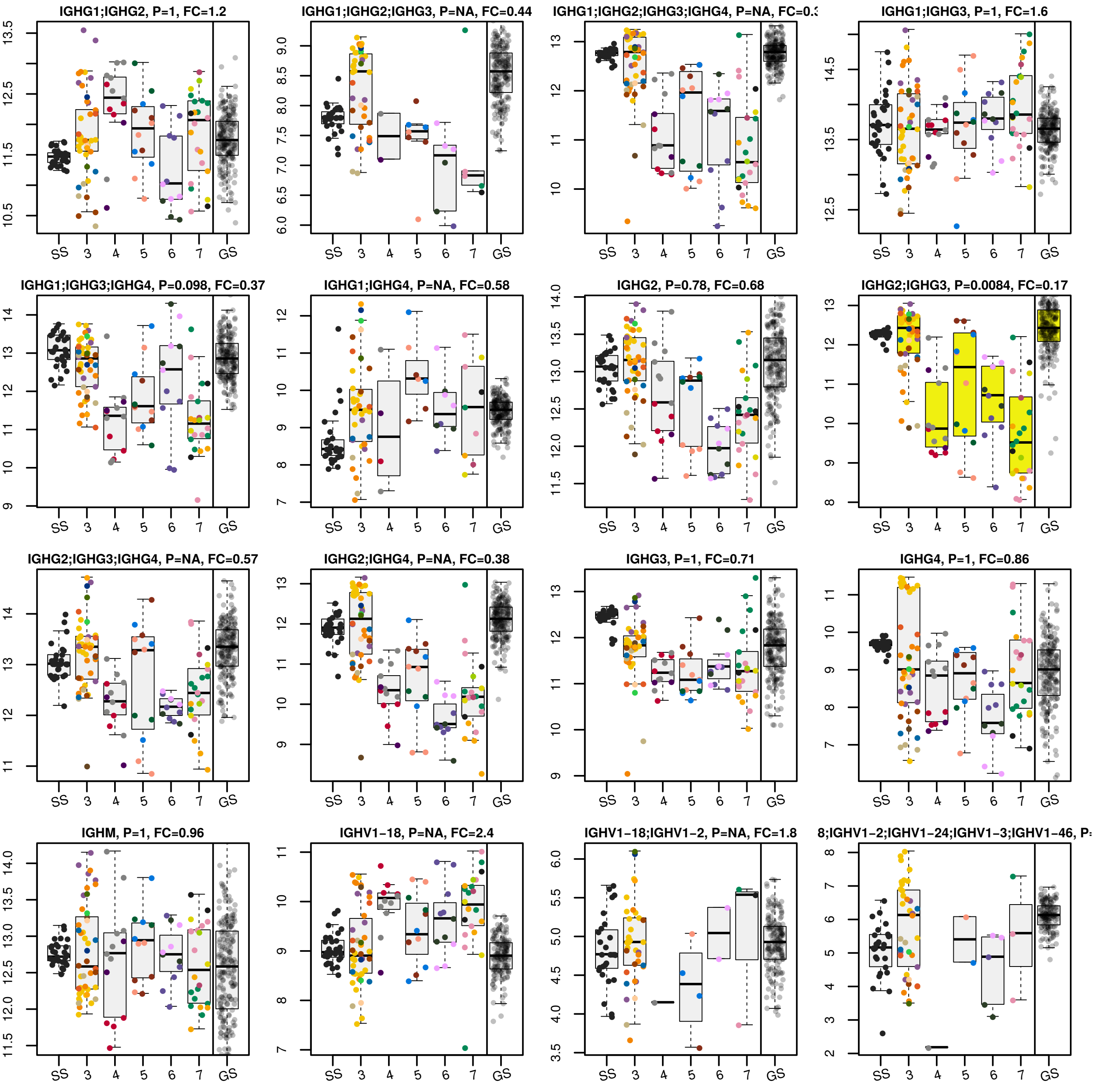
IGHG1;IGHG2, P=1, FC=1.2
IGHG1;IGHG2;IGHG3, P=NA, FC=0.44
IGHG1;IGHG2;IGHG3;IGHG4, P=NA, FC=0.3
IGHG1;IGHG3, P=1, FC=1.6
IGHG1;IGHG3;IGHG4, P=0.098, FC=0.37
IGHG1;IGHG4, P=NA, FC=0.58
IGHG2, P=0.78, FC=0.68
IGHG2;IGHG3, P=0.0084, FC=0.17
IGHG2;IGHG3;IGHG4, P=NA, FC=0.57
IGHG2;IGHG4, P=NA, FC=0.38
IGHG3, P=1, FC=0.71
IGHG4, P=1, FC=0.86
IGHM, P=1, FC=0.96
IGHV1-18, P=NA, FC=2.4
IGHV1-18;IGHV1-2, P=NA, FC=1.8
8;IGHV1-2;IGHV1-24;IGHV1-3;IGHV1-46, P=
SS 3 4 5 6 7 GS

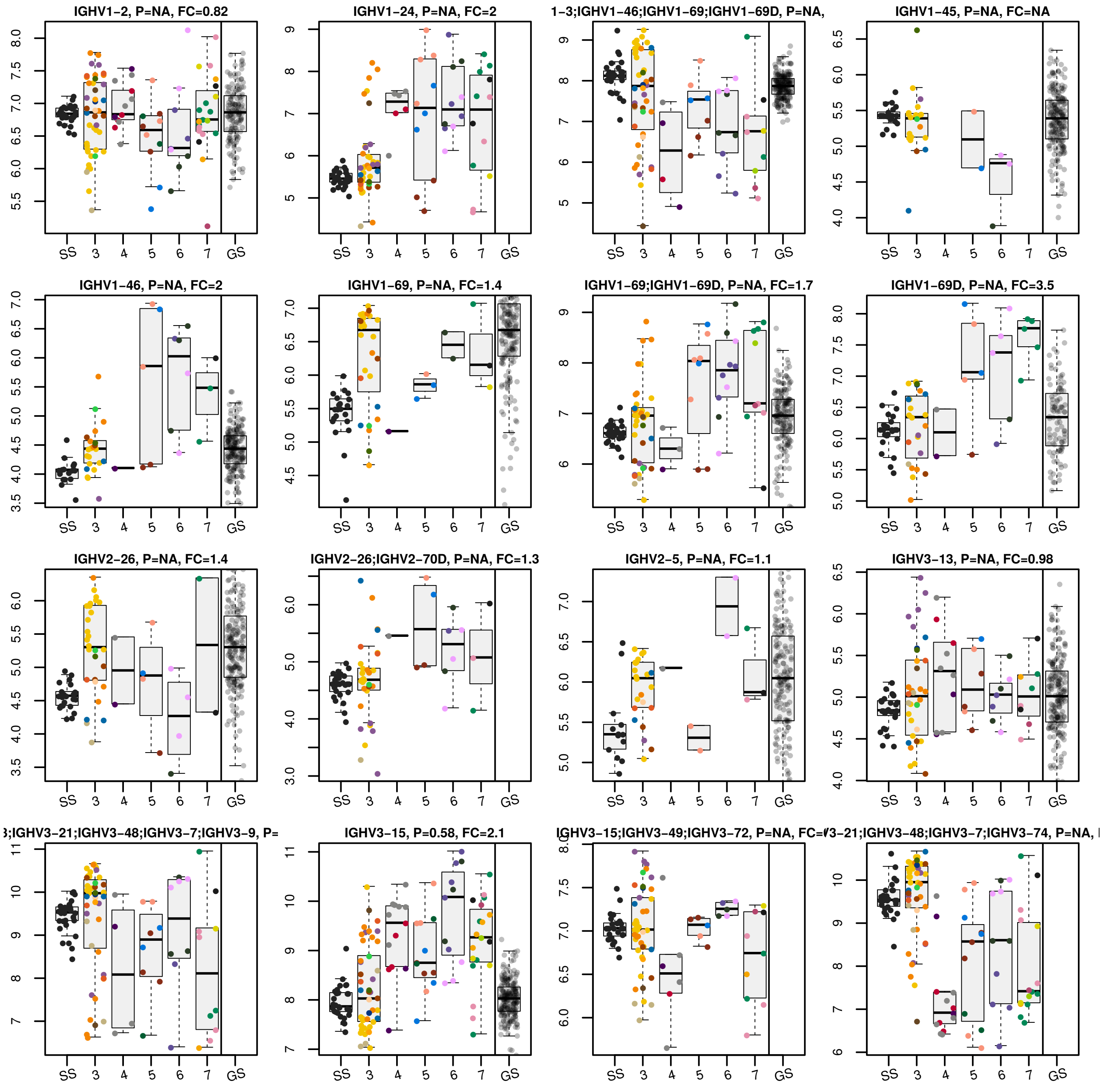
IGHV1−2, P=NA, FC=0.82
IGHV1−24, P=NA, FC=2
1−3;IGHV1−46;IGHV1−69;IGHV1−69D, P=NA,
IGHV1−45, P=NA, FC=NA
IGHV1−46, P=NA, FC=2
IGHV1−69, P=NA, FC=1.4
IGHV1−69;IGHV1−69D, P=NA, FC=1.7
IGHV1−69D, P=NA, FC=3.5
IGHV2−26, P=NA, FC=1.4
IGHV2−26;IGHV2−70D, P=NA, FC=1.3
IGHV2−5, P=NA, FC=1.1
IGHV3−13, P=NA, FC=0.98
3;IGHV3−21;IGHV3−48;IGHV3−7;IGHV3−9, P=
IGHV3−15, P=0.58, FC=2.1
IGHV3−15;IGHV3−49;IGHV3−72, P=NA, FC=
/3−21;IGHV3−48;IGHV3−7;IGHV3−74, P=NA, I
SS
3
4
5
6
7
GS

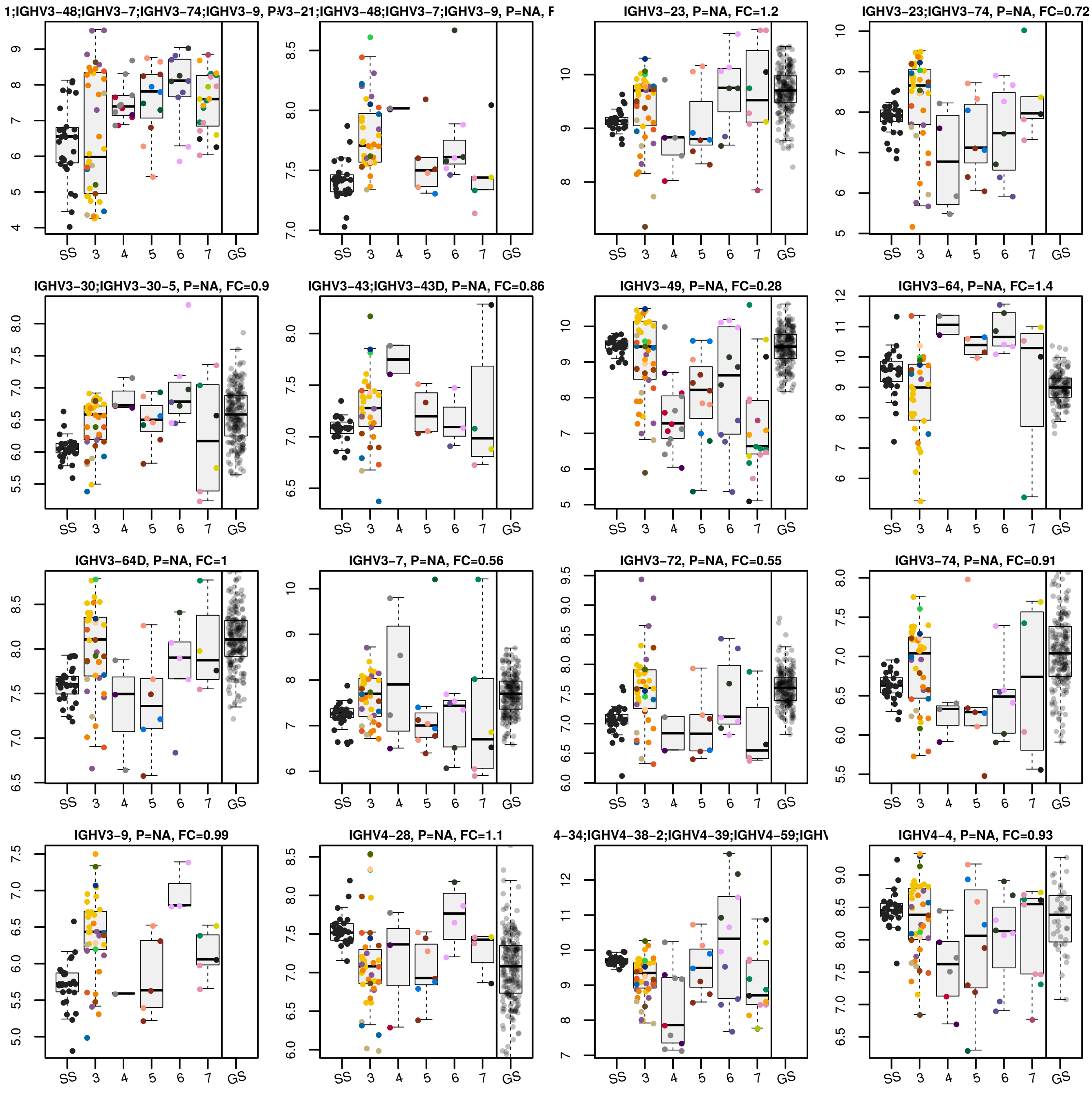
1;IGHV3-48;IGHV3-7;IGHV3-74;IGHV3-9, P
V3-21;IGHV3-48;IGHV3-7;IGHV3-9, P=NA, F
IGHV3-23, P=NA, FC=1.2
IGHV3-23;IGHV3-74, P=NA, FC=0.72
IGHV3-30;IGHV3-30-5, P=NA, FC=0.9
IGHV3-43;IGHV3-43D, P=NA, FC=0.86
IGHV3-49, P=NA, FC=0.28
IGHV3-64, P=NA, FC=1.4
IGHV3-64D, P=NA, FC=1
IGHV3-7, P=NA, FC=0.56
IGHV3-72, P=NA, FC=0.55
IGHV3-74, P=NA, FC=0.91
IGHV3-9, P=NA, FC=0.99
IGHV4-28, P=NA, FC=1.1
4-34;IGHV4-38-2;IGHV4-39;IGHV4-59;IGHV
IGHV4-4, P=NA, FC=0.93
SS
3
4
5
6
7
GS

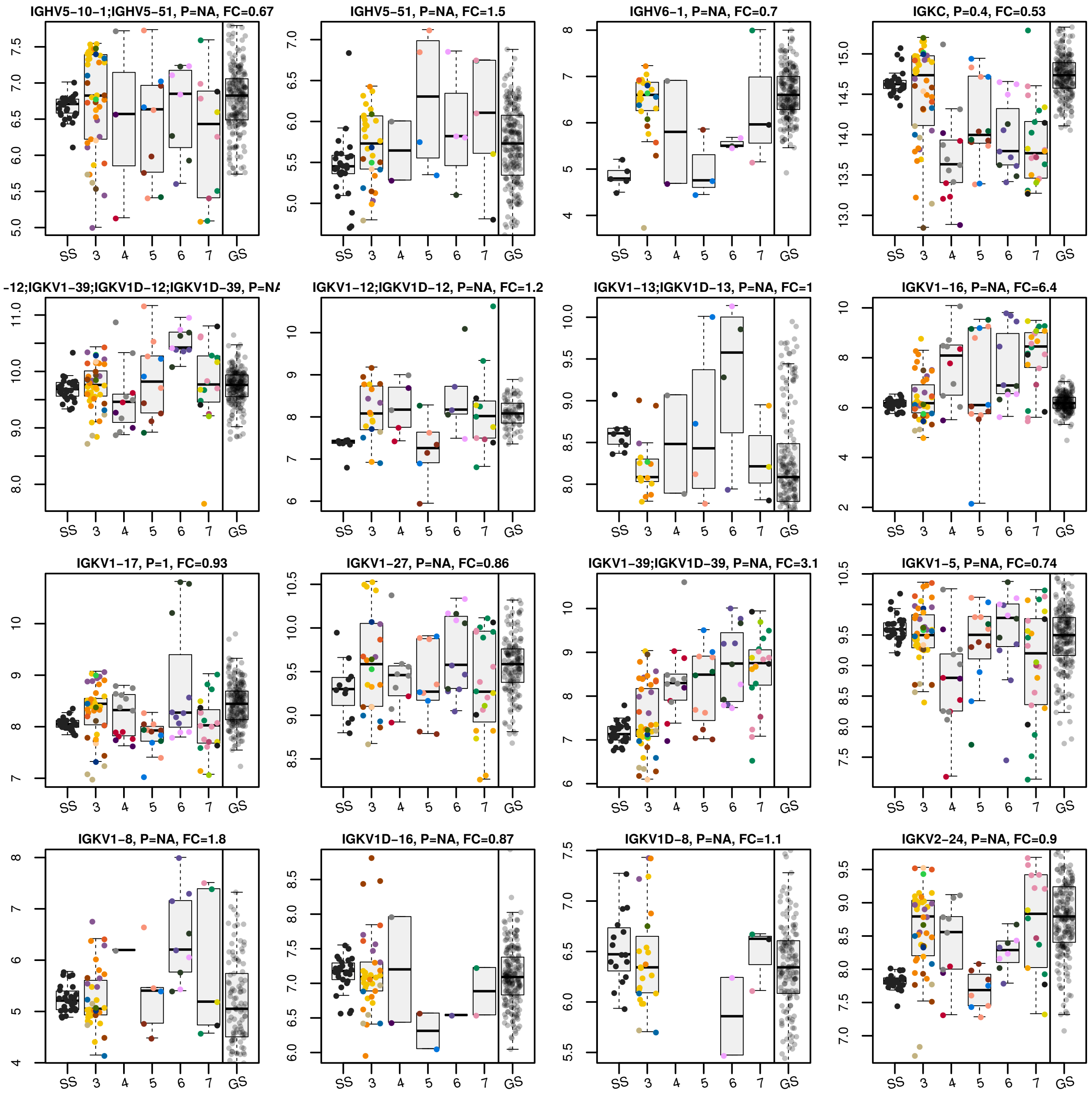
IGHV5−10−1;IGHV5−51, P=NA, FC=0.67
IGHV5−51, P=NA, FC=1.5
IGHV6−1, P=NA, FC=0.7
IGKC, P=0.4, FC=0.53
−12;IGKV1−39;IGKV1D−12;IGKV1D−39, P=NA
IGKV1−12;IGKV1D−12, P=NA, FC=1.2
IGKV1−13;IGKV1D−13, P=NA, FC=1
IGKV1−16, P=NA, FC=6.4
IGKV1−17, P=1, FC=0.93
IGKV1−27, P=NA, FC=0.86
IGKV1−39;IGKV1D−39, P=NA, FC=3.1
IGKV1−5, P=NA, FC=0.74
IGKV1−8, P=NA, FC=1.8
IGKV1D−16, P=NA, FC=0.87
IGKV1D−8, P=NA, FC=1.1
IGKV2−24, P=NA, FC=0.9
SS
3
4
5
6
7
GS

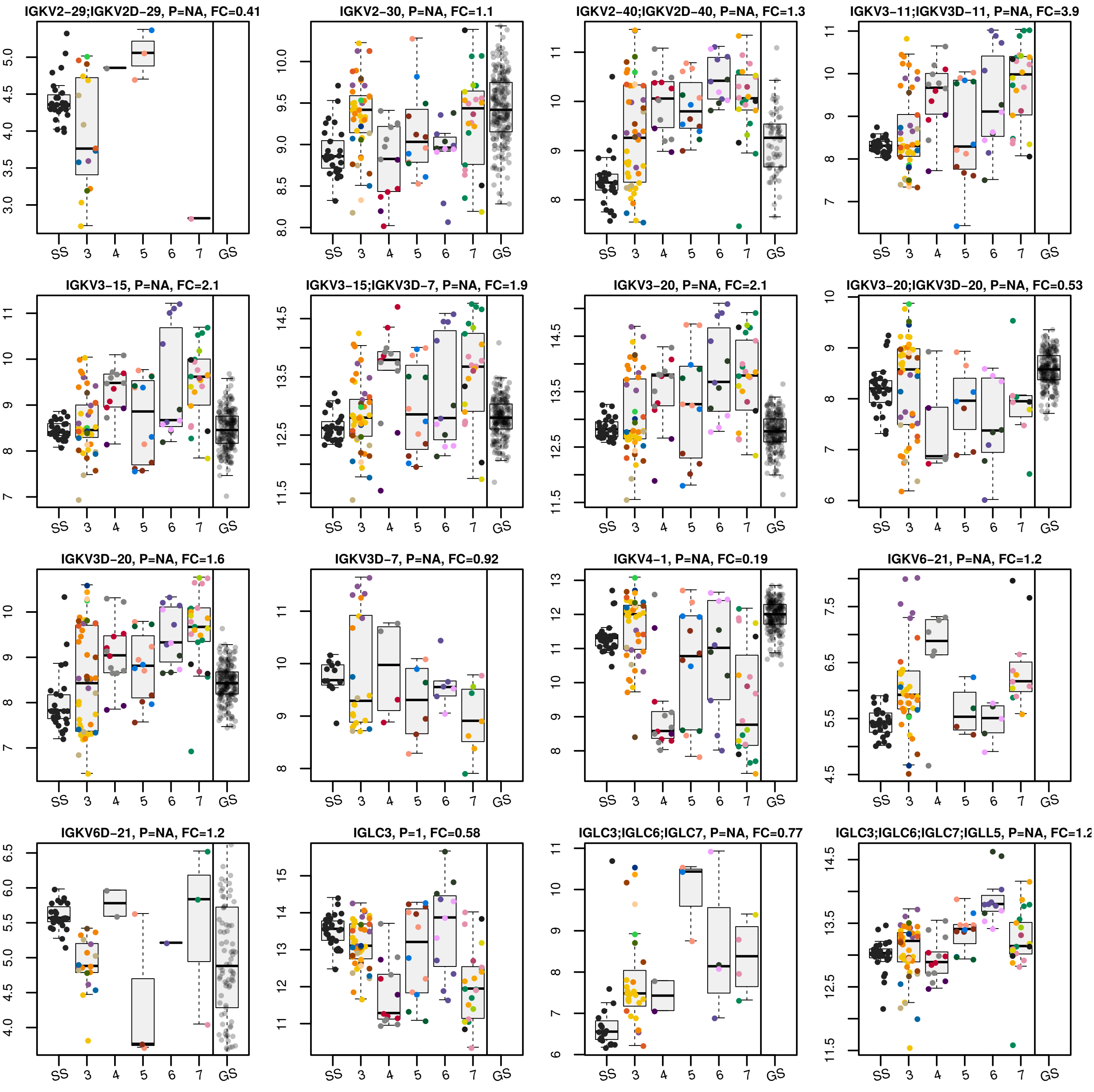
IGKV2−29;IGKV2D−29, P=NA, FC=0.41
IGKV2−30, P=NA, FC=1.1
IGKV2−40;IGKV2D−40, P=NA, FC=1.3
IGKV3−11;IGKV3D−11, P=NA, FC=3.9
IGKV3−15, P=NA, FC=2.1
IGKV3−15;IGKV3D−7, P=NA, FC=1.9
IGKV3−20, P=NA, FC=2.1
IGKV3−20;IGKV3D−20, P=NA, FC=0.53
IGKV3D−20, P=NA, FC=1.6
IGKV3D−7, P=NA, FC=0.92
IGKV4−1, P=NA, FC=0.19
IGKV6−21, P=NA, FC=1.2
IGKV6D−21, P=NA, FC=1.2
IGLC3, P=1, FC=0.58
IGLC3;IGLC6;IGLC7, P=NA, FC=0.77
IGLC3;IGLC6;IGLC7;IGLL5, P=NA, FC=1.2
SS
3
4
5
6
7
GS

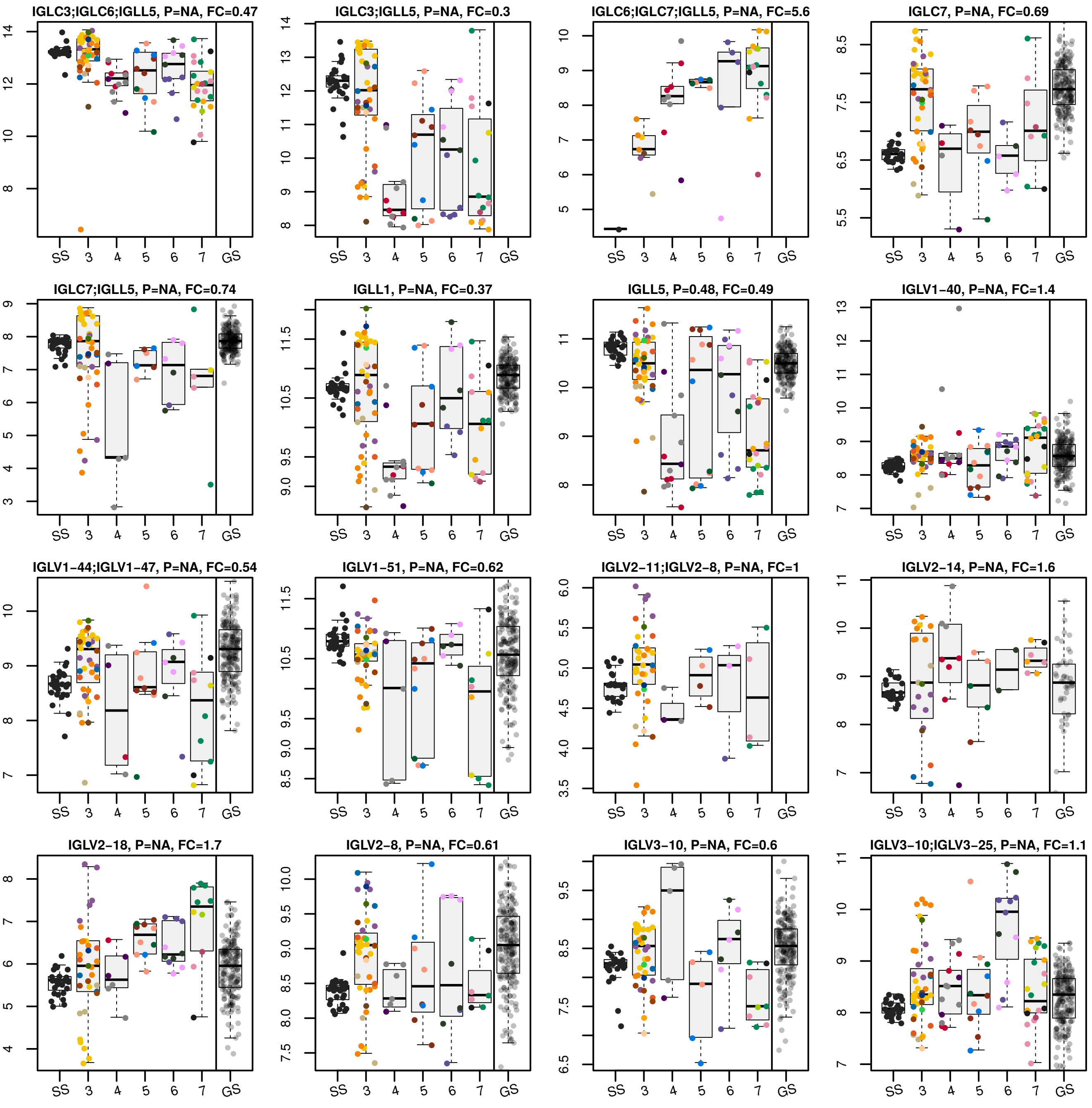
IGLC3;IGLC6;IGLL5, P=NA, FC=0.47
IGLC3;IGLL5, P=NA, FC=0.3
IGLC6;IGLC7;IGLL5, P=NA, FC=5.6
IGLC7, P=NA, FC=0.69
IGLC7;IGLL5, P=NA, FC=0.74
IGLL1, P=NA, FC=0.37
IGLL5, P=0.48, FC=0.49
IGLV1-40, P=NA, FC=1.4
IGLV1-44;IGLV1-47, P=NA, FC=0.54
IGLV1-51, P=NA, FC=0.62
IGLV2-11;IGLV2-8, P=NA, FC=1
IGLV2-14, P=NA, FC=1.6
IGLV2-18, P=NA, FC=1.7
IGLV2-8, P=NA, FC=0.61
IGLV3-10, P=NA, FC=0.6
IGLV3-10;IGLV3-25, P=NA, FC=1.1
SS
3
4
5
6
7
GS

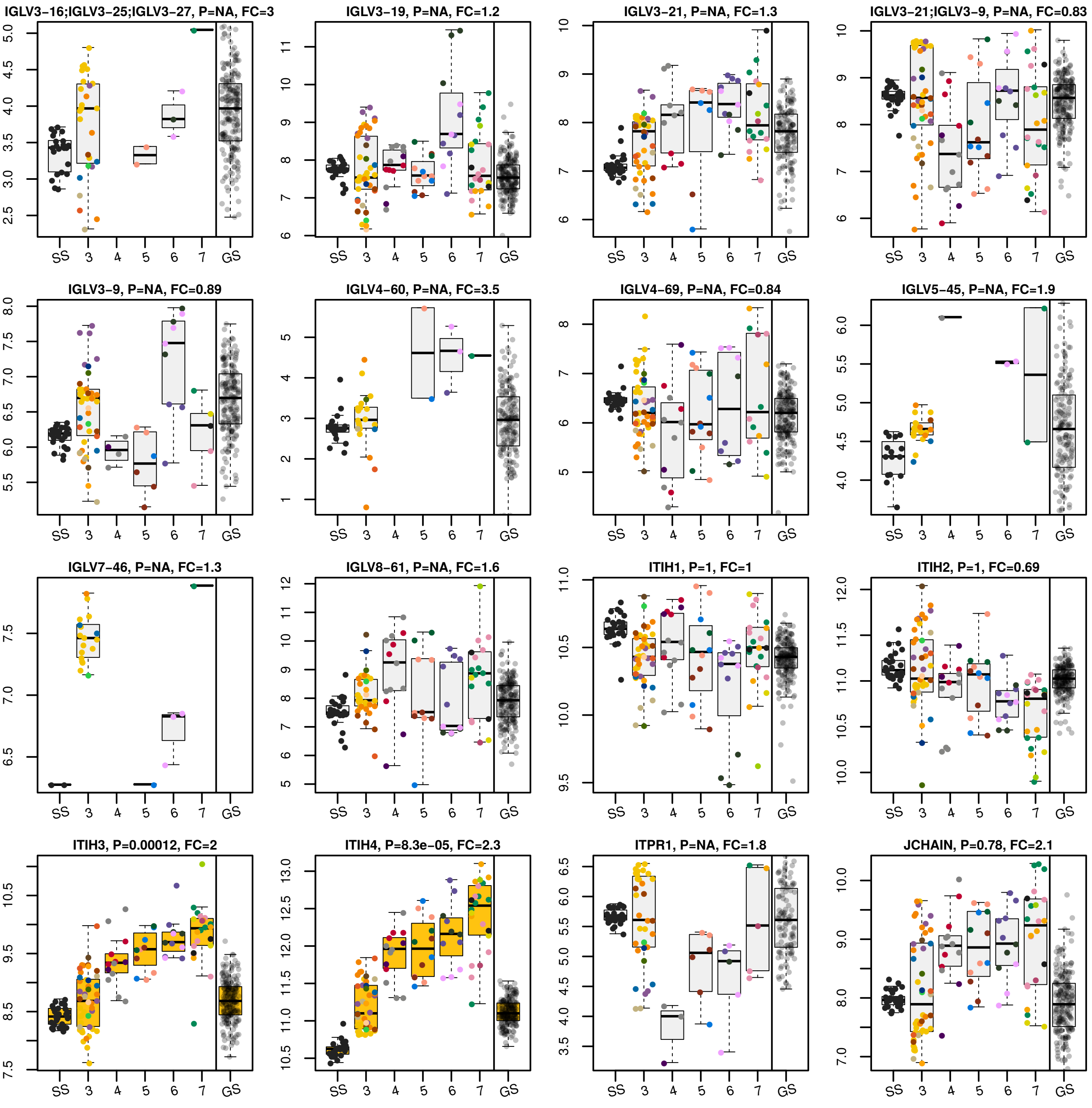
IGLV3−16;IGLV3−25;IGLV3−27, P=NA, FC=3
IGLV3−19, P=NA, FC=1.2
IGLV3−21, P=NA, FC=1.3
IGLV3−21;IGLV3−9, P=NA, FC=0.83
IGLV3−9, P=NA, FC=0.89
IGLV4−60, P=NA, FC=3.5
IGLV4−69, P=NA, FC=0.84
IGLV5−45, P=NA, FC=1.9
IGLV7−46, P=NA, FC=1.3
IGLV8−61, P=NA, FC=1.6
ITIH1, P=1, FC=1
ITIH2, P=1, FC=0.69
ITIH3, P=0.00012, FC=2
ITIH4, P=8.3e−05, FC=2.3
ITPR1, P=NA, FC=1.8
JCHAIN, P=0.78, FC=2.1
SS 3 4 5 6 7 GS

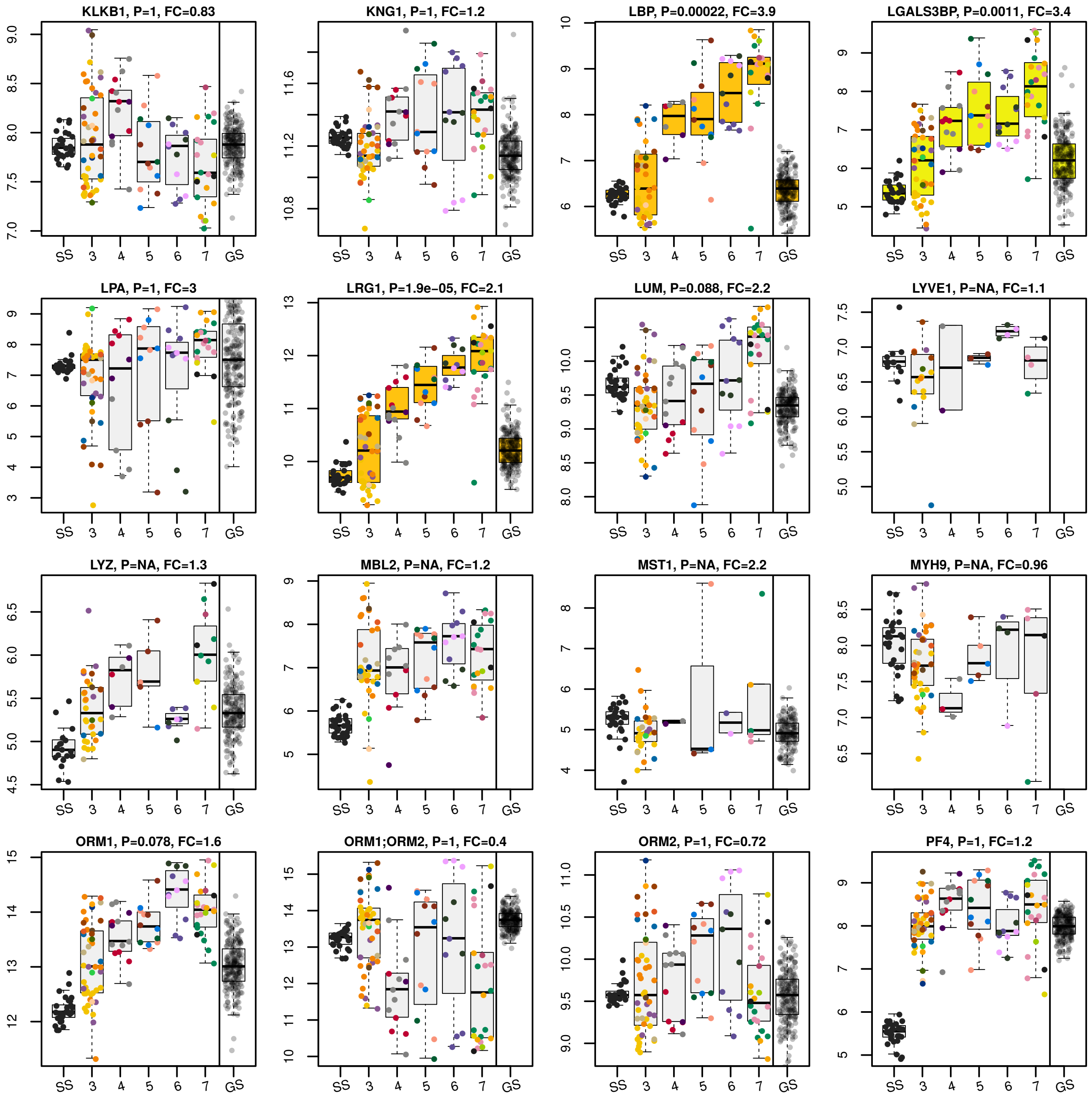
KLKB1, P=1, FC=0.83
KNG1, P=1, FC=1.2
LBP, P=0.00022, FC=3.9
LGALS3BP, P=0.0011, FC=3.4
LPA, P=1, FC=3
LRG1, P=1.9e−05, FC=2.1
LUM, P=0.088, FC=2.2
LYVE1, P=NA, FC=1.1
LYZ, P=NA, FC=1.3
MBL2, P=NA, FC=1.2
MST1, P=NA, FC=2.2
MYH9, P=NA, FC=0.96
ORM1, P=0.078, FC=1.6
ORM1;ORM2, P=1, FC=0.4
ORM2, P=1, FC=0.72
PF4, P=1, FC=1.2
SS 3 4 5 6 7 GS

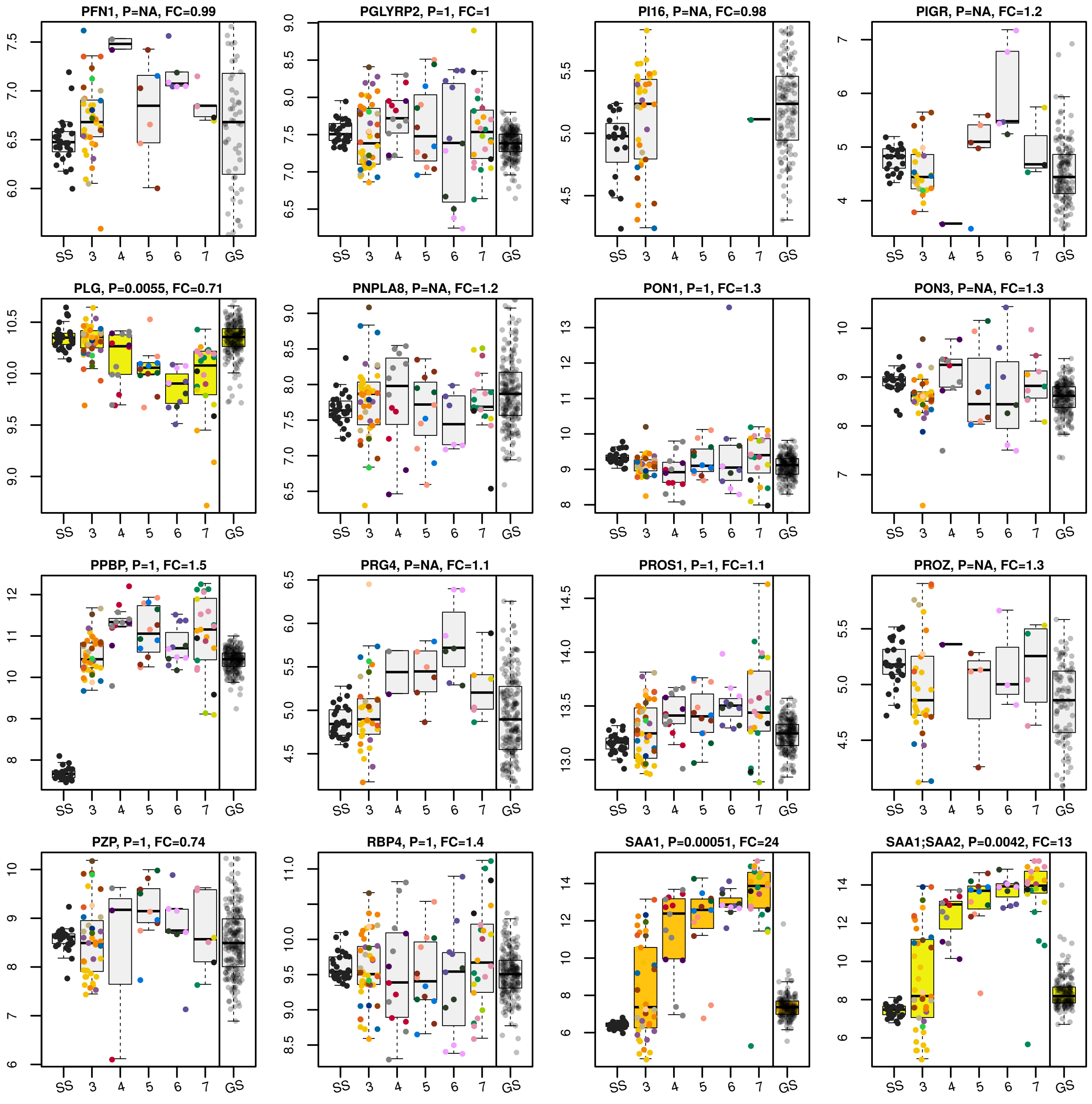
PFN1, P=NA, FC=0.99
PGLYRP2, P=1, FC=1
PI16, P=NA, FC=0.98
PIGR, P=NA, FC=1.2
PLG, P=0.0055, FC=0.71
PNPLA8, P=NA, FC=1.2
PON1, P=1, FC=1.3
PON3, P=NA, FC=1.3
PPBP, P=1, FC=1.5
PRG4, P=NA, FC=1.1
PROS1, P=1, FC=1.1
PROZ, P=NA, FC=1.3
PZP, P=1, FC=0.74
RBP4, P=1, FC=1.4
SAA1, P=0.00051, FC=24
SAA1;SAA2, P=0.0042, FC=13
SS 3 4 5 6 7 GS

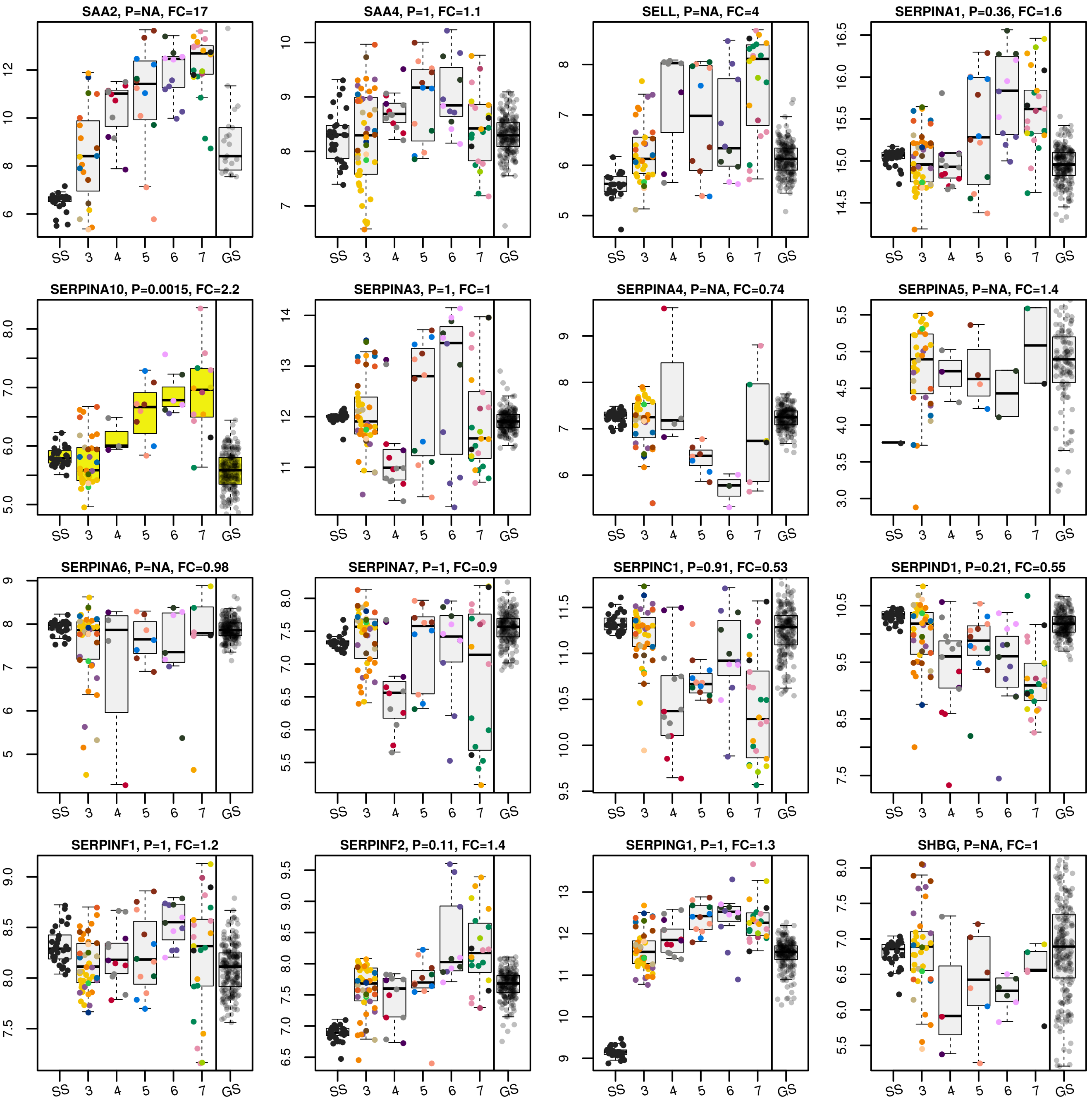
SAA2, P=NA, FC=17
SAA4, P=1, FC=1.1
SELL, P=NA, FC=4
SERPINA1, P=0.36, FC=1.6
SERPINA10, P=0.0015, FC=2.2
SERPINA3, P=1, FC=1
SERPINA4, P=NA, FC=0.74
SERPINA5, P=NA, FC=1.4
SERPINA6, P=NA, FC=0.98
SERPINA7, P=1, FC=0.9
SERPINC1, P=0.91, FC=0.53
SERPIND1, P=0.21, FC=0.55
SERPINF1, P=1, FC=1.2
SERPINF2, P=0.11, FC=1.4
SERPING1, P=1, FC=1.3
SHBG, P=NA, FC=1
SS
3
4
5
6
7
GS

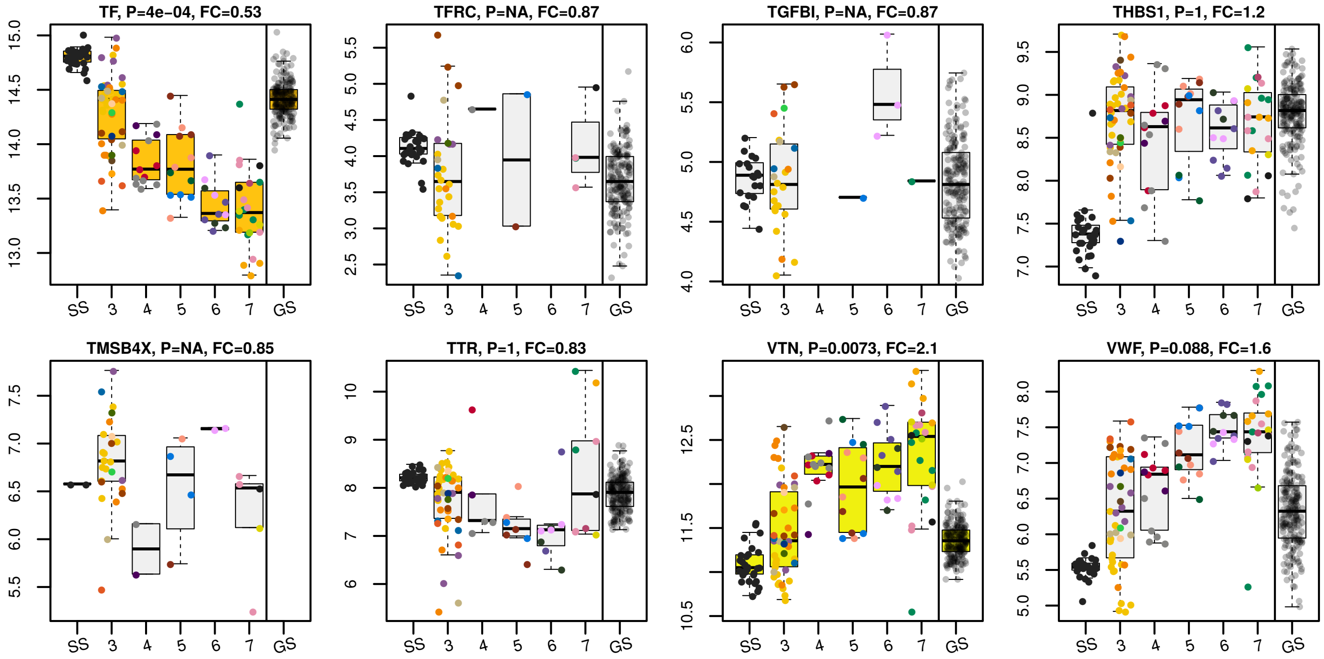
TF, P=4e−04, FC=0.53
TFRC, P=NA, FC=0.87
TGFBI, P=NA, FC=0.87
THBS1, P=1, FC=1.2
TMSB4X, P=NA, FC=0.85
TTR, P=1, FC=0.83
VTN, P=0.0073, FC=2.1
VWF, P=0.088, FC=1.6
SS
3
4
5
6
7
GS

Figure S8 (following 15 pages). **Age-related protein changes in the general population (GS cohort)**.

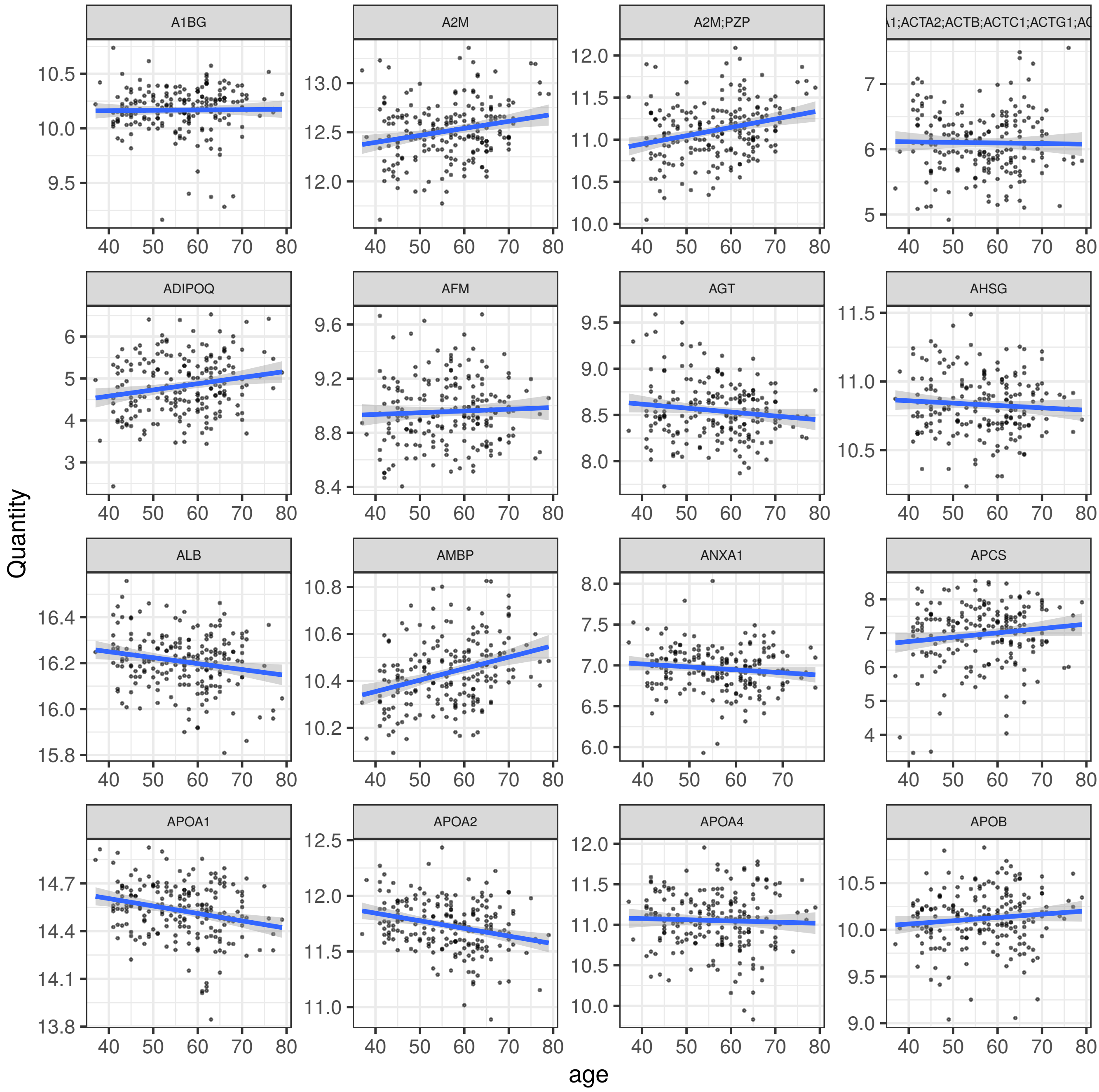
A1BG
A2M
A2M;PZP
1;ACTA2;ACTB;ACTC1;ACTG1;AC
ADIPOQ
AFM
AGT
AHSG
ALB
AMBP
ANXA1
APCS
APOA1
APOA2
APOA4
APOB
Quantity
age

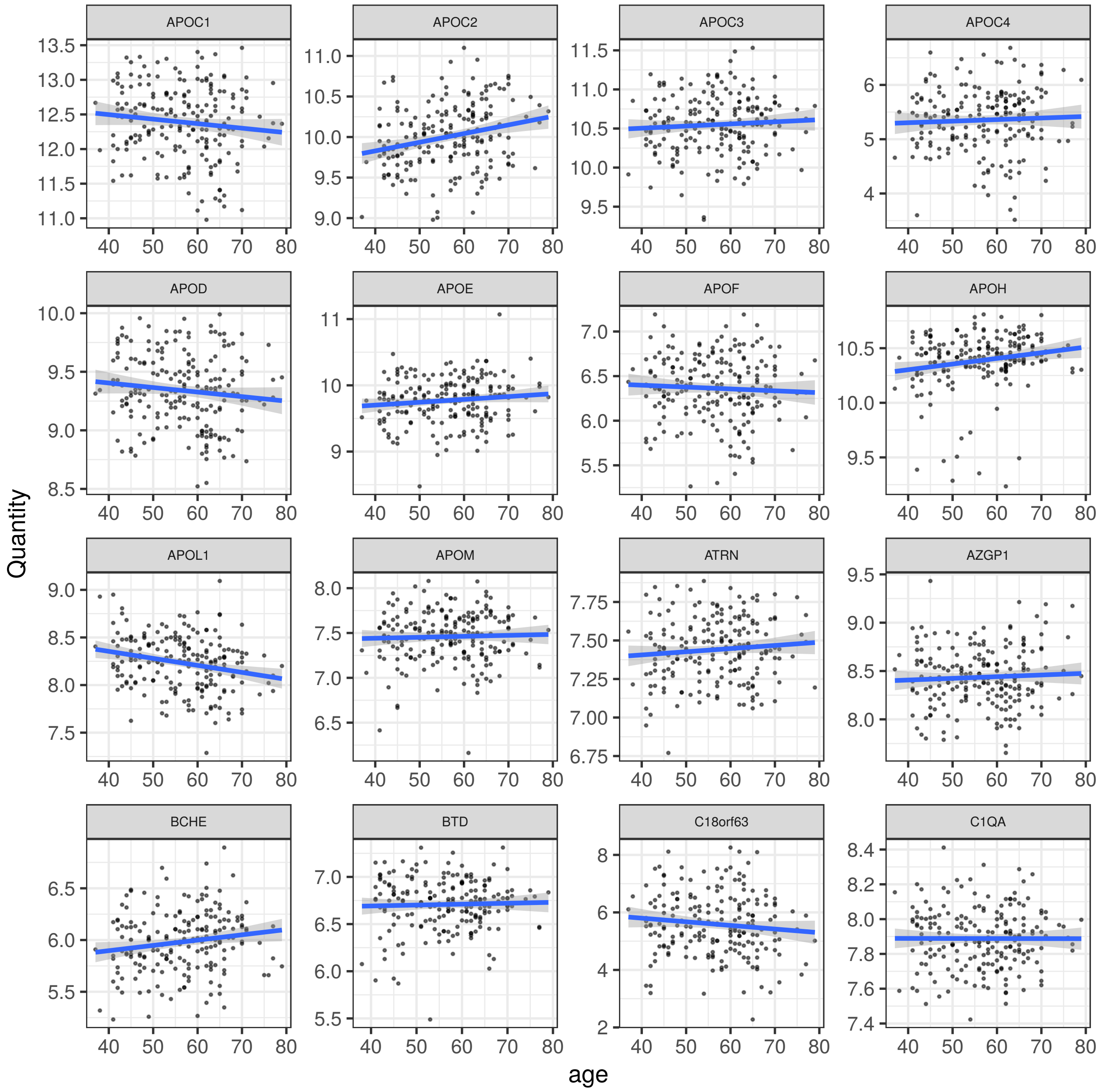
APOC1
APOC2
APOC3
APOC4
APOD
APOE
APOF
APOH
APOL1
APOM
ATRN
AZGP1
BCHE
BTD
C18orf63
C1QA
Quantity
age

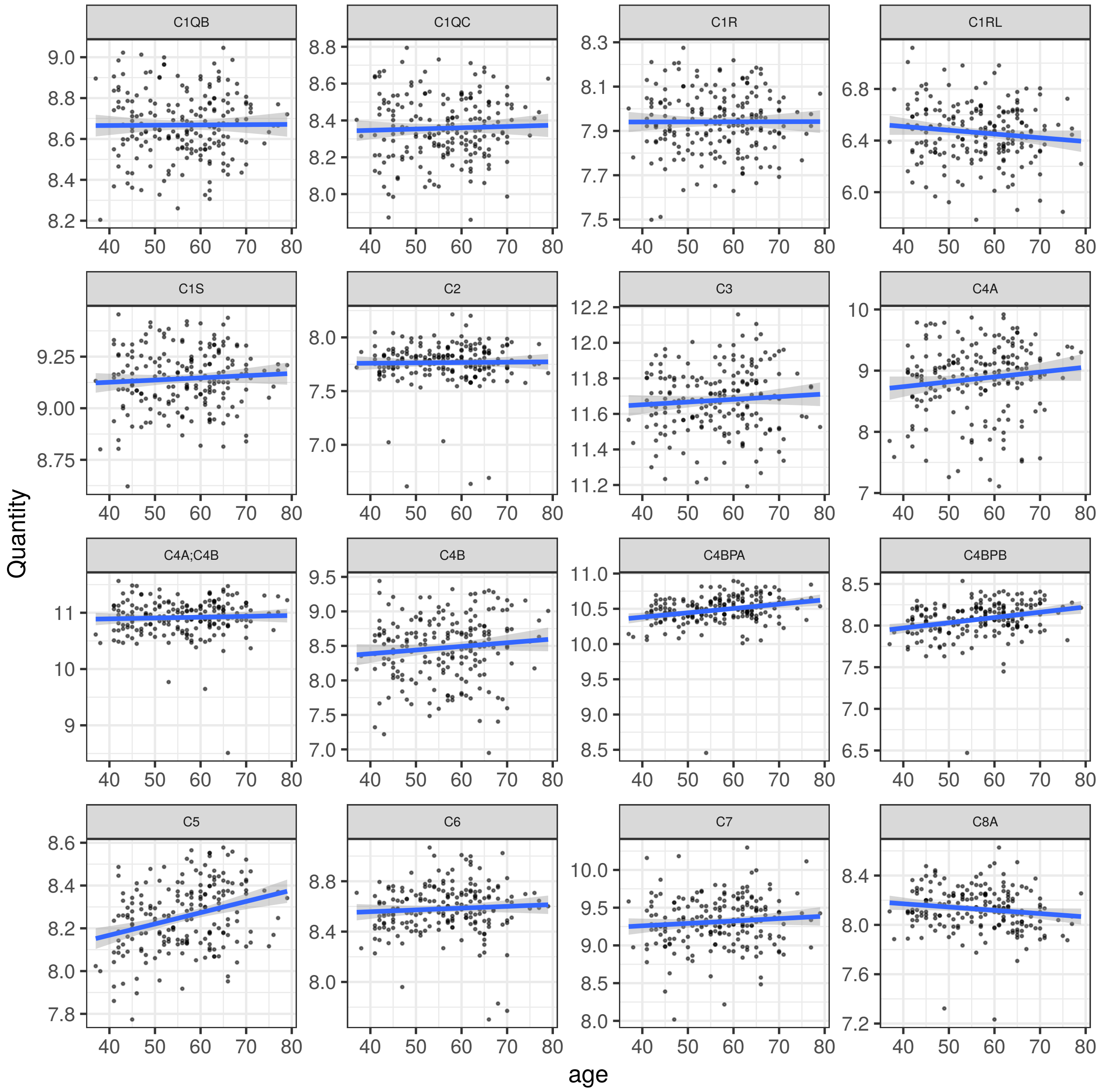

C1QB
C1QC
C1R
C1RL
C1S
C2
C3
C4A
C4A;C4B
C4B
C4BPA
C4BPB
C5
C6
C7
C8A
Quantity
age

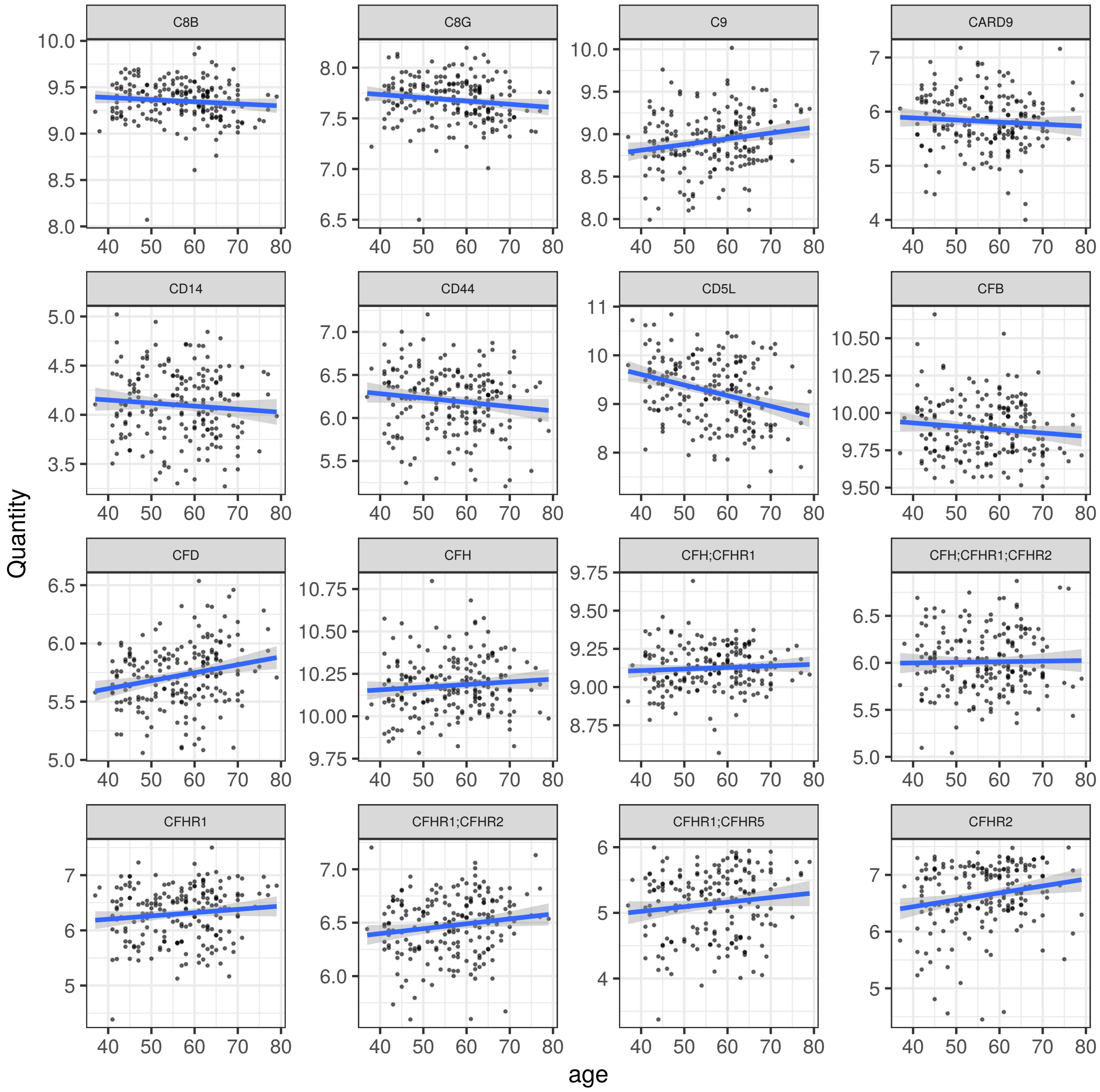
C8B
C8G
C9
CARD9
CD14
CD44
CD5L
CFB
CFD
CFH
CFH;CFHR1
CFH;CFHR1;CFHR2
CFHR1
CFHR1;CFHR2
CFHR1;CFHR5
CFHR2
Quantity
age

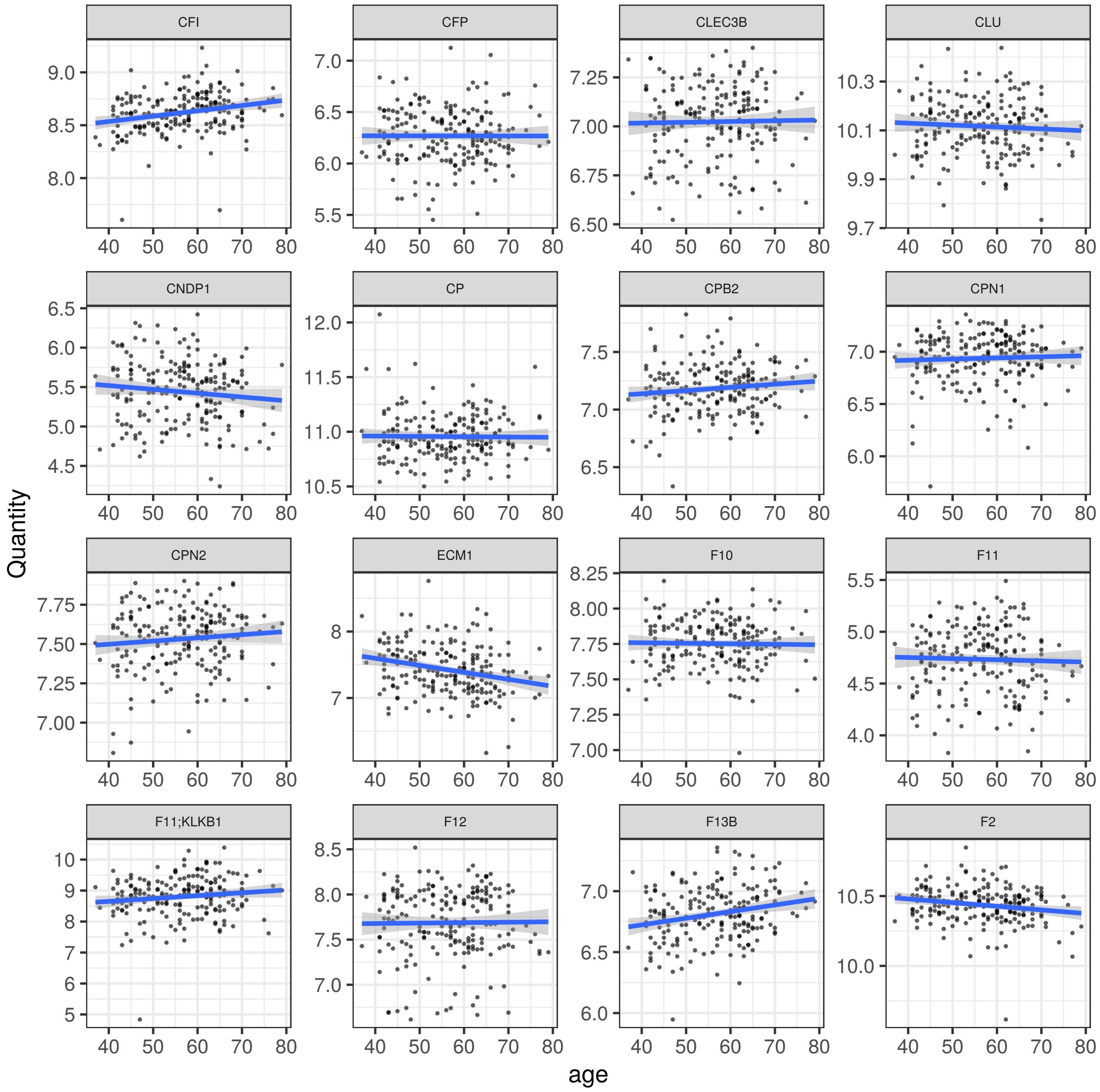
CFI
CFP
CLEC3B
CLU
CNDP1
CP
CPB2
CPN1
CPN2
ECM1
F10
F11
F11;KLKB1
F12
F13B
F2
Quantity
age

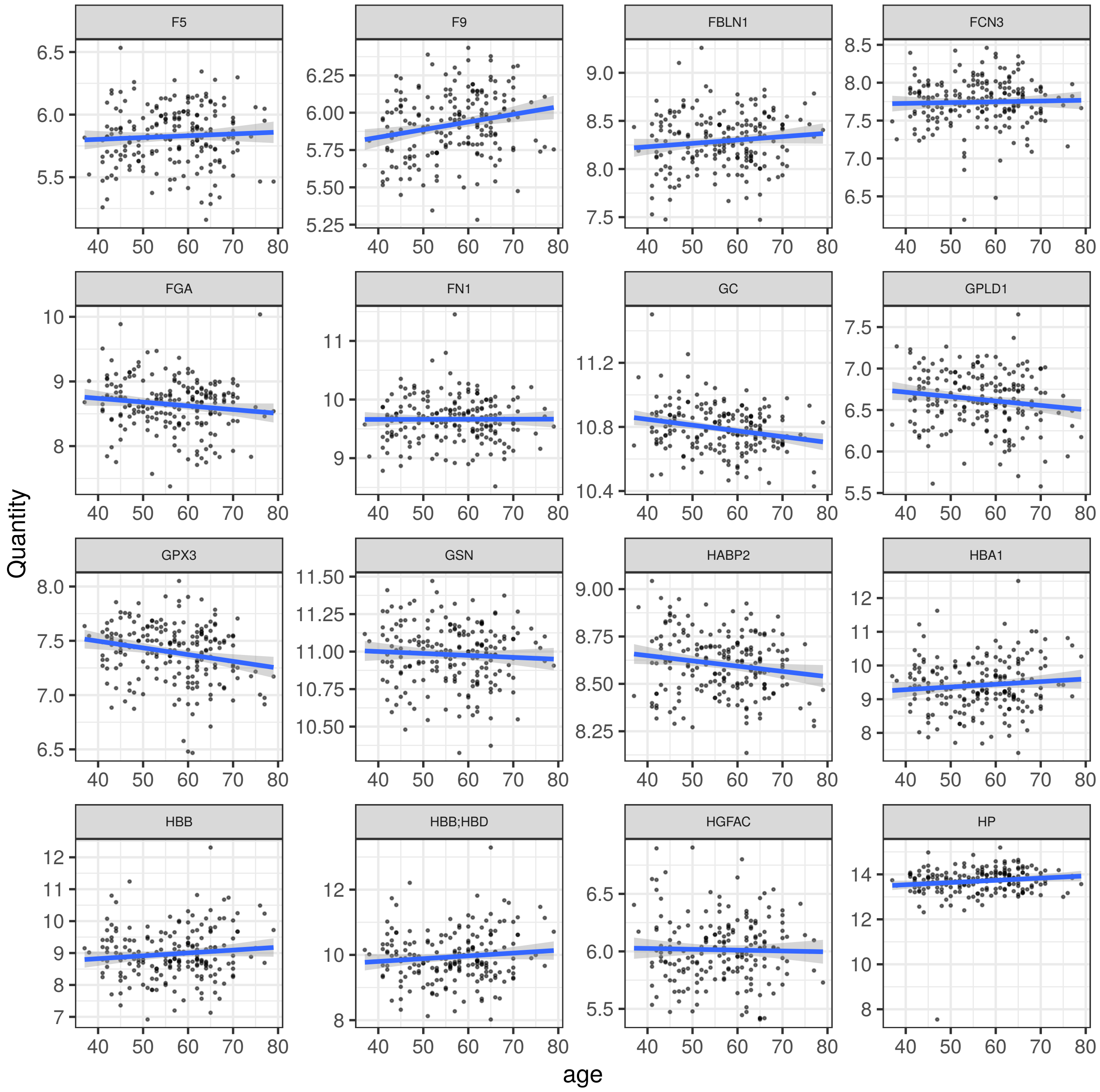
F5
F9
FBLN1
FCN3
FGA
FN1
GC
GPLD1
GPX3
GSN
HABP2
HBA1
HBB
HBB;HBD
HGFAC
HP
Quantity
age

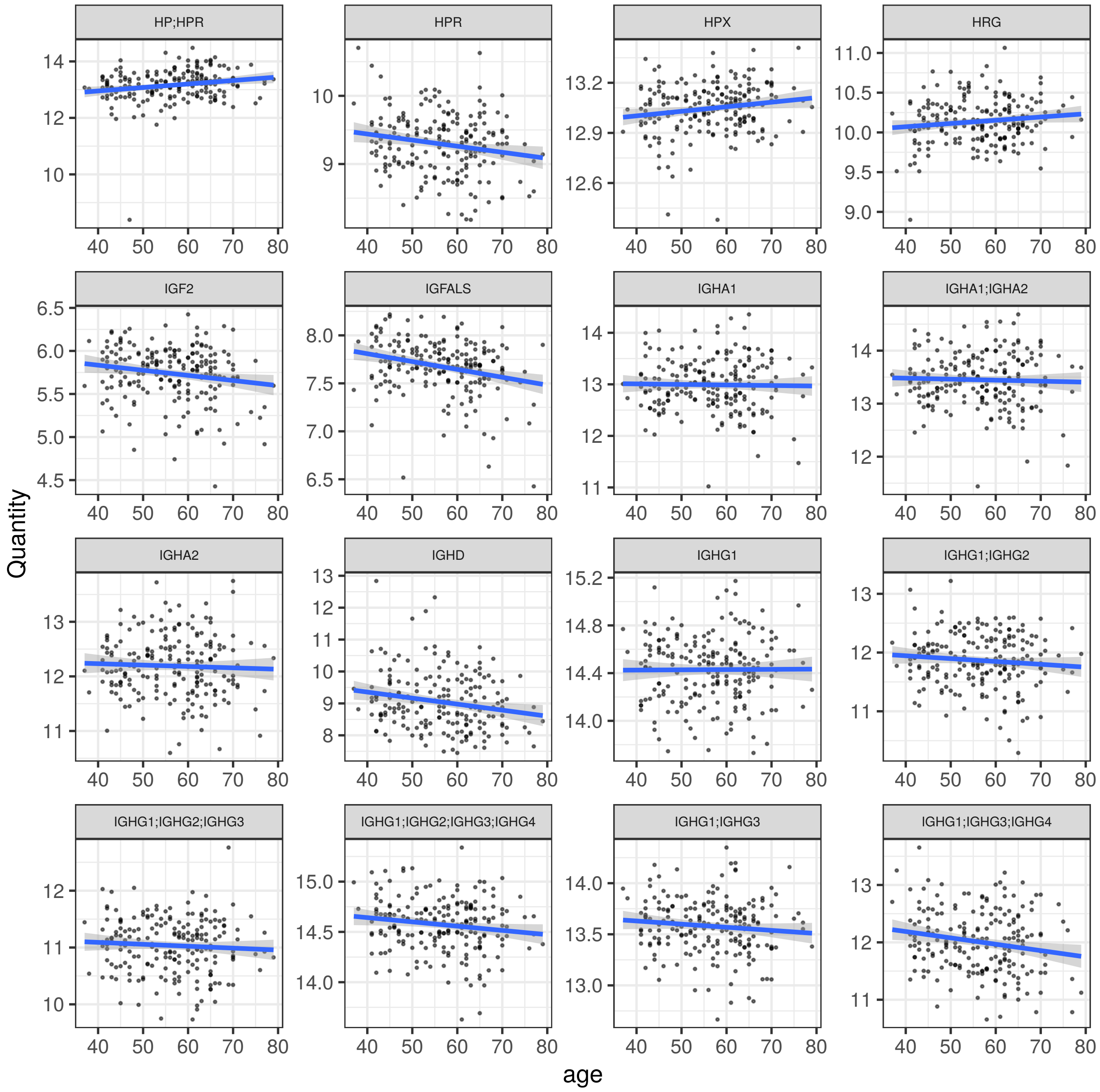
HP;HPR
HPR
HPX
HRG
IGF2
IGFALS
IGHA1
IGHA1;IGHA2
IGHA2
IGHD
IGHG1
IGHG1;IGHG2
IGHG1;IGHG2;IGHG3
IGHG1;IGHG2;IGHG3;IGHG4
IGHG1;IGHG3
IGHG1;IGHG3;IGHG4
Quantity
age

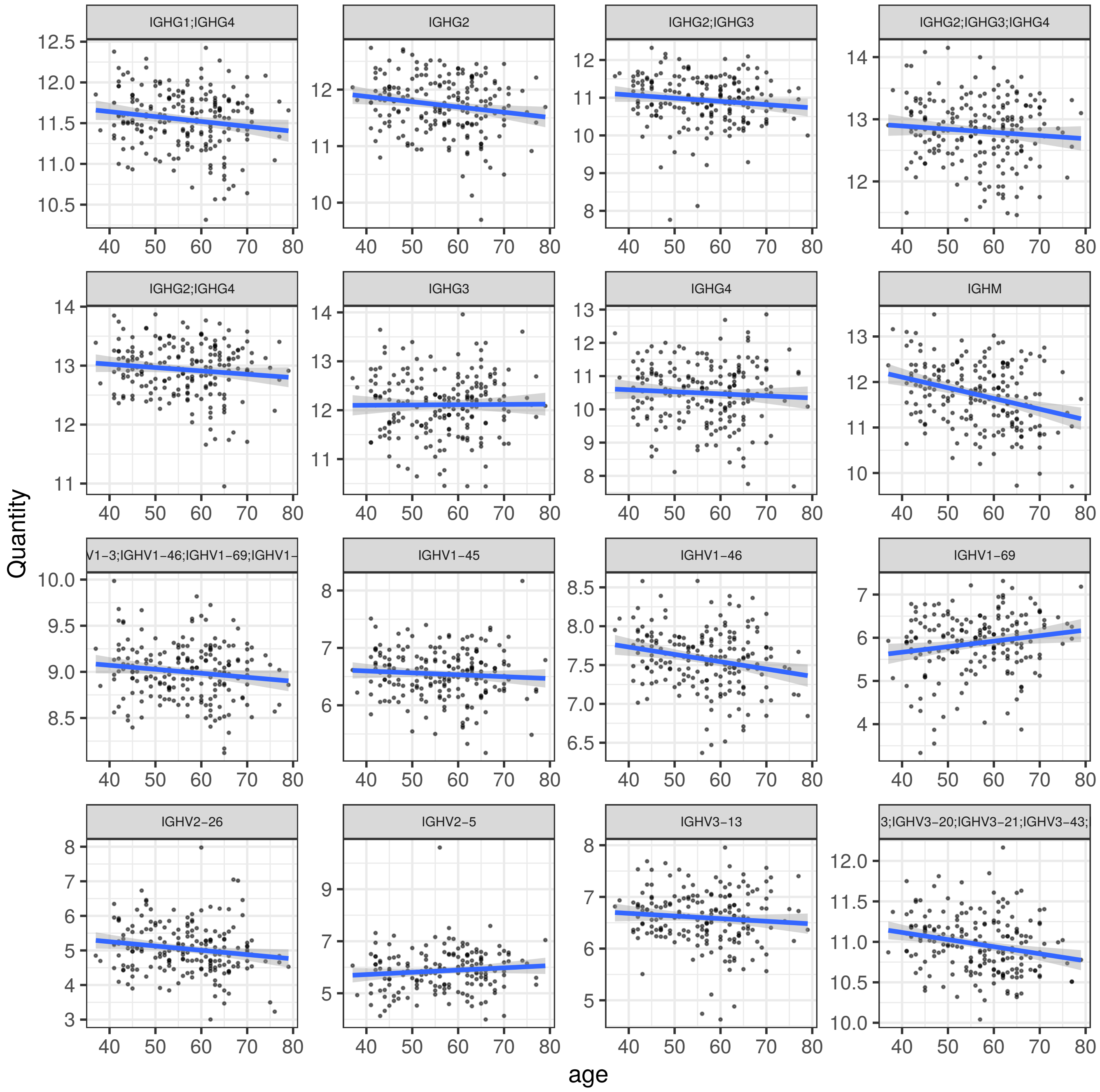
IGHG1;IGHG4
IGHG2
IGHG2;IGHG3
IGHG2;IGHG3;IGHG4
IGHG2;IGHG4
IGHG3
IGHG4
IGHM
V1-3;IGHV1-46;IGHV1-69;IGHV1-
IGHV1-45
IGHV1-46
IGHV1-69
IGHV2-26
IGHV2-5
IGHV3-13
3;IGHV3-20;IGHV3-21;IGHV3-43;
Quantity
age

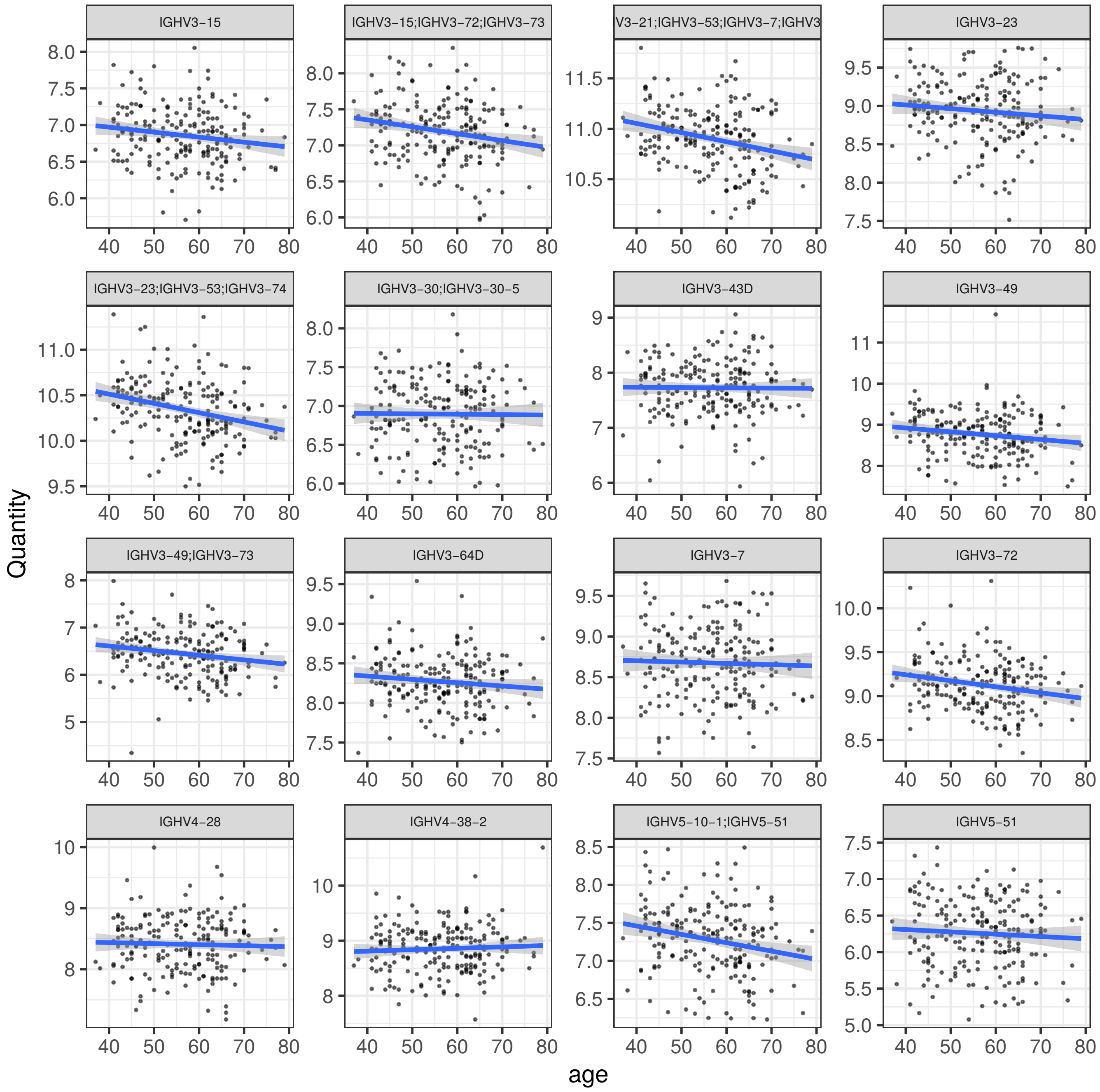

IGHV3-15
IGHV3-15;IGHV3-72;IGHV3-73
V3-21;IGHV3-53;IGHV3-7;IGHV3
IGHV3-23
IGHV3-23;IGHV3-53;IGHV3-74
IGHV3-30;IGHV3-30-5
IGHV3-43D
IGHV3-49
IGHV3-49;IGHV3-73
IGHV3-64D
IGHV3-7
IGHV3-72
IGHV4-28
IGHV4-38-2
IGHV5-10-1;IGHV5-51
IGHV5-51
Quantity
age

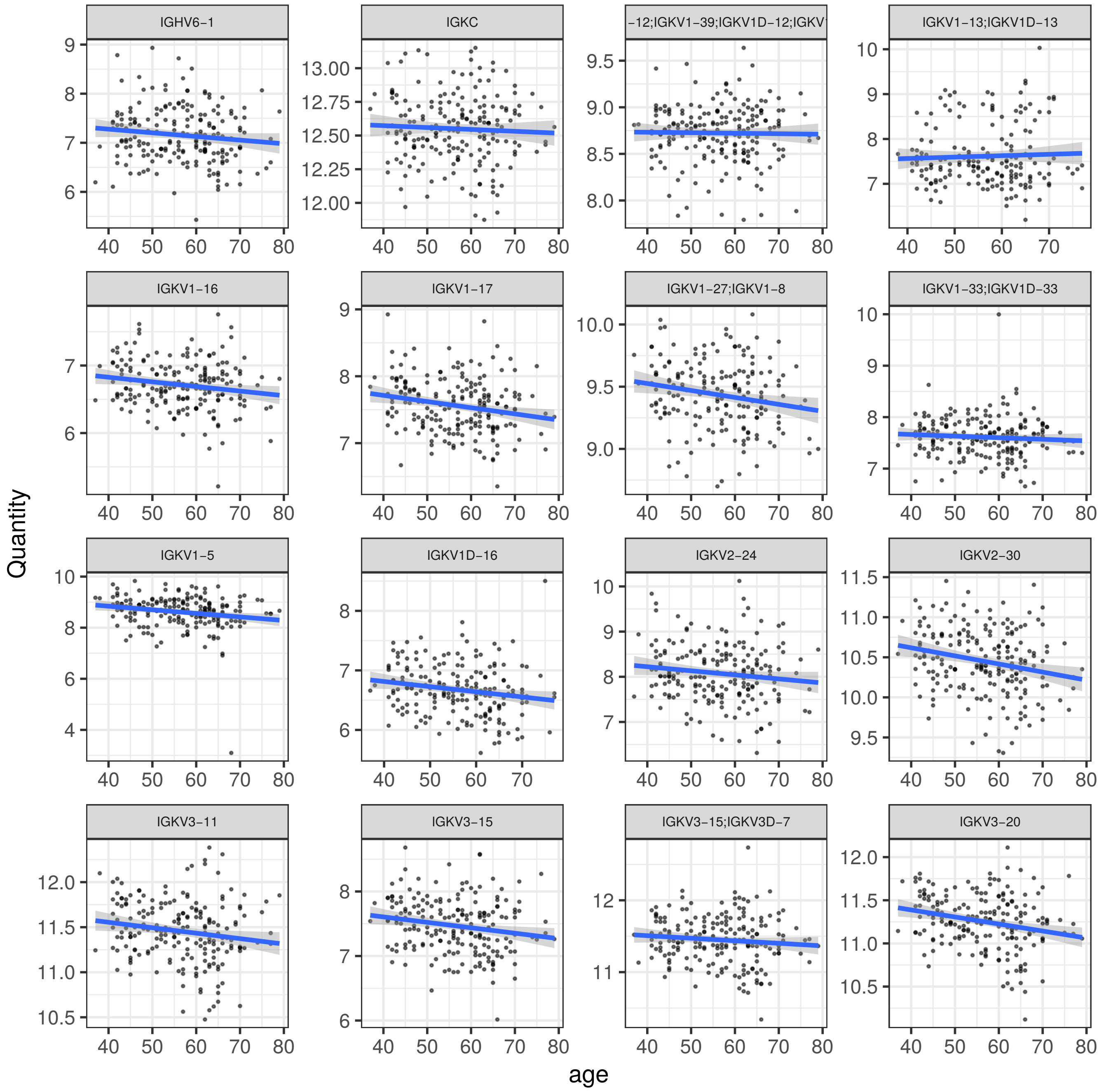

IGHV6-1
IGKC
-12;IGKV1-39;IGKV1D-12;IGKV
IGKV1-13;IGKV1D-13
IGKV1-16
IGKV1-17
IGKV1-27;IGKV1-8
IGKV1-33;IGKV1D-33
IGKV1-5
IGKV1D-16
IGKV2-24
IGKV2-30
IGKV3-11
IGKV3-15
IGKV3-15;IGKV3D-7
IGKV3-20
Quantity
age

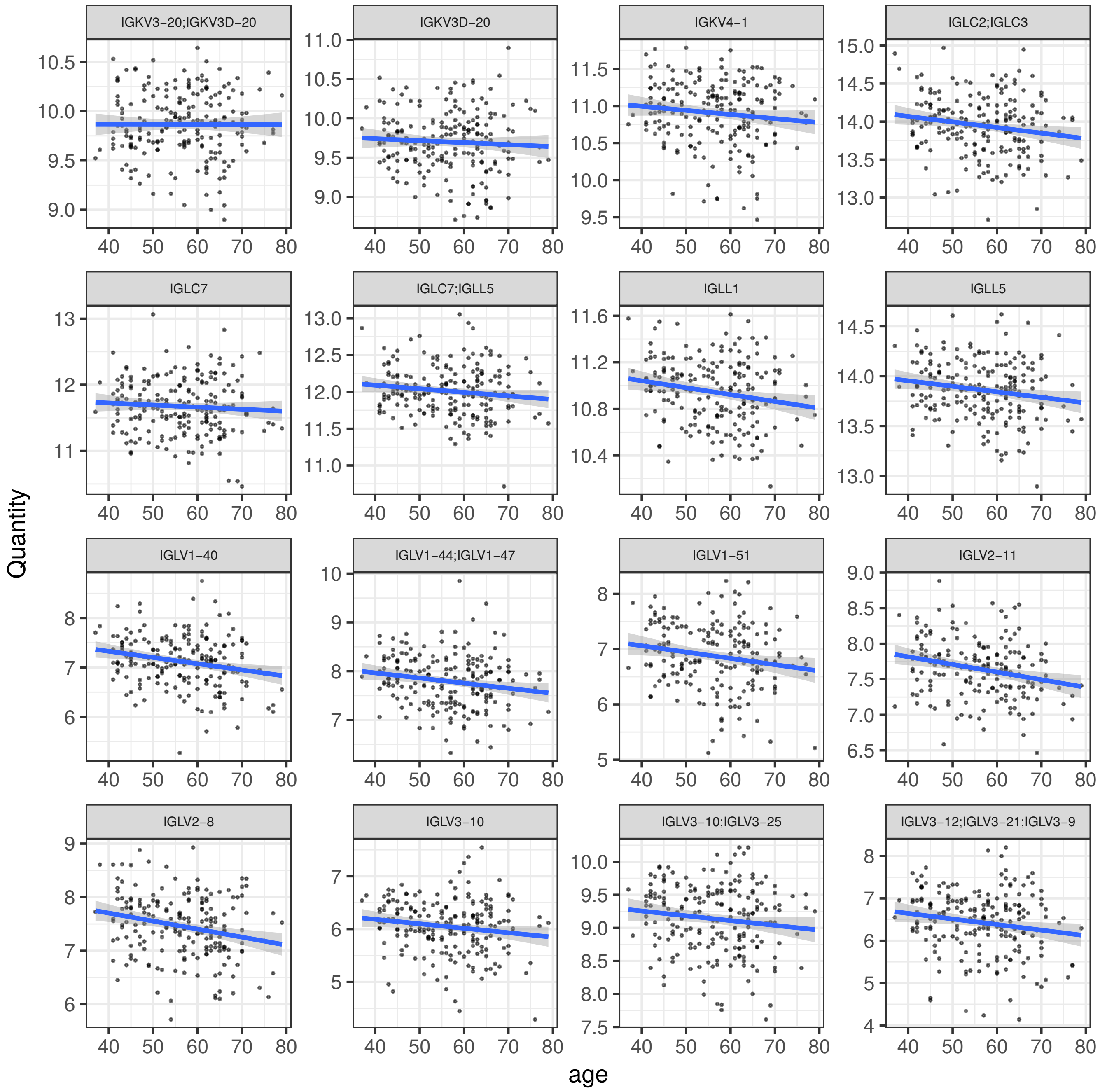
IGKV3-20;IGKV3D-20
IGKV3D-20
IGKV4-1
IGLC2;IGLC3
IGLC7
IGLC7;IGLL5
IGLL1
IGLL5
IGLV1-40
IGLV1-44;IGLV1-47
IGLV1-51
IGLV2-11
IGLV2-8
IGLV3-10
IGLV3-10;IGLV3-25
IGLV3-12;IGLV3-21;IGLV3-9
Quantity
age

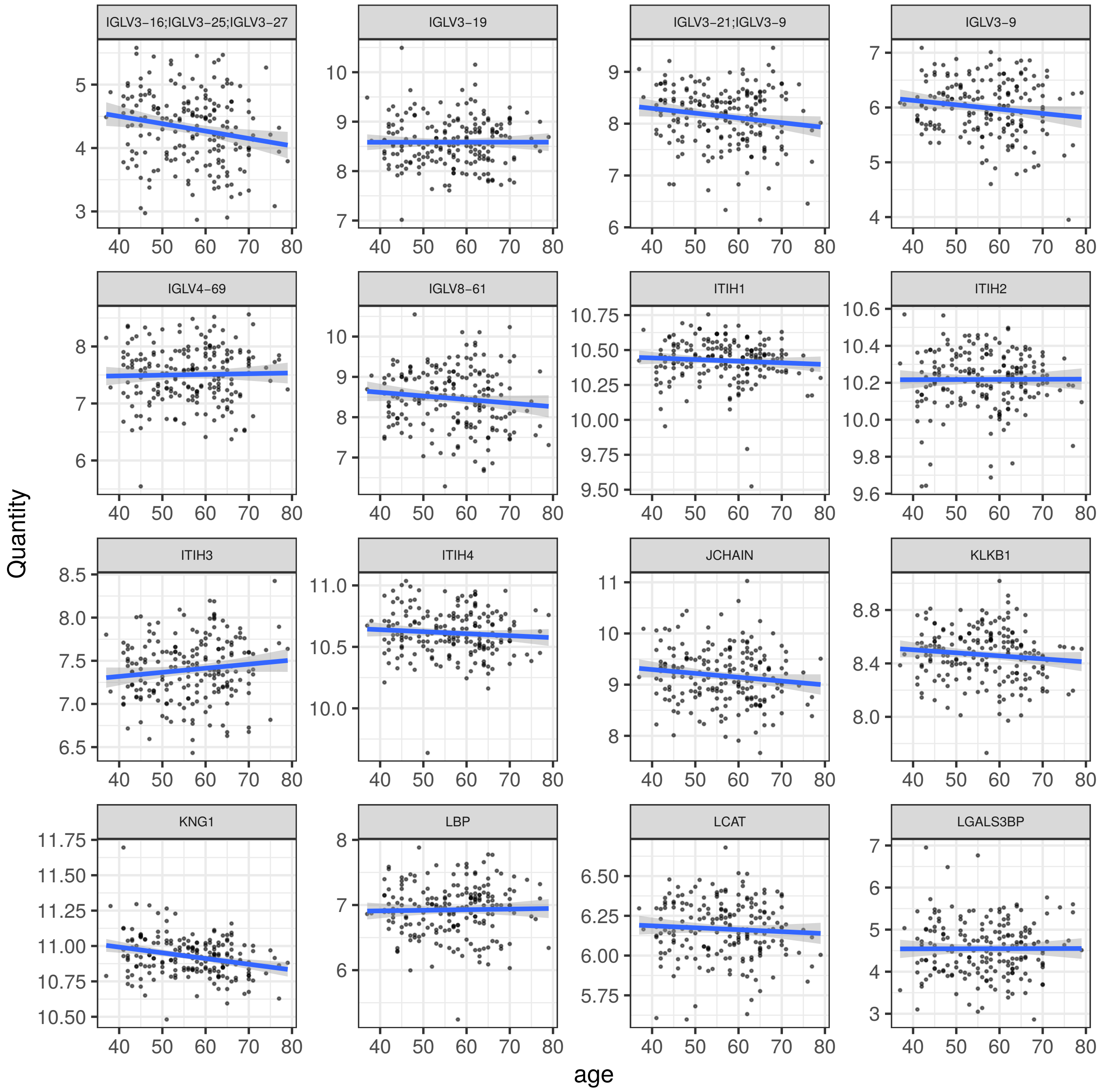
IGLV3−16;IGLV3−25;IGLV3−27
IGLV3−19
IGLV3−21;IGLV3−9
IGLV3−9
IGLV4−69
IGLV8−61
ITIH1
ITIH2
ITIH3
ITIH4
JCHAIN
KLKB1
KNG1
LBP
LCAT
LGALS3BP
Quantity
age

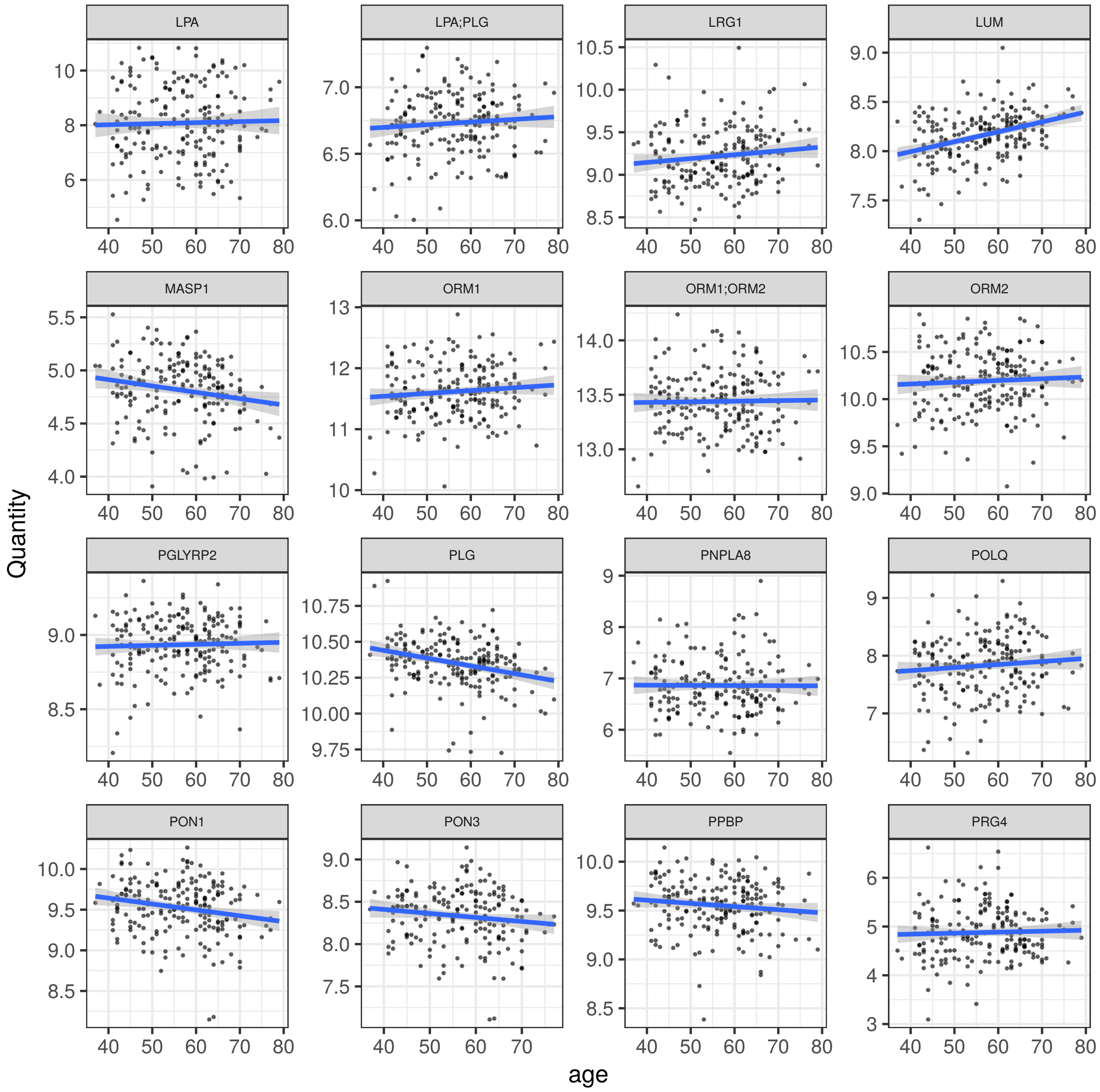
LPA
LPA;PLG
LRG1
LUM
MASP1
ORM1
ORM1;ORM2
ORM2
PGLYRP2
PLG
PNPLA8
POLQ
PON1
PON3
PPBP
PRG4
Quantity
age

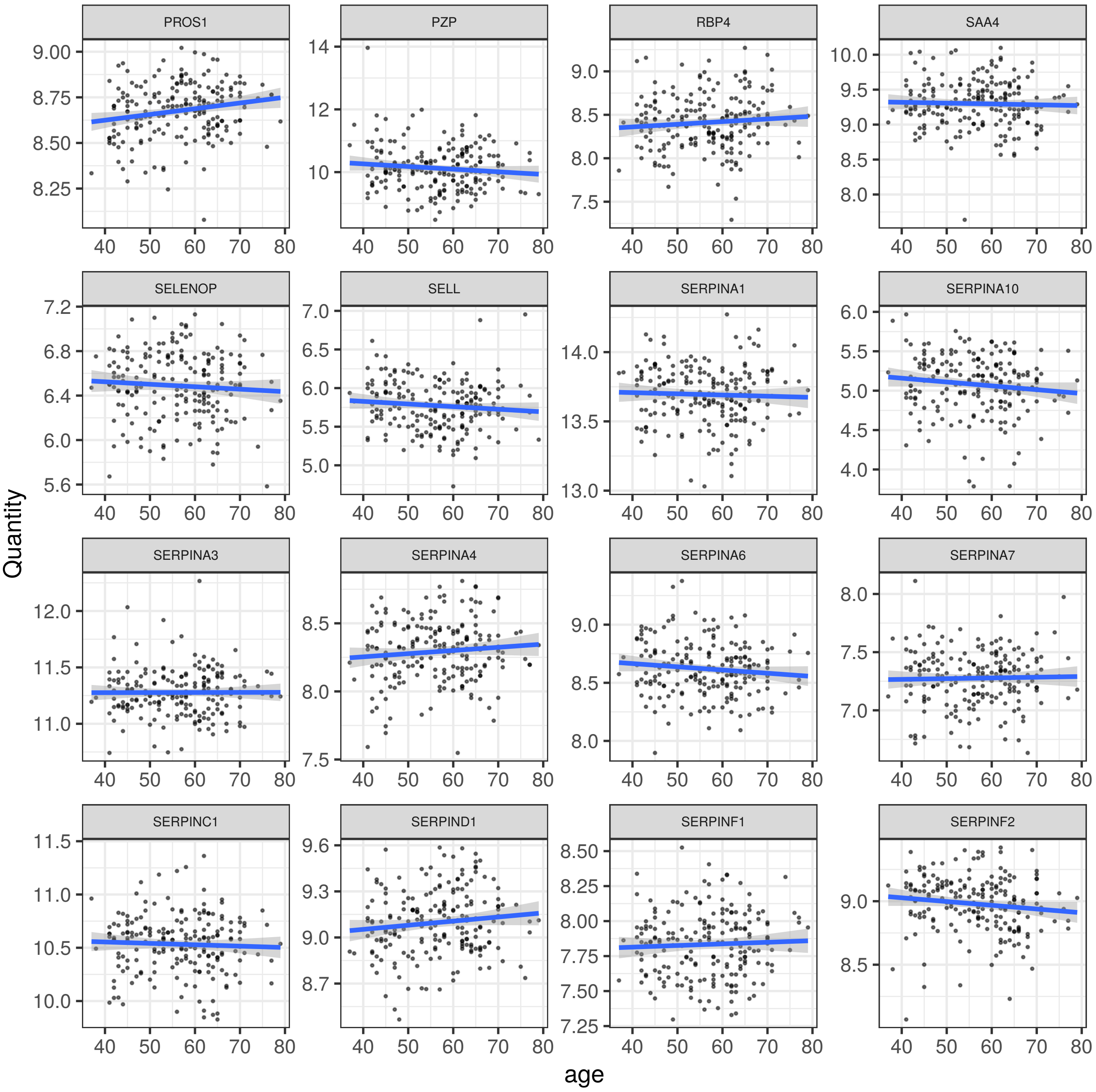
PROS1
PZP
RBP4
SAA4
SELENOP
SELL
SERPINA1
SERPINA10
SERPINA3
SERPINA4
SERPINA6
SERPINA7
SERPINC1
SERPIND1
SERPINF1
SERPINF2
Quantity
age

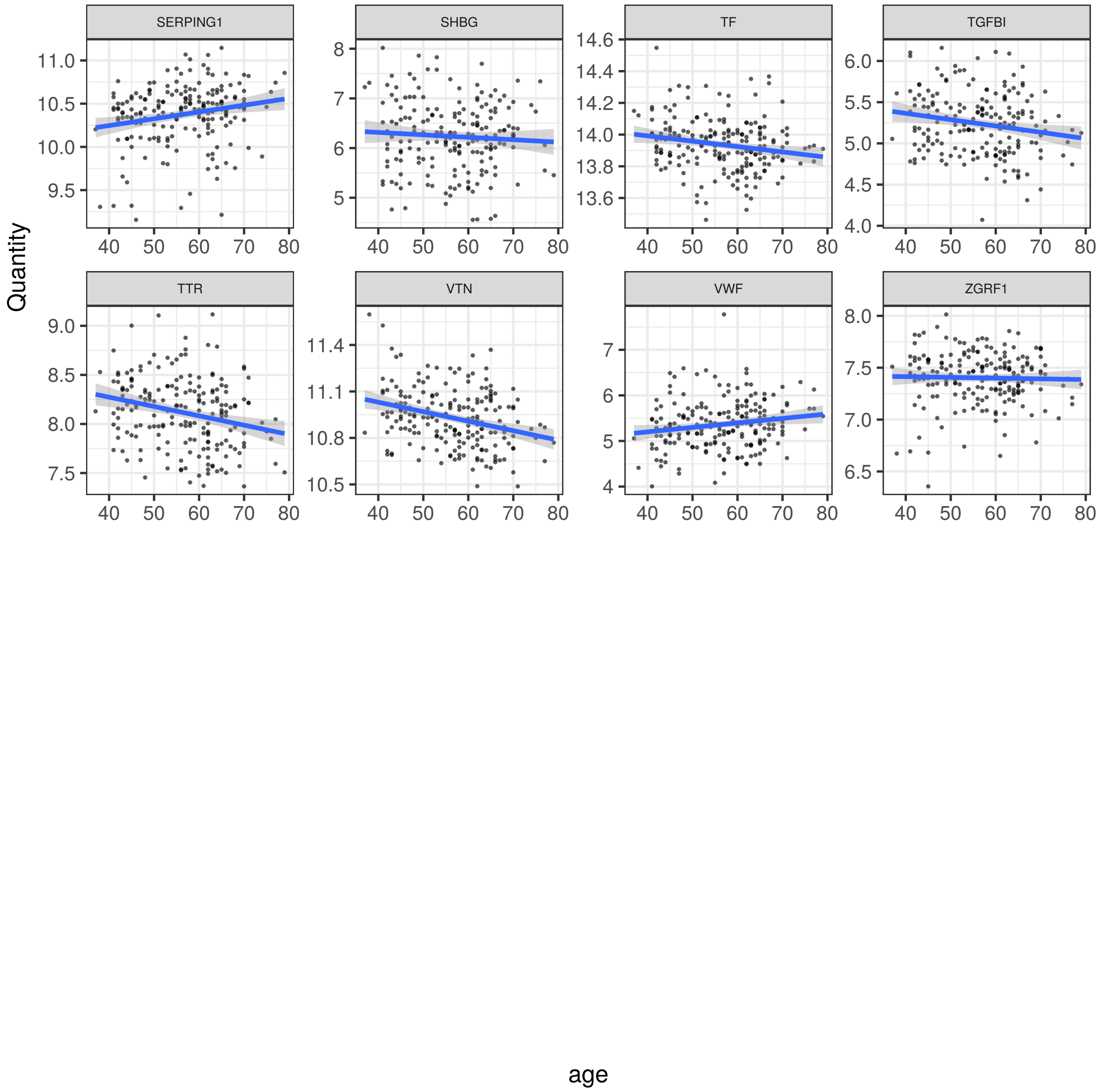

SERPING1
SHBG
TF
TGFBI
TTR
VTN
VWF
ZGRF1
Quantity
age

References Supplemental Information

Almawi, W.Y., Al-Shaikh, F.S., Melemedjian, O.K., and Almawi, A.W. (2013). Protein Z, an anticoagulant protein with expanding role in reproductive biology. Reproduction *146*, R73–R80.

Anas, A., van der Poll, T., and de Vos, A.F. (2010). Role of CD14 in lung inflammation and infection. Crit. Care *14*, 209.

Asare-Werehene, M., Communal, L., Carmona, E., Le, T., Provencher, D., Mes-Masson, A.-M., and Tsang, B.K. (2019). Pre-operative Circulating Plasma Gelsolin Predicts Residual Disease and Detects Early Stage Ovarian Cancer. Sci. Rep. *9*, 13924.

Bas, S., Gauthier, B.R., Spenato, U., Stingelin, S., and Gabay, C. (2004). CD14 is an acute-phase protein. J. Immunol. *172*, 4470–4479.

Bhanumathy, C.D., Tang, Y., Monga, S.P.S., Katuri, V., Cox, J.A., Mishra, B., and Mishra, L. (2002). Itih-4, a serine protease inhibitor regulated in interleukin-6--dependent liver formation: role in liver development and regeneration. Dev. Dyn. *223*, 59–69.

Cheng, Y., Hu, X., Liu, C., Chen, M., Wang, J., Wang, M., Gao, F., Han, J., Zhang, C., Sun, D., et al. (2017). Gelsolin Inhibits the Inflammatory Process Induced by LPS. Cell. Physiol. Biochem. *41*, 205–212.

Cox, J., Hein, M.Y., Luber, C.A., Paron, I., Nagaraj, N., and Mann, M. (2014). Accurate proteome-wide label-free quantification by delayed normalization and maximal peptide ratio extraction, termed MaxLFQ. Mol. Cell. Proteomics *13*, 2513–2526.

DiNubile, M.J. (2008). Plasma gelsolin as a biomarker of inflammation. Arthritis Res. Ther. *10*, 124.

Druhan, L.J., Lance, A., Li, S., Price, A.E., Emerson, J.T., Baxter, S.A., Gerber, J.M., and Avalos, B.R. (2017). Leucine Rich α-2 Glycoprotein: A Novel Neutrophil Granule Protein and Modulator of Myelopoiesis. PLoS One *12*, e0170261.

Fotakis, P., Kothari, V., Thomas, D.G., Westerterp, M., Molusky, M.M., Altin, E., Abramowicz, S., Wang, N., He, Y., Heinecke, J.W., et al. (2019). Anti-Inflammatory Effects of HDL (High-Density Lipoprotein) in Macrophages Predominate Over Proinflammatory Effects in Atherosclerotic Plaques. Arterioscler. Thromb. Vasc. Biol. *39*, e253–e272.

Fuior, E.V., and Gafencu, A.V. (2019). Apolipoprotein C1: Its Pleiotropic Effects in Lipid Metabolism and Beyond. Int. J. Mol. Sci. *20*, 5939.

Gordon, S.M., Hofmann, S., Askew, D.S., and Davidson, W.S. (2011). High density lipoprotein: it’s not just about lipid transport anymore. Trends Endocrinol. Metab. *22*, 9–15.

Hagihara, K., Nishikawa, T., Isobe, T., Song, J., Sugamata, Y., and Yoshizaki, K. (2004). IL-6 plays a critical role in the synergistic induction of human serum amyloid A (SAA) gene when stimulated with proinflammatory cytokines as analyzed with an SAA isoform real-time quantitative RT-PCR assay system. Biochemical and Biophysical Research Communications *314*, 363–369.

Hajishengallis, G., Reis, E.S., Mastellos, D.C., Ricklin, D., and Lambris, J.D. (2017). Novel mechanisms and functions of complement. Nat. Immunol. *18*, 1288–1298.

Hamm, A., Veeck, J., Bektas, N., Wild, P.J., Hartmann, A., Heindrichs, U., Kristiansen, G., Werbowetski-Ogilvie, T., Del Maestro, R., Knuechel, R., et al. (2008). Frequent expression loss of Inter-alpha-trypsin inhibitor heavy chain (ITIH) genes in multiple human solid tumors: a systematic expression analysis. BMC Cancer *8*, 25.

Honda, H., Fujimoto, M., Serada, S., Urushima, H., Mishima, T., Lee, H., Ohkawara, T., Kohno, N., Hattori, N., Yokoyama, A., et al. (2017). Leucine-rich α-2 glycoprotein promotes lung fibrosis by modulating TGF-β signaling in fibroblasts. Physiological Reports *5*, e13556.

Hu, W., Yen, Y.-T., Singh, S., Kao, C.-L., and Wu-Hsieh, B.A. (2012). SARS-CoV Regulates Immune Function-Related Gene Expression in Human Monocytic Cells. Viral Immunology *25*, 277–288.

Jain, S., Gautam, V., and Naseem, S. (2011). Acute-phase proteins: As diagnostic tool. J. Pharm. Bioallied Sci. *3*, 118–127.

Khatri, N., Sagar, A., Peddada, N., Choudhary, V., Chopra, B.S., Garg, V., Garg, R., and Ashish (2014). Plasma gelsolin levels decrease in diabetic state and increase upon treatment with F-actin depolymerizing versions of gelsolin. J Diabetes Res *2014*, 152075.

Kong, Z., Zhou, C., Chen, L., Ren, A., Zhang, D., Basang, Z., Tan, Z., Kang, J., and Li, B. (2019). Multi-Omics Analysis Reveals Up-Regulation of APR Signaling, LXR/RXR and FXR/RXR Activation Pathways in Holstein Dairy Cows Exposed to High-Altitude Hypoxia. Animals (Basel) *9*, 406.

Kumaraswamy, S.B., Linder, A., Åkesson, P., and Dahlbäck, B. (2012). Decreased plasma concentrations of apolipoprotein M in sepsis and systemic inflammatory response syndromes. Crit. Care *16*, R60.

Lee, P.-S., Waxman, A.B., Cotich, K.L., Chung, S.W., Perrella, M.A., and Stossel, T.P. (2007). Plasma gelsolin is a marker and therapeutic agent in animal sepsis. Crit. Care Med. *35*, 849–855.

Li, G.H., Arora, P.D., Chen, Y., McCulloch, C.A., and Liu, P. (2012). Multifunctional roles of gelsolin in health and diseases. Med. Res. Rev. *32*, 999–1025.

Li, S., Lee, C., Hsu, C., Huang, H., and Su, Y. (2020). IL-6 induces haptoglobin expression through activating STAT3 in human head and neck cancer. J. Oral Pathol. Med. *49*, 49–54.

Macpherson, M.E., Halvorsen, B., Yndestad, A., Ueland, T., Mollnes, T.E., Berge, R.K., Rashidi, A., Otterdal, K., Gregersen, I., Kong, X.Y., et al. (2019). Impaired HDL Function Amplifies Systemic Inflammation in Common Variable Immunodeficiency. Sci. Rep. *9*, 9427.

Naka, T., and Fujimoto, M. (2018). LRG is a novel inflammatory marker clinically useful for the evaluation of disease activity in rheumatoid arthritis and inflammatory bowel disease. Immunol Med *41*, 62–67.

Qin, C., Zhou, L., Hu, Z., Zhang, S., Yang, S., Tao, Y., Xie, C., Ma, K., Shang, K., Wang, W., et al. (2020). Dysregulation of immune response in patients with COVID-19 in Wuhan, China. Clin. Infect. Dis. ciaa248.

Ricklin, D., Hajishengallis, G., Yang, K., and Lambris, J.D. (2010). Complement: a key system for immune surveillance and homeostasis. Nat. Immunol. *11*, 785–797.

Ricklin, D., Reis, E.S., and Lambris, J.D. (2016). Complement in disease: a defence system turning offensive. Nat. Rev. Nephrol. *12*, 383–401.

Ruan, Q., Yang, K., Wang, W., Jiang, L., and Song, J. (2020). Correction to: Clinical predictors of mortality due to COVID-19 based on an analysis of data of 150 patients from Wuhan, China. Intensive Care Med. 1–3.

Sack, G.H. (2018). Serum amyloid A – a review. Molecular Medicine *24*, 1–27.

Shi, H., Han, X., Jiang, N., Cao, Y., Alwalid, O., Gu, J., Fan, Y., and Zheng, C. (2020). Radiological findings from 81 patients with COVID-19 pneumonia in Wuhan, China: a descriptive study. Lancet Infect. Dis. *20*, 425–434.

Shirai, R., Hirano, F., Ohkura, N., Ikeda, K., and Inoue, S. (2009). Up-regulation of the expression of leucine-rich alpha(2)-glycoprotein in hepatocytes by the mediators of acute-phase response. Biochem. Biophys. Res. Commun. *382*, 776–779.

Shive, C.L., Jiang, W., Anthony, D.D., and Lederman, M.M. (2015). Soluble CD14 is a nonspecific marker of monocyte activation. AIDS *29*, 1263–1265.

Silverman, A.M., Nakata, R., Shimada, H., and Sposto, R. (2012). A Galectin-3–Dependent Pathway Upregulates Interleukin-6 in the Microenvironment of Human Neuroblastoma. Cancer Res. *72*, 2228–2238.

Sirniö, P., Väyrynen, J.P., Klintrup, K., Mäkelä, J., Mäkinen, M.J., Karttunen, T.J., and Tuomisto, A. (2017). Decreased serum apolipoprotein A1 levels are associated with poor survival and systemic inflammatory response in colorectal cancer. Sci. Rep. *7*, 5374.

Soeters, P.B., Wolfe, R.R., and Shenkin, A. (2019). Hypoalbuminemia: Pathogenesis and Clinical Significance. JPEN J. Parenter. Enteral Nutr. *43*, 181–193.

Stock, A.-M., Klee, F., Edlund, K., Grinberg, M., Hammad, S., Marchan, R., Cadenas, C., Niggemann, B., Zänker, K.S., Rahnenführer, J., et al. (2015). Gelsolin Is Associated with Longer Metastasis-free Survival and Reduced Cell Migration in Estrogen Receptor-positive Breast Cancer. Anticancer Res. *35*, 5277–5285.

Tani, S., Nagao, K., and Hirayama, A. (2016). Association of systemic inflammation with the serum apolipoprotein A-1 level: A cross-sectional pilot study. J. Cardiol. *68*, 168–177.

Wang, X., Abraham, S., McKenzie, J.A.G., Jeffs, N., Swire, M., Tripathi, V.B., Luhmann, U.F.O., Lange, C.A.K., Zhai, Z., Arthur, H.M., et al. (2013). LRG1 promotes angiogenesis by modulating endothelial TGF-β signalling. Nature *499*, 306–311.

White, R., Giordano, S., and Datta, G. (2017). Role of HDL-Associated Proteins and Lipids in

the Regulation of Inflammation. In Advances in Lipoprotein Research, T. Isbir, ed. (intechopen), p. 53.

Wittmann, J., Dieckow, J., Schröder, H., Hampel, U., Garreis, F., Jacobi, C., Milczarek, A., Hsieh, K.L., Pulli, B., Chen, J.W., et al. (2018). Plasma gelsolin promotes re-epithelialization. Sci. Rep. *8*, 13140.

World Health Organisation (2020). WHO R&D Blueprint novel Coronavirus COVID-19 Therapeutic Trial Synopsis.

Xu, G., Xia, Z., Deng, F., Liu, L., Wang, Q., Yu, Y., Wang, F., Zhu, C., Liu, W., Cheng, Z., et al. (2019). Inducible LGALS3BP/90K activates antiviral innate immune responses by targeting TRAF6 and TRAF3 complex. PLoS Pathog. *15*, e1008002.

Zhou, F., Yu, T., Du, R., Fan, G., Liu, Y., Liu, Z., Xiang, J., Wang, Y., Song, B., Gu, X., et al. (2020). Clinical course and risk factors for mortality of adult inpatients with COVID-19 in Wuhan, China: a retrospective cohort study. Lancet *395*, 1054–1062.
