# Supplementary material for: Ultra-High-Throughput Clinical Proteomics Reveals Classifiers of COVID-19 Infection
Source: Cell Syst. 2020 Jul 22;11(1):11–24.e4. doi: 10.1016/j.cels.2020.05.012 (PMC7264033; doi:10.1016/j.cels.2020.05.012)
Supplement: Document S2. Article plus Supplemental Information [file mmc2.pdf]

Cell Systems

Article

Ultra-High-Throughput Clinical Proteomics Reveals Classifiers of COVID-19 Infection

Graphical Abstract

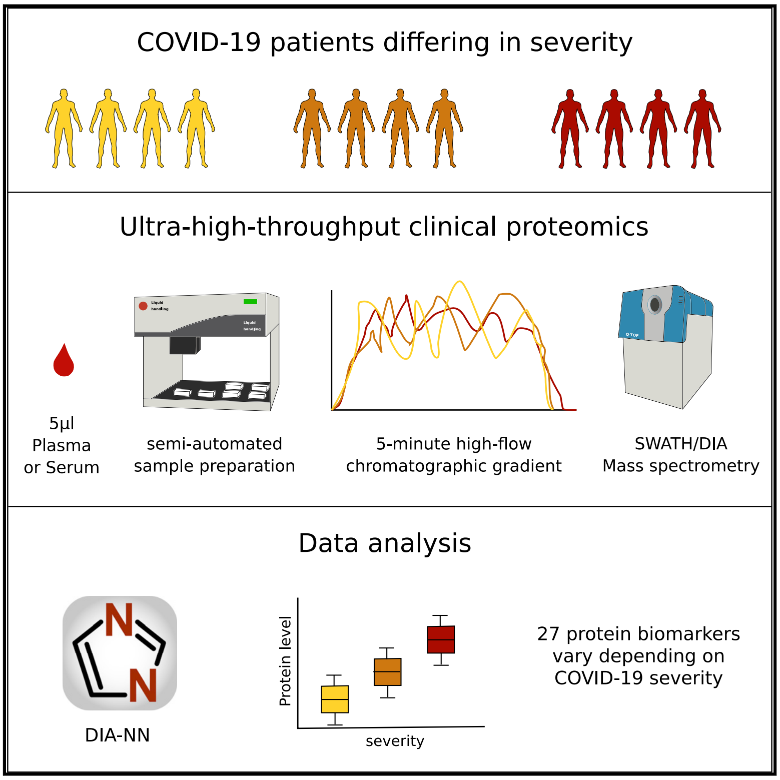

Highlights

- A standardized, ultra-high-throughput clinical platform for serum and plasma proteomics
- Platform enables high precision quantification of 180 human proteomes per day at low cost
- 27 biomarkers are differentially expressed between WHO severity grades for COVID-19
- Biomarkers include proteins not previously associated with COVID-19 infection

Authors

Christoph B. Messner,
Vadim Demichev, Daniel Wendisch, ...,
Florian Kurth, Leif Erik Sander,
Markus Ralser

Correspondence

markus.ralser@charite.de

In Brief

Messner et al. present a standardized, low-cost, ultra-high-throughput platform for serum and plasma proteomics designed for clinical use and apply it to a cohort of hospitalized COVID-19 patients. They identify 27 potential biomarkers that are differentially expressed depending on the WHO severity grade of COVID-19.

Messner et al., 2020, Cell Systems *11*, 11–24
July 22, 2020 © 2020 The Author(s). Published by Elsevier Inc.
https://doi.org/10.1016/j.cels.2020.05.012

CellPress

Cell Systems

CellPress
OPEN ACCESS

Article

Ultra-High-Throughput Clinical Proteomics Reveals Classifiers of COVID-19 Infection

Christoph B. Messner,[1,15] Vadim Demichev,[1,2,15] Daniel Wendisch,[3] Laura Michalick,[4] Matthew White,[1] Anja Freiwald,[5,6] Kathrin Textoris-Taube,[5] Spyros I. Vernardis,[1] Anna-Sophia Egger,[1] Marco Kreidl,[1] Daniela Ludwig,[6] Christiane Kilian,[6] Federica Agostini,[6] Aleksej Zelezniak,[1,7] Charlotte Thibeault,[3] Moritz Pfeiffer,[3] Stefan Hippenstiel,[3] Andreas Hocke,[3] Christof von Kalle,[8] Archie Campbell,[9,10] Caroline Hayward,[11] David J. Porteous,[9] Riccardo E. Marioni,[9] Claudia Langenberg,[1,12] Kathryn S. Lilley,[2] Wolfgang M. Kuebler,[4] Michael Mülleder,[5] Christian Drosten,[13] Norbert Suttorp,[3] Martin Witzenrath,[3] Florian Kurth,[3,14] Leif Erik Sander,[3] and Markus Ralser[1,6,16,*]

[1]The Francis Crick Institute, Molecular Biology of Metabolism Laboratory, London NW11AT, UK
[2]Department of Biochemistry, The University of Cambridge, Cambridge CB21GA, UK
[3]Charité Universitätsmedizin, Berlin, Department of Infectious Diseases and Respiratory Medicine, 10117 Berlin, Germany
[4]Charité Universitätsmedizin, Institute of Physiology, 10117 Berlin, Germany
[5]Charité Universitätsmedizin, Core Facility - High-Throughput Mass Spectrometry, 10117 Berlin, Germany
[6]Charité Universitätsmedizin, Department of Biochemistry, 10117 Berlin, Germany
[7]Department of Biology and Biological Engineering, Chalmers University of Technology, Gothenburg 412 96, Sweden
[8]Berlin Institute of Health (BIH) and Charité Universitätsmedizin, Clinical Study Center (CSC), 10117 Berlin, Germany
[9]Centre for Genomic and Experimental Medicine, Institute of Genetics and Molecular Medicine, University of Edinburgh, Edinburgh EH4 2XU, UK
[10]Usher Institute, University of Edinburgh, Nine, Edinburgh Bioquarter, 9 Little France Road, Edinburgh EH16 4UX, UK
[11]MRC Human Genetics Unit, Institute of Genetics and Molecular Medicine, University of Edinburgh, Edinburgh EH4 2XU, UK
[12]MRC Epidemiology Unit, Institute of Metabolic Science, University of Cambridge, Cambridge CB2 0QQ, UK
[13]Charité Universitätsmedizin, Department of Virology, 10117 Berlin, Germany
[14]Department of Tropical Medicine, Bernhard Nocht Institute for Tropical Medicine, Hamburg, Germany
[15]These authors contributed equally
[16]Lead Contact
*Correspondence: markus.ralser@charite.de
https://doi.org/10.1016/j.cels.2020.05.012

SUMMARY

The COVID-19 pandemic is an unprecedented global challenge, and point-of-care diagnostic classifiers are urgently required. Here, we present a platform for ultra-high-throughput serum and plasma proteomics that builds on ISO13485 standardization to facilitate simple implementation in regulated clinical laboratories. Our low-cost workflow handles up to 180 samples per day, enables high precision quantification, and reduces batch effects for large-scale and longitudinal studies. We use our platform on samples collected from a cohort of early hospitalized cases of the SARS-CoV-2 pandemic and identify 27 potential biomarkers that are differentially expressed depending on the WHO severity grade of COVID-19. They include complement factors, the coagulation system, inflammation modulators, and pro-inflammatory factors upstream and downstream of interleukin 6. All protocols and software for implementing our approach are freely available. In total, this work supports the development of routine proteomic assays to aid clinical decision making and generate hypotheses about potential COVID-19 therapeutic targets.

INTRODUCTION

The ongoing SARS-CoV-2 pandemic has highlighted the pressing need for technologies that can accelerate our understanding of emerging diseases in order to (1) find markers that define disease severity, have prognostic value, or define a specific phase of the disease; (2) identify preventive strategies; and (3) discover therapeutic targets. PCR-based diagnostics can be implemented and scaled quickly but do not provide information about severity of the disease as well as likely illness trajectories (Chen et al., 2020; Corman et al., 2020). Furthermore, conventional biomarker as well as serological assays depend on affinity reagents, such as antibodies. Developing these takes time and requires prior knowledge of epitopes and the disease mechanisms (Petherick, 2020). Indeed, the host response to each viral infection is significantly different, specifically, as several viruses can evade the host immune system (Bussey and Brinkmann, 2018; Kikkert, 2020). Each novel infective agent requires a new assessment of the host response, as well as requires a unique set of biomarkers for predicting disease trajectories.

Mass spectrometry (MS)-based proteomics does not depend on affinity reagents and can be set up in an untargeted fashion,

This is an open access article under the CC BY license (http://creativecommons.org/licenses/by/4.0/).

such that it does not depend on prior knowledge of the disease. It can quickly deliver substantial amounts of clinical and biological information from accessible biological material, such as blood plasma or serum. MS-based proteomics hence has the potential to become an ideal technology to be applied in situations when rapid responses are required. Currently, MS-based proteomic workflows are well established in research laboratories, where they are routinely used for biomarker discovery and profiling (Bruderer et al., 2019; Geyer et al., 2016a, 2016b, 2017, 2019; Liu et al., 2015; Niu et al., 2019; Wewer Albrechtsen et al., 2018).

Increasingly, MS-based proteomics is also entering regulated clinical and diagnostic environments (He, 2019; Mischak et al., 2012; Crutchfield et al., 2016). It has the potential to yield complex and predictive biomarker signatures that support clinical decision making, as well as to enable the prediction of patient trajectories via machine learning methods with datasets of sufficient depth and size (Ahadi et al., 2020). However, in clinical practice, its potential is yet to be completely realized. For routine applications, MS-based proteomic methods must combine precision, reproducibility, and robustness with low cost and high throughput, such that results can be routinely compared within and between clinical studies and laboratories (Geyer et al., 2017, 2019; Nilsson et al., 2010; Wright and Van Eyk, 2017). These requirements might necessitate a compromise with proteomic depth, which has often been a key objective of proteomics in research settings, but which has been, in turn, often achieved through long measurement times and high cost (Bruderer et al., 2019; Geyer et al., 2016a; Liu et al., 2015). A further hurdle is that current MS-based proteomic workflows require, partially due to their dependency on chromatographic flow rates in the range of nanoliters or low microliters per minute, a high level of expert knowledge to achieve the level of necessary robustness for implementation in the clinical laboratory.

Here, we present a redesigned high-throughput MS platform that enables the cost-effective (less than 10€ for consumables per sample) in-depth analysis of disease susceptibility and progression in patients as well as biomarker discovery. Our platform is optimized at all steps from sample preparation, chromatography, and data acquisition to data processing, in comparison to existing research pipelines (Bache et al., 2018; Bekker-Jensen et al., 2020; Bian et al., 2020; Bluemlein and Ralser, 2011; Bruderer et al., 2019; Geyer et al., 2016a; Liu et al., 2015; Müller et al., 2020; Vowinckel et al., 2018). It includes a semi-automated sample preparation workflow that scales to high sample numbers through the use of liquid handling robotics and minimum hands-on-time and includes effective strategies to mitigate longitudinal batch effects. It also makes use of short-gradient high-flow liquid chromatography (LC), a technology that is the basis of several FDA-approved clinical assays (Grebe and Singh, 2011; Nair and Clarke, 2016), applicable to high-throughput proteomic experiments. Using this approach, we were able to reduce measurements to 5-min gradient length, inter-runtime to 3 min or less, as well as to use flow rates of 800 μL/min, thereby substantially increasing sample turnover and reducing costs, while increasing stability and precision.

We first benchmarked the platform on a cohort-based epidemiological study, Generation Scotland (GS) (Smith et al., 2013), and at considerably higher throughput, demonstrated a level of precision and consistency that, to our knowledge, is yet unachieved in comparable large-scale proteomic studies. We then employed this workflow in an immediate response to the SARS-CoV-2 pandemic outbreak in Germany, by applying it to a cohort that includes the first COVID-19 patients hospitalized at the Charité Universitätsmedizin Berlin. We measured samples from a primary exploratory cohort comprising thirty-one COVID-19 patients to identify clinical classifiers, candidate biomarkers as well as potential targets that picture the host immune response specific to a SARS-CoV-2 infection (Figure 4A). We validated these on a smaller cohort of seventeen independent patients and fifteen healthy volunteers (Table S1). We identified protein expression signatures that can classify COVID-19 patients according to WHO grading, introduced as of April 2020 (World Health Organization, 2020).

Our analysis associates several proteins with COVID-19 severity that have not been previously associated with the infection and the host response. These are alpha-1B-glycoprotein (A1BG), beta and gamma-1 actin (ACTB;ACTG1), monocyte differentiation antigen and lipopolysaccharide co-receptor CD14, lipopolysaccharide-binding protein (LBP), galectin 3-binding protein (LGALS3BP), leucine-rich alpha-2-glycoprotein (LRG1), haptoglobin (HP), protein Z-dependent protease inhibitor (SERPINA10), apolipoprotein C1 (APOC1), gelsolin (GSN), and transferrin (TF). Our results highlight the role of complement factors, the coagulation system, several inflammation modulators as well as pro-inflammatory signaling both upstream and downstream of interleukin (IL)-6. In addition, our study provided evidence that proteomic signatures have the potential to outperform conventional clinical assays. Two individuals with differing proteomic signatures were identified through a clustering approach. In one case, a clinical re-assessment changed the diagnosis (the patient was in fact suffering from an influenza type B infection) and revealed in the other case a strong comorbidity caused by anti-cancer chemotherapy. None of the currently applied clinical tests spotted this situation.

In total, our study demonstrates the value and power of robust high-throughput MS in a global public health crisis. Very fast and reliable proteome technologies can play a vital role both in clinical classification as well as in the rapid identification of therapeutic targets against arising infecting agents.

RESULTS AND DISCUSSION

A Platform for Clinical Proteomics Yields High Quantitative Precision at Low Costs and High Throughput

We addressed throughput, precision, costs, and practical hurdles in clinical implementation of MS-based proteomics, by designing a proteomics platform, in which we refined sample preparation, chromatography, mass spectrometric acquisition, and data analysis. Our workflow reaches a high level of standardization and documentation, for which ISO 13485 was used as a reference (Figure S1). After the transfer of the clinical plasma or serum samples, obtained with standard operating procedures, to 96-well plates, all pipetting and mixing steps are conducted with liquid handling robots. The workflow has a total hands-on time of around 3.5 h and is designed so that a single person using a single liquid handling unit can start and complete

every day one or two 4-plate batches. Effectively four to eight plates containing up to 768 proteome samples exit the workflow every day and are ready for the mass spectrometric analysis.

Among several improvements in handling (see STAR Methods for details), the preparation workflow includes a simple but effective handling improvement that mitigates batch variation in sample preparation reagents, which has proven so far a major and prohibitive contributor to quantification inconsistencies in (large-scale) proteomic experiments (Fu et al., 2018; Lowenthal et al., 2014; Piehowski et al., 2013). Instead of pipetting reagents on the samples at each step, initial common stock solutions (urea and ammonium bicarbonate buffer, dithiothreitol, iodoacetamide, formic acid, and trypsin) are pre-filled into multi-well plates and are then stored at −80°C for whole projects in perpetuity. These plates enter the workflow at different stages (Figure 1), thereby not only reducing hands-on time but maintaining the exact same reagents for projects of, in theory, unlimited scale. A second key step concerns the cleanup of the digested peptides, which is done with 96-well solid-phase extraction plates (Bennike et al., 2018; Bruderer et al., 2019). In our workflow, four are processed in parallel to reduce technical variability. Finally, the inclusion of sample preparation controls on each plate enables cross-batch normalization, to correct batch effects in case these emerge at the sample preparation or the acquisition step of the workflow (Figure 1).

Next, we developed a data acquisition scheme that could be implemented in regulated environments without major hurdles and maintains very low variability across large sample series. Here, we focused on the implementation of a high-flow chromatographic regime in a proteomic workflow. The current standard technique for bottom-up proteomics, nano-flow LC, used owing to its high sensitivity, is a main contributor to batch variability in LC-MS experiments (Gama et al., 2013; Shishkova et al., 2016). Several recent studies have shown that run-to-run variability improves by switching from nano-flow to micro-flow regimes or to specialized chromatographic devices that operate with pre-formed gradients. These allow faster runtimes and sample turnover, show better retention time stability, and improve column lifetime (Bache et al., 2018; Bian et al., 2020; Bruderer et al., 2019; Vowinckel et al., 2018).

Alternatively, high-flow LC (also known as analytical LC) in conjunction with very fast chromatographic gradients, a technology that reaches the requirements of regulated clinical laboratories, could further and substantially improve throughput and chromatographic properties. However, typically it has not been applied to short-gradient proteomics for two important reasons. First, on this type of fast chromatography, conventional mass spectrometric acquisition schemes do not reach sufficient sampling velocity in data-dependent mode (as peaks elute too fast). Second, when using data-independent acquisition (DIA) schemes, which do not sample each peak individually, conventional software cannot deconvolute the interference-rich short-gradient data produced (Demichev et al., 2020; Messner et al., 2019).

We overcame these issues and developed an acquisition scheme based on 5-min water to acetonitrile chromatographic gradients at a flow rate of 800 μL/min. Separating tryptic digests of non-depleted human plasma using an 1290 Infinity II UPLC (Agilent) or H Class UPLC systems (Waters) coupled to a TripleTOF 6600 (Sciex) mass spectrometer illustrates that total ion chromatograms (TICs) are virtually unchanged over repeated injections (Figure 2A). The nearly complete overlap of the TICs indicates not only the stability of the applied chromatographic system but also the stability of the electrospray, which is facilitated by the gases that assist the desolvation process in high-flow ion sources. By using a short 5-cm column and by increasing the flow rate post-gradient to 1,200 μL/min and 1,000 μL/min during washing and equilibration, respectively, we were able to reduce the total runtime, including overhead, to less than 8 min. In this particular test, the setup allows a theoretical throughput of 180 samples/day on a single mass spectrometer, an at least 5-fold improvement compared with microLC or nanoLC platforms optimized for throughput (Bruderer et al., 2019; Geyer et al., 2016a; Vowinckel et al., 2018). Moreover, the columns used in high-flow chromatography have higher capacities and thus are less prone to carryover. Indeed, blank injection after 10 acquisitions of non-depleted plasma tryptic digests shows no significant carryover even with an applied wash time of less than 1 min (Figure 2A).

The separation of a K562 cell line tryptic digest (Promega) at different gradient lengths illustrates the chromatographic properties achieved. The high-flow setup achieved a median peak full width at half maximum (FWHM) of 3 s with a 20-min gradient length. For comparison, an extensively optimized micro-flow LC (Demichev et al., 2020; Messner et al., 2019), achieved a FWHM of 5 s, at the same gradient length (Figure 2C). Furthermore, high-flow gradients as fast as 5 min resulted in peak capacities comparable to the highly optimized 20-min micro-flow setup (Demichev et al., 2020; Messner et al., 2019) (Figure 2D). In order to achieve a sufficiently fast mass spectrometric duty cycle, we used a q-TOF instrument with a very fast sampling rate (Schilling et al., 2017) and applied SWATH-MS, a DIA method specifically developed to minimize stochastic elements in data acquisition (Gillet et al., 2012; Ludwig et al., 2018). To record sufficient data points per chromatographic peak, we optimized the method for duty cycles of 700 ms and scan a precursor mass range of m/z 450–850 using 25 windows with variable window size (Table S6) and with 25-ms accumulation time.

In order to deconvolute the complex data recorded, we built on our recent developments of DIA-NN software, that includes several algorithms that boost the number of true positive precursor identifications in the short-gradient DIA-MS runs. DIA-NN can handle complex short-gradient data as it contains algorithms that correct for signal interferences and uses deep neural networks to assign confidence scores to peptide-spectrum matches and identify true positive signals (Demichev et al., 2020). Applied to analyzing 5 μg of human cell line (K562) tryptic digest, the short-gradient high-flow method yielded 2,829 unique proteins (at 1% FDR) in triplicate injections, while the numbers of unique proteins quantified with a coefficient of variation (CV) less than 20% and less than 10% were 1,873 and 1,349, respectively (Figure S2E). Hence, despite the ultra-high throughput and the use of high-flow chromatography, the analytical method is able to achieve proteomic depth even on complex samples. Finally, we improved the DIA-NN (version 1.7.10) workflow for the high-throughput processing of plasma and serum proteomes, through integration of the MaxLFQ protein quantification algorithm (Cox et al., 2014) in a DIA-NN R-package. Originally, MaxLFQ was designed for shotgun-MS studies but was

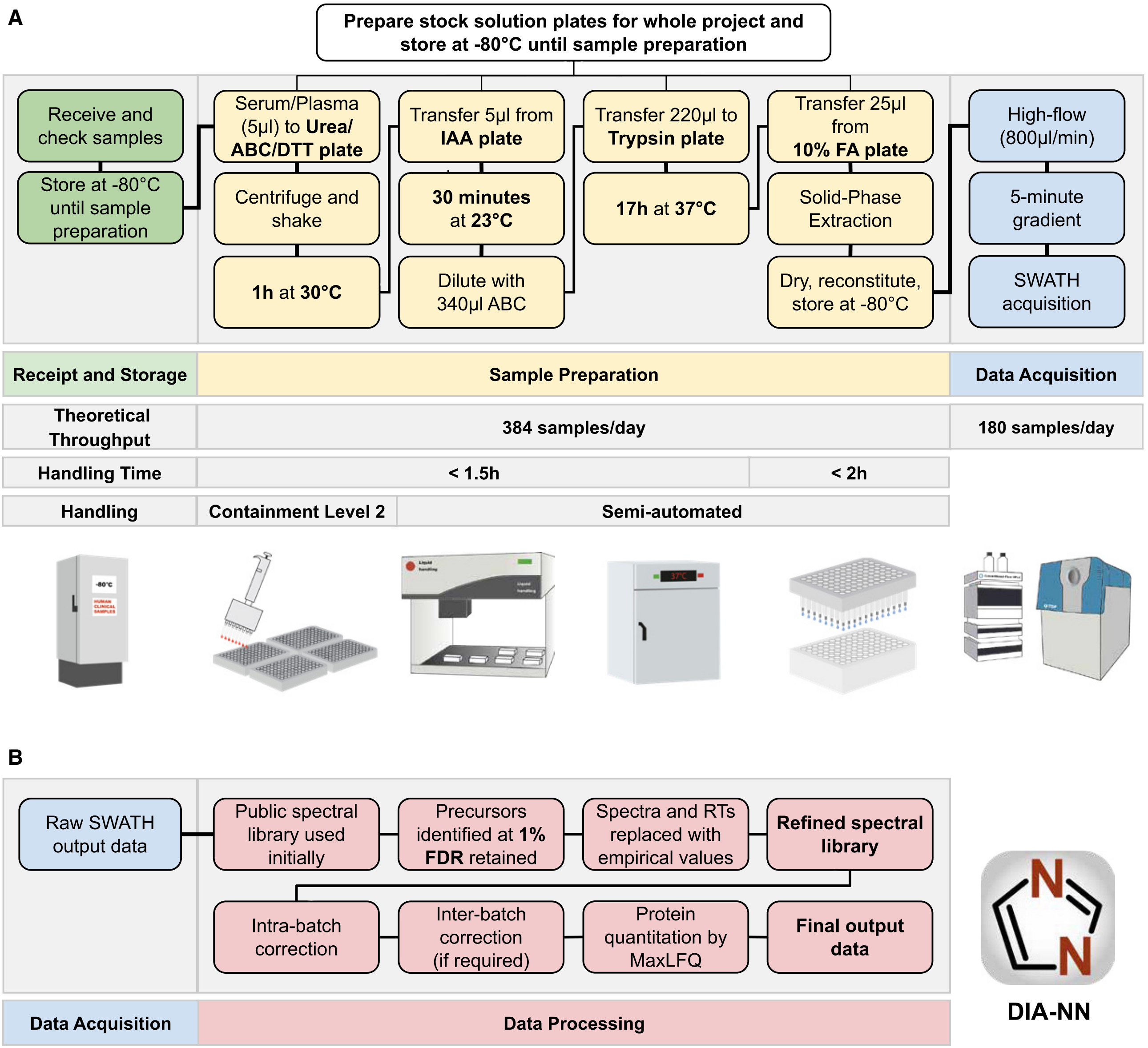

Figure 1. A High-Throughput Proteomics Platform for Large-Scale and Longitudinal Clinical Proteomic Studies

(A) Experimental part of the workflow. Receipt and storage (green boxes): clinical or epidemiological samples are collected using a standard operating procedure, received, and stored at −80°C, then aliquoted to 96-well plates alongside control samples. For plasma and serum, 5 µL are processed and yield sufficient tryptic digest for five measurements on the high-flow rate LC-MS platform. Sample preparation (yellow boxes): the sample preparation workflow is designed for handling 384 samples per batch (four 96-well plates). Batch effects are mitigated by using pre-aliquoted stock solution plates—prepared for whole projects and stored at −80°C—that enter the workflow at different steps, as well as by using a liquid handling robot for pipetting and mixing. Sample cleanup is done with 384 samples/batch by using 96-well solid-phase extraction plates (BioPureSPE, the Nest Group) and a liquid handler for pipetting. The hands-on time for cleanup is <2 h and although the digestion is done overnight, the total hands-on time for the sample preparation is <3.5 h. Data acquisition (blue boxes): ultra-fast measurements of the digested samples are facilitated in 300-s chromatographic gradients using high-flow chromatography (800 µL/min) with a short reversed phase C18 column (50 × 2.1 mm, 1.8 µm particle size) to accelerate equilibration and washing steps. A 700 ms duty cycle, required to record sufficient data points per chromatographic peaks that elute at FWHM of about 3 s is achieved with an optimized SWATH data acquisition method. The theoretical throughput of data acquisition for one mass spectrometer is 180 samples/day.

(B) Data processing (red boxes): the analysis of the highly complex short-gradient DIA data is achieved with an optimized version (1.7.10) of DIA-NN (Demichev et al., 2020). DIA-NN is based on neural networks to enable confident peptide identification with fast gradients and achieves a throughput of >2,000 samples/day on a conventional PC. First, a spectral library is automatically "refined" using the dataset in question: only detectable peptide precursors are retained, and their reference spectra and retention times are replaced with empirically observed. Reanalysis with this refined library is then followed by batch correction and, finally, protein quantification using MaxLFQ (Cox et al., 2014). Abbreviations: ABC, ammonium bicarbonate; DTT, dithiothreitol; IAA, iodoacetamide; FA, formic acid.

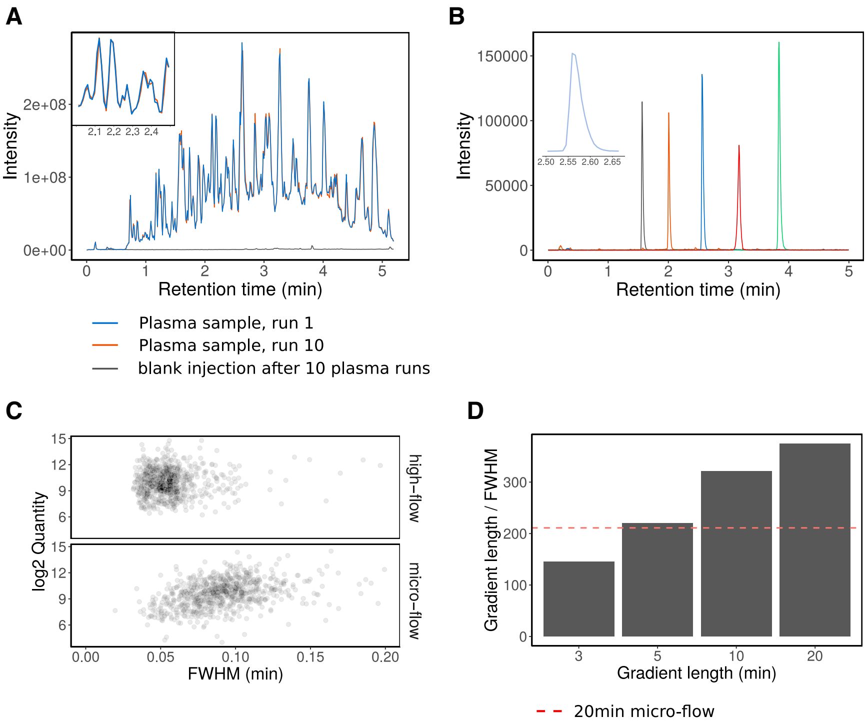

Figure 2. High-Flow LC and Its Application to Short-Gradient MS-Based Proteomics

(A) A tryptic digest of human blood plasma was injected 10 times. The peptides were separated with a 300 s linear water to acetonitrile chromatographic gradient using an Agilent 1290 Infinity II LC system coupled to a TripleTOF 6600 mass analyzer. The TICs of the first and last injection were overlaid and colored with blue and red, respectively. The time from the start of one run to the next was reduced to 8 min (including instrument overheads), which enables a throughput of ~180 samples/day. After the 10 plasma injections, water was injected and the TIC (black line) shows no significant carryover despite the short washing time.

(B) Extracted ion chromatograms of 5 synthetic peptides (AETSELHTSLK [m/z 408.55, black line], LDSTSIPVAK [m/z 519.80, orange line], ALENDIGVPSDATVK [m/z 768.90, blue line], AVYFYAPQIPLYANK [m/z = 883.47, green line], and TVESLFPEEAETPGSAVR [m/z 964.97741, red line]) from a synthetic peptide mixture (Pepcal, Sciex) as separated on the 300 s linear gradient. Chromatograms were extracted from TOF MS data, width = 0.1 Da.

(C) A tryptic digest of K562 human cell lines was separated with a 20-min linear gradient ramping from 3% ACN 0.1% FA to 36% ACN, 0.1% FA on high-flow (800 μL/min; C18 column 50 mm × 2.1, column length). Peak widths at FWHM of the eluting peptides were compared to a 20-min micro-flow run (5 μL/min; 15-cm column; Demichev et al., 2020), analyzed on the same mass spectrometer (Sciex TripleTOF 6600).

(D) Peak capacities (gradient length divided by FWHM) for 3, 5, 10, and 20-min linear gradients (3% ACN/0.1% FA to 36% ACN/0.1% FA) on high-flow compared with 20-min micro-flow chromatographic gradients (red dashed line).

recently introduced to DIA proteomics (Pham et al., 2020) and increased the quantification precision for serum and plasma proteomes.

Benchmarking Acquisition Depth, Quality, and Data Consistency in an Epidemiological Study

To assess the suitability of our high-throughput platform for human blood plasma and serum proteomics, we generated proteomes for undepleted serum samples derived from 199 random individuals that participated in the GS epidemiological study (Smith et al., 2013). GS is a family-based cohort of approximately 24,000 individuals in 7,000 family groups from across Scotland, aged between 18 and 98 (Smith et al., 2013). We also included a large number of commercial plasma (tebu-bio, 91 total) and serum (tebu-bio, 79 total) samples as quality controls for the sample preparation workflow, as well as repeated injections of a single sample every 11 samples (pooled from 32 prepared commercial serum samples, 39 total) as a control for the LC-MS performance. The sample preparation was done in four 96-well plates and the experiment involved 409 non-blank injections.

For interrogating the raw data, we made use of a high-quality spectral library (Bruderer et al., 2019) that was refined with DIA-NN based on the actual data (Figure 1B). Upon protein extraction and batch effect correction (STAR Methods), we assessed the robustness and consistency of protein identification achieved and herein illustrate the key quality parameters. The high-flow LC setup yielded exceptional retention time stability across the whole experiment (Figure 3A). As expected, due to the short gradients, the total proteomic depth (total number of peptides quantified) is lower than achieved with MS workflows that use pre-fractionation and longer gradients with lower flow rates and have a slower duty cycle to scan over a larger mass range. In undepleted plasma, these typically detect 250 to 450 proteins per injection (Bian et al., 2020; Bruderer et al., 2019; Geyer et al., 2016a; Liu et al., 2015). However, with an average of ~270 protein groups detected per injection and 311 in total, our much faster platform still covers most of the typical plasma proteins (Anderson and Anderson, 2002) also seen by the other methods.

Indeed, this compendium of proteins quantified includes at least 44 FDA-approved protein biomarkers (Table S5). Moreover, for large-scale experiments, the numbers of consistently quantified peptides and proteins are more relevant than the maximum number of protein groups identified, as only consistent detection allows for quantitative comparison between individuals and is suitable for the development of clinical assays. We consistently identified around 3,000 peptide precursors (i.e., peptides ionized to a specific charge; Figure 3B) and 200 unique proteins (i.e., gene products identified with specific proteotypic peptides; Figure 3C) across all 409 proteome acquisitions. In total, we detected 311 protein groups, out of which 245 uniquely identified proteins were measured with 87% data completeness and with at least five peptides. Among these, 182 unique proteins were quantified with 99% data completeness (Figure 3D).

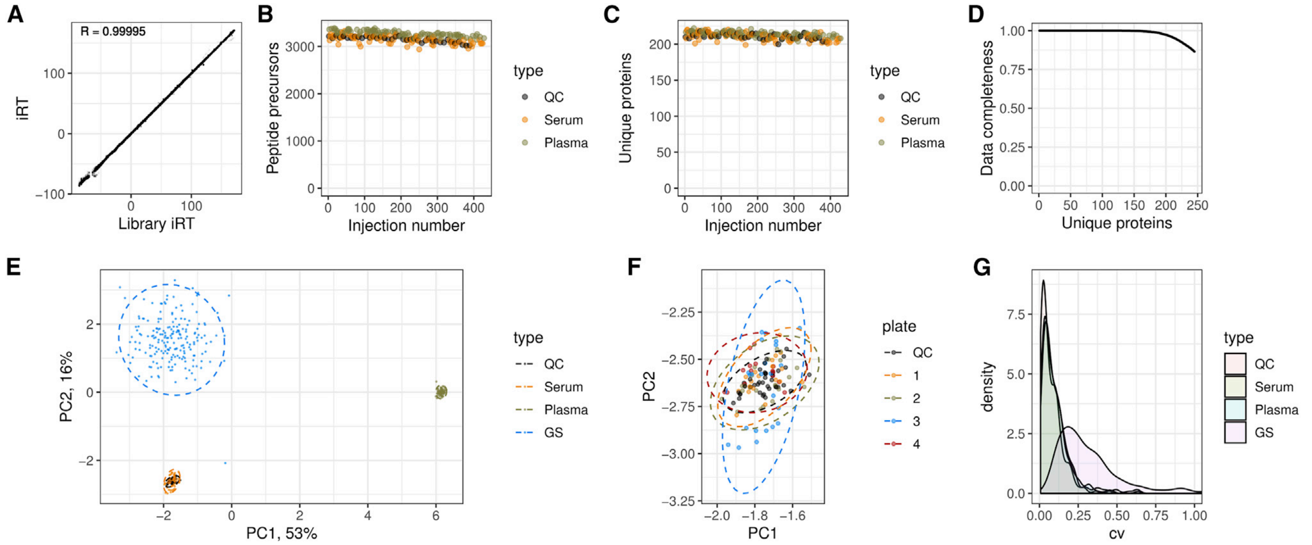

Figure 3. Robustness and Quantitative Precision of the Proteomic Platform Applied to a Population-Based Epidemiological Cohort

409 serum proteomes were analyzed for characterizing 199 participants of the GS study. The sample series are composed of 39 repeat injections (“QC”), 79 serum and 91 plasma commercial sample preparation controls, and 200 serum samples derived from the 199 participants of the GS study (“GS”).

(A) Overlaid aligned retention times (Biognosys iRT scale) of all peptide identifications in the whole experiment. Median iRT standard deviation (SD) was 0.22 (relative SD = 0.0009) and correlation between the observed iRT and library iRT was 0.99995, indicating very high retention time stability.

(B and C) (B) Numbers of peptide precursors and (C) unique proteins identified in control samples.

(D) Data completeness in the whole experiment plotted against the number of proteins identified. The data completeness for all 245 unique proteins was 87%, whereas 182 proteins were identified with data completeness 99%.

(E) PCA using consistently identified proteins (log-transformed quantities).

(F) The “serum” cluster on the PCA plot, with samples prepared on different 96-well plates colored differently. No bias between the plates can be detected.

(G) CV. After accounting for instrument drift, median CV values are 5.4% for replicate injections (“QC”), 7.6% for serum controls, 7.3% for plasma controls, and 25.6% for the participants’ samples.

To assess the quantitative precision, we first illustrated the proteomic data using principal component analysis (PCA) (Figure 3E). The PCA data fully separate in PC1 all serum from plasma samples, and in PC2, the control serum samples from the GS serum samples. The difference between the GS samples and the commercial samples might be explained by different serum collection and/or storage procedures. Moreover, the biological variability across the randomly chosen individuals is much higher than the technical variability (spread of GS samples versus serum or plasma samples) and hence is detected with high confidence by our platform. We further examined in detail the “serum” cluster of points and did not detect any bias between different sample preparation plates (Figure 3F).

Finally, we evaluated the quantification precision by calculating the CV of protein quantities across the studies. Median values obtained are 5.4% for repeat reference sample injections (“QC”) after instrument drift correction, with high abundant proteins being measured with a less than 2% CV of protein quantities (Figure S2B). Serum controls and plasma controls were measured with 7.6% and 7.3% CV, reflecting the precision of the entire workflow including sample preparation, acquisition, and data analysis. These values are much lower than the biological variation detected across the randomly chosen GS participants; when this biological variation is expressed as a CV value, it corresponds to a variation of 25.6% (Figure 3G). The platform hence confidently identifies biological variability in large-scale serum proteomic experiments of randomly chosen and presumed healthy individuals. Indeed, to our knowledge, such high precision values (<2% for high abundant proteins, 5.4% CV for all proteins [in the LC-MS part of the workflow], 7.3% for the entire workflow including sample preparation over processing 409 proteomes) have not been achieved to date in comparable large-scale proteomic studies.

Rapid and Precise High-Flow Rate Proteomics Identifies Biomarkers for COVID-19

We applied the developed workflow for the analysis of serum and citrate plasma samples for two independent COVID-19 cohorts that included patients who were among the first that were hospitalized at Charité Universitätsmedizin Berlin, between March 1st and March 26th 2020. Thirty-one SARS-CoV-2 infected patients were included in the exploratory cohort to identify biomarkers (Figure 4A; Table S1). 11/31 (35%) patients were female and 20/31 (65%) were male, median age was 54 years (range 21–81). Severity of COVID-19 was graded using the WHO ordinal outcome scale of clinical improvement (score 3 = hospitalized, no oxygen therapy; score 4 = oxygen by mask or nasal prongs; score 5 = non-invasive ventilation or high-flow oxygen; score 6 = intubation and mechanical ventilation; score 7 = ventilation and additional organ support) (World Health Organization, 2020) (Table S2). Four patients (13%) died from COVID-19, and 4 patients remain hospitalized at the time of writing. All other patients have been discharged in good health from hospital. Successively, a control group, consisting of 15 healthy volunteers

CellPress
OPEN ACCESS

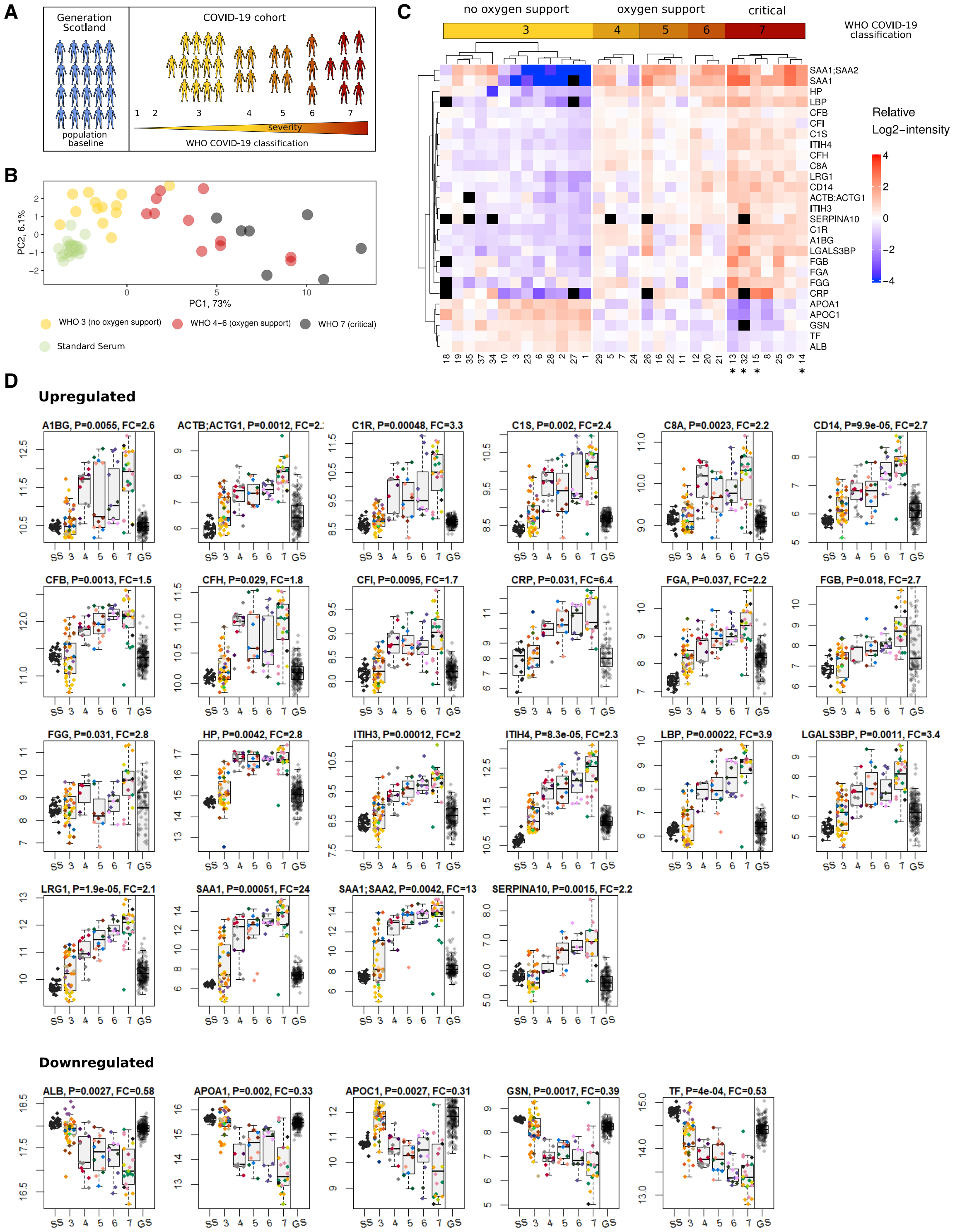

Figure 4. Protein Signatures Indicate Clinical Severity in COVID-19

(A) Study design. 199 random individuals from the GS study were measured to assess the performance of the platform and to obtain a population baseline. Protein responses based on COVID-19 severity were obtained from a cohort of 31 hospitalized SARS-CoV-2 infected patients. Severity of COVID-19 was graded using the WHO ordinal outcome scale of clinical improvement (World Health Organization, 2020).

(B) PCA based on proteins found differentially expressed depending on COVID-19 severity. Median quantities across all time points were calculated for each patient and 29 proteins without missing values were used to generate the PCA plot (quantities were standardized). Cases with the severity “3” on the WHO scale (hospitalized, no oxygen therapy) are well separated from cases with the severity “7” along the first principle component, with “4”–“6” cases in between.

(legend continued on next page)

and 17 further patients suffering from COVID-19, was recruited at the same hospital and used for validation of the biomarkers discovered (Table S1).

Because of the rapid action required in an early phase of a pandemic, we sampled depending on the first patients hospitalized (i.e., there was no other inclusion criteria than a hospitalization due to a SARS-CoV-2 infection). Such a cohort certainly differs from a long-term planned epidemiological cohort such as the GS study. With samples collected as part of the hospital routine by different medical professionals, the level of sample variability is expected to be higher. Moreover, it is difficult to assemble a control cohort that is matched for the key confounding factors, like age. Nonetheless, our proteomic platform yielded only slightly inferior values compared to the ideal-case scenario of the GS study. In the exploratory cohort, which included 104 serum samples obtained from the 31 of the earliest COVID-19 patients, we quantified 297 protein groups among which 229 unique proteins were detected with 75% data completeness. We surmise that this somewhat lower data completeness was caused by the massive changes in levels of a number of proteins upon severe SARS-CoV-2 infection as well as by the decrease in total serum protein content, also observed previously in patients hospitalized in ICU units (Nie et al., 2020). To account for this biological limitation, we applied very strict filtering to the dataset, namely, we only tested for differential abundance of proteins which had at least five different peptide precursors identified at least in one of the acquisitions.

We identified 37 protein groups with either increasing or decreasing levels, depending on the severity of the disease (0.05 significance, multiple testing corrected, Theil-Sen test against the WHO severity score; see STAR Methods for testing methodology) (Figure S7). Next, to validate the biomarkers, we processed the validation cohort (Table S1) and recorded 96 proteomes in triplicates for 15 healthy volunteers and 17 COVID-19 patients (Table S1). The experiment quantified 319 protein groups among which 248 unique proteins were detected with 85% data completeness. Despite being conducted on a different matrix (citrate plasma), this independent study confirmed 27 of the protein groups with either increasing or decreasing levels (A1BG, ACTB;ACTG1, ALB, APOA1, APOC1, C1R, C1S, C8A, CD14, CFB, CFH, CFI [complement factor I], CRP, FGA, FGB, FGG [fibrinogen alpha, beta, and gamma chains], GSN, HP, ITIH3 [inter-alpha-trypsin inhibitor heavy chain 3], ITIH4 [inter-alpha-trypsin inhibitor heavy chain 4], LBP, LGALS3BP, LRG1, SAA1 [serum amyloid A1], SAA1;SAA2 [serum amyloid A1 and A2 protein group], SERPINA10, TF; 0.05 significance, multiple testing corrected, Figure S4). This set of proteins thus represents potential biomarkers of disease severity. Out of the remaining 10 proteins, 9 (AGT [Angiotensinogen], AZGP1 [Zinc-alpha-2-glycoprotein], C2 [Complement C2], C7 [Complement component C7], C8B [Complement component C8 beta chain], CLU [Clusterin], CPN1 [Carboxypeptidase N catalytic chain], PLG [Plasminogen], and VTN [Vitronectin]) did not reach statistical significance in the smaller validation group, whereas the IGHG2;IGHG3 (Immunoglobulin Heavy Constant Gamma 2 and 3) protein group showed the opposite trend. We illustrate the quantitative variability of the validated biomarkers for COVID-19 severity on a heatmap (Figure 4C) and as boxplots (Figures 4D) as well as summarize their potential connection to COVID-19 in Table S3.

To exclude the possibility that concentration changes in these markers are due to frequent confounders, like age, we have plotted the variability of the same proteins in the GS cohort, with age spanning from 37 to 79, on samples that have been collected before the COVID-19 outbreak. We note that for identified proteins the change between mild and severe COVID-19 substantially exceeds the variation seen in the general population (Figure 4D). Moreover, plotting the protein abundance values against the age did not reveal significant correlations across the GS population baseline (Figure S8).

A PCA categorizes the individuals according to the severity of COVID-19 (Figure 4B). This shows that plasma proteomes, as measured with our platform, suit as clinical classifiers. Moreover, we illustrate the concentration changes of the biomarkers validated in the control cohort, upon grouping of the patients according the WHO severity criteria, ranging from scale 3 (hospitalized, no oxygen therapy) to the most critical (scale 7) in a heatmap (Table S1 for the grading of each patient), which graphically illustrates how level changes in these proteins reflect a progression from mild to severe COVID-19 (Figure 4C). Of note, WHO criteria consider patients up and including category 4 as “mild,” a classification that orients on the situation, that until this point, clinical care does not include invasive treatments that are difficult to provide, like intubation. However, our proteome data indicate the most substantial changes between categories 3 and 4, upon which a patient is put on oxygen supply. Our unbiased analysis hence indicates that at a molecular level, the requirement of oxygen supply coincides with a progression to severe disease.

Case studies indicate the clinical utility of the proteome signatures. First, two patients (#13 and #32) in the group of critical patients, which later died from COVID-19, clearly clustered in the heatmap and indeed had one of the most pronounced proteomic signatures (Figure 4C), implying that proteomics bears the potential to support predictions of clinical trajectories. Moreover, two case studies indicate that systematically recorded

(C) Heatmap shows protein signatures that report on COVID-19 severity. Visualization was performed using the ComplexHeatmap R package (Gu et al., 2016). Black “squares” indicate missing values. Patients labeled with an asterisk (*) had a fatal outcome of the disease.

(D) Proteins upregulated (top panel) and downregulated (lower panel) depending on COVID-19 severity (WHO grade; SS, standard serum; GS, Generation Scotland), as well as the population spread of the protein abundance in 199 randomly selected individuals of an independent cohort (Generation Scotland; GS). As the absolute quantities from the COVID-19 and GS studies cannot be compared directly (samples were obtained in a different manner), we simplified the visual assessment of the population spread, by normalizing by the median of GS quantities to the median of WHO grade 3 (no oxygen support) COVID-19 cases (the normalized values were used for illustration purposes only and not used for testing for statistical significance). The boxes show first and third quartile as well as the median (middle) and the whiskers extend to the most extreme data point, which is no more than 1.5 times the interquartile range from the box. Proteins upregulated with increasing severity of COVID-19: A1BG, ACTB;ACTG1, C1R (complement C1r), C1S (complement C1s), C8A (complement C8 alpha chain), CD14 (monocyte differentiation antigen CD14), CFB (complement factor B), CFH (complement factor H), CFI, CRP, FGA, FGB and FGG, HP, ITIH3, ITIH4, LBP, LGALS3BP, LRG1, SAA1, SAA1;SAA2, and SERPINA10; proteins downregulated with increasing severity of COVID-19: ALB, APOA1, APOC1, GSN, and TF.

CellPress
OPEN ACCESS

proteomes provide information that is beyond that of currently applied clinical assays. When we first obtained the dataset, two individuals (patients 4 and 6) initially clinically assessed as severe (Kurth et al., 2020) clustered with patients that suffered from the mild form of COVID-19 (Figure S3). These results triggered a retrospective assessment, which revealed that patient 4 turned out to be suffering from type B influenza rather than a SARS-CoV-2 infection, whereas patient 6 was classified as severe due to recent chemo-immunotherapy due to a hematological malignancy, applied just ten days before his COVID-19-related hospitalization. We have, as a consequence, excluded patient 4 from the COVID-19 dataset (all results shown in Figure 4). Both clinical case findings indicate however a high prognostic precision of the proteomic biomarker signatures and demonstrate that proteomes may very well outperform conventional clinical assays: no current clinical assays would have identified this type of outliers in the clinical assessment of the two individuals.

Proteome Signatures of Inflammation and Acute-Phase Response in Severe COVID-19

Each virus triggers its own host response, with some effectively evading the innate immune system (Kikkert, 2020). Some viral infections are hence causing only minor inflammatory responses, for example, HIV or many herpesviruses (Beachboard and Horner, 2016; Ongrádi, 2016; Sauter and Kirchhoff, 2016). Other viruses elicit dramatic inflammation and dysregulated coagulation, for example hemorrhagic fevers, e.g., Ebola virus (Baseler et al., 2017). The host immune response to SARS-CoV-2 is so far largely unknown. We detected consistent activation of both the classical complement pathway (C1R, C1S, and C8A) as well as the alternative pathway factor B (CFB) and the complement modulators: factors I (CFI) and H (CFH). Other differentially expressed proteins included the common acute-phase reactants, such as C-reactive protein (CRP) (upregulated), albumin (ALB) (downregulated), or serum amyloid proteins SAA1 and SAA2 (upregulated).

We observed upregulation of a number of proteins implicated in IL-6 signaling (Figure 4; Table S3). In addition to SAA1 and SAA2, these include inter-α-trypsin inhibitor heavy chain 4 (ITIH4), which plays an important role in extracellular matrix organization and is implicated in inflammation (Bost et al., 1998; Yang et al., 2012), HP, an acute-phase response protein (Jain et al., 2011), LRG1, a promoter of cell proliferation and angiogenesis, implicated in local inflammation and fibrosis (Honda et al., 2017), monocyte differentiation antigen CD14, primarily involved in bacterial LPS recognition (Kielian and Blecha, 1995), and the LBP, as well as LGALS3BP, a pro-inflammatory factor, which is known to induce IL-6 expression (Silverman et al., 2012). Thus, the proteomic approach surprisingly revealed a very IL-6-centered response. With the caveat that the depth of plasma proteomes is limiting, we found little evidence of involvement of other common inflammatory mediators (e.g., tumor necrosis factor (TNF), interferon (IFN) gamma, and their targets). Our study thus puts further emphasis on the importance of studying IL-6 function in relationship to new proteins and as therapeutic and diagnostic candidates.

We also observe upregulation of fibrinogen, a coagulation factor, and SERPINA10, an inhibitor of the F10a coagulation factor, further highlighting the importance of coagulation in SARS-CoV-2 infection, established by previous observations of elevated coagulation in severe COVID-19 cases (Zhou et al., 2020a).

A parallel study by (Shen et al., 2020) used a more conventional and time consuming (120× pre-fractionation consolidated in 40 fractions with TMT-16 plex) proteomics method to characterize plasma samples from 99 study participants, including 46 samples from patients with COVID-19 diagnosed in China. Despite the different cohorts and different technologies used, the proteomes implicate similar biological mechanisms in the differentiation of mild, severe, or critical disease progression. Many of the proteins that differentiate the groups in both studies belong to the complement system, acute-phase, and inflammatory response. For example, both studies agree with independently conducted clinical investigations on a number of differentially expressed proteins, in particular, ALB, the complement factors, serum amyloid proteins, ITIH3 and ITIH4 (Nie et al., 2020; Shen et al., 2020). Despite these similarities, we note important differences. For instance, we cannot confirm the downregulation of pro-platelet basic protein (PPBP) and platelet factor 4 (PF4) in severe COVID-19 as highlighted by Shen et al. We can offer several potential explanations. We note that different SARS-CoV-2 clades might exhibit different degrees of pathogenicity (Yao et al., 2020) and thus elicit different physiological responses, especially in different populations. However, when examining the response of PF4 and PPBP at the peptide level, we discovered that although several peptides maintain a relatively stable level, other PF4- and PPBP-specific peptides increase or decrease in concentration (Figure S5). This situation might indicate that instead of being differentially expressed, PF4 and PPBP might be differentially post-translationally modified.

Plasma Proteomes Provide Insights into the Virulence Mechanisms and Potential Therapeutic Targets for COVID-19

Extensive worldwide efforts have been directed recently into finding drug targets for COVID-19 treatment. As most of the damage associated with severe SARS-CoV-2 infection appears to be indirect and caused by excessive inflammation in the lungs, it is of crucial importance to seek opportunities not only to target the pathway of entry and the replication mechanism of the virus but to also identify and examine the possibilities for targeting host factors responsible for harmful inflammatory responses to both alleviate the severity of the infection and to reduce the chance of long-lasting complications (Huang et al., 2020). Some preliminary results in that direction appear promising. For example, pro-inflammatory signaling via IL-6 has been determined to be a marker of severe COVID-19 (Chen et al., 2020; Ruan et al., 2020) and preliminary results on the inhibition of IL-6 receptor (IL-6R) with tocilizumab seem to indicate clinical improvement (Coomes and Haghbayan, 2020). Here, we discovered several proteins that are differentially expressed with the severity of COVID-19 that are linked to IL-6-mediated proinflammatory cytokine signaling: (1) the CD14-LBP LPS recognition system, (2) upregulation of LRG1, an angiogenesis and anti-apoptotic factor associated with inflammation, and (3) upregulation of the

LGALS3BP, an inducer of IL-6. Below, we discuss the potential significance of these findings.

Coronaviruses are known to actively disrupt the host immune response (Enjuanes et al., 2016). For example, their papain-like proteases (PLPs) act as interferon antagonists (Niemeyer et al., 2018), causing delayed type-I interferon response, macrophage-mediated inflammation, and lung damage (Channappanavar et al., 2016). Here, we observed upregulation of both monocyte differentiation antigen CD14 (~2.7×) and LBP (~3.9×) in severe COVID-19. As the response to bacterial LPS is one of the primary functions of both CD14 and LBP, which can act in complex to sensitize toll-like receptor-mediated LPS recognition (Ranoa et al., 2013), this observation reflects the dysregulation of the innate immune response by SARS-CoV-2, leading to the activation of the anti-bacterial defense and sensitization to LPS, thus contributing to excessive inflammation, with effects likely more pronounced in case of a concomitant secondary bacterial infection. Interestingly, CD14 and LBP upregulation has been observed in viral pneumonia before (Van Gucht et al., 2005), while CD14 is one of the primary mediators of lung inflammation (Anas et al., 2010). Of note, the glycosylphosphatidylinositol (GPI)-anchored form of CD14 is primarily displayed by monocytes and macrophages (Marcos et al., 2010), while the proportion of $CD14^{+}CD16^{+}$ inflammatory monocytes in the peripheral blood increases along with COVID-19 severity (Zhou et al., 2020b). At the same time, IL-6 induces soluble CD14 production in the liver (Bas et al., 2004) as well as, along with other cytokines, release of CD14 from monocytes upon their activation (Shive et al., 2015). Given that CD14 is a potent activator of pro-inflammatory cytokine signaling (Zanoni and Granucci, 2013), it might present a potential therapeutic target for COVID-19.

LRG1 is another pro-inflammatory factor induced by IL-6 (Shirai et al., 2009), which is known to promote angiogenesis and cell proliferation, while inhibiting apoptosis (Meng et al., 2016; Naka and Fujimoto, 2018; Wang et al., 2013). Some recent findings indicate its role in promoting skin fibrosis and lung fibrosis in transforming growth factor beta (TGF-β)-mediated fashion (Gao et al., 2019; Honda et al., 2017). Given the previously reported association of the Middle East respiratory syndrome (MERS) infection with lung fibrosis (Zhao et al., 2008) and emerging reports (British Thoracic Society, 2020) of lung fibrosis in a substantial proportion of COVID-19 survivors, we hypothesize that the ~2.1× elevation of serum LRG1 levels we observe in critical COVID-19 cases in comparison with the mild cases might indicate the increased risk of fibrosis, highlighting LRG1 as yet another potential therapeutic candidate for COVID-19 treatment. Furthermore, we detected about ~3.4× upregulation of LGALS3BP, which is known to induce the expression of IL-6 by stromal cells in galectin-3-dependent manner (Silverman et al., 2012). Of note, Galectin-3 has long been considered an attractive drug target in combating various forms of TGF-β-mediated fibrosis and pathological inflammatory conditions (Brinchmann et al., 2018; Mackinnon et al., 2012; Shen et al., 2018; Yu et al., 2013). Inhibition of galectin-3-mediated signaling pathways hence represents another potential therapeutic target against COVID-19.

Tissue Injury and Dysregulation of Modulators of Inflammation

We report substantially decreased (~2.6×) levels of GSN (Figures 4C and 4D). Plasma GSN is a part of the extracellular actin scavenger system (EASS), which removes toxic F-actin filaments that have been released from necrotic cells to the bloodstream (Piktel et al., 2018). Low levels of plasma GSN are associated with inflammation: it is believed that GSN is recruited to the sites of tissue injury to handle the released actin, depleting its plasma levels. Interestingly, we do observe the increase in serum actin concentration (beta and gamma-1 actin, ~2×), indicative of tissue injury (DiNubile, 2008), which could explain the GSN depletion from the blood. Importantly, plasma GSN is a powerful modulator of inflammation, which carries a protective function (DiNubile, 2008; Li et al., 2012). Low plasma GSN is a marker of poor prognosis in various pathological conditions, including diabetes (Khatri et al., 2014), cancers (Asare-Werehene et al., 2019; Stock et al., 2015), and sepsis (Lee et al., 2007), leading to suggestions and animal tests for its therapeutic use. Going forward, it will be important to assess GSN levels in at-risk populations for severe COVID-19, e.g., patients with diabetes. Of note, treatment with GSN has been observed to decrease IL-6 levels in mice (Cheng et al., 2017) and has been suggested to promote epithelial repair (Wittmann et al., 2018). The development of therapies to stabilize the GSN levels could hence be of direct therapeutic value for treating COVID-19.

Notably, we observed a decrease in the expression levels of APOA1 (APOA1; ~3×). APOA1 is a major component of the high-density lipoprotein (HDL) complex, which is a modulator of innate immune response and inflammation (Fotakis et al., 2019; Gordon et al., 2011; Macpherson et al., 2019; White et al., 2017). We also observe decreased levels of APOC1 (~3.2×), a component of several lipoprotein complexes (Fuior and Gafencu, 2019). Based on the GS study, we note that the serum levels of APOA1 are correlated with those of HDL cholesterol (Figure S6). Although decreased APOA1 levels have been observed in systemic inflammatory response (Kumaraswamy et al., 2012; Sirniö et al., 2017), including in COVID-19 (Nie et al., 2020), a potential explanation of the downregulation we observe here would also be provided, if naturally lower APOA1 and hence a different metabolic condition of the individual were associated with a higher risk of severe SARS-CoV-2 disease progression.

In conclusion, SARS-CoV-2, SARS, and MERS constitute a class of emerging coronaviruses of high public health concern. It is likely that other viruses will emerge in the future for which at time of outbreak insufficient biochemical knowledge will be available to identify biomarkers and to define point-of-care clinical classifiers. Serum and plasma proteomics can present valuable and unbiased information about disease progression and therapeutic candidates, without prior knowledge about the etiologies and biomolecules involved. We present a workflow for rapid and large-scale clinical proteomics that is re-designed in comparison to previous platforms. The sample preparation workflow scales to high sample numbers, enables high quantification precision, and reduces batch effects for large-scale and longitudinal studies, while the data acquisition and processing workflow is able to exploit the advantages of high-flow chromatography in short-gradient proteomics. Our platform improves

throughput, data quality, and greatly simplifies implementation in regulated laboratories, as it builds on ISO13485 standardization as a reference. We demonstrate a quantification precision and acquisition robustness that, to our knowledge, has not previously been shown in large-scale proteomic experiments. We then applied the technology to a cohort of early hospitalized cases of the SARS-CoV-2 pandemic. We identify a series of proteins that are differentially expressed depending on the severity of COVID-19 and demonstrate the potential of proteome signatures to act as clinical classifiers. The proteome signatures capture the host response to COVID-19 infection, highlighting the role of complement factors, the coagulation system, and indicate a high specificity of several inflammation modulators as well as pro-inflammatory signaling both upstream and downstream of IL-6. The proteomic signatures and biomarkers identified pave the way for the development of routine assays to support clinical decision making, as well as provide hypotheses about potential COVID-19 therapeutic targets.

STAR★METHODS

Detailed methods are provided in the online version of this paper and include the following:

- KEY RESOURCES TABLE
- RESOURCE AVAILABILITY
  - Lead Contact
  - Materials Availability
  - Data and Code Availability
- EXPERIMENTAL MODEL AND SUBJECT DETAILS
  - Clinical Samples of COVID-19 Patients
  - Generation Scotland Study
- METHOD DETAILS
  - Plasma and Serum Sample Preparation
  - Liquid Chromatography-Mass Spectrometry Setup
- QUANTIFICATION AND STATISTICAL ANALYSIS
  - Mass Spectrometry Data Processing, Batch Correction and Quality Control
  - Differential Expression Analysis
  - Chromatographic Peaks Full Width at Half Maximum (FWHM) Estimations
  - Total Ion and Extracted Ion Chromatograms

SUPPLEMENTAL INFORMATION

Supplemental Information can be found online at https://doi.org/10.1016/j.cels.2020.05.012.

ACKNOWLEDGMENTS

We thank Trefor Mitchell for his help with the quality management system; Stephan Kamrad and Simran Aulakh for proofreading the manuscript; Jan-David Manntz (Beckman, Germany) for help with the Biomek i7; and Robert Lane, Jean-Baptiste Vincedent, and Nick Morrice (SCIEX) for help with the TripleTOF 6600. This work was supported by the Ministry of Education and Research (BMBF), as part of the National Research Node "Mass spectrometry in Systems Medicine" (MSCoresys), under grant agreement 031L0220A. The study was further supported by the Francis Crick Institute, which receives its core funding from Cancer Research UK (FC001134), the UK Medical Research Council (FC001134), and the Wellcome Trust (FC001134) and received specific funding from the BBSRC (BB/ N015215/1 and BB/N015282/1) and the Wellcome Trust (200829/Z/16/Z) as well as a Crick Idea to Innovation (i2i) initiative (grant number: 10658) as well as the Crick lifeArc Project 1290305 (to M.R.). The Generation Scotland study received core support from the Chief Scientist Office of the Scottish Government Health Directorates (CZD/16/6) and the Scottish Funding Council (HR03006) and is now supported by the Wellcome Trust (216767/Z/19/Z). A. C. is funded by HDR UK and the Wellcome Trust (216767/Z/19/Z). C. H. is supported by an MRC University Unit Programme grant (MC_UU_00007/10) (QTL in Health and Disease). R.E.M. is supported by an Alzheimer's Research UK project grant (ARUK-PG2017B-10). L.E.S. is supported by the German Research Foundation (DFG, SFB-TR84 114933180) and by the Berlin Institute of Health (BIH), which receives funding from the Ministry of Education and Research (BMBF). M.Witzenrath is supported by grants from the German Research Foundation, SFB-TR84 C6 and C9 and by the German Ministry of Education and Research (BMBF) in the framework of the CAPSyS (01ZX1304B) SYMPATH (FKZ 01ZX1906A) and PROVID project (FKZ 01KI20160A)

AUTHOR CONTRIBUTIONS

M.R., L.E.S., F.K., M. Witzenrath, N.S., C.D., M.M., W.M.K., K.S.L., C.L., R.E.M., D.J.P., C.H., A.C., and C.v.K. designed and supervised the study; L.E.S., F.K., M. Witzenrath, C.v.K., A.H., N.S., S.H., M.P., L.M., and D.W. conducted clinical assessments and supervised clinical sample handling; C.B.M., V.D., A.Z., and M.R. designed and developed the proteomics high-throughput platform as well as the proteomic quality control procedures; A.F., M.M., M. White, C.B.M., K.T.-T., S.I.V., A.-S.E., M.K., D.L.,C.K., and F.A. carried out sample preparation and measurements; V.D. and A.Z. designed and developed data processing and analysis workflow and analyzed the proteomic data; M.R., L.E.S, F.K., V.D., C.B.M., and S.V. wrote the paper with contributions from all authors.

DECLARATION OF INTERESTS

The authors declare no competing interests.

Received: April 26, 2020
Revised: May 22, 2020
Accepted: May 27, 2020
Published: June 2, 2020

REFERENCES

Ahadi, S., Zhou, W., Schüssler-Fiorenza Rose, S.M., Sailani, M.R., Contrepois, K., Avina, M., Ashland, M., Brunet, A., and Snyder, M. (2020). Personal aging markers and ageotypes revealed by deep longitudinal profiling. Nat. Med. *26*, 83–90.

Anas, A., van der Poll, T., and de Vos, A.F. (2010). Role of CD14 in lung inflammation and infection. Crit. Care *14*, 209.

Anderson, N.L., and Anderson, N.G. (2002). The human plasma proteome: history, character, and diagnostic prospects. Mol. Cell. Proteomics *1*, 845–867.

Asare-Werehene, M., Communal, L., Carmona, E., Le, T., Provencher, D., Mes-Masson, A.M., and Tsang, B.K. (2019). Pre-operative circulating plasma gelsolin predicts residual disease and detects early stage ovarian cancer. Sci. Rep. *9*, 13924.

Bache, N., Geyer, P.E., Bekker-Jensen, D.B., Hoerning, O., Falkenby, L., Treit, P.V., Doll, S., Paron, I., Müller, J.B., Meier, F., et al. (2018). A novel LC system embeds analytes in pre-formed gradients for rapid, ultra-robust proteomics. Mol. Cell. Proteomics *17*, 2284–2296.

Bas, S., Gauthier, B.R., Spenato, U., Stingelin, S., and Gabay, C. (2004). CD14 is an acute-phase protein. J. Immunol. *172*, 4470–4479.

Baseler, L., Chertow, D.S., Johnson, K.M., Feldmann, H., and Morens, D.M. (2017). The pathogenesis of Ebola virus disease. Annu. Rev. Pathol. *12*, 387–418.

Beachboard, D.C., and Horner, S.M. (2016). Innate immune evasion strategies of DNA and RNA viruses. Curr. Opin. Microbiol. *32*, 113–119.

Bekker-Jensen, D.B., Martínez-Val, A., Steigerwald, S., Rüther, P., Fort, K.L., Arrey, T.N., Harder, A., Makarov, A., and Olsen, J.V. (2020). A compact

quadrupole-Orbitrap mass spectrometer with FAIMS interface improves proteome coverage in short LC gradients. Mol. Cell. Proteomics *19*, 716–729.

Bennike, T.B., Bellin, M.D., Xuan, Y., Stensballe, A., et al. (2018). Journal of proteome research *17*, 1983–1992.

Bian, Y., Zheng, R., Bayer, F.P., Wong, C., Chang, Y.C., Meng, C., Zolg, D.P., Reinecke, M., Zecha, J., Wiechmann, S., et al. (2020). Robust, reproducible and quantitative analysis of thousands of proteomes by micro-flow LC–MS/MS. Nat. Commun. *11*, 157.

Bluemlein, K., and Ralser, M. (2011). Monitoring protein expression in whole-cell extracts by targeted label- and standard-free LC-MS/MS. Nat. Protoc. *6*, 859–869.

Bost, F., Diarra-Mehrpour, M., and Martin, J.P. (1998). Inter-alpha-trypsin inhibitor proteoglycan family– a group of proteins binding and stabilizing the extracellular matrix. Eur. J. Biochem. *252*, 339–346.

Brinchmann, M.F., Patel, D.M., and Iversen, M.H. (2018). The role of galectins as modulators of metabolism and inflammation. Mediators Inflamm *2018*, 9186940.

British Thoracic Society (2020). British thoracic society advice for managing interstitial lung disease patients during COVID-19 pandemic. https://www.brit-thoracic.org.uk/media/455101/bts-management-advice-for-ild-patients-v10-23-march-2020.pdf.

Bruderer, R., Muntel, J., Müller, S., Bernhardt, O.M., Gandhi, T., Cominetti, O., Macron, C., Carayol, J., Rinner, O., Astrup, A., et al. (2019). Analysis of 1508 plasma samples by capillary-flow data-independent acquisition profiles proteomics of weight loss and maintenance. Mol. Cell. Proteomics *18*, 1242–1254.

Bussey, K.A., and Brinkmann, M.M. (2018). Strategies for immune evasion by human tumor viruses. Curr. Opin. Virol. *32*, 30–39.

Channappanavar, R., Fehr, A.R., Vijay, R., Mack, M., Zhao, J., Meyerholz, D.K., and Perlman, S. (2016). Dysregulated type I interferon and inflammatory monocyte-macrophage responses cause lethal pneumonia in SARS-CoV-infected mice. Cell Host Microbe *19*, 181–193.

Chen, N., Zhou, M., Dong, X., Qu, J., Gong, F., Han, Y., Qiu, Y., Wang, J., Liu, Y., Wei, Y., et al. (2020). Epidemiological and clinical characteristics of 99 cases of 2019 novel coronavirus pneumonia in Wuhan, China: a descriptive study. Lancet *395*, 507–513.

Cheng, Y., Hu, X., Liu, C., Chen, M., Wang, J., Wang, M., Gao, F., Han, J., Zhang, C., Sun, D., and Min, R. (2017). Gelsolin inhibits the inflammatory process induced by LPS. Cell. Physiol. Biochem. *41*, 205–212.

Coomes, E.A., and Haghbayan, H. (2020). Interleukin-6 in COVID-19: a systematic review and meta-analysis. medRxiv. https://doi.org/10.1101/2020.03.30.20048058.

Corman, V.M., Landt, O., Kaiser, M., Molenkamp, R., Meijer, A., Chu, D.K., Bleicker, T., Brünink, S., Schneider, J., Schmidt, M.L., et al. (2020). Detection of 2019 novel coronavirus (2019-nCoV) by real-time RT-PCR. EURO Surveill. *25*, 2000045.

Cox, J., Hein, M.Y., Luber, C.A., Paron, I., Nagaraj, N., and Mann, M. (2014). Accurate proteome-wide label-free quantification by delayed normalization and maximal peptide ratio extraction, termed MaxLFQ. Mol. Cell. Proteomics *13*, 2513–2526.

Crutchfield, C.A., Thomas, S.N., Sokoll, L.J., and Chan, D.W. (2016). Advances in mass spectrometry-based clinical biomarker discovery. Clin. Proteomics *13*, 1.

Demichev, V., Messner, C.B., Vernardis, S.I., Lilley, K.S., and Ralser, M. (2020). DIA-NN: neural networks and interference correction enable deep proteome coverage in high throughput. Nat. Methods *17*, 41–44.

DiNubile, M.J. (2008). Plasma gelsolin as a biomarker of inflammation. Arthritis Res. Ther. *10*, 124.

Enjuanes, L., Zuñiga, S., Castaño-Rodriguez, C., Gutierrez-Alvarez, J., Canton, J., and Sola, I. (2016). Molecular basis of coronavirus virulence and vaccine development. Coronaviruses. Adv Virus Res. *96*, 245–286.

Fotakis, P., Kothari, V., Thomas, D.G., Westerterp, M., Molusky, M.M., Altin, E., Abramowicz, S., Wang, N., He, Y., Heinecke, J.W., et al. (2019). Anti-inflammatory effects of HDL (high-density lipoprotein) in macrophages predominate over proinflammatory effects in atherosclerotic plaques. Arterioscler. Thromb. Vasc. Biol. *39*, e253–e272.

Fu, Q., Kowalski, M.P., Mastali, M., Parker, S.J., Sobhani, K., van den Broek, I., Hunter, C.L., and Van Eyk, J.E. (2018). Highly reproducible automated proteomics sample preparation workflow for quantitative mass spectrometry. J. Proteome Res. *17*, 420–428.

Fuior, E.V., and Gafencu, A.V. (2019). Apolipoprotein C1: its pleiotropic effects in lipid metabolism and beyond. Int. J. Mol. Sci. *20*, 5939.

Gama, M.R., Collins, C.H., and Bottoli, C.B.G. (2013). Nano-liquid chromatography in pharmaceutical and biomedical research. J. Chromatogr. Sci. *51*, 694–703.

Gao, Y., Zhou, J., Xie, Z., Wang, J., Ho, C.K., Zhang, Y., and Li, Q. (2019). Mechanical strain promotes skin fibrosis through LRG-1 induction mediated by ELK1 and ERK signalling. Commun. Biol. *2*, 359.

Geyer, P.E., Holdt, L.M., Teupser, D., and Mann, M. (2017). Revisiting biomarker discovery by plasma proteomics. Mol. Syst. Biol. *13*, 942.

Geyer, P.E., Kulak, N.A., Pichler, G., Holdt, L.M., Teupser, D., and Mann, M. (2016a). Plasma proteome profiling to assess human health and disease. Cell Syst *2*, 185–195.

Geyer, P.E., Voytik, E., Treit, P.V., Doll, S., Kleinhempel, A., Niu, L., Müller, J.B., Buchholtz, M.L., Bader, J.M., Teupser, D., et al. (2019). Plasma Proteome Profiling to detect and avoid sample-related biases in biomarker studies. EMBO Mol. Med. *11*, e10427.

Geyer, P.E., Wewer Albrechtsen, N.J.W., Tyanova, S., Grassl, N., Iepsen, E.W., Lundgren, J., Madsbad, S., Holst, J.J., Torekov, S.S., and Mann, M. (2016b). Proteomics reveals the effects of sustained weight loss on the human plasma proteome. Mol. Syst. Biol. *12*, 901.

Gillet, L.C., Navarro, P., Tate, S., Röst, H., Selevsek, N., Reiter, L., Bonner, R., and Aebersold, R. (2012). Targeted data extraction of the MS/MS spectra generated by data-independent acquisition: a new concept for consistent and accurate proteome analysis. Mol. Cell. Proteomics *11*, O111.016717.

Gordon, S.M., Hofmann, S., Askew, D.S., and Davidson, W.S. (2011). High density lipoprotein: it's not just about lipid transport anymore. Trends Endocrinol. Metab. *22*, 9–15.

Grebe, S.K.G., and Singh, R.J. (2011). LC-MS/MS in the clinical laboratory–where to from here? Clin. Biochem. Rev. *32*, 5–31.

Gu, Z., Eils, R., and Schlesner, M. (2016). Complex heatmaps reveal patterns and correlations in multidimensional genomic data. Bioinformatics *32*, 2847–2849.

He, T. (2019). PROTEOMICS- Clinical Applications *13*, 1800198.

Honda, H., Fujimoto, M., Serada, S., Urushima, H., Mishima, T., Lee, H., Ohkawara, T., Kohno, N., Hattori, N., Yokoyama, A., and Naka, T. (2017). Leucine-rich α-2 glycoprotein promotes lung fibrosis by modulating TGF-β signaling in fibroblasts. Physiol. Rep. *5*, e13556.

Huang, C., Wang, Y., Li, X., Ren, L., Zhao, J., Hu, Y., Zhang, L., Fan, G., Xu, J., Gu, X., et al. (2020). Clinical features of patients infected with 2019 novel coronavirus in Wuhan, China. Lancet *395*, 497–506.

Jain, S., Gautam, V., and Naseem, S. (2011). Acute-phase proteins: as diagnostic tool. J. Pharm. Bioallied Sci. *3*, 118–127.

Khatri, N., Sagar, A., Peddada, N., Choudhary, V., Chopra, B.S., Garg, V., Garg, R., and Ashish. (2014). Plasma gelsolin levels decrease in diabetic state and increase upon treatment with F-actin depolymerizing versions of gelsolin. J. Diabetes Res. *2014*, 152075.

Kielian, T.L., and Blecha, F. (1995). CD14 and other recognition molecules for lipopolysaccharide: a review. Immunopharmacology *29*, 187–205.

Kikkert, M. (2020). Innate immune evasion by human respiratory RNA viruses. J. Innate Immun. *12*, 4–20.

Kumaraswamy, S.B., Linder, A., Åkesson, P., and Dahlbäck, B. (2012). Decreased plasma concentrations of apolipoprotein M in sepsis and systemic inflammatory response syndromes. Crit. Care *16*, R60.

Kurth, F., Roennefarth, M., Thibeault, C., Corman, V.M., Mueller-Redezky, H., Mittermaier, M., Ruwwe-Glosenkamp, C., Heim, K.M., Krannich, A., Zvorc, S., et al. (2020). Infection.

Lee, P.S., Waxman, A.B., Cotich, K.L., Chung, S.W., Perrella, M.A., and Stossel, T.P. (2007). Plasma gelsolin is a marker and therapeutic agent in animal sepsis. Crit. Care Med. *35*, 849–855.

Li, G.H., Arora, P.D., Chen, Y., McCulloch, C.A., and Liu, P. (2012). Multifunctional roles of gelsolin in health and diseases. Med. Res. Rev. *32*, 999–1025.

Liu, Y., Buil, A., Collins, B.C., Gillet, L.C.J., Blum, L.C., Cheng, L.Y., Vitek, O., Mouritsen, J., Lachance, G., Spector, T.D., et al. (2015). Quantitative variability of 342 plasma proteins in a human twin population. Mol. Syst. Biol. *11*, 786.

Lowenthal, M.S., Liang, Y., Phinney, K.W., and Stein, S.E. (2014). Quantitative bottom-up proteomics depends on digestion conditions. Anal. Chem. *86*, 551–558.

Ludwig, C., Gillet, L., Rosenberger, G., Amon, S., Collins, B.C., and Aebersold, R. (2018). Data-independent acquisition-based SWATH-MS for quantitative proteomics: a tutorial. Mol. Syst. Biol. *14*, e8126.

Mackinnon, A.C., Gibbons, M.A., Farnworth, S.L., Leffler, H., Nilsson, U.J., Delaine, T., Simpson, A.J., Forbes, S.J., Hirani, N., Gauldie, J., and Sethi, T. (2012). Regulation of transforming growth factor-β1-driven lung fibrosis by galectin-3. Am. J. Respir. Crit. Care Med. *185*, 537–546.

Macpherson, M.E., Halvorsen, B., Yndestad, A., Ueland, T., Mollnes, T.E., Berge, R.K., Rashidi, A., Otterdal, K., Gregersen, I., Kong, X.Y., et al. (2019). Impaired HDL function amplifies systemic inflammation in common variable immunodeficiency. Sci. Rep. *9*, 9427.

Marcos, V., Latzin, P., Hector, A., Sonanini, S., Hoffmann, F., Lacher, M., Koller, B., Bufler, P., Nicolai, T., Hartl, D., and Griese, M. (2010). Expression, regulation and clinical significance of soluble and membrane CD14 receptors in pediatric inflammatory lung diseases. Respir. Res. *11*, 32.

Meng, H., Song, Y., Zhu, J., Liu, Q., Lu, P., Ye, N., Zhang, Z., Pang, Y., Qi, J., and Wu, H. (2016). LRG1 promotes angiogenesis through upregulating the TGF-β1 pathway in ischemic rat brain. Mol. Med. Rep. *14*, 5535–5543.

Messner, C., Demichev, V., Bloomfield, N., Ivosev, G., Wasim, F., Zelezniak, A., Lilley, K., Tate, S., and Ralser, M. (2019). ScanningSWATH enables ultra-fast proteomics using high-flow chromatography and minute-scale gradients. bioRxiv. https://doi.org/10.1101/656793.

Millard, S.P. (2014). EnvStats, an RPackage for environmental statistics. In Wiley StatsRef: Statistics Reference Online, N. Balakrishnan, T. Colton, B. Everitt, W. Piegorsch, F. Ruggeri, and J.L. Teugels, eds. (Wiley). https://doi.org/10.1002/9781118445112.stat07181.

Mischak, H., Ioannidis, J.P.A., Argilis, A., Attwood, T.K., et al. (2012). European Journal of Clinical Investigation *42*, 1027–1036.

Müller, T., Kalxdorf, M., Longuespée, R., Kazdal, D.N., Stenzinger, A., and Krijgsveld, J. (2020). Automated sample preparation with SP3 for low-input clinical proteomics. Mol. Syst. Biol. *16*, e9111.

Nair, H., and Clarke, W. (2016). Mass spectrometry for the Clinical Laboratory (Academic Press).

Naka, T., and Fujimoto, M. (2018). LRG is a novel inflammatory marker clinically useful for the evaluation of disease activity in rheumatoid arthritis and inflammatory bowel disease. Immunol. Med. *41*, 62–67.

Nie, S., Zhao, X., Zhao, K., Zhang, Z., Zhang, Z., and Zhang, Z. (2020). Metabolic disturbances and inflammatory dysfunction predict severity of coronavirus disease 2019 (COVID-19): a retrospective study. medRxiv. https://doi.org/10.1101/2020.03.24.20042283.

Niemeyer, D., Mösbauer, K., Klein, E.M., Sieberg, A., Mettelman, R.C., Mielech, A.M., Dijkman, R., Baker, S.C., Drosten, C., and Müller, M.A. (2018). The papain-like protease determines a virulence trait that varies among members of the SARS-coronavirus species. PLoS Pathog. *14*, e1007296.

Nilsson, T., Mann, M., Aebersold, R., Yates, J.R., 3rd, Bairoch, A., and Bergeron, J.J.M. (2010). Mass spectrometry in high-throughput proteomics: ready for the big time. Nat. Methods *7*, 681–685.

Niu, L., Geyer, P.E., Wewer Albrechtsen, N.J.W., Gluud, L.L., Santos, A., Doll, S., Treit, P.V., Holst, J.J., Knop, F.K., Vilsbøll, T., et al. (2019). Plasma proteome profiling discovers novel proteins associated with non-alcoholic fatty liver disease. Mol. Syst. Biol. *15*, e8793.

Ongrádi, J. (2016). Herpesviridae (InTechOpen).

Perez-Riverol, Y., Csordas, A., Bai, J., Bernal-Llinares, M., Hewapathirana, S., Kundu, D.J., Inuganti, A., Griss, J., Mayer, G., Eisenacher, M., et al. (2019). The PRIDE database and related tools and resources in 2019: improving support for quantification data. Nucleic Acids Res. *47*, D442–D450.

Petherick, A. (2020). Developing antibody tests for SARS-CoV-2. Lancet *395*, 1101–1102.

Pham, T.V., Henneman, A.A., and Jimenez, C.R. (2020). iq: an R package to estimate relative protein abundances from ion quantification in DIA-MS-based proteomics. Bioinformatics *36*, 2611–2613.

Piehowski, P.D., Petyuk, V.A., Orton, D.J., Xie, F., Moore, R.J., Ramirez-Restrepo, M., Engel, A., Lieberman, A.P., Albin, R.L., Camp, D.G., et al. (2013). Sources of technical variability in quantitative LC–MS proteomics: human brain tissue sample analysis. J. Proteome Res. *12*, 2128–2137.

Piktel, E., Levental, I., Durnaś, B., Janmey, P.A., and Bucki, R. (2018). Plasma gelsolin: indicator of inflammation and its potential as a diagnostic tool and therapeutic target. Int. J. Mol. Sci. *19*, 2516.

Ranoa, D.R.E., Kelley, S.L., and Tapping, R.I. (2013). Human lipopolysaccharide-binding protein (LBP) and CD14 independently deliver triacylated lipoproteins to toll-like receptor 1 (TLR1) and TLR2 and enhance formation of the ternary signaling complex. J. Biol. Chem. *288*, 9729–9741.

Ruan, Q., Yang, K., Wang, W., Jiang, L., and Song, J. (2020). Correction to: clinical predictors of mortality due to COVID-19 based on an analysis of data of 150 patients from Wuhan, China. Intensive Care Med. 1–3, https://doi.org/10.1007/s00134-020-06028-z.

Sauter, D., and Kirchhoff, F. (2016). HIV replication: a game of hide and sense. Curr. Opin. HIV AIDS *11*, 173–181.

Schilling, B., Gibson, B.W., and Hunter, C.L. (2017). Generation of high-quality SWATH® acquisition data for label-free quantitative proteomics studies using TripleTOF® mass spectrometers. Methods Mol. Biol. *1550*, 223–233.

Shen, H., Wang, J., Min, J., Xi, W., Gao, Y., Yin, L., Yu, Y., Liu, K., Xiao, J., Zhang, Y.F., and Wang, Z.-N. (2018). Activation of TGF-β1/α-SMA/Col I Profibrotic pathway in fibroblasts by galectin-3 contributes to atrial fibrosis in experimental models and patients. Cell. Physiol. Biochem. *47*, 851–863.

Shen, B., Yi, X., Sun, Y., Bi, X., Du, J., Zhang, C., Quan, S., Zhang, F., Sun, R., Qian, L., et al. (2020). Proteomic and metabolomic characterization of COVID-19 patient sera. Cell *182*, https://doi.org/10.1016/j.cell.2020.05.032.

Shirai, R., Hirano, F., Ohkura, N., Ikeda, K., and Inoue, S. (2009). Up-regulation of the expression of leucine-rich alpha(2)-glycoprotein in hepatocytes by the mediators of acute-phase response. Biochem. Biophys. Res. Commun. *382*, 776–779.

Shishkova, E., Hebert, A.S., and Coon, J.J. (2016). Now, more than ever, proteomics needs better chromatography. Cell Syst. *3*, 321–324.

Shive, C.L., Jiang, W., Anthony, D.D., and Lederman, M.M. (2015). Soluble CD14 is a nonspecific marker of monocyte activation. AIDS *29*, 1263–1265.

Silverman, A.M., Nakata, R., Shimada, H., Sposto, R., and DeClerck, Y.A. (2012). A galectin-3–dependent pathway upregulates interleukin-6 in the microenvironment of human neuroblastoma. Cancer Res. *72*, 2228–2238.

Sirniö, P., Väyrynen, J.P., Klintrup, K., Mäkelä, J., Mäkinen, M.J., Karttunen, T.J., and Tuomisto, A. (2017). Decreased serum apolipoprotein A1 levels are associated with poor survival and systemic inflammatory response in colorectal cancer. Sci. Rep. *7*, 5374.

Smith, B.H., Campbell, A., Linksted, P., Fitzpatrick, B., Jackson, C., Kerr, S.M., Deary, I.J., MacIntyre, D.J., Campbell, H., McGilchrist, M., et al. (2013). Cohort profile: generation Scotland: Scottish family health study (GS:SFHS). The study, its participants and their potential for genetic research on health and illness. Int. J. Epidemiol. *42*, 689–700.

Stock, A.M., Klee, F., Edlund, K., Grinberg, M., Hammad, S., Marchan, R., Cadenas, C., Niggemann, B., Zänker, K.S., Rahnenführer, J., et al. (2015). Gelsolin is associated with longer metastasis-free survival and reduced cell migration in estrogen receptor-positive breast cancer. Anticancer Res. *35*, 5277–5285.

UniProt Consortium (2019). UniProt: a worldwide hub of protein knowledge. Nucleic Acids Res. *47*, D506–D515.

Van Gucht, S., Van Reeth, K., Nauwynck, H., and Pensaert, M. (2005). Porcine reproductive and respiratory syndrome virus infection increases CD14 expression and lipopolysaccharide-binding protein in the lungs of pigs. Viral Immunol. *18*, 116–126.

Vowinckel, J., Zelezniak, A., Bruderer, R., Mülleder, M., Reiter, L., and Ralser, M. (2018). Cost-effective generation of precise label-free quantitative proteomes in high-throughput by microLC and data-independent acquisition. Sci. Rep. *8*, 4346.

Wang, X., Abraham, S., McKenzie, J.A.G., Jeffs, N., Swire, M., Tripathi, V.B., Luhmann, U.F.O., Lange, C.A.K., Zhai, Z., Arthur, H.M., et al. (2013). LRG1 promotes angiogenesis by modulating endothelial TGF-β signalling. Nature *499*, 306–311.

Wewer Albrechtsen, N.J., Geyer, P.E., Doll, S., Treit, P.V., Bojsen-Møller, K.N., Martinussen, C., Jørgensen, N.B., Torekov, S.S., Meier, F., Niu, L., et al. (2018). Plasma proteome profiling reveals dynamics of inflammatory and lipid homeostasis markers after Roux-en-Y gastric bypass surgery. Cell Syst *7*, 601–612.e3.

White, R., Giordano, S., and Datta, G. (2017). Role of HDL-associated proteins and lipids in the regulation of inflammation. In Advances in lipoprotein research, T. Isbir, ed., p. 53.

Wittmann, J., Dieckow, J., Schröder, H., Hampel, U., Garreis, F., Jacobi, C., Milczarek, A., Hsieh, K.L., Pulli, B., Chen, J.W., et al. (2018). Plasma gelsolin promotes re-epithelialization. Sci. Rep. *8*, 13140.

World Health Organization. (2020). R&D blueprint novel coronavirus COVID-19 therapeutic trial synopsis (WHO). https://www.who.int/teams/blueprint/covid-19.

Wright, I., and Van Eyk, J.E. (2017). A roadmap to successful clinical proteomics. Clin. Chem. *63*, 245–247.

Yang, M.H., Yang, Y.H., Lu, C.Y., Jong, S.B., Chen, L.J., Lin, Y.F., Wu, S.J., Chu, P.Y., Chung, T.W., and Tyan, Y.C. (2012). Activity-dependent neuroprotector homeobox protein: a candidate protein identified in serum as diagnostic biomarker for Alzheimer's disease. J. Proteomics *75*, 3617–3629.

Yao, H., Lu, X., Chen, Q., Xu, K., Chen, Y., Cheng, L., Liu, F., Wu, Z., Wu, H., Jin, C., et al. (2020). Patient-derived mutations impact pathogenicity of SARS-CoV-2. medRxiv. https://doi.org/10.1101/2020.04.14.20060160.

Yu, L., Ruifrok, W.P., Meissner, M., Bos, E.M., van Goor, H., Sanjabi, B., van der Harst, P., Pitt, B., Goldstein, I.J., Koerts, J.A., et al. (2013). Genetic and pharmacological inhibition of galectin-3 prevents cardiac remodeling by interfering with myocardial fibrogenesis. Circ. Heart Fail. *6*, 107–117.

Zanoni, I., and Granucci, F. (2013). Role of CD14 in host protection against infections and in metabolism regulation. Front. Cell. Infect. Microbiol. *3*, 32.

Zhao, X., Nicholls, J.M., and Chen, Y.G. (2008). Severe acute respiratory syndrome-associated coronavirus nucleocapsid protein interacts with Smad3 and modulates transforming growth factor-β signaling. J. Biol. Chem. *283*, 3272–3280.

Zhou, F., Yu, T., Du, R., Fan, G., Liu, Y., Liu, Z., Xiang, J., Wang, Y., Song, B., Gu, X., et al. (2020a). Clinical course and risk factors for mortality of adult inpatients with COVID-19 in Wuhan, China: a retrospective cohort study. Lancet *395*, 1054–1062.

Zhou, Y., Fu, B., Zheng, X., Wang, D., Zhao, C., Qi, Y., and Sun, R. (2020b). Aberrant pathogenic GM-CSF+ T cells and inflammatory CD14+ CD16+ monocytes in severe pulmonary syndrome patients of a new coronavirus. bioRxiv. https://doi.org/10.1101/2020.02.12.945576.

CellPress
OPEN ACCESS

STAR★METHODS

KEY RESOURCES TABLE

REAGENT or RESOURCE	SOURCE	IDENTIFIER
Biological Samples		
Human Serum	Sigma-Aldrich	Cat# S7023-50MB
Human Plasma (EDTA, Pooled Donor)	Genetex	Cat# GTX73265
Human Serum, Normal off-the-Clot, Frozen	tebu-bio	Cat# 088SER
Human Recovered Plasma, Pooled- frozen K2EDTA	tebu-bio	Cat# 088SER-PLP200-EDTA
Chemicals, Peptides, and Recombinant Proteins		
Water (Optima LC-MS Grade, Fisher Chemical)	Fisher Scientific	Cat# W6500
Acetonitrile (Optima LC-MS Grade, Fisher Chemical)	Fisher Scientific	Cat# A955-500
Methanol (Optima LC-MS Grade, Fisher Chemical)	Fisher Scientific	Cat# A456-212
DL-Dithiothreitol (BioUltra)	Sigma-Aldrich	Cat# 43815
Iodoacetamide (BioUltra)	Sigma-Aldrich	Cat# I1149
Ammonium Bicarbonate (Eluent additive for LC-MS)	Sigma-Aldrich	Cat# 40867
Urea (puriss. P.a., reag. Ph. Eur.)	Honeywell Research Chemicals	Cat# 33247H
Acetic Acid (Eluent additive for LC-MS)	Honeywell Research Chemicals	Cat# 49199
Trypsin (Sequence grade)	Promega	Cat# V511X
Mass Spec-Compatible Human Extract	Promega	Cat# V6951
Retention time peptides Biognosys iRT kit	Biognosys	Cat# Ki-30002-b
MS synthetic peptide calibration kit	SCIEX	Cat# 5045759
Deposited Data		
Raw data (commercial plasma and serum control samples within the GS study)	This study	Pride:PXD018874
Software and Algorithms		
Proteomics data analysis via Deep Neural Networks, DIA-NN	Demichev et al., 2020	https://github.com/vdemichev/DiaNN
Spectronaut 13 (Version 13.12.200217.43644)	Biognosys	Product number: Sw-3001
PeakView (Version 2.2)	SCIEX	N/A
DIA-NN R package	Demichev et al., 2020	https://github.com/vdemichev/diann-rpackage
ComplexHeatmap R package	Gu et al., 2016	https://github.com/jokergoo/ComplexHeatmap
EnvStats R package	Millard, 2014	https://CRAN.R-project.org/package=EnvStats
Zoo R package		https://CRAN.R-project.org/package=zoo
Other		
Zorbax RRHD Eclipse Plus 95A C18, 2.1 x 50mm, 1.8 um, 1200 bar	Agilent	Cat# 959757-902
BioPureSPE Macro 96-Well, 100mg PROTO 300 C18	The Nest Group, Inc.	HNS S18V-L

RESOURCE AVAILABILITY

Lead Contact

Further information and requests for resources and reagents should be directed to and will be fulfilled by the Lead Contact, Markus Ralser (markus.ralser@charite.de).

Materials Availability

This study did not generate new materials.

Data and Code Availability

The raw data of the acquired commercial plasma and serum control samples within the GS study have been deposited to the ProteomeXchange Consortium via PRIDE (Perez-Riverol et al., 2019) partner repository with the dataset identifier Pride:PXD018874. According to the terms of consent for GenerationScotland participants, access to individual-level data (omics and phenotypes) must be reviewed by the GS Access Committee. Applications should be made to access@generationscotland.org. The DIA-NN software suite and DiaNN R package are open source and are freely available for download at https://github.com/vdemichev/DiaNN and https://github.com/vdemichev/diann-rpackage respectively.

EXPERIMENTAL MODEL AND SUBJECT DETAILS

Clinical Samples of COVID-19 Patients

Sampling was performed as part of the Pa-COVID-19 study, a prospective observational cohort study assessing pathophysiology and clinical characteristics of patients with COVID-19 at Charité Universitätsmedizin Berlin (Kurth et al., 2020). All patients with SARS-CoV-2 infection proven by positive PCR from respiratory specimens and willing to provide written informed consent are eligible for inclusion. Exclusion criteria are refusal to participate in the clinical study by patient or legal representative or clinical conditions that do not allow for blood sampling. The study assesses epidemiological and demographic parameters, medical history, clinical course, morbidity and quality of life during hospital stay of COVID-19 patients. Moreover, serial high-quality bio-sampling consisting of various sample types with deep molecular, immunological and virological phenotyping is performed. Treatment and medical interventions follow standard of care as recommended by current international and German guidelines for COVID-19. Severity of illness in the present study follows the WHO ordinal outcome scale (Tables S1 and S2). The Pa-COVID-19 study is carried out according to the Declaration of Helsinki and the principles of Good Clinical Practice (ICH 1996) where applicable and was approved by the ethics committee of Charité- Universitätsmedizin Berlin (EA2/066/20).

Generation Scotland Study

199 serum samples from random individuals that participated in the Generation Scotland (GS) epidemiological study (Smith et al., 2013) were used. GS is a family-based cohort of approximately 24,000 individuals in 7,000 family groups from across Scotland, aged between 18 and 98 (Smith et al., 2013). All components of Generation Scotland received ethical approval from the NHS Tayside Committee on Medical Research Ethics (REC Reference Number: 05/S1401/89). All participants provided broad and enduring written informed consent for biomedical research. Generation Scotland has also been granted Research Tissue Bank status by the East of Scotland Research Ethics Service (REC Reference Number: 15/0040/ES), providing generic ethical approval for a wide range of uses within medical research. This study was performed in accordance with the Helsinki declaration.

METHOD DETAILS

Plasma and Serum Sample Preparation

The protocol was designed for preparing four 96-well plates in parallel and that a single person using a single liquid handling unit can start and complete every day up to two 4-plate batches. The total hands-on time per batch is 3.5 hrs only and the workflow fits within the 8hr time-window. All liquid transfer and mixing except the addition of serum/plasma to the denaturing buffer was carried out by the liquid handling robot, either a Beckman Coulter Biomek NXp (Crick laboratory) or Biomek i7 liquid handling robot (Charité Universitätsmedizin Berlin). There are slight differences between the protocols due to the two different laboratories. To our knowledge, these have no detectable influence on the results. Where applicable these differences are indicated as “Biomek NXp” or “Biomek i7 protocol”, respectively.

Before starting the sample preparation, 96-well plates were prefilled with Trypsin (12.5μl, 0.1μg/μl solution; four plates/batch), denaturation/reduction buffer (55μl 8M Urea, 100mM ammonium bicarbonate (ABC) and 4.5mM dithiothreitol (DTT) (Biomek NXp protocol) or 50mM DTT (Biomek i7 protocol); four plates/batch) and iodoacetamide (IAA) (100mM, > 20 μl, one plate/batch) and stored sealed at -80°C until the day of the experiment. These stock solutions are thawed/brought to room temperature just before adding them to the sample, which prevents evaporation.

5μl of thawed serum/plasma samples were transferred to the pre-made denaturation/reduction stock solution plates. Subsequently the plates were centrifuged for 15s at pulse setting (Eppendorf Centrifuge 5810R), mixed and incubated at 30°C for 60 minutes. The mixing in this step was done either 30s at 1000rpm on a Thermomixer (Eppendorf Thermomixer C) (Biomek NXp protocol)

or by resuspension (Biomek i7 protocol). 5μl IAA was then transferred from the respective stock solution plate to the sample plate and incubated in the dark at 23°C for 30 minutes before dilution with 100mM ABC buffer (340μl). 220μl of this solution was transferred to the pre-made trypsin stock solution plate and incubated at 37°C for 17 h (Memmert IPP55 incubator). The trypsin/total protein ratio was ~1/40 as this provided high and reproducible identification numbers (Figure S2D). The digestion was quenched by addition of formic acid (10% v/v, 25μl). The digestion mixture was cleaned-up using C18 96-well plates (BioPureSPE Macro 96-Well, 100mg PROTO C18, The Nest Group). For the solid phase extraction, 1 minute of centrifugation at the described speeds (Eppendorf Centrifuge 5810R) was used to push the liquids through the stationary phase and the liquid handler was used to pipette the liquids onto the material in order to make four 96-well plates/batch feasible. The plates were conditioned with methanol (200μl, centrifuged at 50g), washed twice with 50% ACN (200μl, centrifuged at 150g and flow through discarded), equilibrated twice with 0.1% FA (200μl, centrifuged at 150g and flow through discarded). Then 200μl of digested and quenched samples were loaded (centrifuged at 150g), washed twice with 0.1% FA (200μl, centrifuged at 150g). After the last washing step, the plates were centrifuged another time at 200g before the peptides were eluted in 3 steps with 110μl 50% ACN (200g) into a collection plate (1.1ml, Square well, V-bottom). Collected material was completely dried on a vacuum concentrator (Eppendorf Concentrator Plus (Biomek NXp protocol) or Fisher Scientific, SPD300P1 (Biomek i7 protocol) and redissolved in 50μl 1% ACN, 0.1% formic acid (Biomek NXp protocol) or 50μl 0.1% formic acid (Biomek i7 protocol), then stored at -80°C until data acquisition. The samples for the SARS-CoV-2 studies were analysed without freezing. QC samples for repeat injections were prepared by pooling commercial serum samples and were spiked with iRT peptides (Biognosys).

Liquid Chromatography-Mass Spectrometry Setup

Liquid chromatography was established on two complementary and exchangeable ultra-high-pressure high-flow LC-MS systems, an Agilent 1290 Infinity II (Crick laboratory) and Waters H-Class (Charité Universitätsmedizin Berlin) system, both coupled to a TripleTOF 6600 mass spectrometer (SCIEX) equipped with IonDrive Tubo V Source (Sciex). In both cases, the peptides were separated in reversed phase mode using a C18 ZORBAX Rapid Resolution High Definition (RRHD) column 2.1mm x 50mm, 1.8μm particles at a column temperature of 30°C. A linear gradient was applied which ramps from 3% B to 36% B in 5 minutes (Buffer A: 0.1% FA; Buffer B: ACN/0.1% FA) with a flow rate of 800μl/min. For washing the column, the organic solvent was increased to 80% B in 0.5 minutes and was kept for 0.2 minutes at this composition before going back to 1% B in 0.3 min. The equilibration times were 2.8 minutes (Water H Class protocol) or 4.2 minutes (Agilent Infinity II protocol). Data was acquired in high sensitivity mode and the amount of total proteins injected was 5μg (GS study) and 10μg (SARS-CoV-2). The sample load was optimised (Figure S2A) and is a balance between how frequently one needs to clean the instrument and the identification numbers. The DIA/SWATH method consisted of an MS1 scan from m/z 100 to m/z 1500 (20ms accumulation time) and 25 MS2 scans (25ms accumulation time) with variable precursor isolation width covering the mass range from m/z 450 to m/z 850 (Table S6). Ion source gas 1 (nebulizer gas), ion source gas 2 (heater gas) and curtain gas were set to 50, 40 and 25 respectively. The source temperature was set to 450°C and the ion spray voltage to 5500V.

QUANTIFICATION AND STATISTICAL ANALYSIS

Mass Spectrometry Data Processing, Batch Correction and Quality Control

The raw data were processed using DIA-NN 1.7.10 in the "robust LC (high precision)" mode with RT-dependent median-based cross-run normalisation enabled. MS2 and MS1 mass accuracies were set to 20 and 12 ppm, respectively, and scan window size set to 6. Although DIA-NN can optimise such parameters automatically, we fixed them to these values to ensure comparability. For all the experiments, we used a project-independent public spectral library (Bruderer et al., 2019). Human UniProt (UniProt Consortium, 2019) isoform sequence database (3AUP000005640) was used to annotate the library. The library was first automatically refined based on the dataset in question at 0.01 global q-value (using the "Generate spectral library" option in DIA-NN). DIA-NN performs such refinement by finding the highest-scoring identification for each library precursor, across all runs in the experiment, and then replacing the library data with the empirically observed spectrum and retention time. The purpose of the refinement step is twofold: (i) retain only those peptide precursors in the library that are detectable in the experiment of interest; (ii) make sure that the library spectra and retention times are optimised specifically for the experimental setup in question, thus improving identification performance. The refined library was then used to reanalyse the data. The resulting report was stringently filtered at 0.01 precursor-level q-value, 0.005 precursor-level library q-value and 0.05 protein group-level q-value. Intra-batch correction was performed for each peptide precursor separately: based on repeat injection controls in "GS" and sample preparation controls in the coronavirus cohorts. Log-transformed peptide precursor quantities were adjusted using linear regression ("GS") or running median smoothing (coronavirus cohorts). Linear regression was applied only for at least 10 data points. If the p-value for non-zero slope was below 0.01, the slope multiplied by the centered injection number (i.e. the injection number in the given run minus its mean value in all runs) was subtracted from the precursor quantities for all runs. Running median smoothing was performed in two steps using the 'runmed' function with the algorithm set to 'Stuetzle'. First, a 5-point running median smoothing was performed on the quantities in the control samples, to remove outliers. Second, interpolation of the resulting values to all runs was performed using the 'na.approx' function (with 'rule' set to 2) from the 'zoo' R package. Finally 41-point running median smoothing was applied. Protein quantification was performed using the MaxLFQ algorithm (Cox et al., 2014) as implemented in the diann R package (https://github.com/vdemichev/diann-rpackage, version 1.0, commit "eb4607a"). Data completeness was defined as the proportion of non-missing

values in the proteins x samples quantities matrix. The coefficient of variation (CV) was calculated for each protein as its empirical standard deviation divided by its empirical mean. PCA analysis was always performed only on ubiquitously identified proteins: imputation was not used.

Differential Expression Analysis

In the exploratory cohort, differential expression was tested only for proteins quantified using at least five peptide precursors in one of the acquisitions. Further, protein groups were quantified using only precursors detected in at least 10% of patient samples (both cohorts). As we were specifically interested in proteins which could serve as biomarkers of COVID-19 severity, the test was performed using the Kendall's Tau test for the Theil-Sen trend estimator (as implemented in the EnvStats R package (Millard, 2014)) against the disease severity as classified according to the WHO ordinal scale (Table S1). The input for the test was obtained by calculating, for each patient, median protein levels across the timepoints or replicates measured (Table S4 for timepoint information). Multiple testing correction was performed using the Holm-Bonferroni method for FWER control, as implemented in the p.adjust R function, and the significance threshold was set to 0.05. The choice of a nonparametric test (Theil-Sen) was dictated by the fact that such widespread methods as ANOVA or linear regression are only valid under the assumption of Gaussian errors with the same variance across all conditions. In the case of this dataset, however, we observed very significant differences in the variance, e.g. many proteins seem a lot more variable between patients with severe and critical COVID-19 than mild COVID-19. For such a situation a nonparametric test is ideal: although it would typically have less power (less proteins detected as differentially expressed), the p-values produced are reliable.

Chromatographic Peaks Full Width at Half Maximum (FWHM) Estimations

Median peak FWHM was estimated using Spectronaut 13 (version 13.12.200217.43644; Biognosys). Only precursors ubiquitously identified in all runs (3 minutes, 5 minutes, 10 minutes and 20-minute high-flow as well as with the 20-minute micro-flow run) and with a q-value of < 0.001 were considered (804 precursors total).

Total Ion and Extracted Ion Chromatograms

Total ion chromatograms and extracted ion chromatograms were generated with the PeakView software (Version 2.2, SCIEX), exported and plotted in R (R core team, www.R-project.org).

Cell Systems, Volume *11*

Supplemental Information

Ultra-High-Throughput Clinical Proteomics

Reveals Classifiers of COVID-19 Infection

Christoph B. Messner, Vadim Demichev, Daniel Wendisch, Laura Michalick, Matthew White, Anja Freiwald, Kathrin Textoris-Taube, Spyros I. Vernardis, Anna-Sophia Egger, Marco Kreidl, Daniela Ludwig, Christiane Kilian, Federica Agostini, Aleksej Zelezniak, Charlotte Thibeault, Moritz Pfeiffer, Stefan Hippenstiel, Andreas Hocke, Christof von Kalle, Archie Campbell, Caroline Hayward, David J. Porteous, Riccardo E. Marioni, Claudia Langenberg, Kathryn S. Lilley, Wolfgang M. Kuebler, Michael Mülleder, Christian Drosten, Norbert Suttorp, Martin Witzenrath, Florian Kurth, Leif Erik Sander, and Markus Ralser

Table S1. **Scoring of exploratory and validation COVID-19 cohorts**.

**Exploratory Cohort**						
Patient	WHO score	WHO category	Clinical assessment (Charité)	Age	Sex	Outcome
1	3	mild	mild	21	m	discharged
2	3	mild	mild	31	f	discharged
3	3	mild	mild	45	m	discharged
10	3	mild	mild	50	f	discharged
18	3	mild	mild	60	f	discharged
19	3	mild	mild	52	m	discharged
23	3	mild	mild	44	f	discharged
27	3	mild	mild	40	f	discharged
28	3	mild	mild	64	m	discharged
34	3	mild	mild	47	m	discharged
35	3	mild	mild	37	f	discharged
37	3	mild	mild	78	m	discharged
4*			severe*	37	m	
6	3	mild	severe	24	m	discharged
5	4	mild	severe	71	m	discharged
7	4	mild	severe	32	m	discharged
24	4	mild	severe	64	m	discharged
29	4	mild	severe	53	m	discharged
11	5	severe	severe	61	m	discharged
16	5	severe	severe	78	m	still hospitalized
22	5	severe	severe	62	m	discharged
26	5	severe	severe	56	f	discharged
12	6	severe	critical	75	m	discharged
20	6	severe	critical	54	f	discharged
21	6	severe	critical	52	m	discharged
8	7	severe	critical	63	m	still hospitalized
9	7	severe	critical	80	f	still hospitalized
13	7	severe	critical	71	m	death
14	7	severe	critical	81	m	death
15	7	severe	critical	54	m	death
25	7	severe	critical	62	f	still hospitalized
32	7	severe	critical	50	f	death

Validation Cohort						
Individual/ Patient	WHO score	WHO category	Clinical category	Age	Sex	Outcome
HD-1	0	uninfected	healthy	27	f	
HD-2	0	uninfected	healthy	23	m	
HD-3	0	uninfected	healthy	34	f	
HD-4	0	uninfected	healthy	31	f	
HD-5	0	uninfected	healthy	30	m	
HD-6	0	uninfected	healthy	41	f	
HD-7	0	uninfected	healthy	24	f	
HD-8	0	uninfected	healthy	27	m	
HD-9	0	uninfected	healthy	30	f	
HD-10	0	uninfected	healthy	34	f	
HD-11	0	uninfected	healthy	42	m	
HD-12	0	uninfected	healthy	30	f	
HD-13	0	uninfected	healthy	33	f	
HD-14	0	uninfected	healthy	29	f	
HD-15	0	uninfected	healthy	36	f	
49	3	mild	mild	72	f	discharged
56	3	mild	mild	58	f	discharged
65	3	mild	mild	22	m	discharged
66	3	mild	mild	75	f	discharged
75	3	mild	mild	84	m	discharged
38	4	mild	severe	70	m	discharged
48	4	mild	severe	48	m	discharged
50	4	mild	severe	78	m	discharged
63	5	severe	severe	61	m	discharged
57	6	severe	critical	55	f	discharged
60	6	severe	critical	65	m	discharged
62	6	severe	critical	72	m	discharged
33	7	severe	critical	35	f	Still hospitalized
58	7	severe	critical	26	m	Still hospitalized
59	7	severe	critical	86	m	death
61	7	severe	critical	54	m	Still hospitalized
64	7	severe	critical	57	m	Still hospitalized

*A secondary assessment triggered by blind proteome clustering (Figure S3) revealed an Influenza Type B rather than a COVID-19 infection.

Table S2. **WHO scoring for COVID-19 cases used in the study (World Health Organisation 2020).**

**Patient state**	**Descriptor**	**Score**
Uninfected	No clinical or virological evidence of infection	0
Ambulatory	No limitation of activities	1
	Limitation of activities	2
Hospitalised - mild disease	No oxygen therapy	3
	Oxygen by mask or nasal prongs	4
Hospitalised - severe disease	Non-invasive ventilation or high-flow oxygen	5
	Intubation and mechanical ventilation	6
	Ventilation + additional organ support (pressors, renal replacement therapy (RRT), extracorporeal membrane oygenation (ECMO))	7

Table S3. **Proteins differentially expressed depending on COVID-19 severity**.

Gene symbols	Names	Possible relevance to SARS-CoV-2 infection (speculative)
**Upregulated**		
A1BG	Alpha-1B-Glycoprotein	Function poorly understood, maybe related to hypoxia as indicated from a study in cattle (Kong et al., 2019).
ACTB;ACTG1	Actin Beta and Gamma-1	F-actin is released at the site of tissue injury and scavenged by plasma Gelsolin (DiNubile, 2008)
C1R	Complement C1r	Initiates complement activation (Hajishengallis et al., 2017; Ricklin et al., 2010, 2016).
C1S	Complement C1s	Initiates complement activation, activated by C1R (Hajishengallis et al., 2017; Ricklin et al., 2010, 2016).
C8A	Complement C8 Alpha Chain	Part of the complement system (Hajishengallis et al., 2017; Ricklin et al., 2010, 2016).
CD14	Monocyte Differentiation Antigen CD14	CD14 is primarily displayed by monocytes and macrophages and can be released in a soluble form; soluble CD14 is also produced by the liver. Primarily involved in the recognition of bacterial LPS, its upregulation might be indicative in immune response dysregulation by SARS-CoV-2. CD14 has also been implicated in a broader spectrum antigen response and lung inflammation (Anas et al., 2010). Upregulation of CD14 expression levels in monocytes was previously reported upon SARS-Cov infection (Hu et al., 2012). Cytokines can induce the release of soluble CD14 (Shive et al., 2015) and production of soluble CD14 in the liver is induced by IL-6 (Bas et al., 2004).
CFB	Complement Factor B	Part of the alternative complement pathway (Hajishengallis et al., 2017; Ricklin et al., 2010, 2016).
CFH	Complement Factor H	Modulates complement activation mainly via inhibition of the alternative complement pathway (Hajishengallis et al., 2017; Ricklin et al., 2010,

		2016).
CFI	Complement Factor I	Modulates complement activity via degradation of C3b and C4b (Hajishengallis et al., 2017; Ricklin et al., 2010, 2016).
CRP	C-Reactive Protein	Acute phase response protein, strongly upregulated in COVID-19 (Shi et al., 2020).
FGA, FGB, FGG	Fibrinogen Alpha, Beta and Gamma Chains	Fibrinogen levels are elevated in acute-phase response (Jain et al., 2011), upregulated in COVID-19 (Shi et al., 2020).
HP	Haptoglobin	Elevated in acute-phase response (Jain et al., 2011). Of note, HP expression is known to be promoted by IL-6 (Li et al., 2020), which is elevated in severe COVID-19 (Ruan et al., 2020).
ITIH3	Inter-Alpha-Trypsin Inhibitor Heavy Chain 3	Involved in inflammatory response to trauma (Hamm et al., 2008)
ITIH4	Inter-Alpha-Trypsin Inhibitor Heavy Chain 4	Involved in inflammatory response to trauma (Hamm et al., 2008). Production of ITIH4 in the liver is upregulated by IL-6 (Bhanumathy et al., 2002).
LBP	Lipopolysaccharide Binding Protein	Recognises bacterial LPS, upregulation might be indicative in immune response dysregulation by SARS-CoV-2.
LGALS3BP	Galectin 3 Binding Protein	Upregulated in viral infections, promotes inflammation (Xu et al., 2019), has been shown to induce IL-6 expression (Silverman et al., 2012).
LRG1	Leucine-Rich Alpha-2-Glycoprotein	LRG1 expression is induced by IL-6 (Shirai et al., 2009), which is elevated in severe COVID-19 (Ruan et al., 2020). LRG1 promotes angiogenesis (Wang et al., 2013) and has been reported to be associated with local inflammation (Naka and Fujimoto, 2018). LRG1 is known to promote lung fibrosis (Honda et al., 2017). Secretion of LRG1 by neutrophils upon activation has been reported (Druhan et al., 2017). Of note, neutrophil-lymphocyte-ratio is elevated in COVID-19 (Qin et al., 2020).
SAA1 and SAA1;SAA2	Serum Amyloid A1 and A2	SAA1 and SAA2 are markers of inflammatory response and tissue injury (Sack, 2018).

		Expression is known to be induced by IL-6 (Hagihara et al., 2004).
SERPINA10	Protein Z-Dependent Protease Inhibitor	Acts in complex with Protein Z to inhibit the F10a coagulation factor (Almawi et al., 2013), while coagulation has been observed to be increased in severe COVID-19 (Zhou et al., 2020).
**Downregulated**		
ALB	Albumin	Low levels associated with acute-phase response (Soeters et al., 2019) and observed in critical COVID-19 (Zhou et al., 2020).
APOA1	Apolipoprotein A1	Major component of the High-Density Lipoprotein (HDL) complex, which is a modulator of innate immune response and inflammation (Fotakis et al., 2019; Gordon et al., 2011; Macpherson et al., 2019; White et al., 2017). Although it might be that APOA1 levels drop upon the infection, we can also speculate that low APOA1 levels are a risk factor for COVID-19. Notably, We observe clear correlation of APOA1 with HDL cholesterol and only weak negative correlation with age (Figure S6). Of note, decreased APOA1 levels have been associated with poor prognosis in systemic inflammatory response (Kumaraswamy et al., 2012; Sirniö et al., 2017; Tani et al., 2016).
APOC1	Apolipoprotein C1	Part of multiple apolipoprotein complexes, has links to immune response and inflammation (Fuior and Gafencu, 2019).
GSN	Gelsolin	Low levels of plasma gelsolin are associated with inflammation. One of the primary mechanisms leading to this is believed to be the recruitment of gelsolin, which has actin recycling function, to the sites of tissue injury, depleting its plasma levels. Of note, we observed increased serum levels of the ACTB;ACTG1 protein group in severe and critical COVID-19 cases. Importantly, plasma gelsolin is a modulator of inflammation, which carries a protective function (DiNubile, 2008; Li et al., 2012). Low plasma gelsolin is a marker of poor prognosis in various pathological conditions, including diabetes (Khatri et al., 2014), cancers (Asare-Werehene et al., 2019; Stock et al., 2015) and sepsis (Lee et al., 2007), leading to

		suggestions and animal tests for its therapeutic use. Of note, treatment with gelsolin has been observed to decrease IL-6 levels in mice (Cheng et al., 2017). Gelsolin treatment has been suggested to promote epithelial repair (Wittmann et al., 2018).
TF	Transferrin	Iron carrier, observed to decrease in acute phase response (Jain et al., 2011).

Table S4. **Date of symptom onset and blood sampling for the patients from the exploratory cohort.**

Patient	Onset of symptoms	Admission to hospital	blood sampling
1	15/02/2020	01/03/2020	02/03,04/03,06/03,07/03,09/03,10/03,11/03,12/03,13/03,16/03,18/03,20/03,23/03,23/03,25/03,27/03
2	25/02/2020	02/03/2020	04/03,06/03,07/03,08/03,09/03
3	29/02/2020	11/03/2020	11/03,12/03,13/03,16/03,18/03,20/03,23/03,23/03,25/03
5	28/02/2020	11/03/2020	12/03,13/03,16/03,18/03
6	11/03/2020	12/03/2020	13/03,16/03,18/03
7	08/03/2020	14/03/2020	16/03,19/03,20/03,23/03,25/03
8	21/02/2020	15/03/20	20/03,23/03,25/03,27/03
9	12/03/2020	16/03/2020	17/03,17/03,20/03,23/03,25/03,27/03
10	09/03/2020	17/03/2020	19/03,20/03
11	08/03/2020	17/03/2020	20/03,23/03,25/03,27/03
12	21/02/2020	18/03/2020	17/03,20/03,23/03,25/03,27/03
13	13/03/2020	19/03/2020	20/03,23/03,25/03,27/03
14	15/03/2020	21/03/2020	23/03
15	NA	17/03/2020	23/03,25/03
16	19/03/2020	22/03/2020	23/03,25/03,27/03

18	04/03/2020	10/30/2020	25/03
19	14/03/2020	19/03/2020	23/03,25/03
20	12/03/2020	20/30/2020	23/03,25/03,27/03
21	12/03/2020	20/03/2020	23/03,25/03,27/03
22	09/03/2020	18/03/2020	23/03,25/03,27/03
23	NA	23/03/2020	23/03,25/03,27/03
24	06/03/2020	21/03/2020	25/03,27/03
25	NA	23/03/2020	25/03,27/03
26	15/03/2020	24/03/2020	25/03,27/03
27	12/03/2020	12/03/2020	25/03
28	20/03/2020	23/03/2020	25/03
29	13/03/2020	23/03/2020	25/03,27/03
32	NA	21/03/2020	27/03
34	21/03/2020	26/03/2020	27/03
35	15/03/2020	25/03/2020	27/03
37	10/03/2020	24/03/2020	27/03

Table S5. **List of FDA approved biomarkers, quantified with ultra-high-throughput serum proteomics.**

**Protein Name**
Albumin<br>α-1-acid glycoprotein<br>α-1-antitrypsin<br>α-2-antiplasmin<br>α-2-HS-glycoprotein<br>α-2-macroglobulin<br>Antithrombin III<br>Apolipoprotein B<br>β-2-microglobulin

β-Thromboglobulin
Biotinidase
Ceruloplasmin
Cholinesterase
Complement C1
Complement C1Q
Complement C3
Complement C4
Complement C5
CRP
Cystatin C
Factor IX antigen
Factor X
Factor XIII
Fibrinogen
Fibronectin
Haptoglobin
Hemopexin
IgG
IgM
Insulin-like growth factor II
IGFBP-3
κ light chains
λ light chains
Lipoprotein (a)
Lysozyme
Plasminogen
Prealbumin / transthyretin
Properdin factor B
Protein S
Retinol binding protein
Sex hormone-binding globulin
Thyroxine binding globulin
Transferrin receptor
Von Willebrand factor

Table S6. **Lower and upper m/z limits of the SWATH precursor selection windows used.**

Lower m/z limit	Upper m/z limit	CE Spread
449.5	468.9	5
467.9	486.4	5
485.4	503.3	5
502.3	519.8	5
518.8	535.7	5
534.7	550.9	5
549.9	565.5	5
564.5	579.7	5
578.7	594	5
593	607.9	5
606.9	621.9	5
620.9	635.8	5
634.8	649.7	5
648.7	663.3	5
662.3	676.3	5
675.3	689.6	5
688.6	702.9	5
701.9	716.8	5
715.8	732.3	5
731.3	748.9	5
747.9	766.4	5
765.4	784.2	5
783.2	803	5

802	824.7	5
823.7	849.6	5

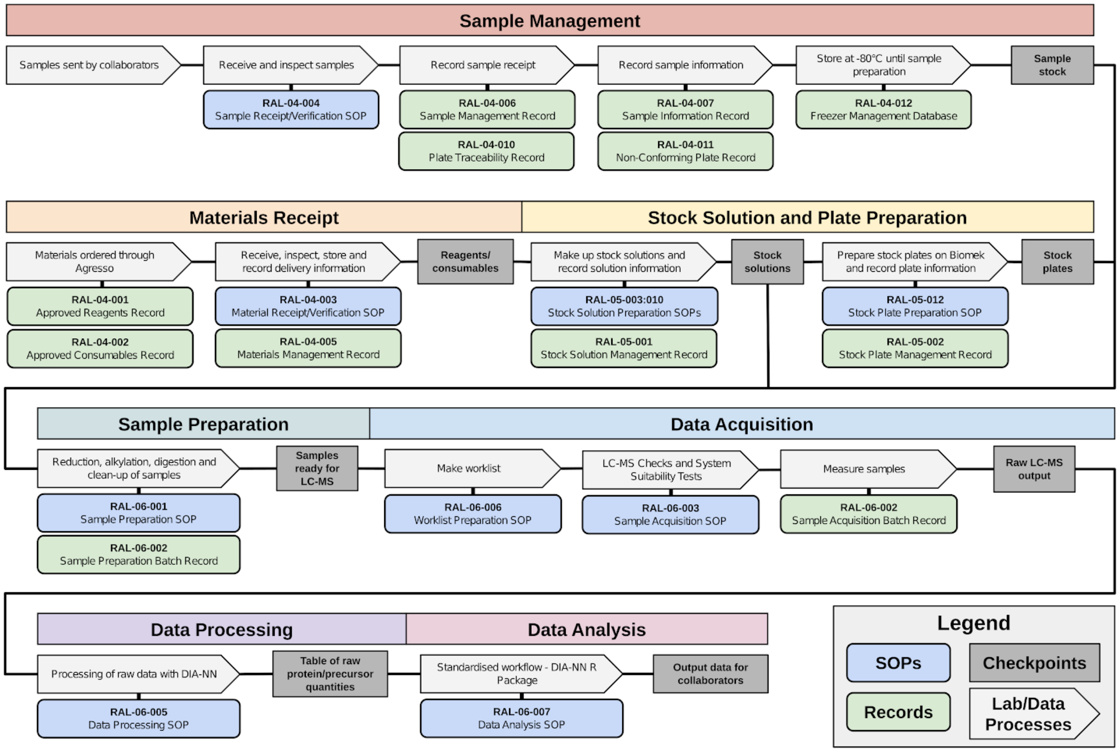

Figure S1, Related to Figure 1. **Detailed overview of the high-throughput proteomics workflow.** This figure details the documentation and management tasks orienting on ISO13485 standardisation for medical devices as reference.

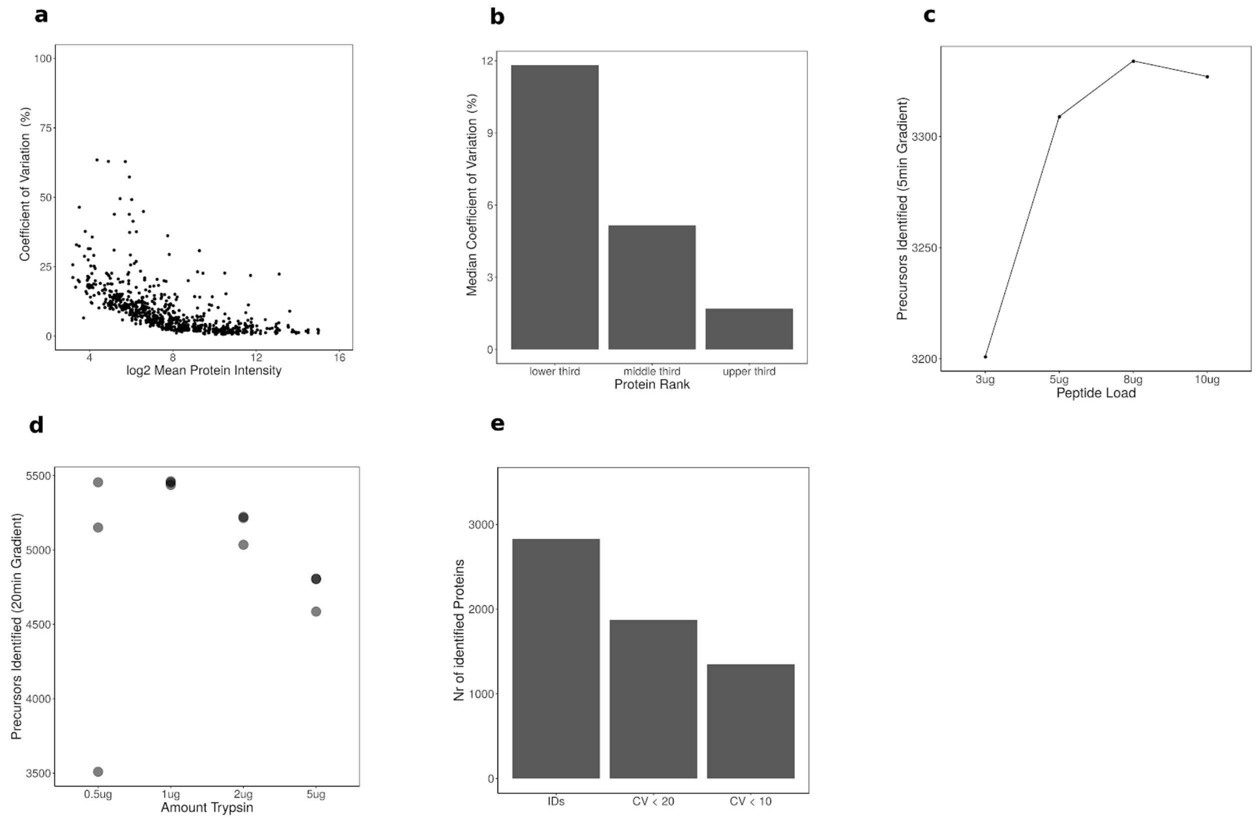

Figure S2. **Quality parameters of protein quantification in ultra-high-throughput proteomics. a**. Coefficient of Variation (%) as a function of log2 mean protein intensity for 39 repeated injections of a pooled whole-serum tryptic digest that were run over a period of > 4 days, to test for instrument stability.. **b.** Median CV value for low, middle, and high-intensity proteins in the 39 repeated injections. **c.** Number of precursors identified in 5-min high-flow SWATH runs with different amounts of serum proteins injected. **d.** Number of precursors identified in a plasma sample with different amounts of trypsin used for digestion, run with a 20-min water to acetonitrile gradient. **e.** Proteins identified in triplicate injections of a mammalian (K562) cell lysate using the 5-minute high-flow SWATH runs presented in this study. Total number of unique Proteins (1% FDR), number of unique Proteins quantified with less than 20% coefficient of variation (CV) and less than 10% CV are shown.

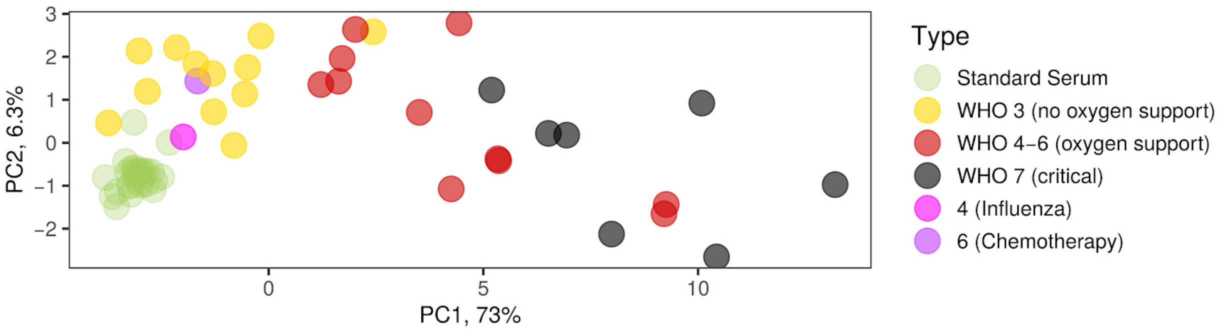

Figure S3: **Clustering of untargeted plasma proteomes led to the reclassification of two study participants.** Principal component analysis based on proteins differentially expressed depending on COVID-19 severity grading according to WHO. Patients 4 and 6 were initially clinically assessed as “severe” (Braun et al, 2020) but clustered with patients that suffered from a mild form of COVID-19. A retrospective assessment revealed that patient 6 had received R-CHOP chemotherapy 10 days prior to study inclusion and patient 4 was in fact suffering from type B influenza rather than SARS-CoV-2 infection. Patient 6 was categorized as mild by using the WHO ordinal outcome scale of clinical improvement (World Health Organisation, 2020) and Patient 4 was removed from the analysis. Please note that the PCA transformation is slightly different from that in Figure 4b, as the addition of two extra samples for patient 4 led to slightly different protein quantities being calculated for all the samples by the MaxLFQ algorithm (Cox et al., 2014).

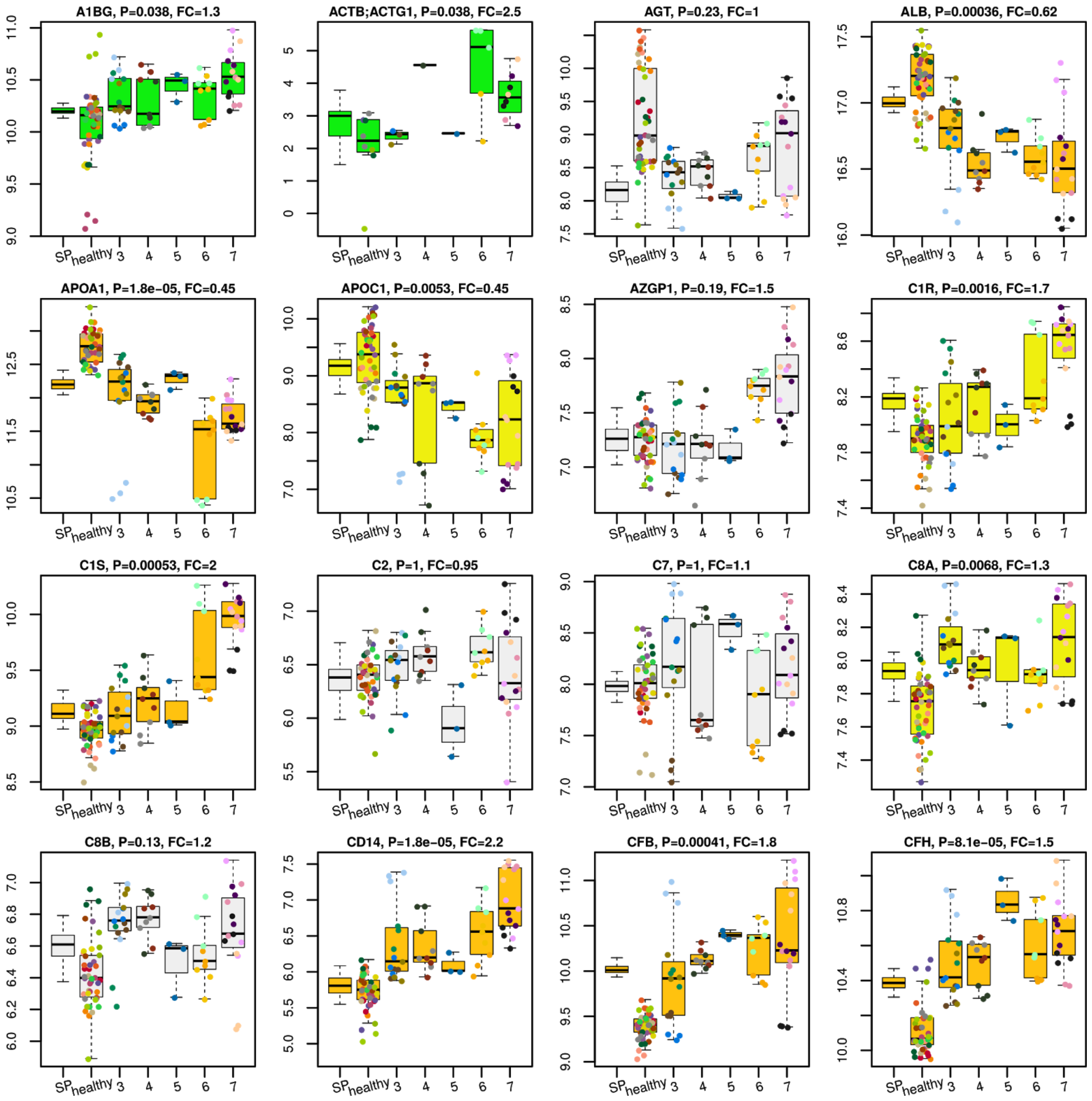
A1BG, P=0.038, FC=1.3
ACTB;ACTG1, P=0.038, FC=2.5
AGT, P=0.23, FC=1
ALB, P=0.00036, FC=0.62
APOA1, P=1.8e-05, FC=0.45
APOC1, P=0.0053, FC=0.45
AZGP1, P=0.19, FC=1.5
C1R, P=0.0016, FC=1.7
C1S, P=0.00053, FC=2
C2, P=1, FC=0.95
C7, P=1, FC=1.1
C8A, P=0.0068, FC=1.3
C8B, P=0.13, FC=1.2
CD14, P=1.8e-05, FC=2.2
CFB, P=0.00041, FC=1.8
CFH, P=8.1e-05, FC=1.5
SPhealthy
3
4
5
6
7

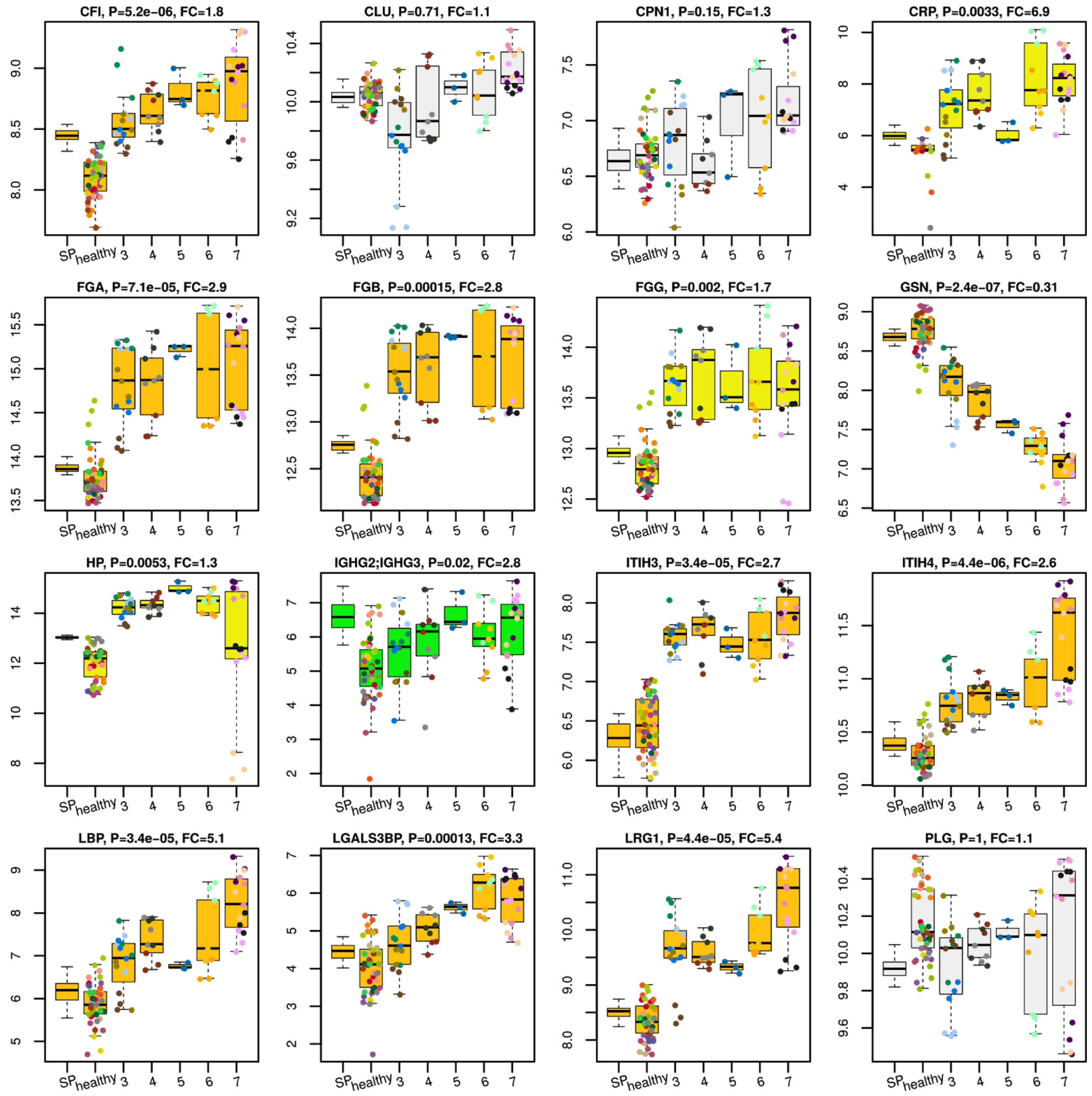
CFI, P=5.2e−06, FC=1.8
CLU, P=0.71, FC=1.1
CPN1, P=0.15, FC=1.3
CRP, P=0.0033, FC=6.9
FGA, P=7.1e−05, FC=2.9
FGB, P=0.00015, FC=2.8
FGG, P=0.002, FC=1.7
GSN, P=2.4e−07, FC=0.31
HP, P=0.0053, FC=1.3
IGHG2;IGHG3, P=0.02, FC=2.8
ITIH3, P=3.4e−05, FC=2.7
ITIH4, P=4.4e−06, FC=2.6
LBP, P=3.4e−05, FC=5.1
LGALS3BP, P=0.00013, FC=3.3
LRG1, P=4.4e−05, FC=5.4
PLG, P=1, FC=1.1
SP healthy 3 4 5 6 7

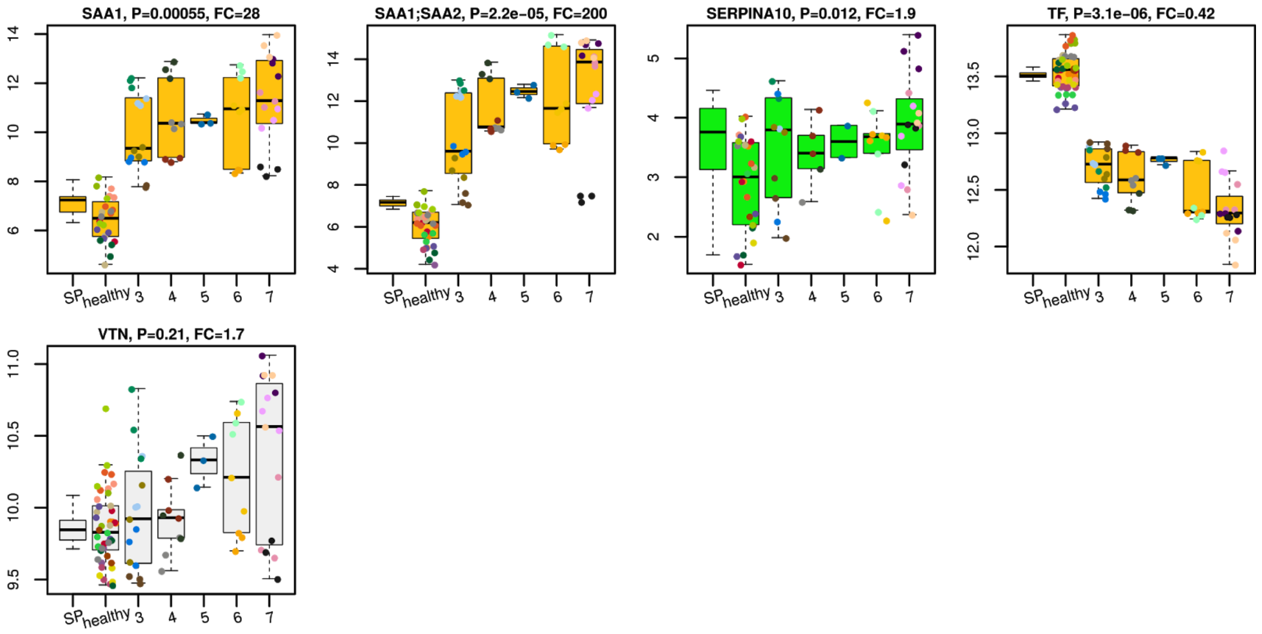

Figure S4. **Proteomes in mild, severe and critical SARS-CoV-2 infection for patients in the validation cohort**. Differentially expressed proteins are highlighted with colours depending on the multiple testing-corrected p-values: green: P < 0.05, yellow: P < 0.01, orange: P < 0.001.

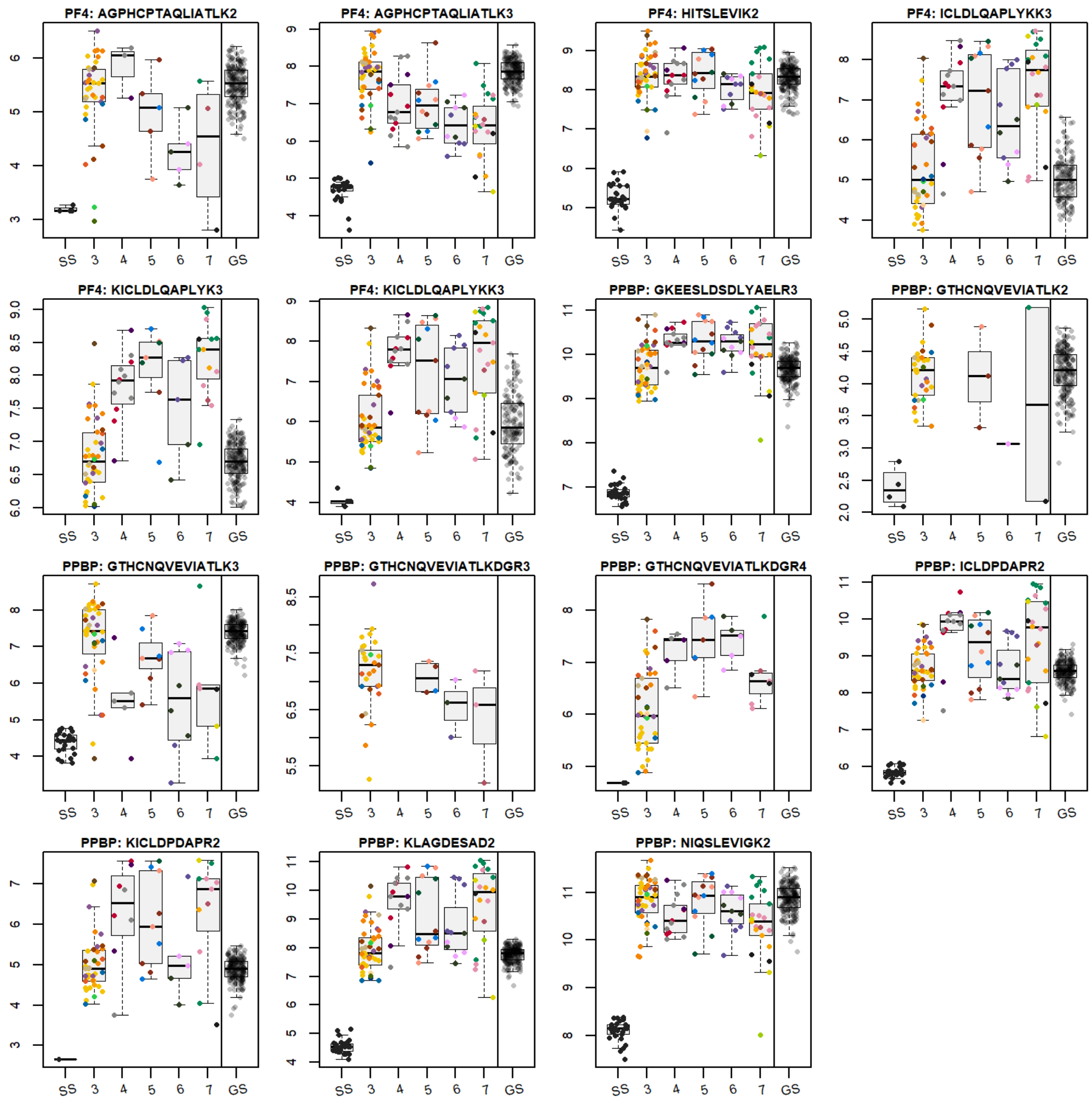

Figure S5. **Differential response of peptide precursors mapping to PF4 and PPBP**. Boxplots are presented for log_2-transformed peptide precursor quantities. The peptide precursor charge is indicated with a number after the amino acid sequence. All cysteines were considered carbamidomethylated. Boxplots are drawn based on individual measurements (multiple timepoints per patient), which are indicated with points (different colours for different patients, ordered from left to right by the time taken). Standard Serum sample preparation controls are indicated with “SS” and represent the technical variability (sample preparation + LC-MS). The last box represents the variability seen in the GS study; as the absolute quantities from the two studies cannot be compared directly (the data was acquired on different LC-MS setups), to

simplify the visual assessment of the variability, the median of GS quantities was matched to the median of WHO grade 3 (no oxygen support) COVID-19 cases.

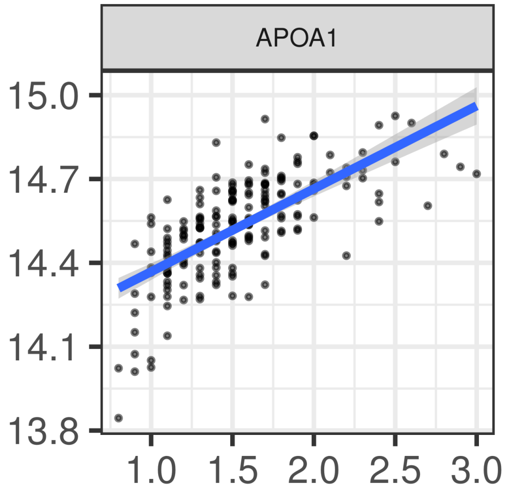

Figure S6. **Log$_2$-transformed serum levels of Apolipoprotein A1 (APOA1; arbitrary units) plotted against the HDL-Cholesterol concentration (mmol/L) in the GS study**.

Figure S7 (following 18 pages). **Proteomes in mild, severe and critical SARS-CoV-2 infection (complete list)**. Differentially expressed proteins are highlighted with colours depending on the multiple testing-corrected p-values: green: P < 0.05, yellow: P < 0.01, orange: P < 0.001.

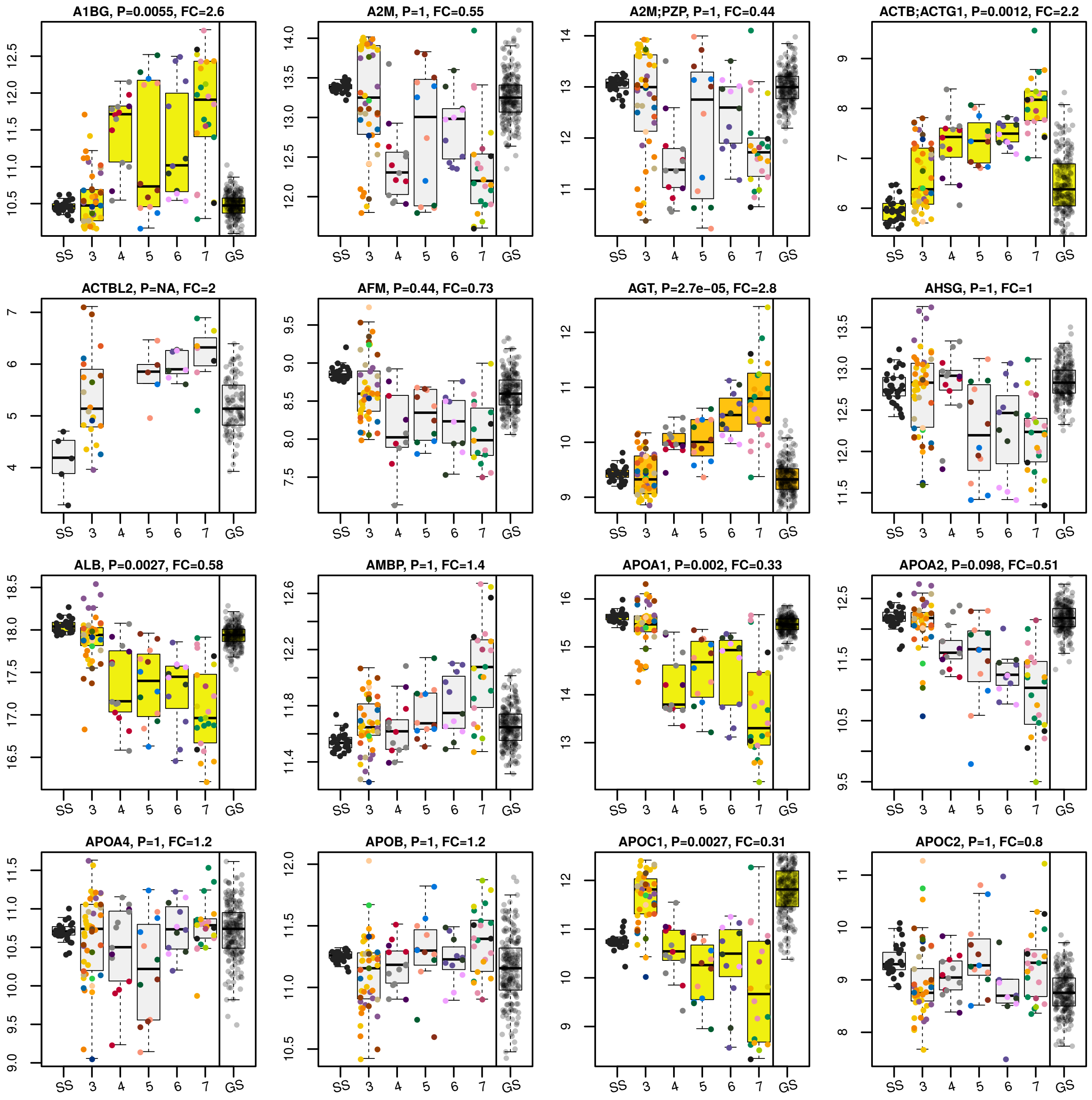
A1BG, P=0.0055, FC=2.6
A2M, P=1, FC=0.55
A2M;PZP, P=1, FC=0.44
ACTB;ACTG1, P=0.0012, FC=2.2
ACTBL2, P=NA, FC=2
AFM, P=0.44, FC=0.73
AGT, P=2.7e-05, FC=2.8
AHSG, P=1, FC=1
ALB, P=0.0027, FC=0.58
AMBP, P=1, FC=1.4
APOA1, P=0.002, FC=0.33
APOA2, P=0.098, FC=0.51
APOA4, P=1, FC=1.2
APOB, P=1, FC=1.2
APOC1, P=0.0027, FC=0.31
APOC2, P=1, FC=0.8
SS
3
4
5
6
7
GS

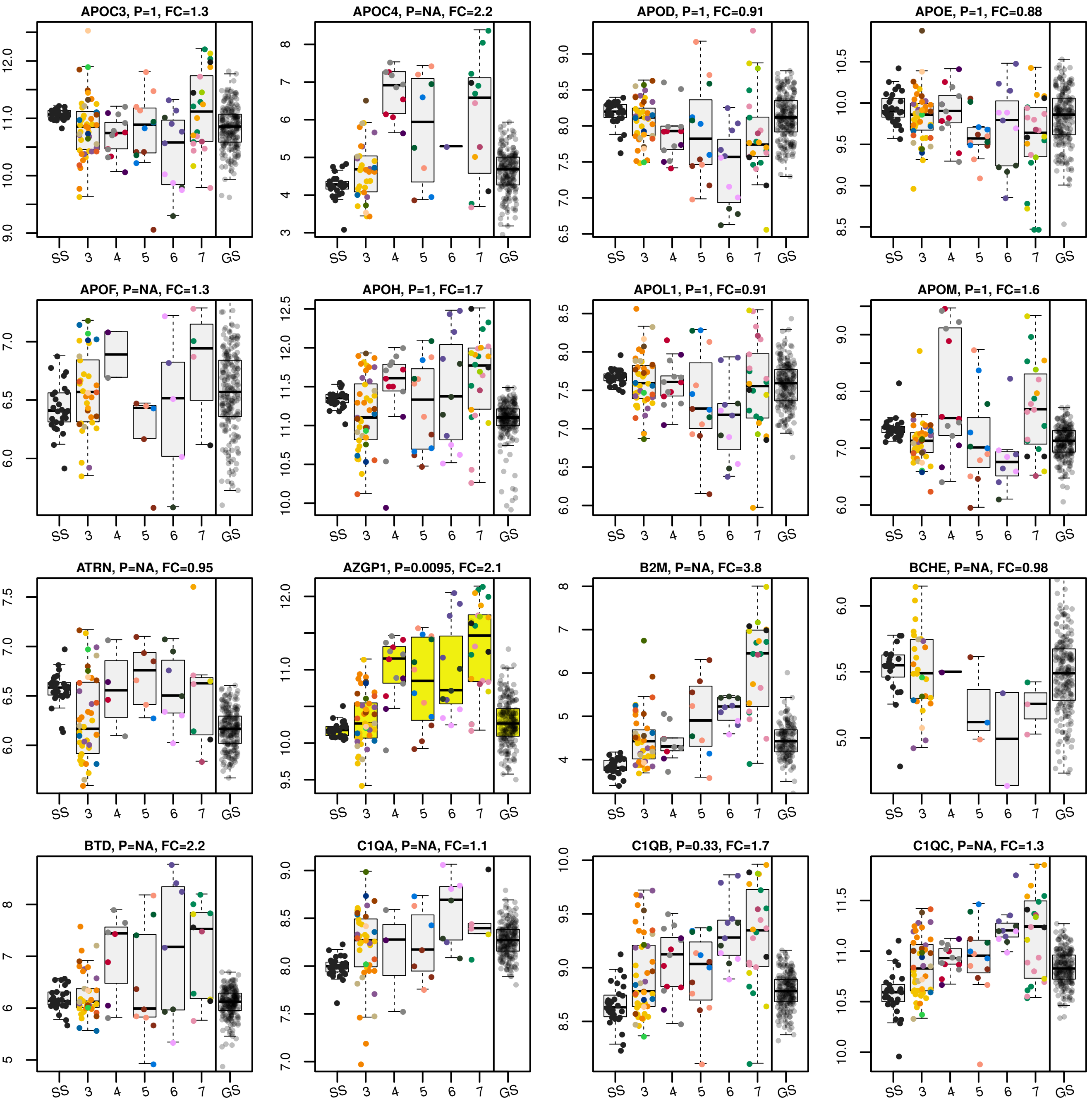
APOC3, P=1, FC=1.3
APOC4, P=NA, FC=2.2
APOD, P=1, FC=0.91
APOE, P=1, FC=0.88
APOF, P=NA, FC=1.3
APOH, P=1, FC=1.7
APOL1, P=1, FC=0.91
APOM, P=1, FC=1.6
ATRN, P=NA, FC=0.95
AZGP1, P=0.0095, FC=2.1
B2M, P=NA, FC=3.8
BCHE, P=NA, FC=0.98
BTD, P=NA, FC=2.2
C1QA, P=NA, FC=1.1
C1QB, P=0.33, FC=1.7
C1QC, P=NA, FC=1.3
SS 3 4 5 6 7 GS

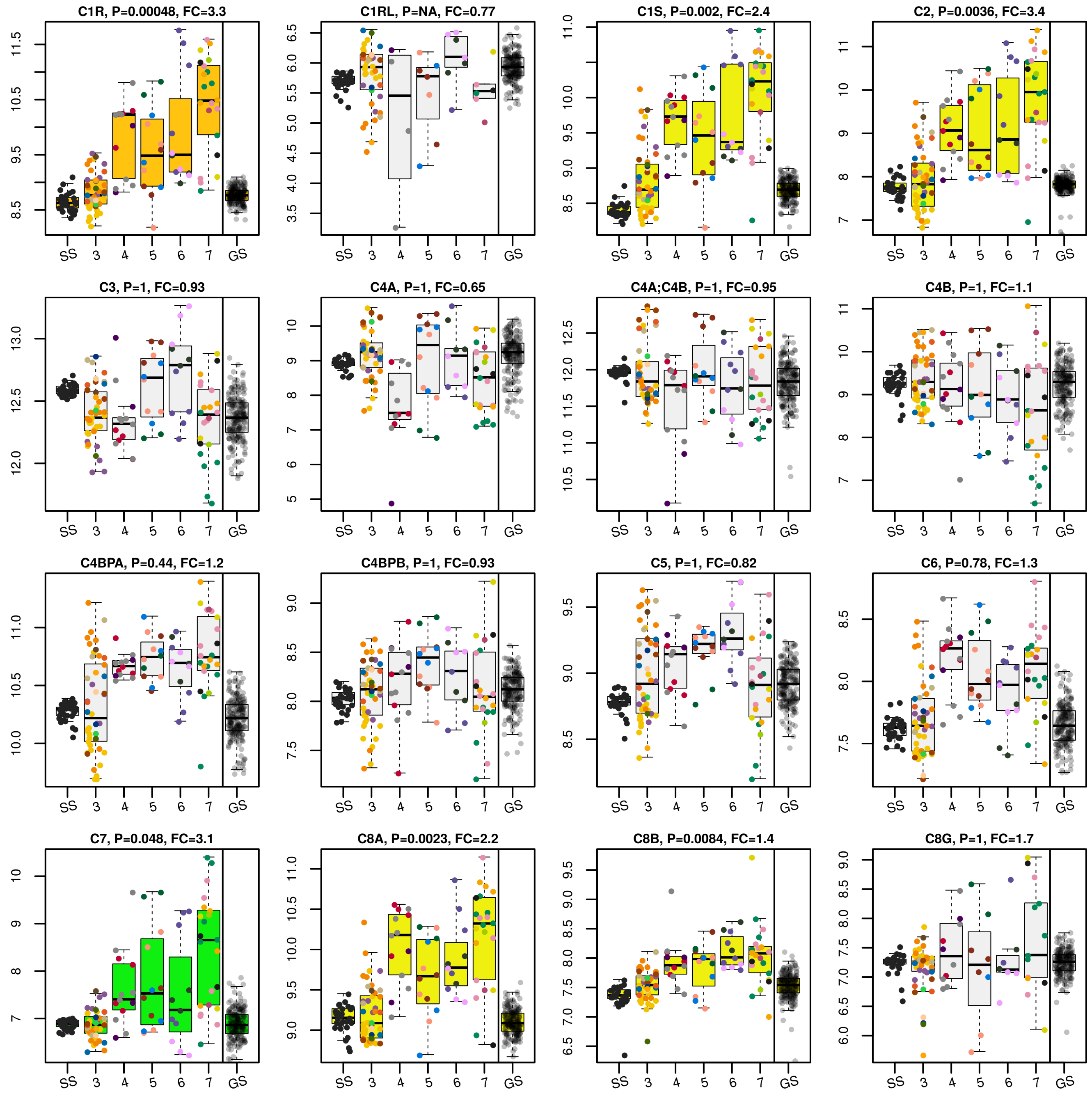
C1R, P=0.00048, FC=3.3
C1RL, P=NA, FC=0.77
C1S, P=0.002, FC=2.4
C2, P=0.0036, FC=3.4
C3, P=1, FC=0.93
C4A, P=1, FC=0.65
C4A;C4B, P=1, FC=0.95
C4B, P=1, FC=1.1
C4BPA, P=0.44, FC=1.2
C4BPB, P=1, FC=0.93
C5, P=1, FC=0.82
C6, P=0.78, FC=1.3
C7, P=0.048, FC=3.1
C8A, P=0.0023, FC=2.2
C8B, P=0.0084, FC=1.4
C8G, P=1, FC=1.7
SS 3 4 5 6 7 GS

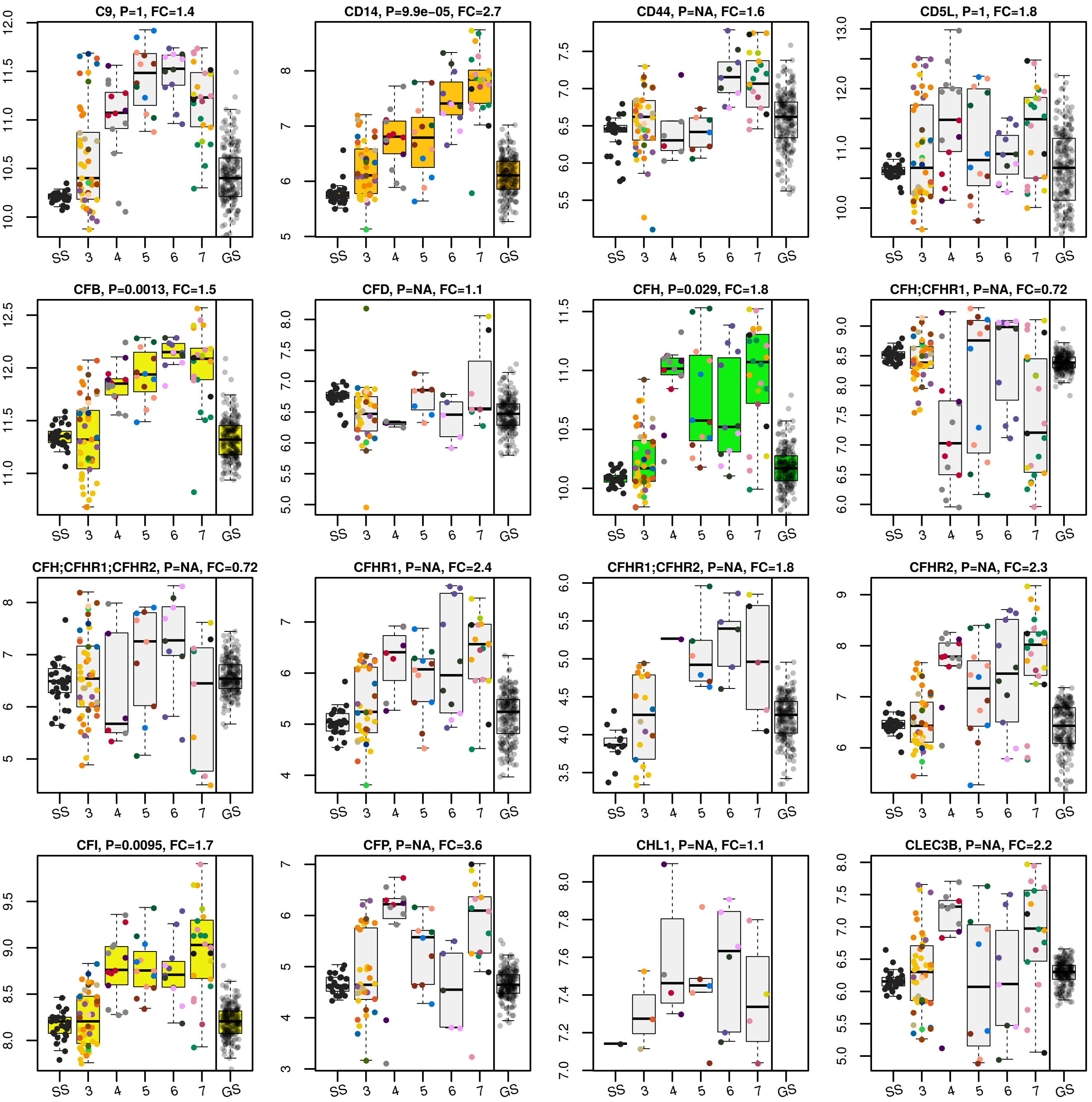
C9, P=1, FC=1.4
CD14, P=9.9e−05, FC=2.7
CD44, P=NA, FC=1.6
CD5L, P=1, FC=1.8
CFB, P=0.0013, FC=1.5
CFD, P=NA, FC=1.1
CFH, P=0.029, FC=1.8
CFH;CFHR1, P=NA, FC=0.72
CFH;CFHR1;CFHR2, P=NA, FC=0.72
CFHR1, P=NA, FC=2.4
CFHR1;CFHR2, P=NA, FC=1.8
CFHR2, P=NA, FC=2.3
CFI, P=0.0095, FC=1.7
CFP, P=NA, FC=3.6
CHL1, P=NA, FC=1.1
CLEC3B, P=NA, FC=2.2
SS 3 4 5 6 7 GS

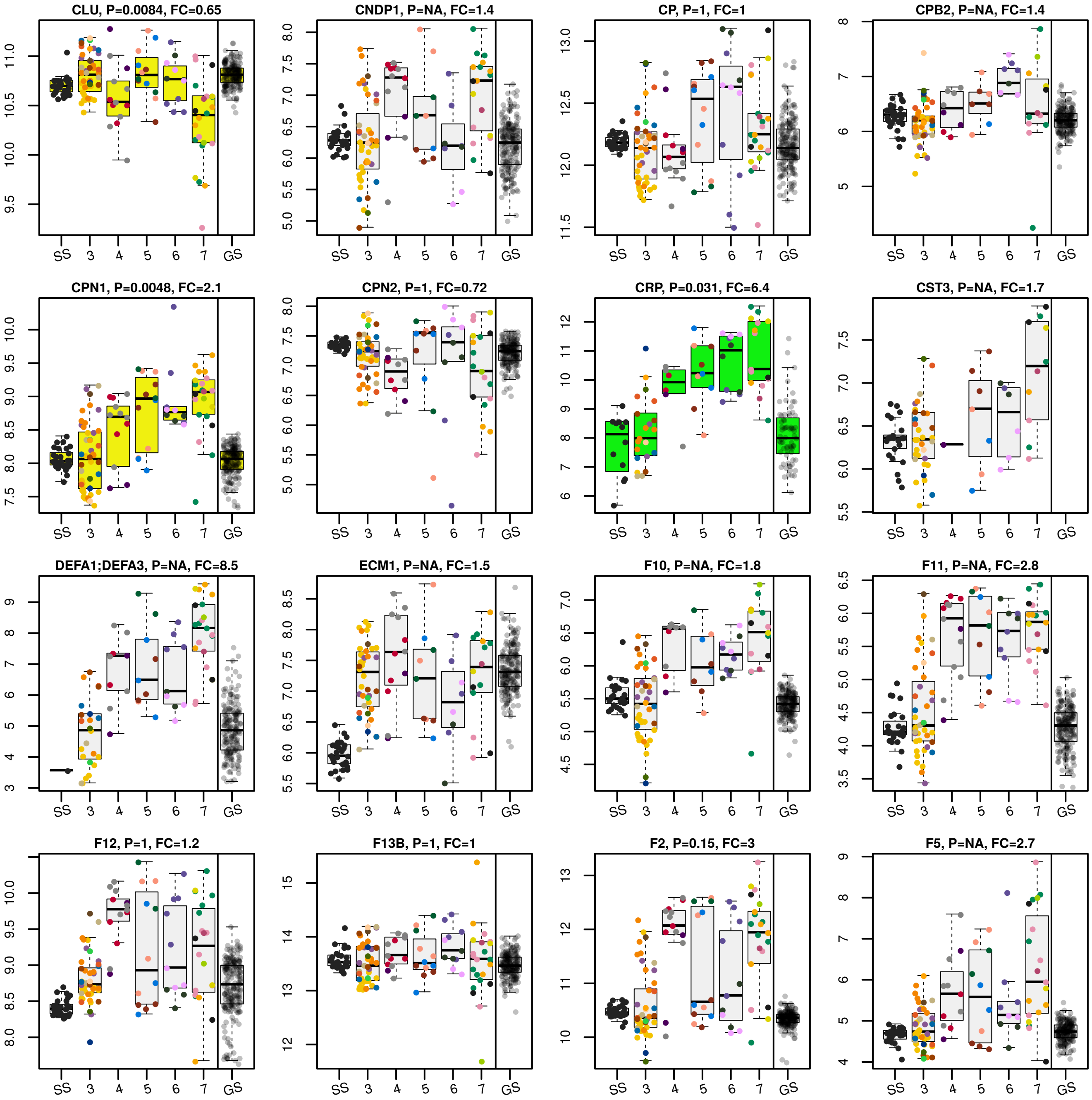

CLU, P=0.0084, FC=0.65
CNDP1, P=NA, FC=1.4
CP, P=1, FC=1
CPB2, P=NA, FC=1.4
CPN1, P=0.0048, FC=2.1
CPN2, P=1, FC=0.72
CRP, P=0.031, FC=6.4
CST3, P=NA, FC=1.7
DEFA1;DEFA3, P=NA, FC=8.5
ECM1, P=NA, FC=1.5
F10, P=NA, FC=1.8
F11, P=NA, FC=2.8
F12, P=1, FC=1.2
F13B, P=1, FC=1
F2, P=0.15, FC=3
F5, P=NA, FC=2.7
SS
3
4
5
6
7
GS

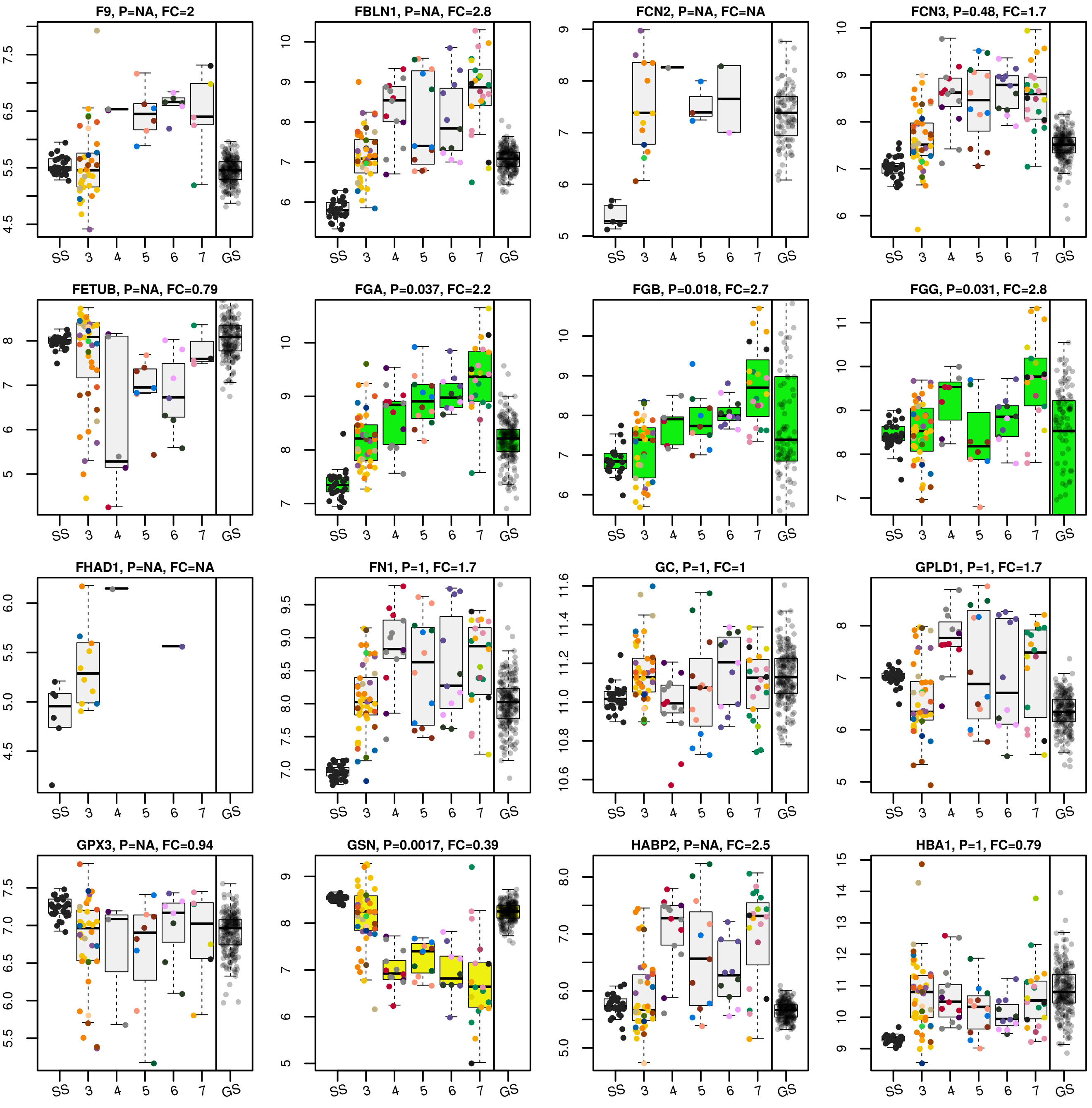

F9, P=NA, FC=2
FBLN1, P=NA, FC=2.8
FCN2, P=NA, FC=NA
FCN3, P=0.48, FC=1.7
FETUB, P=NA, FC=0.79
FGA, P=0.037, FC=2.2
FGB, P=0.018, FC=2.7
FGG, P=0.031, FC=2.8
FHAD1, P=NA, FC=NA
FN1, P=1, FC=1.7
GC, P=1, FC=1
GPLD1, P=1, FC=1.7
GPX3, P=NA, FC=0.94
GSN, P=0.0017, FC=0.39
HABP2, P=NA, FC=2.5
HBA1, P=1, FC=0.79
SS
3
4
5
6
7
GS

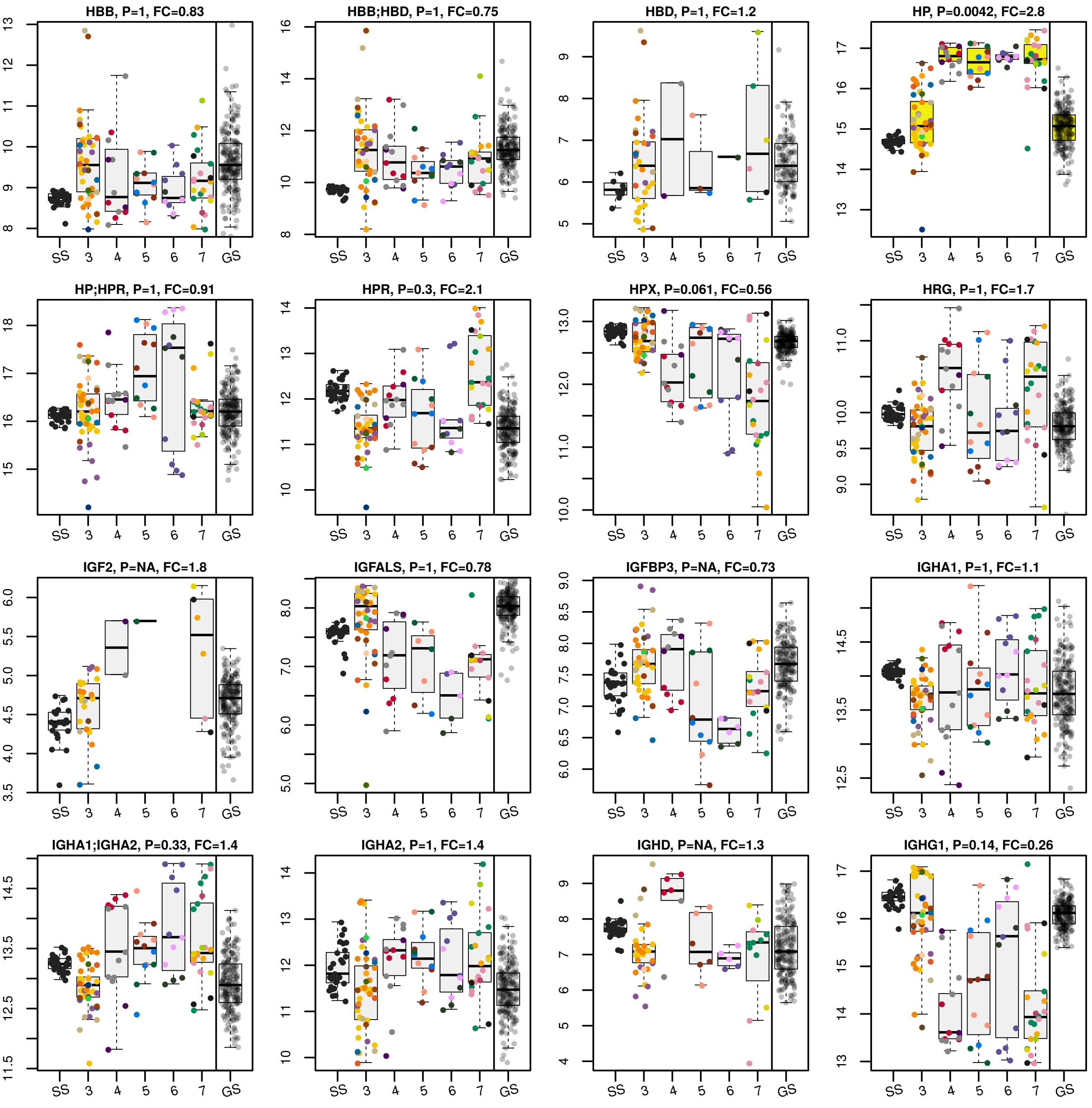
HBB, P=1, FC=0.83
HBB;HBD, P=1, FC=0.75
HBD, P=1, FC=1.2
HP, P=0.0042, FC=2.8
HP;HPR, P=1, FC=0.91
HPR, P=0.3, FC=2.1
HPX, P=0.061, FC=0.56
HRG, P=1, FC=1.7
IGF2, P=NA, FC=1.8
IGFALS, P=1, FC=0.78
IGFBP3, P=NA, FC=0.73
IGHA1, P=1, FC=1.1
IGHA1;IGHA2, P=0.33, FC=1.4
IGHA2, P=1, FC=1.4
IGHD, P=NA, FC=1.3
IGHG1, P=0.14, FC=0.26
SS
3
4
5
6
7
GS

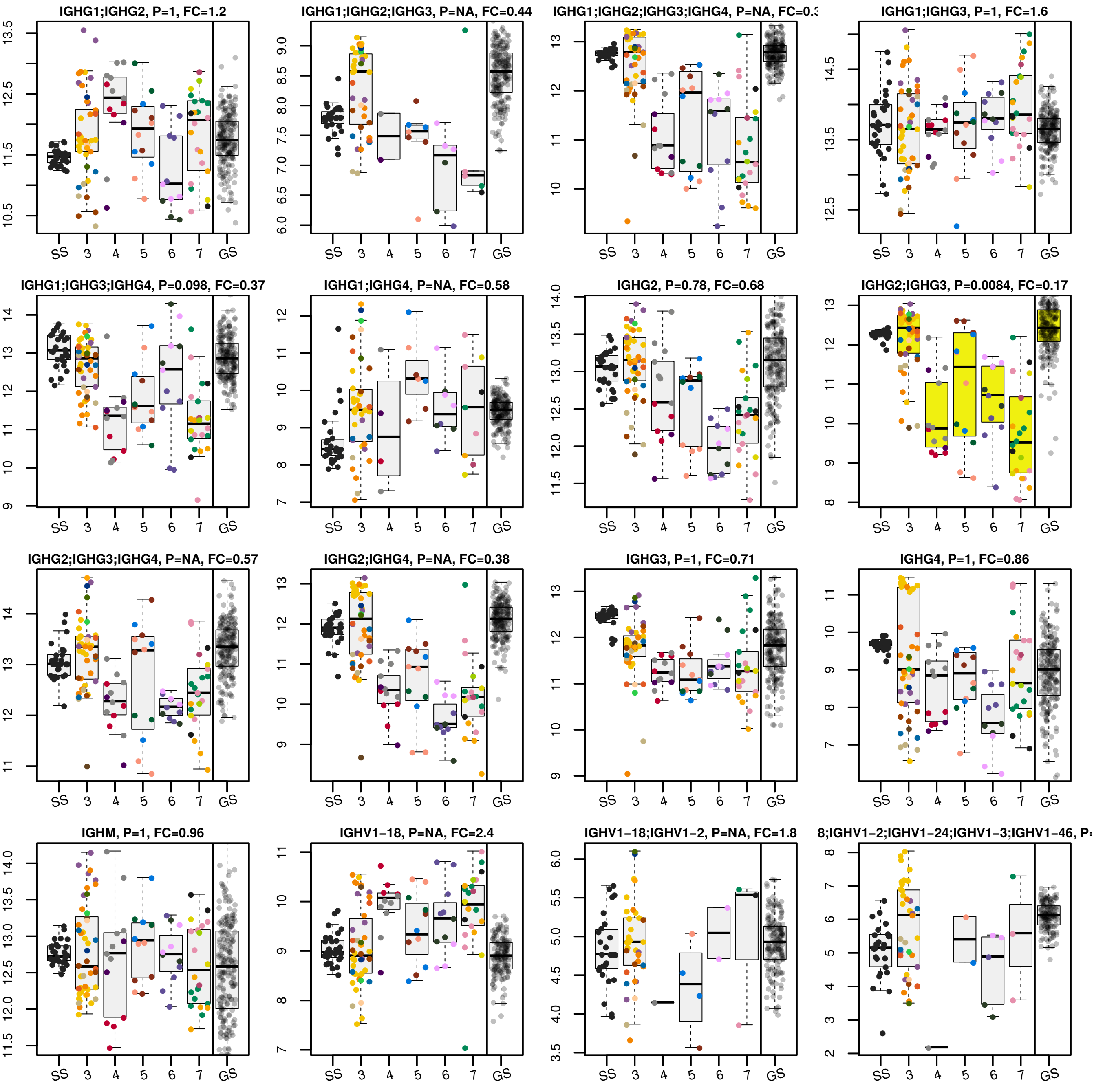
IGHG1;IGHG2, P=1, FC=1.2
IGHG1;IGHG2;IGHG3, P=NA, FC=0.44
IGHG1;IGHG2;IGHG3;IGHG4, P=NA, FC=0.3
IGHG1;IGHG3, P=1, FC=1.6
IGHG1;IGHG3;IGHG4, P=0.098, FC=0.37
IGHG1;IGHG4, P=NA, FC=0.58
IGHG2, P=0.78, FC=0.68
IGHG2;IGHG3, P=0.0084, FC=0.17
IGHG2;IGHG3;IGHG4, P=NA, FC=0.57
IGHG2;IGHG4, P=NA, FC=0.38
IGHG3, P=1, FC=0.71
IGHG4, P=1, FC=0.86
IGHM, P=1, FC=0.96
IGHV1−18, P=NA, FC=2.4
IGHV1−18;IGHV1−2, P=NA, FC=1.8
8;IGHV1−2;IGHV1−24;IGHV1−3;IGHV1−46, P=
SS 3 4 5 6 7 GS

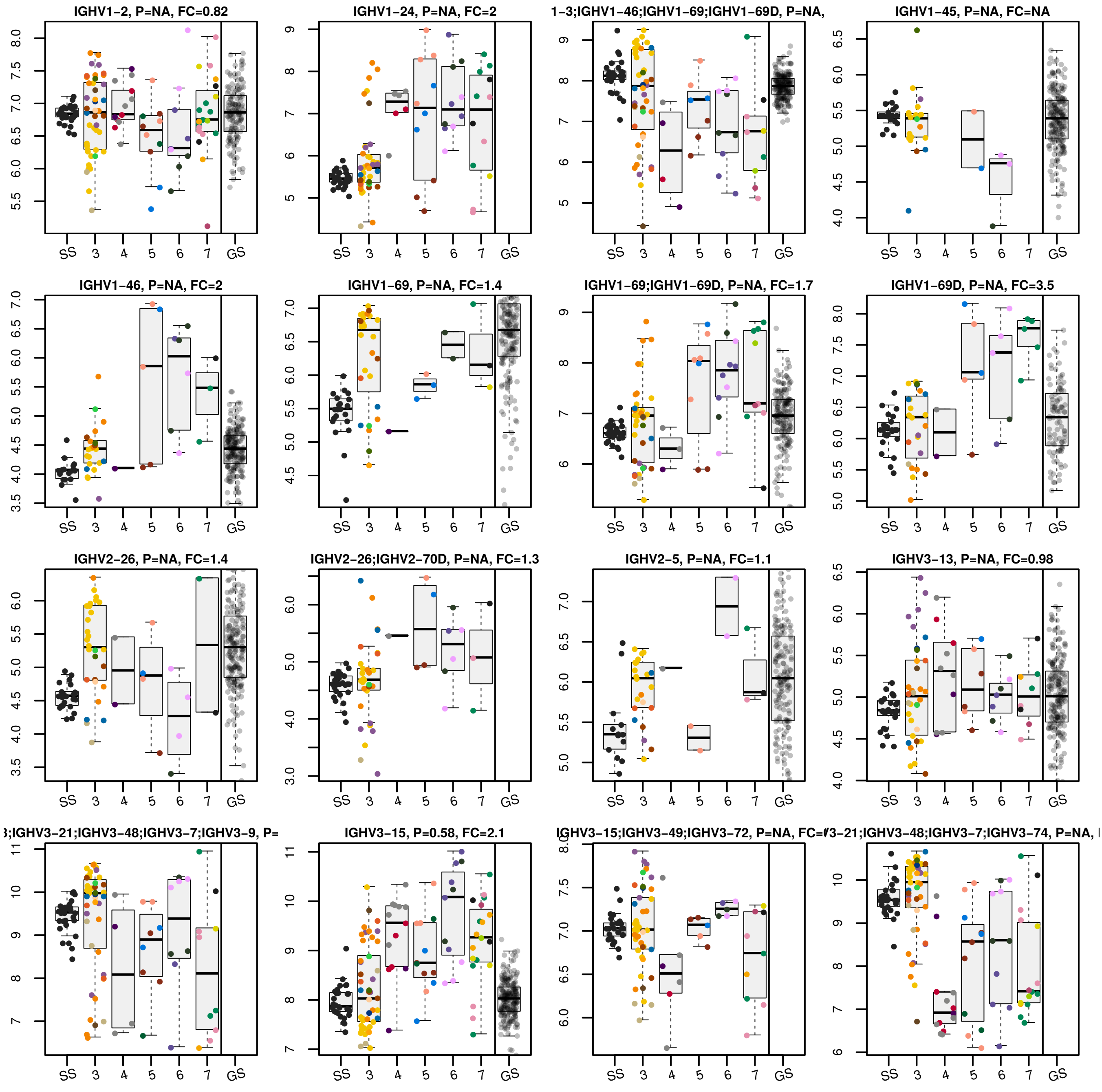

IGHV1−2, P=NA, FC=0.82
SS 3 4 5 6 7 GS
IGHV1−24, P=NA, FC=2
SS 3 4 5 6 7 GS
1−3;IGHV1−46;IGHV1−69;IGHV1−69D, P=NA,
SS 3 4 5 6 7 GS
IGHV1−45, P=NA, FC=NA
SS 3 4 5 6 7 GS
IGHV1−46, P=NA, FC=2
SS 3 4 5 6 7 GS
IGHV1−69, P=NA, FC=1.4
SS 3 4 5 6 7 GS
IGHV1−69;IGHV1−69D, P=NA, FC=1.7
SS 3 4 5 6 7 GS
IGHV1−69D, P=NA, FC=3.5
SS 3 4 5 6 7 GS
IGHV2−26, P=NA, FC=1.4
SS 3 4 5 6 7 GS
IGHV2−26;IGHV2−70D, P=NA, FC=1.3
SS 3 4 5 6 7 GS
IGHV2−5, P=NA, FC=1.1
SS 3 4 5 6 7 GS
IGHV3−13, P=NA, FC=0.98
SS 3 4 5 6 7 GS
3;IGHV3−21;IGHV3−48;IGHV3−7;IGHV3−9, P=
SS 3 4 5 6 7 GS
IGHV3−15, P=0.58, FC=2.1
SS 3 4 5 6 7 GS
IGHV3−15;IGHV3−49;IGHV3−72, P=NA, FC=
SS 3 4 5 6 7 GS
/3−21;IGHV3−48;IGHV3−7;IGHV3−74, P=NA, I
SS 3 4 5 6 7 GS

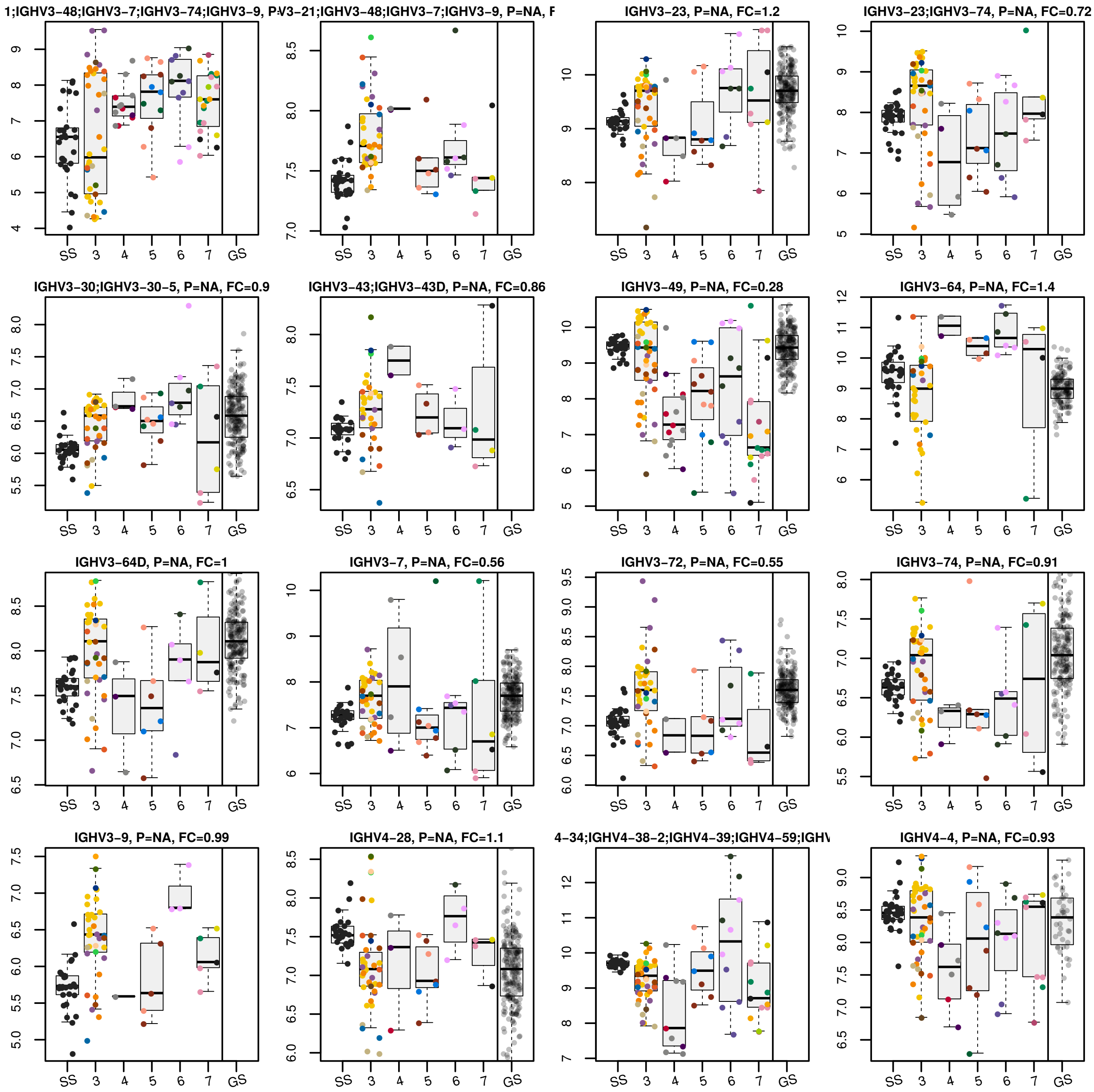
1;IGHV3−48;IGHV3−7;IGHV3−74;IGHV3−9, P
V3−21;IGHV3−48;IGHV3−7;IGHV3−9, P=NA, F
IGHV3−23, P=NA, FC=1.2
IGHV3−23;IGHV3−74, P=NA, FC=0.72
IGHV3−30;IGHV3−30−5, P=NA, FC=0.9
IGHV3−43;IGHV3−43D, P=NA, FC=0.86
IGHV3−49, P=NA, FC=0.28
IGHV3−64, P=NA, FC=1.4
IGHV3−64D, P=NA, FC=1
IGHV3−7, P=NA, FC=0.56
IGHV3−72, P=NA, FC=0.55
IGHV3−74, P=NA, FC=0.91
IGHV3−9, P=NA, FC=0.99
IGHV4−28, P=NA, FC=1.1
4−34;IGHV4−38−2;IGHV4−39;IGHV4−59;IGHV
IGHV4−4, P=NA, FC=0.93
SS 3 4 5 6 7 GS

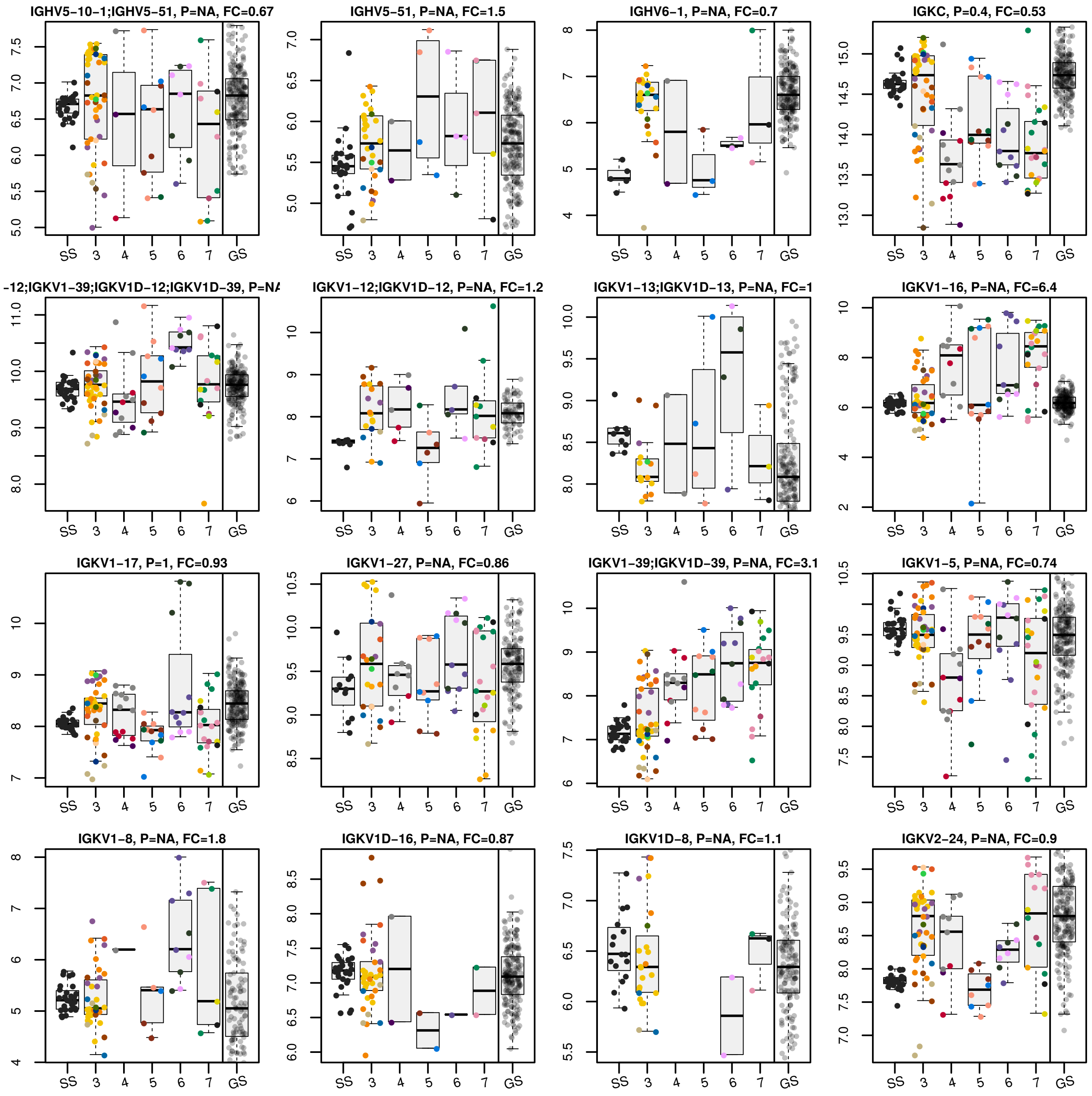
IGHV5−10−1;IGHV5−51, P=NA, FC=0.67
IGHV5−51, P=NA, FC=1.5
IGHV6−1, P=NA, FC=0.7
IGKC, P=0.4, FC=0.53
−12;IGKV1−39;IGKV1D−12;IGKV1D−39, P=NA
IGKV1−12;IGKV1D−12, P=NA, FC=1.2
IGKV1−13;IGKV1D−13, P=NA, FC=1
IGKV1−16, P=NA, FC=6.4
IGKV1−17, P=1, FC=0.93
IGKV1−27, P=NA, FC=0.86
IGKV1−39;IGKV1D−39, P=NA, FC=3.1
IGKV1−5, P=NA, FC=0.74
IGKV1−8, P=NA, FC=1.8
IGKV1D−16, P=NA, FC=0.87
IGKV1D−8, P=NA, FC=1.1
IGKV2−24, P=NA, FC=0.9
SS 3 4 5 6 7 GS

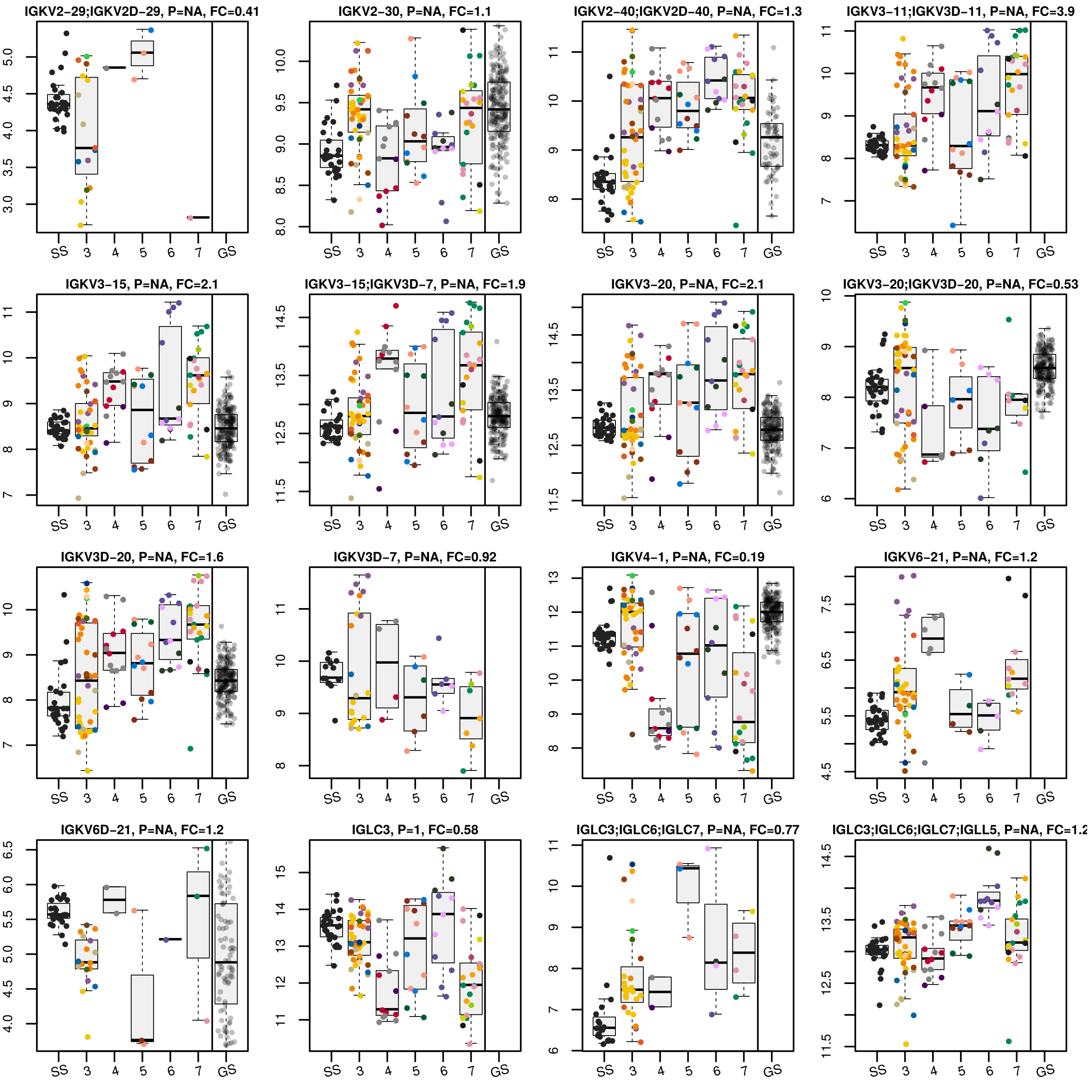
IGKV2−29;IGKV2D−29, P=NA, FC=0.41
IGKV2−30, P=NA, FC=1.1
IGKV2−40;IGKV2D−40, P=NA, FC=1.3
IGKV3−11;IGKV3D−11, P=NA, FC=3.9
IGKV3−15, P=NA, FC=2.1
IGKV3−15;IGKV3D−7, P=NA, FC=1.9
IGKV3−20, P=NA, FC=2.1
IGKV3−20;IGKV3D−20, P=NA, FC=0.53
IGKV3D−20, P=NA, FC=1.6
IGKV3D−7, P=NA, FC=0.92
IGKV4−1, P=NA, FC=0.19
IGKV6−21, P=NA, FC=1.2
IGKV6D−21, P=NA, FC=1.2
IGLC3, P=1, FC=0.58
IGLC3;IGLC6;IGLC7, P=NA, FC=0.77
IGLC3;IGLC6;IGLC7;IGLL5, P=NA, FC=1.2
SS 3 4 5 6 7 GS

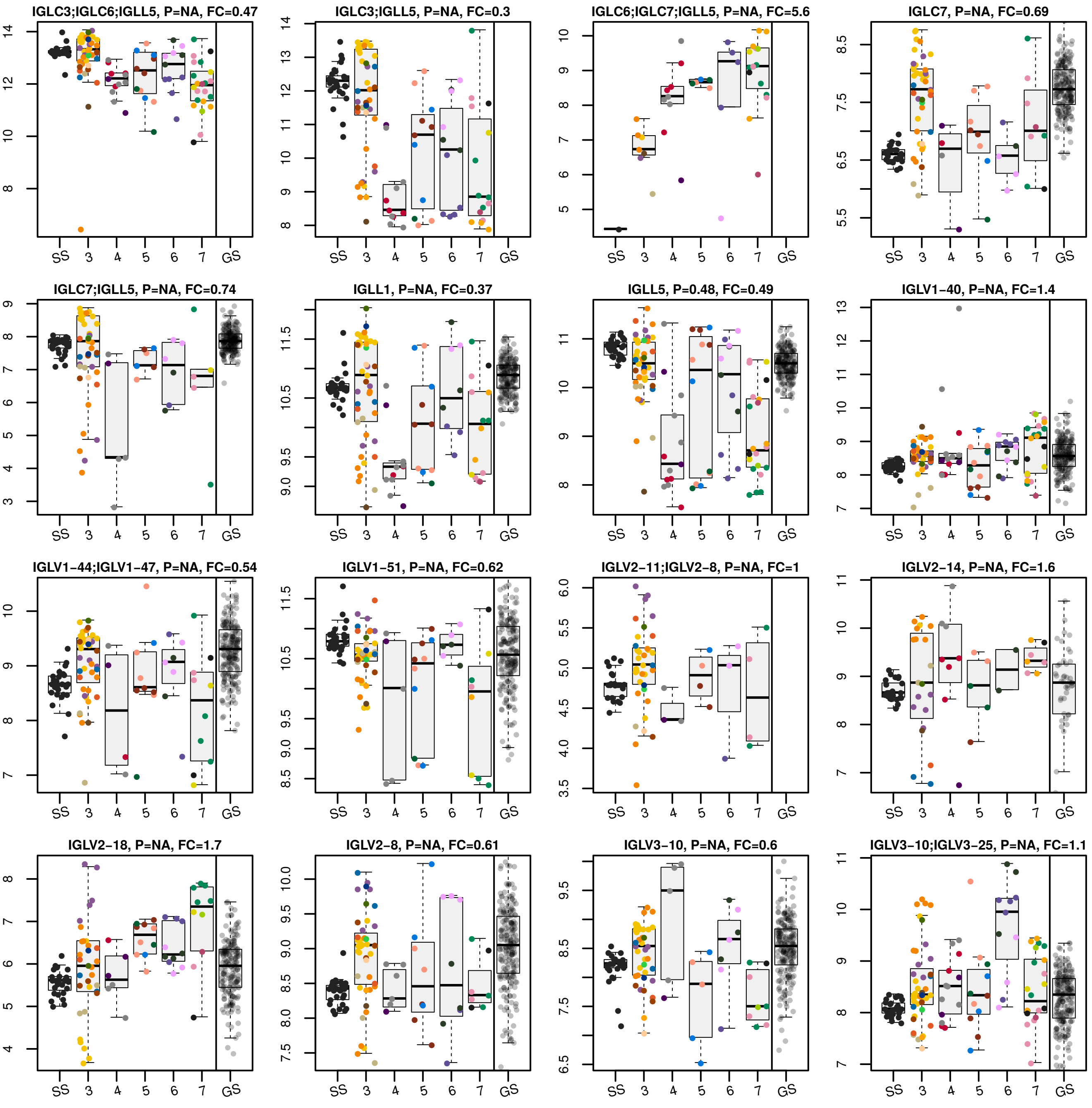
IGLC3;IGLC6;IGLL5, P=NA, FC=0.47
IGLC3;IGLL5, P=NA, FC=0.3
IGLC6;IGLC7;IGLL5, P=NA, FC=5.6
IGLC7, P=NA, FC=0.69
IGLC7;IGLL5, P=NA, FC=0.74
IGLL1, P=NA, FC=0.37
IGLL5, P=0.48, FC=0.49
IGLV1−40, P=NA, FC=1.4
IGLV1−44;IGLV1−47, P=NA, FC=0.54
IGLV1−51, P=NA, FC=0.62
IGLV2−11;IGLV2−8, P=NA, FC=1
IGLV2−14, P=NA, FC=1.6
IGLV2−18, P=NA, FC=1.7
IGLV2−8, P=NA, FC=0.61
IGLV3−10, P=NA, FC=0.6
IGLV3−10;IGLV3−25, P=NA, FC=1.1
SS 3 4 5 6 7 GS

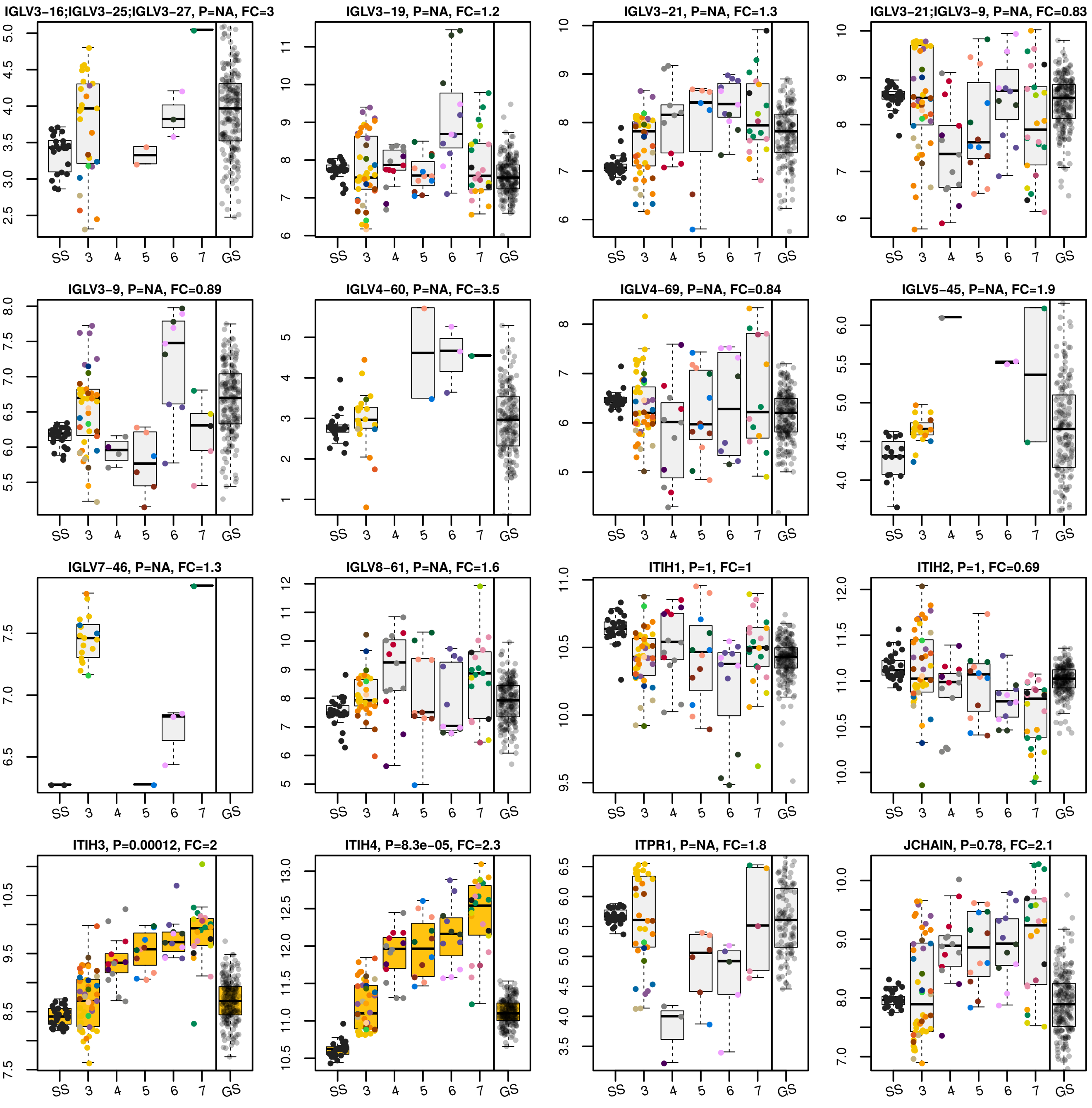
IGLV3−16;IGLV3−25;IGLV3−27, P=NA, FC=3
IGLV3−19, P=NA, FC=1.2
IGLV3−21, P=NA, FC=1.3
IGLV3−21;IGLV3−9, P=NA, FC=0.83
IGLV3−9, P=NA, FC=0.89
IGLV4−60, P=NA, FC=3.5
IGLV4−69, P=NA, FC=0.84
IGLV5−45, P=NA, FC=1.9
IGLV7−46, P=NA, FC=1.3
IGLV8−61, P=NA, FC=1.6
ITIH1, P=1, FC=1
ITIH2, P=1, FC=0.69
ITIH3, P=0.00012, FC=2
ITIH4, P=8.3e−05, FC=2.3
ITPR1, P=NA, FC=1.8
JCHAIN, P=0.78, FC=2.1
SS 3 4 5 6 7 GS

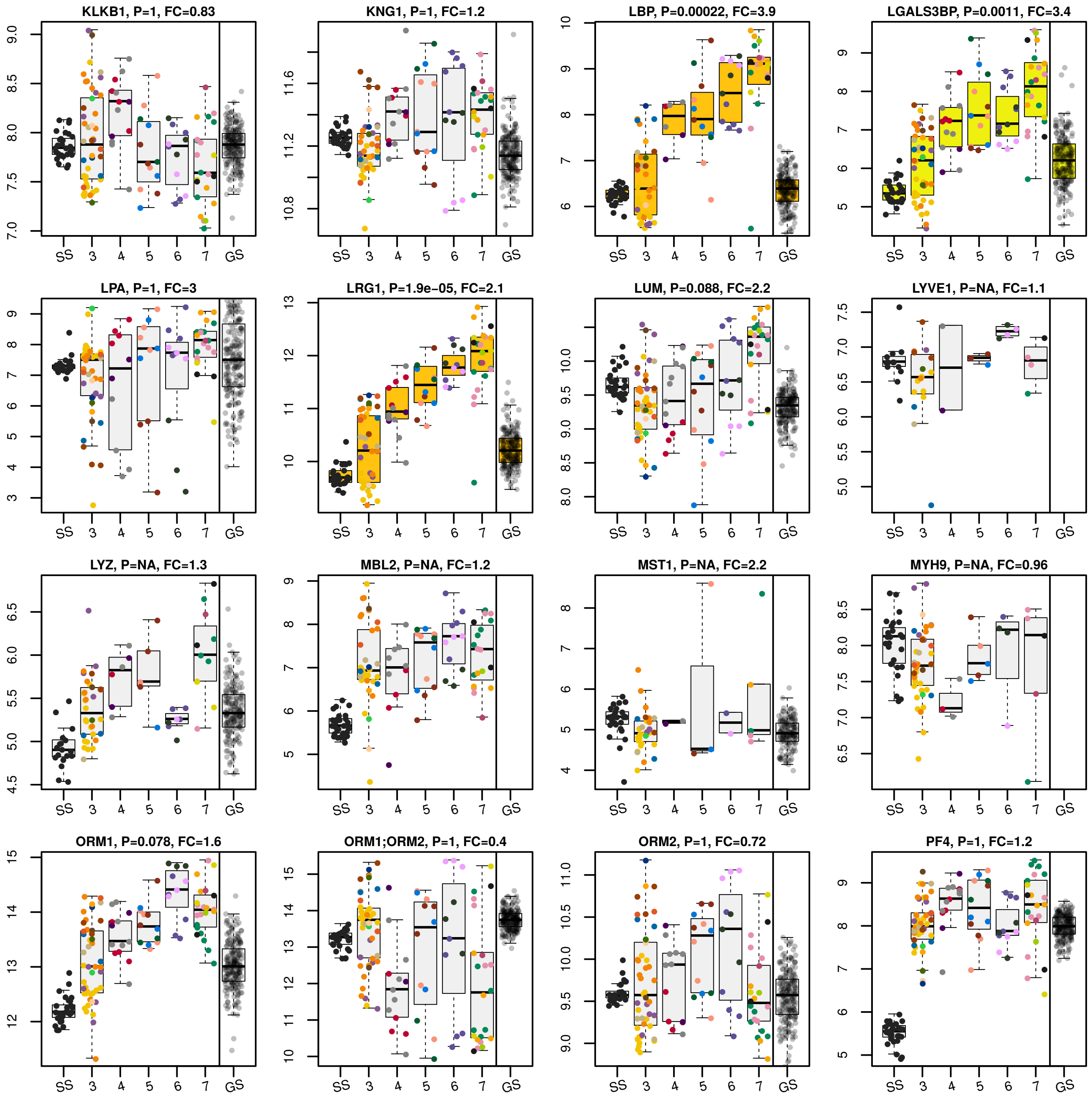
KLKB1, P=1, FC=0.83
KNG1, P=1, FC=1.2
LBP, P=0.00022, FC=3.9
LGALS3BP, P=0.0011, FC=3.4
LPA, P=1, FC=3
LRG1, P=1.9e−05, FC=2.1
LUM, P=0.088, FC=2.2
LYVE1, P=NA, FC=1.1
LYZ, P=NA, FC=1.3
MBL2, P=NA, FC=1.2
MST1, P=NA, FC=2.2
MYH9, P=NA, FC=0.96
ORM1, P=0.078, FC=1.6
ORM1;ORM2, P=1, FC=0.4
ORM2, P=1, FC=0.72
PF4, P=1, FC=1.2
SS 3 4 5 6 7 GS

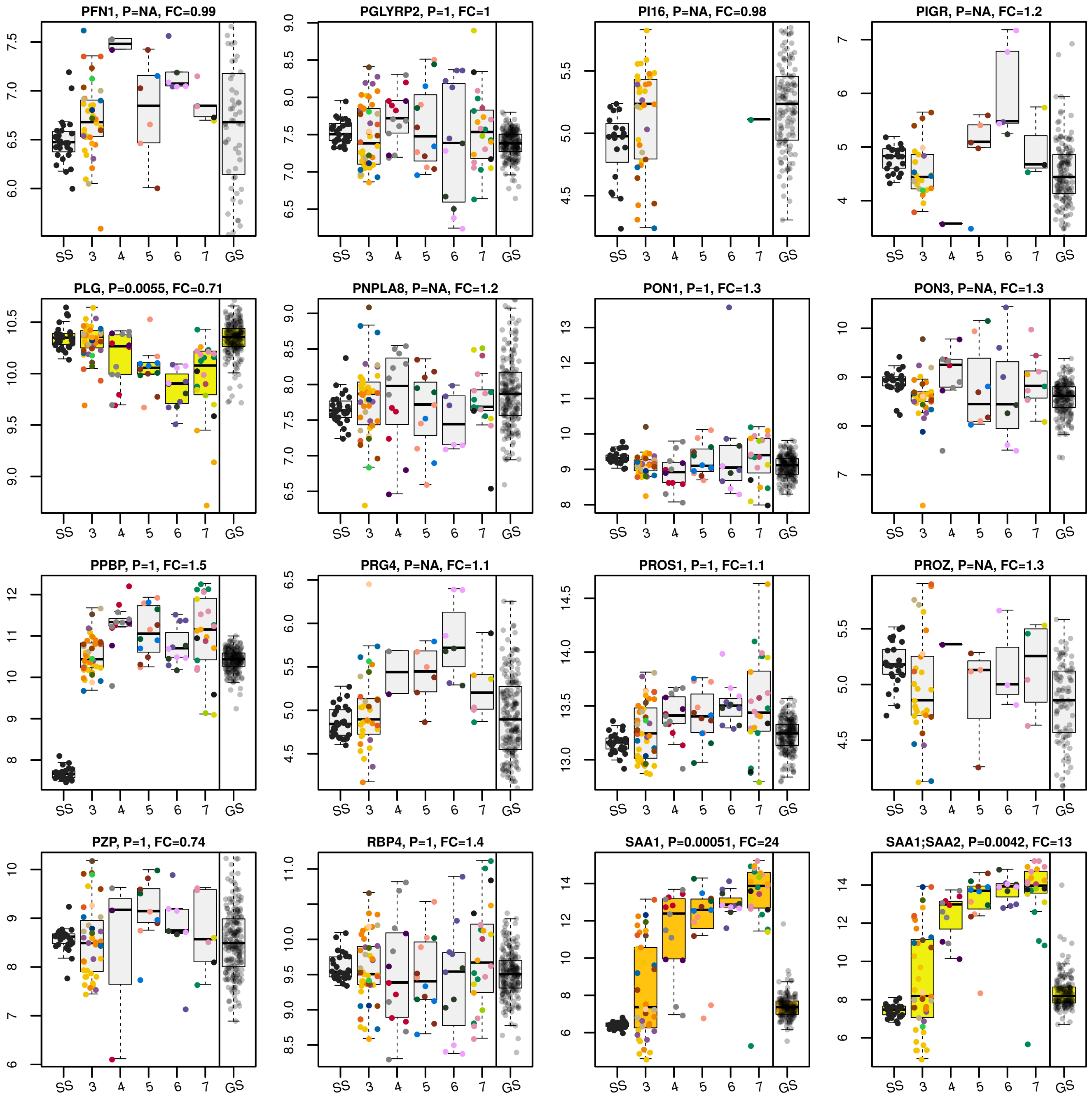
PFN1, P=NA, FC=0.99
PGLYRP2, P=1, FC=1
PI16, P=NA, FC=0.98
PIGR, P=NA, FC=1.2
PLG, P=0.0055, FC=0.71
PNPLA8, P=NA, FC=1.2
PON1, P=1, FC=1.3
PON3, P=NA, FC=1.3
PPBP, P=1, FC=1.5
PRG4, P=NA, FC=1.1
PROS1, P=1, FC=1.1
PROZ, P=NA, FC=1.3
PZP, P=1, FC=0.74
RBP4, P=1, FC=1.4
SAA1, P=0.00051, FC=24
SAA1;SAA2, P=0.0042, FC=13
SS
3
4
5
6
7
GS

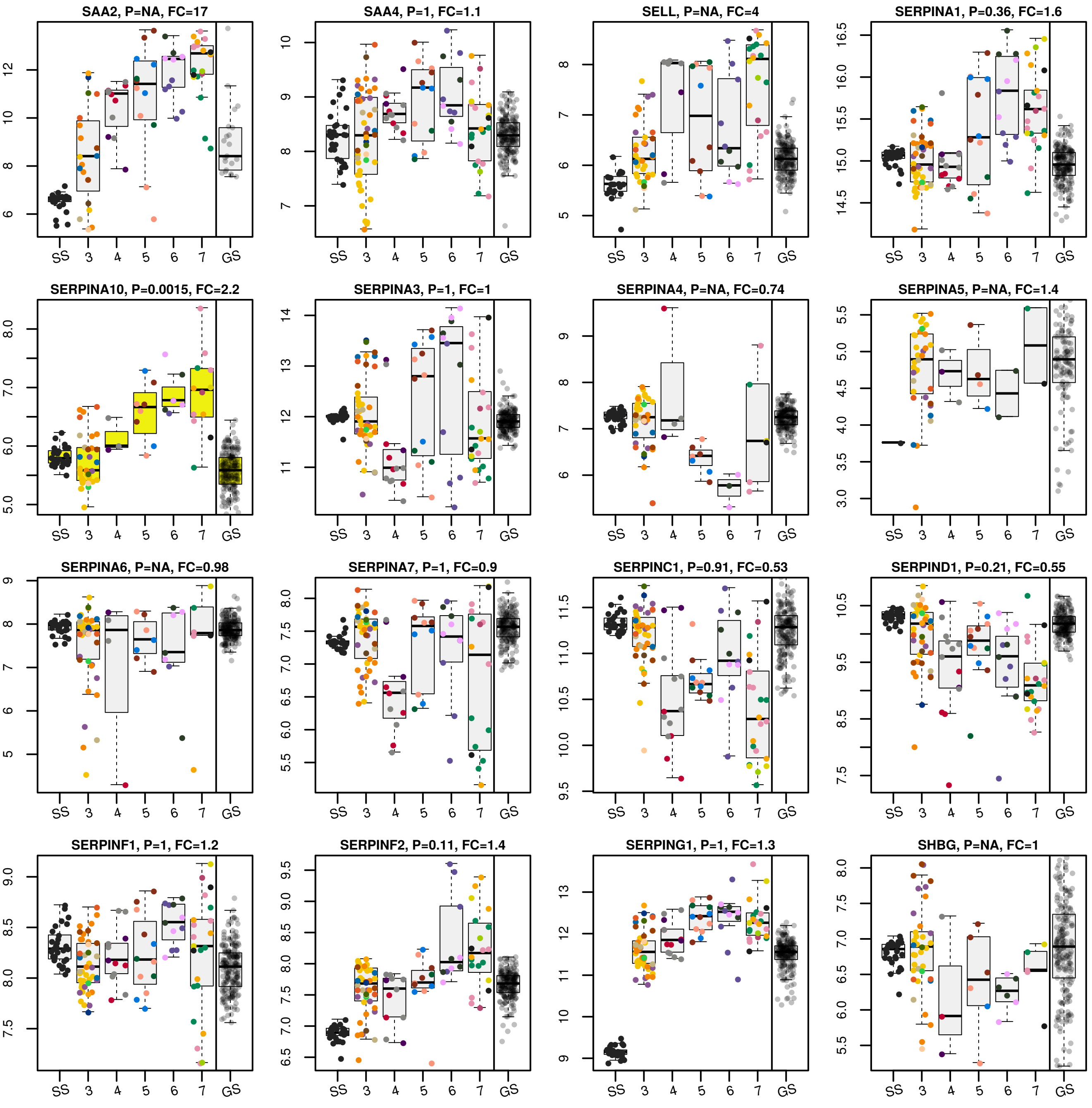
SAA2, P=NA, FC=17
SAA4, P=1, FC=1.1
SELL, P=NA, FC=4
SERPINA1, P=0.36, FC=1.6
SERPINA10, P=0.0015, FC=2.2
SERPINA3, P=1, FC=1
SERPINA4, P=NA, FC=0.74
SERPINA5, P=NA, FC=1.4
SERPINA6, P=NA, FC=0.98
SERPINA7, P=1, FC=0.9
SERPINC1, P=0.91, FC=0.53
SERPIND1, P=0.21, FC=0.55
SERPINF1, P=1, FC=1.2
SERPINF2, P=0.11, FC=1.4
SERPING1, P=1, FC=1.3
SHBG, P=NA, FC=1
SS 3 4 5 6 7 GS

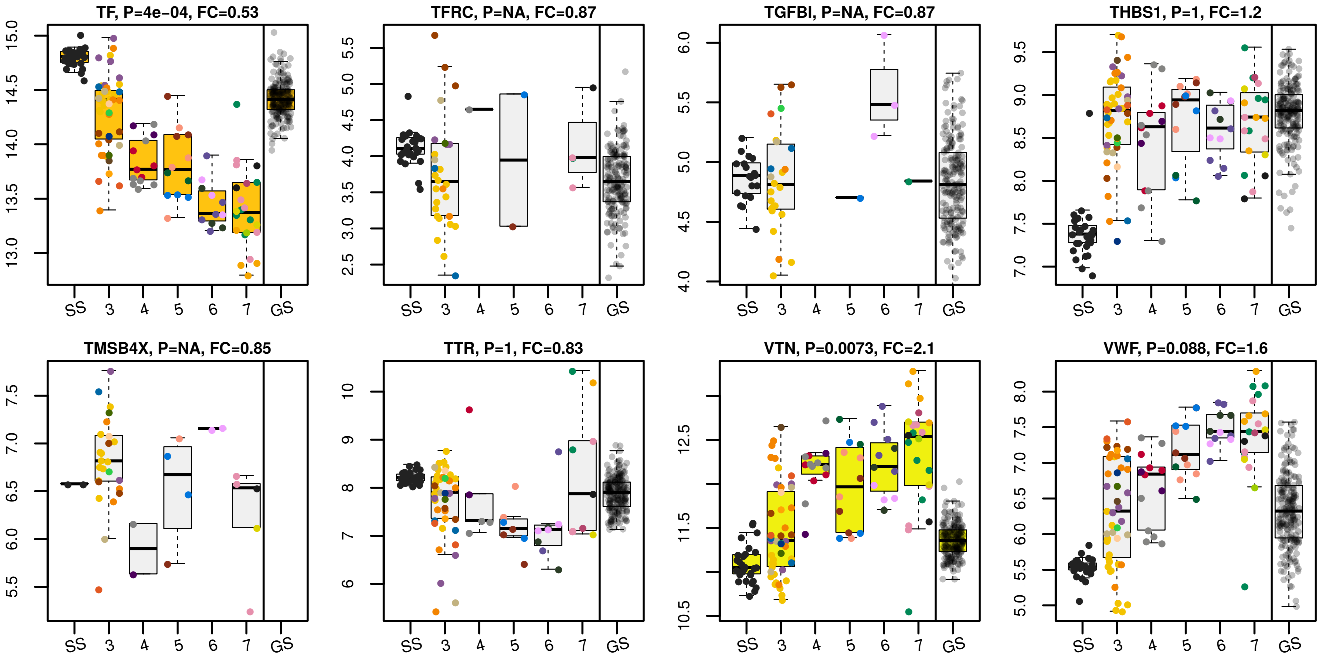

TF, P=4e−04, FC=0.53
TFRC, P=NA, FC=0.87
TGFBI, P=NA, FC=0.87
THBS1, P=1, FC=1.2
TMSB4X, P=NA, FC=0.85
TTR, P=1, FC=0.83
VTN, P=0.0073, FC=2.1
VWF, P=0.088, FC=1.6
SS 3 4 5 6 7 GS

Figure S8 (following 15 pages). **Age-related protein changes in the general population (GS cohort)**.

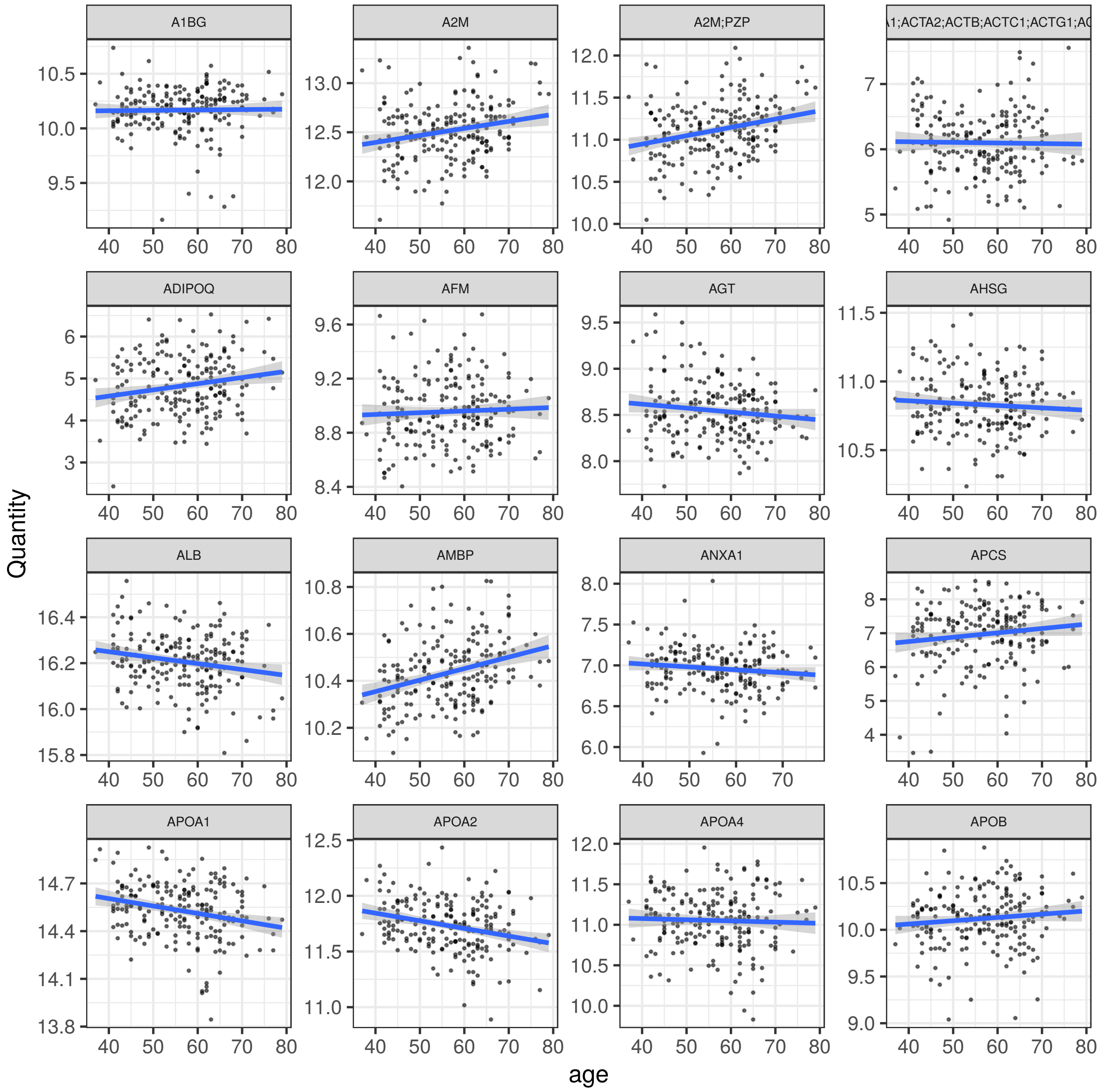
A1BG
A2M
A2M;PZP
A1;ACTA2;ACTB;ACTC1;ACTG1;AC
ADIPOQ
AFM
AGT
AHSG
ALB
AMBP
ANXA1
APCS
APOA1
APOA2
APOA4
APOB
Quantity
age

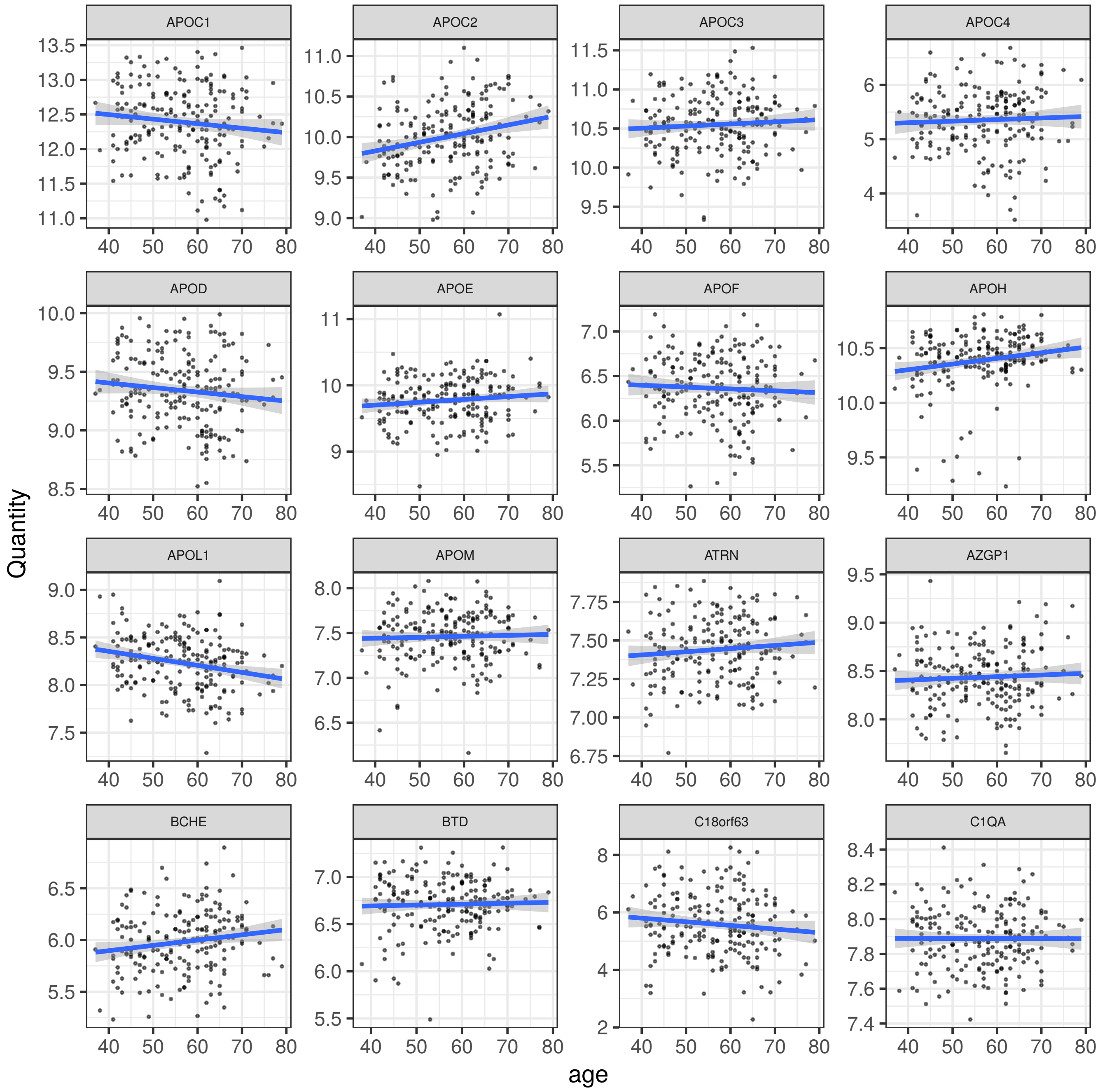
APOC1
APOC2
APOC3
APOC4
APOD
APOE
APOF
APOH
APOL1
APOM
ATRN
AZGP1
BCHE
BTD
C18orf63
C1QA
Quantity
age

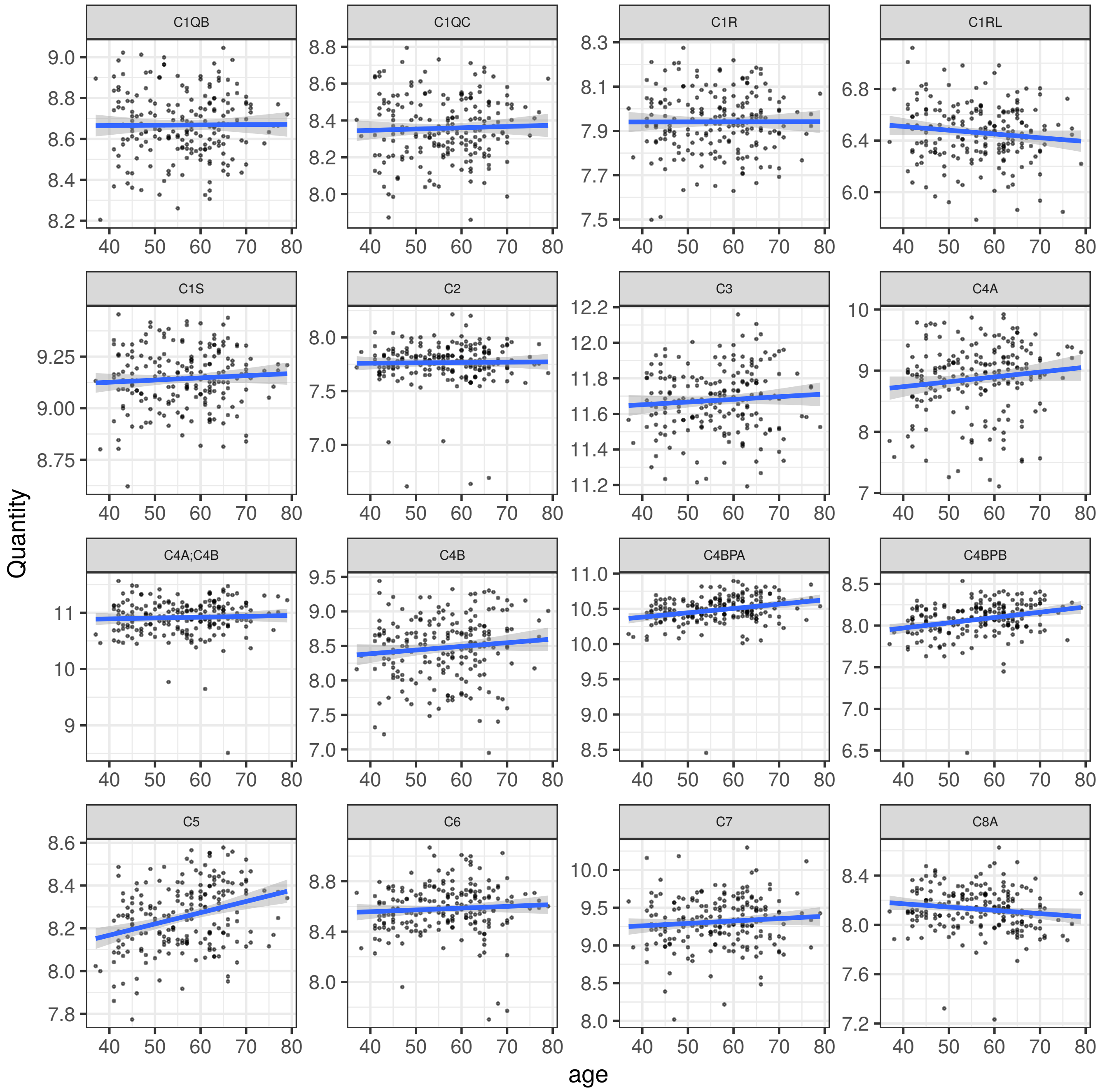
C1QB
C1QC
C1R
C1RL
C1S
C2
C3
C4A
C4A;C4B
C4B
C4BPA
C4BPB
C5
C6
C7
C8A
Quantity
age

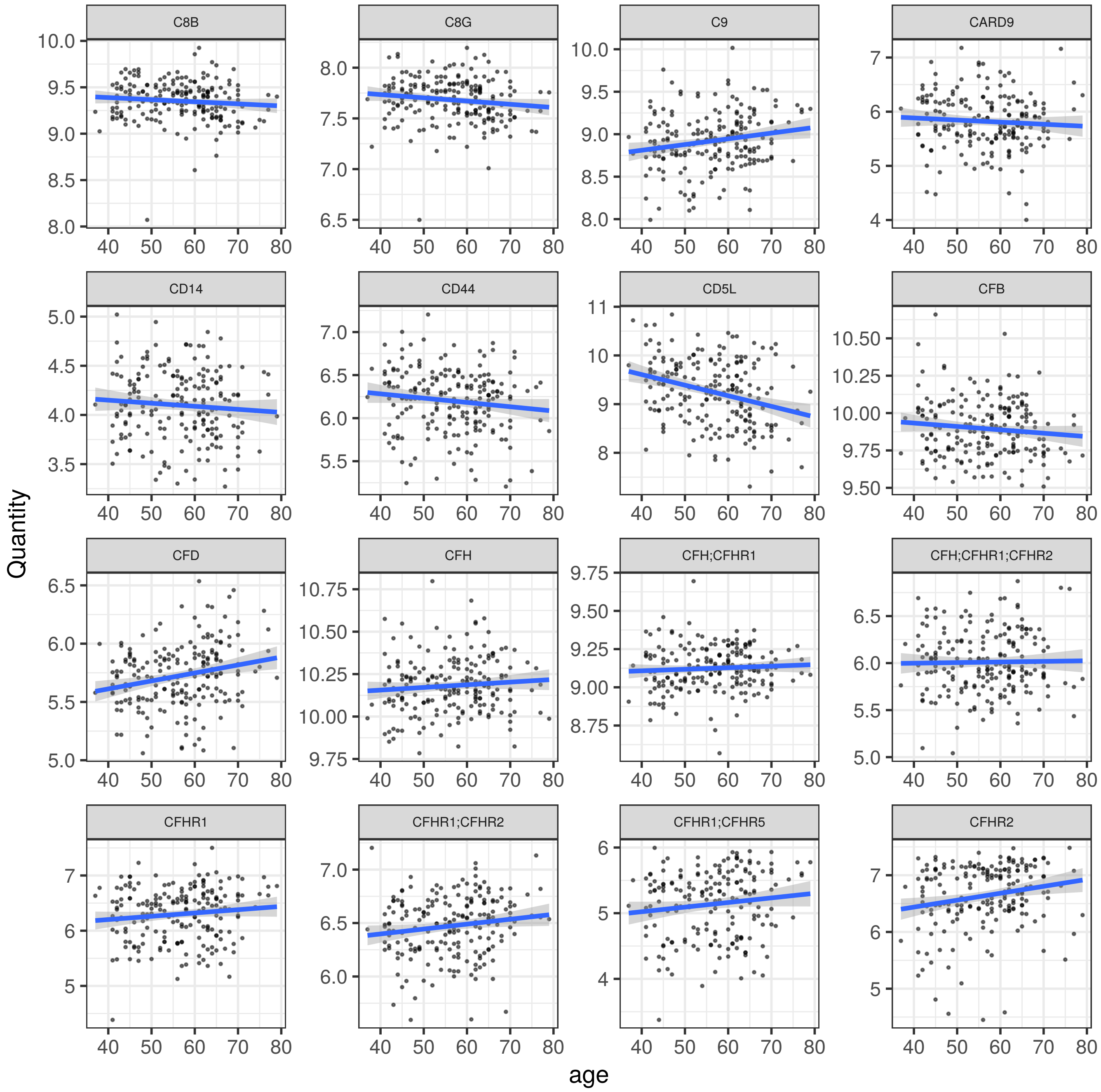

C8B
C8G
C9
CARD9
CD14
CD44
CD5L
CFB
CFD
CFH
CFH;CFHR1
CFH;CFHR1;CFHR2
CFHR1
CFHR1;CFHR2
CFHR1;CFHR5
CFHR2
Quantity
age

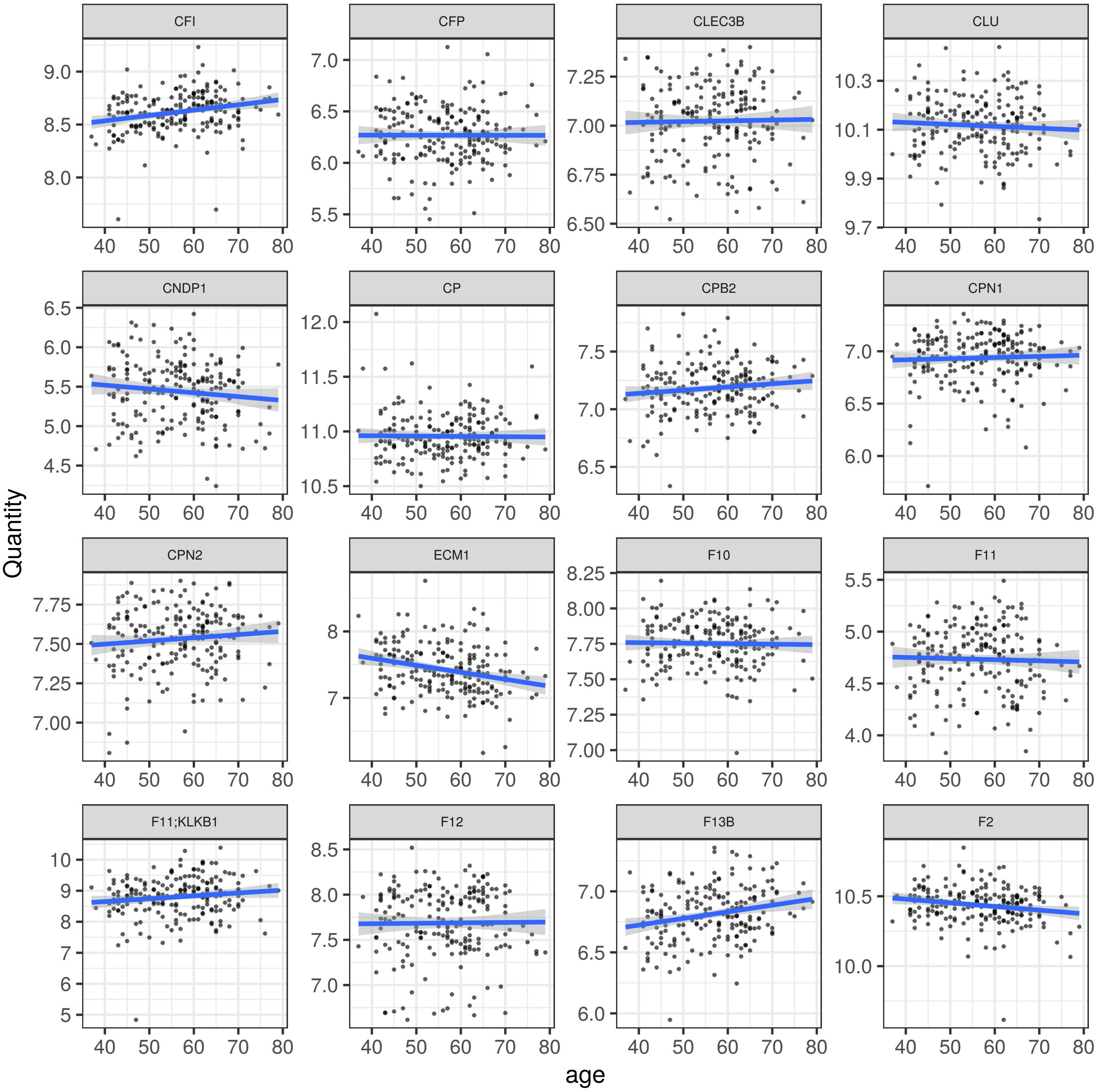

CFI
CFP
CLEC3B
CLU
CNDP1
CP
CPB2
CPN1
CPN2
ECM1
F10
F11
F11;KLKB1
F12
F13B
F2
Quantity
age

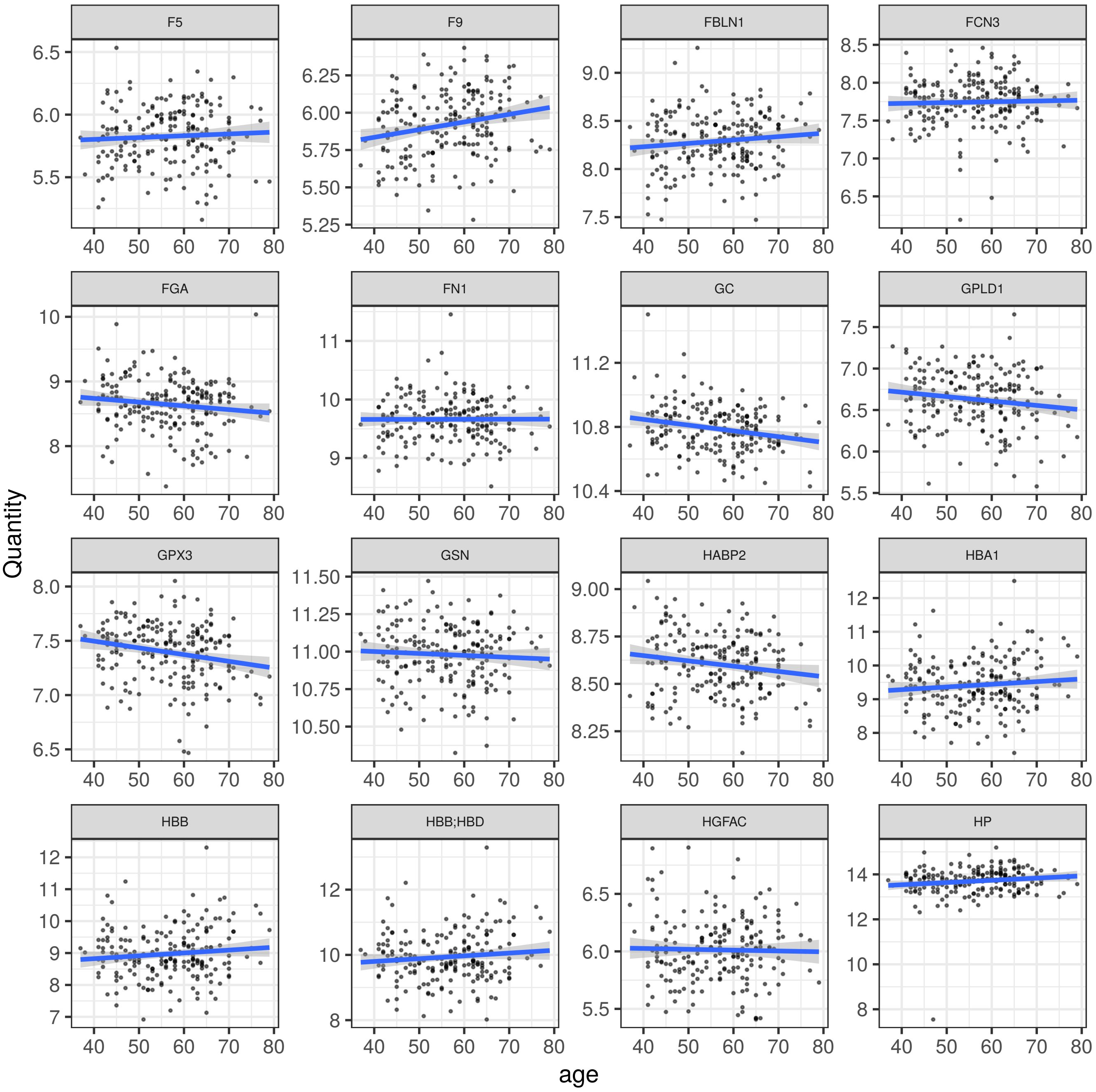
F5
F9
FBLN1
FCN3
FGA
FN1
GC
GPLD1
GPX3
GSN
HABP2
HBA1
HBB
HBB;HBD
HGFAC
HP
Quantity
age

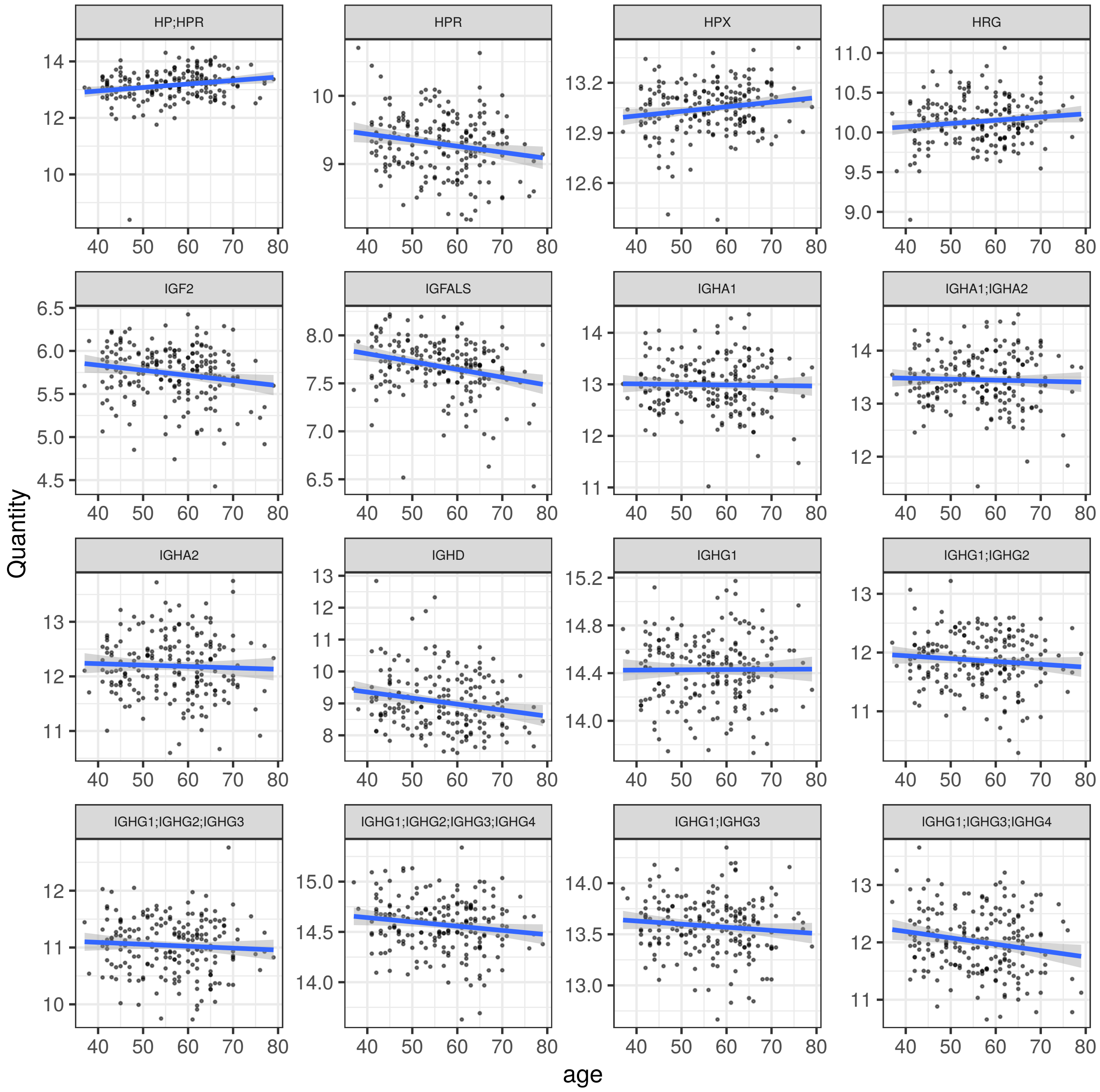
HP;HPR
HPR
HPX
HRG
IGF2
IGFALS
IGHA1
IGHA1;IGHA2
IGHA2
IGHD
IGHG1
IGHG1;IGHG2
IGHG1;IGHG2;IGHG3
IGHG1;IGHG2;IGHG3;IGHG4
IGHG1;IGHG3
IGHG1;IGHG3;IGHG4
Quantity
age

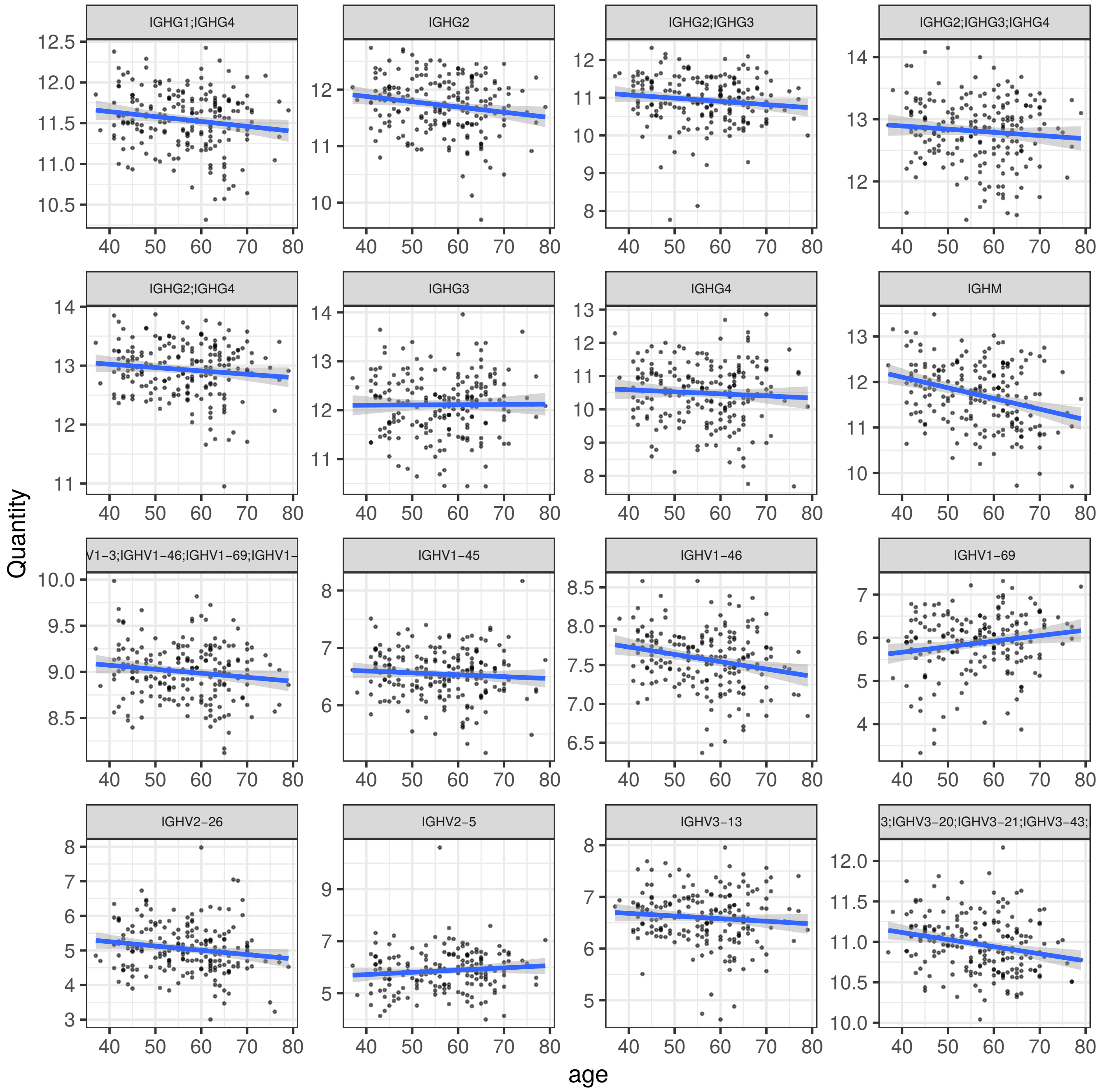
IGHG1;IGHG4
IGHG2
IGHG2;IGHG3
IGHG2;IGHG3;IGHG4
IGHG2;IGHG4
IGHG3
IGHG4
IGHM
V1-3;IGHV1-46;IGHV1-69;IGHV1-
IGHV1-45
IGHV1-46
IGHV1-69
IGHV2-26
IGHV2-5
IGHV3-13
3;IGHV3-20;IGHV3-21;IGHV3-43;
Quantity
age

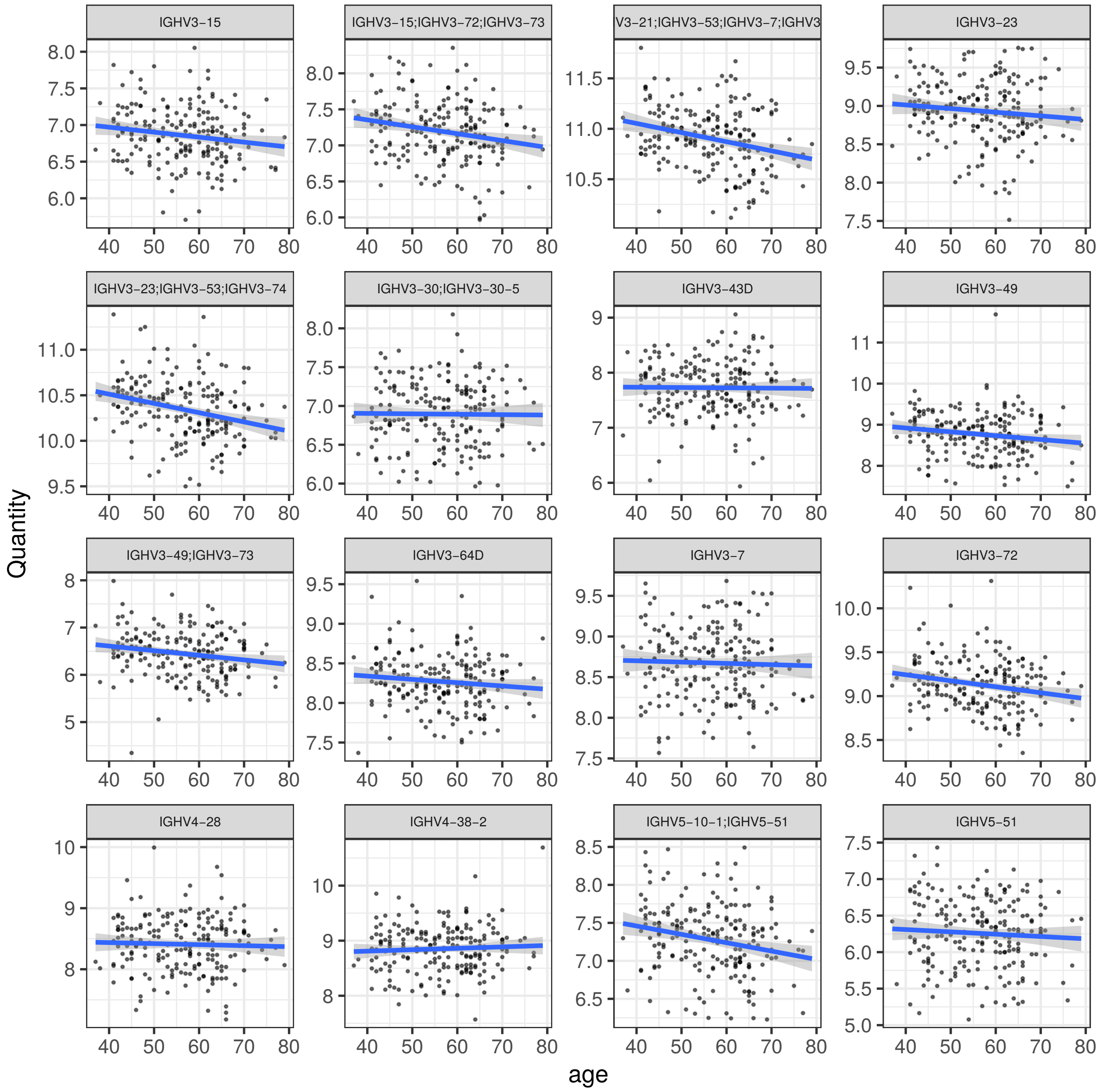
IGHV3-15
IGHV3-15;IGHV3-72;IGHV3-73
V3-21;IGHV3-53;IGHV3-7;IGHV3
IGHV3-23
IGHV3-23;IGHV3-53;IGHV3-74
IGHV3-30;IGHV3-30-5
IGHV3-43D
IGHV3-49
IGHV3-49;IGHV3-73
IGHV3-64D
IGHV3-7
IGHV3-72
IGHV4-28
IGHV4-38-2
IGHV5-10-1;IGHV5-51
IGHV5-51
Quantity
age

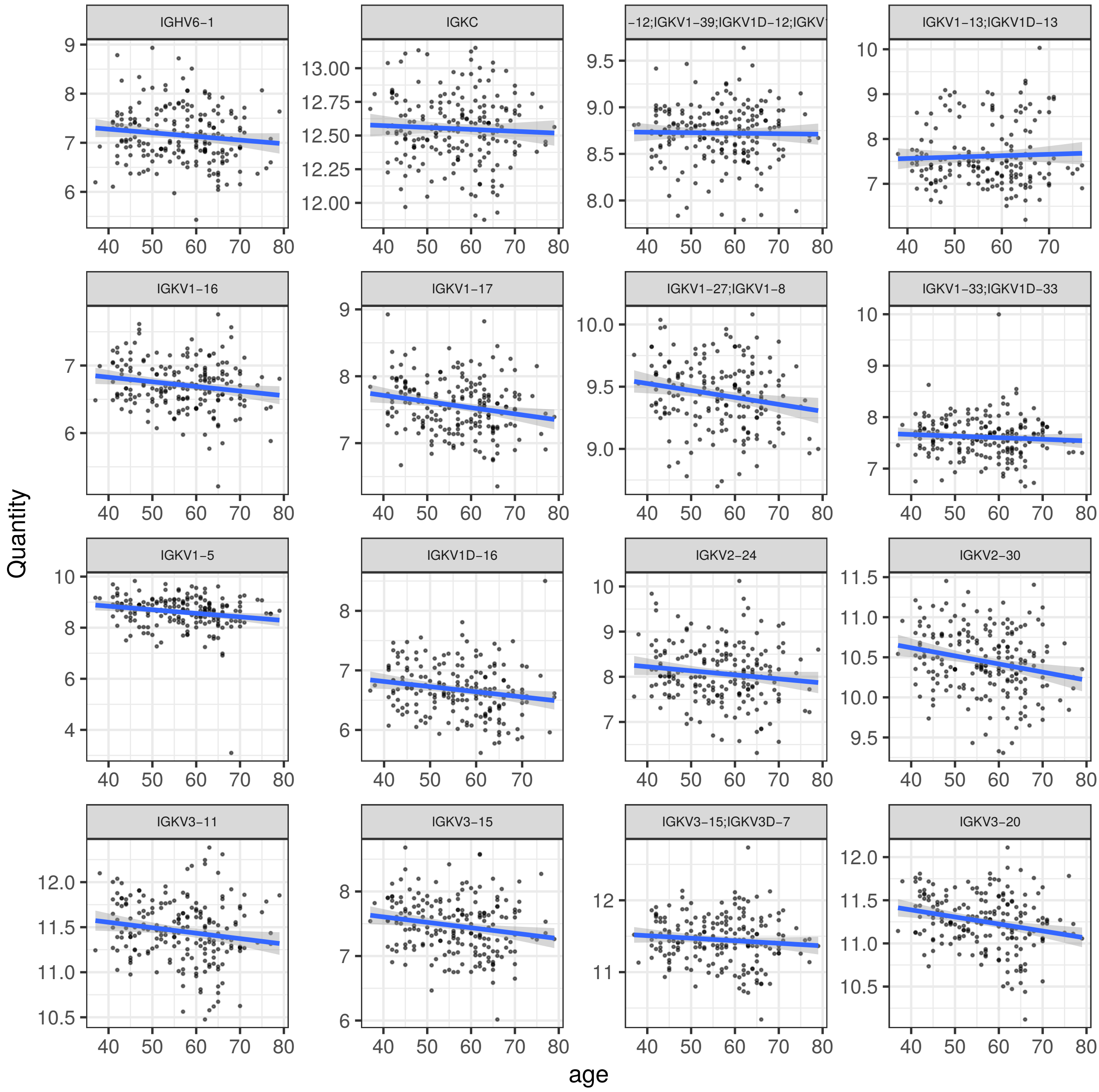
IGHV6−1
IGKC
−12;IGKV1−39;IGKV1D−12;IGKV
IGKV1−13;IGKV1D−13
IGKV1−16
IGKV1−17
IGKV1−27;IGKV1−8
IGKV1−33;IGKV1D−33
IGKV1−5
IGKV1D−16
IGKV2−24
IGKV2−30
IGKV3−11
IGKV3−15
IGKV3−15;IGKV3D−7
IGKV3−20
Quantity
age

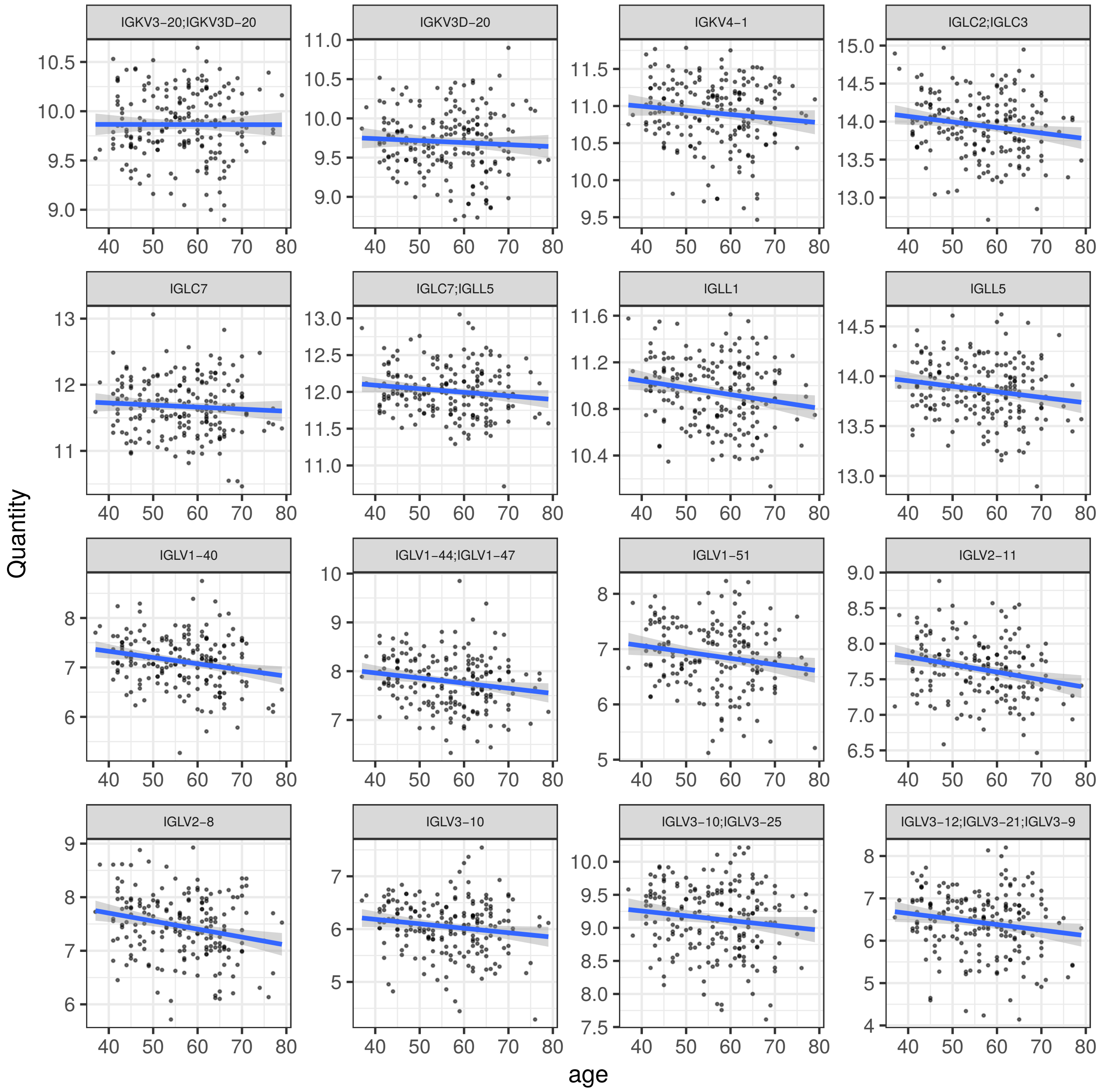
IGKV3-20;IGKV3D-20
IGKV3D-20
IGKV4-1
IGLC2;IGLC3
IGLC7
IGLC7;IGLL5
IGLL1
IGLL5
IGLV1-40
IGLV1-44;IGLV1-47
IGLV1-51
IGLV2-11
IGLV2-8
IGLV3-10
IGLV3-10;IGLV3-25
IGLV3-12;IGLV3-21;IGLV3-9
Quantity
age

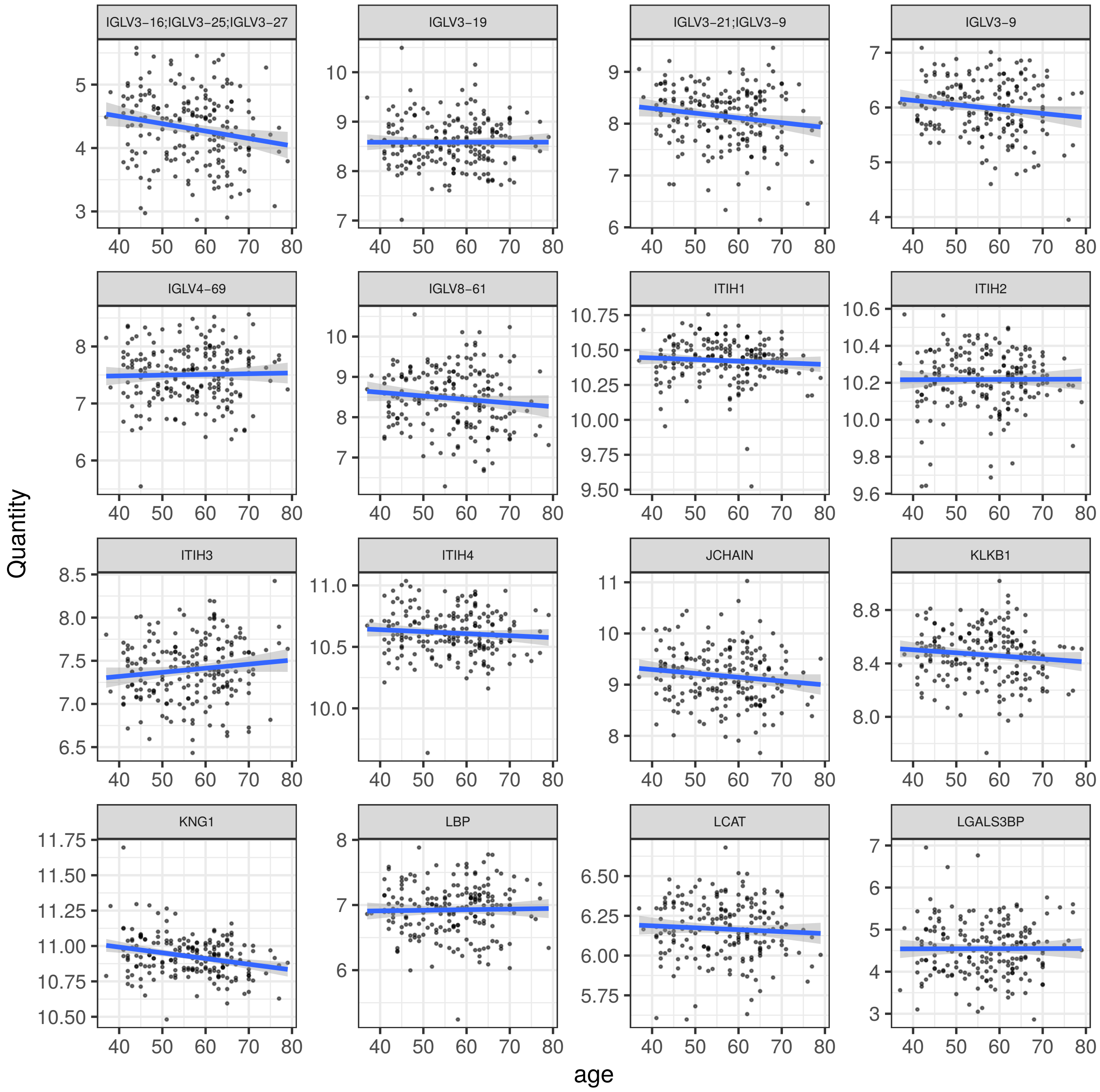
IGLV3−16;IGLV3−25;IGLV3−27
IGLV3−19
IGLV3−21;IGLV3−9
IGLV3−9
IGLV4−69
IGLV8−61
ITIH1
ITIH2
ITIH3
ITIH4
JCHAIN
KLKB1
KNG1
LBP
LCAT
LGALS3BP
Quantity
age

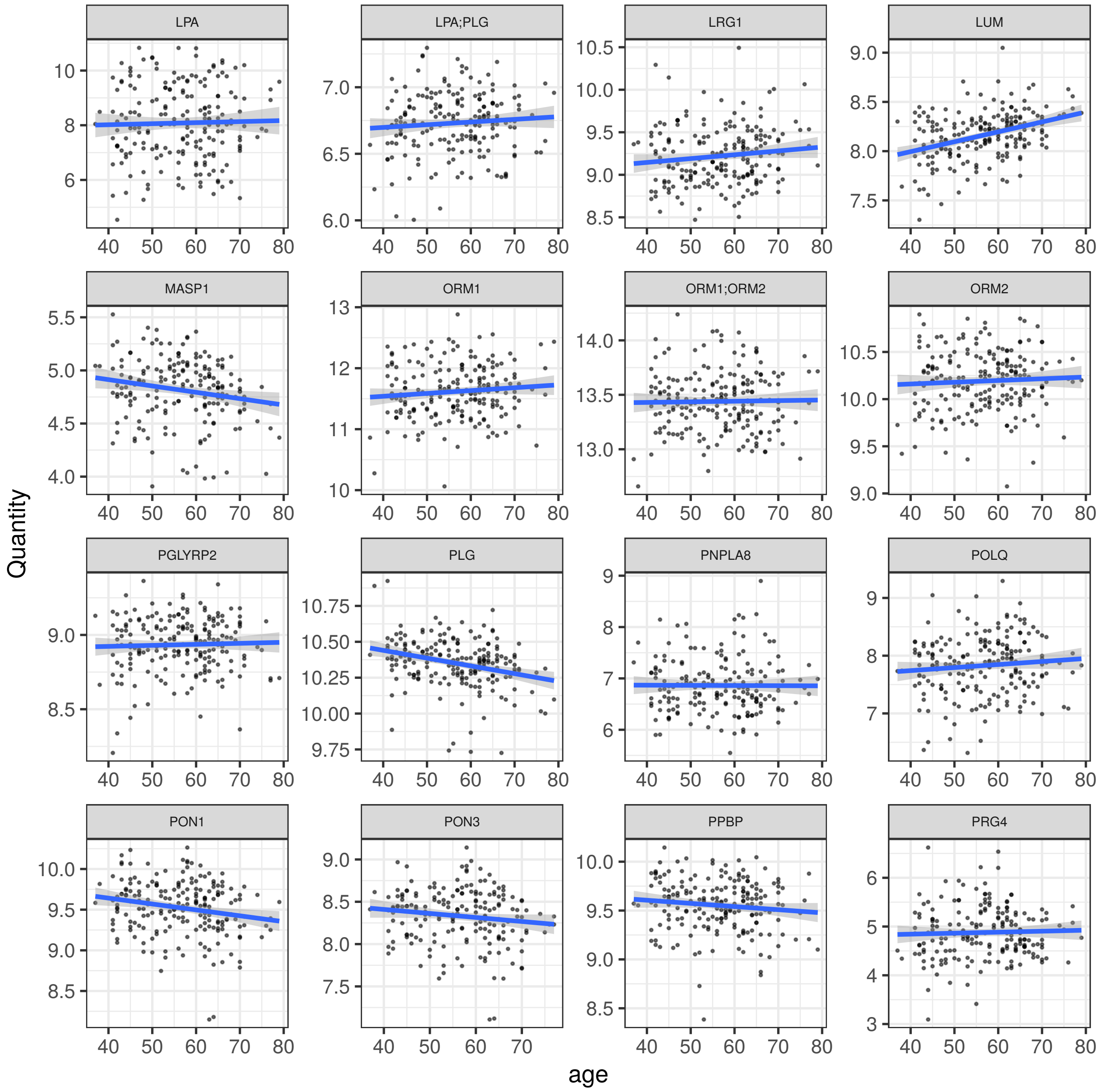

LPA
LPA;PLG
LRG1
LUM
MASP1
ORM1
ORM1;ORM2
ORM2
PGLYRP2
PLG
PNPLA8
POLQ
PON1
PON3
PPBP
PRG4
Quantity
age

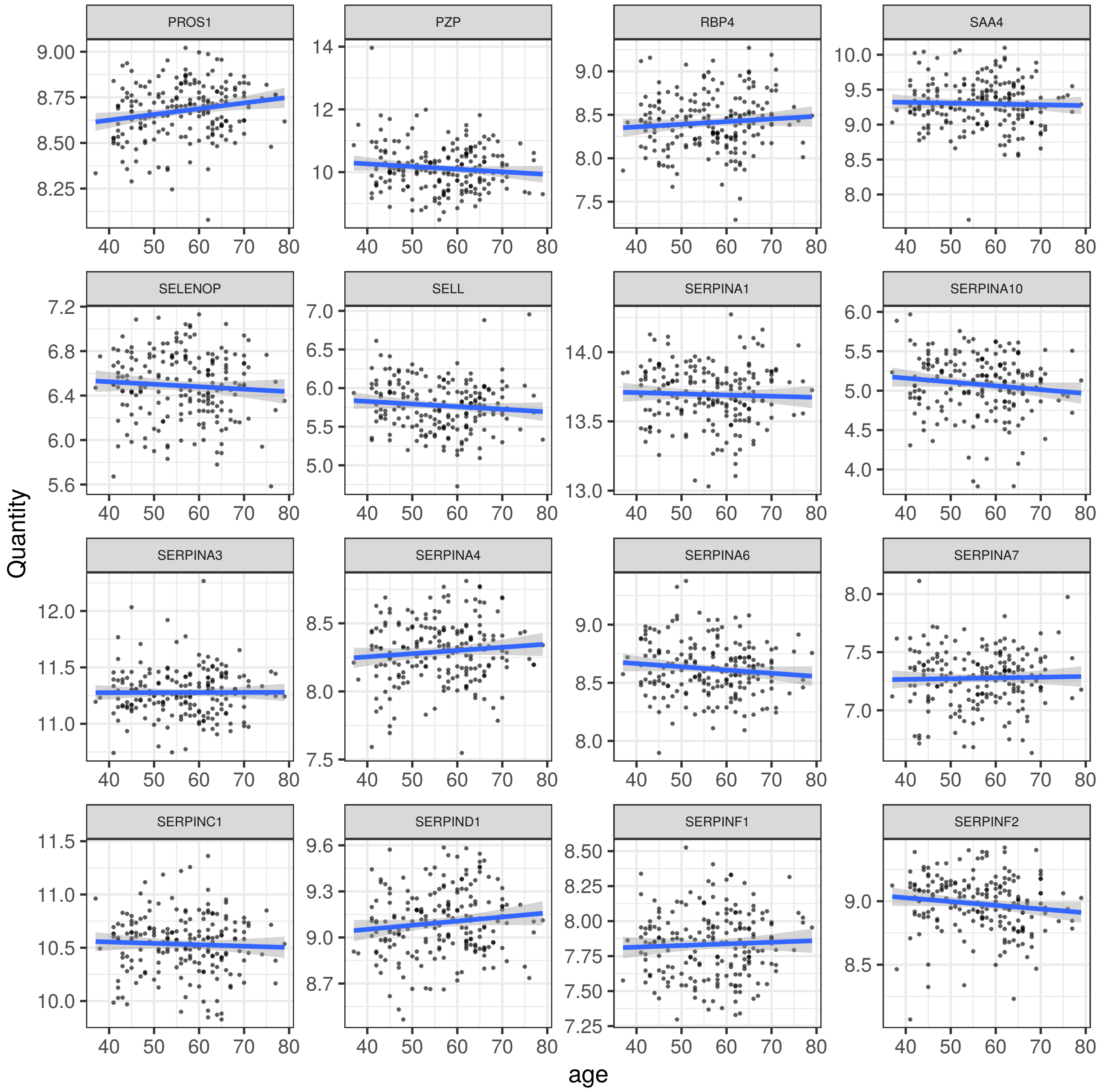

PROS1
PZP
RBP4
SAA4
SELENOP
SELL
SERPINA1
SERPINA10
SERPINA3
SERPINA4
SERPINA6
SERPINA7
SERPINC1
SERPIND1
SERPINF1
SERPINF2
Quantity
age

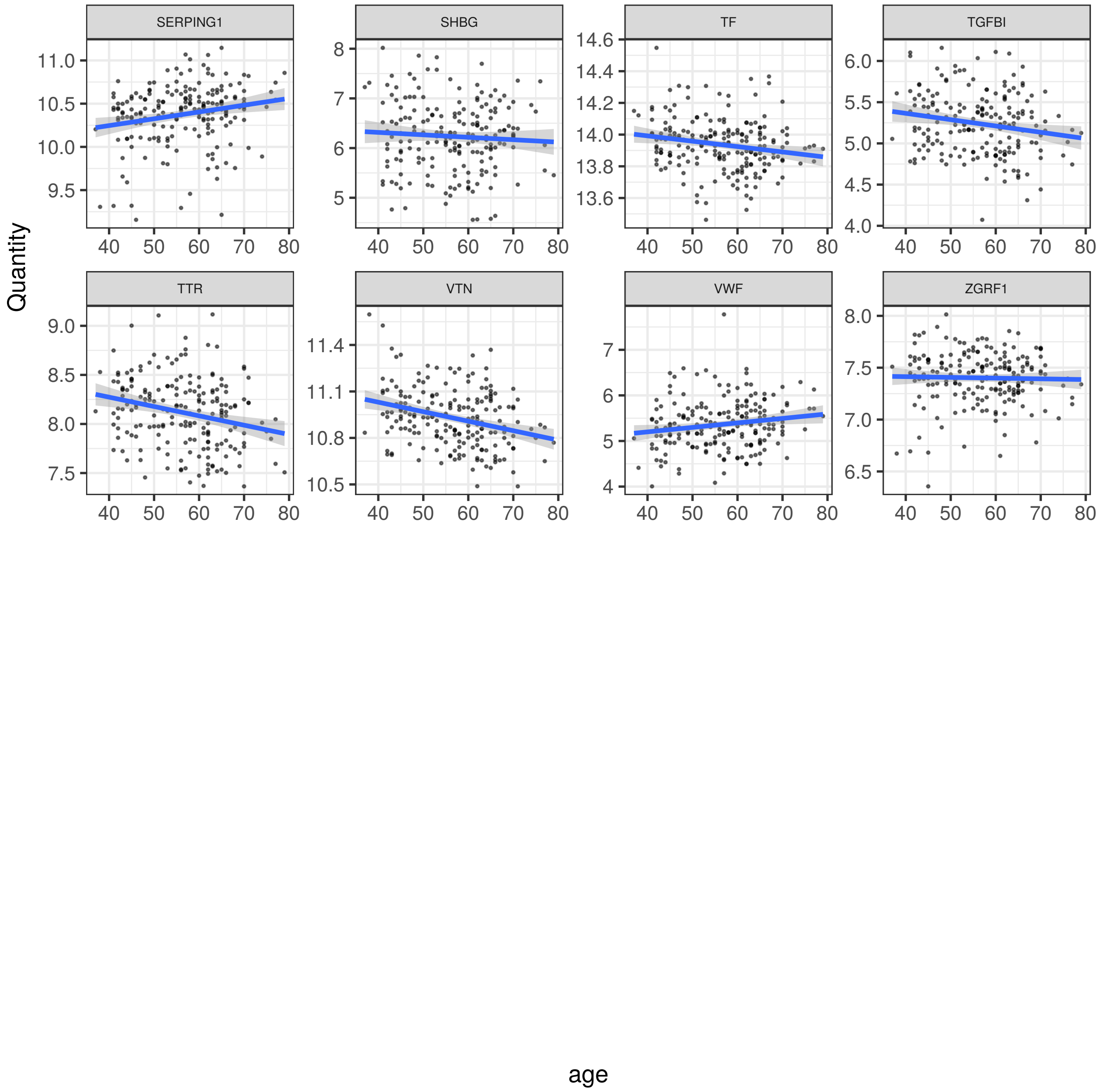

SERPING1
SHBG
TF
TGFBI
TTR
VTN
VWF
ZGRF1
Quantity
age

References Supplemental Information

Almawi, W.Y., Al-Shaikh, F.S., Melemedjian, O.K., and Almawi, A.W. (2013). Protein Z, an anticoagulant protein with expanding role in reproductive biology. Reproduction *146*, R73–R80.

Anas, A., van der Poll, T., and de Vos, A.F. (2010). Role of CD14 in lung inflammation and infection. Crit. Care *14*, 209.

Asare-Werehene, M., Communal, L., Carmona, E., Le, T., Provencher, D., Mes-Masson, A.-M., and Tsang, B.K. (2019). Pre-operative Circulating Plasma Gelsolin Predicts Residual Disease and Detects Early Stage Ovarian Cancer. Sci. Rep. *9*, 13924.

Bas, S., Gauthier, B.R., Spenato, U., Stingelin, S., and Gabay, C. (2004). CD14 is an acute-phase protein. J. Immunol. *172*, 4470–4479.

Bhanumathy, C.D., Tang, Y., Monga, S.P.S., Katuri, V., Cox, J.A., Mishra, B., and Mishra, L. (2002). Itih-4, a serine protease inhibitor regulated in interleukin-6--dependent liver formation: role in liver development and regeneration. Dev. Dyn. *223*, 59–69.

Cheng, Y., Hu, X., Liu, C., Chen, M., Wang, J., Wang, M., Gao, F., Han, J., Zhang, C., Sun, D., et al. (2017). Gelsolin Inhibits the Inflammatory Process Induced by LPS. Cell. Physiol. Biochem. *41*, 205–212.

Cox, J., Hein, M.Y., Luber, C.A., Paron, I., Nagaraj, N., and Mann, M. (2014). Accurate proteome-wide label-free quantification by delayed normalization and maximal peptide ratio extraction, termed MaxLFQ. Mol. Cell. Proteomics *13*, 2513–2526.

DiNubile, M.J. (2008). Plasma gelsolin as a biomarker of inflammation. Arthritis Res. Ther. *10*, 124.

Druhan, L.J., Lance, A., Li, S., Price, A.E., Emerson, J.T., Baxter, S.A., Gerber, J.M., and Avalos, B.R. (2017). Leucine Rich α-2 Glycoprotein: A Novel Neutrophil Granule Protein and Modulator of Myelopoiesis. PLoS One *12*, e0170261.

Fotakis, P., Kothari, V., Thomas, D.G., Westerterp, M., Molusky, M.M., Altin, E., Abramowicz, S., Wang, N., He, Y., Heinecke, J.W., et al. (2019). Anti-Inflammatory Effects of HDL (High-Density Lipoprotein) in Macrophages Predominate Over Proinflammatory Effects in Atherosclerotic Plaques. Arterioscler. Thromb. Vasc. Biol. *39*, e253–e272.

Fuior, E.V., and Gafencu, A.V. (2019). Apolipoprotein C1: Its Pleiotropic Effects in Lipid Metabolism and Beyond. Int. J. Mol. Sci. *20*, 5939.

Gordon, S.M., Hofmann, S., Askew, D.S., and Davidson, W.S. (2011). High density lipoprotein: it's not just about lipid transport anymore. Trends Endocrinol. Metab. *22*, 9–15.

Hagihara, K., Nishikawa, T., Isobe, T., Song, J., Sugamata, Y., and Yoshizaki, K. (2004). IL-6 plays a critical role in the synergistic induction of human serum amyloid A (SAA) gene when stimulated with proinflammatory cytokines as analyzed with an SAA isoform real-time quantitative RT-PCR assay system. Biochemical and Biophysical Research Communications *314*, 363–369.

Hajishengallis, G., Reis, E.S., Mastellos, D.C., Ricklin, D., and Lambris, J.D. (2017). Novel mechanisms and functions of complement. Nat. Immunol. *18*, 1288–1298.

Hamm, A., Veeck, J., Bektas, N., Wild, P.J., Hartmann, A., Heindrichs, U., Kristiansen, G., Werbowetski-Ogilvie, T., Del Maestro, R., Knuechel, R., et al. (2008). Frequent expression loss of Inter-alpha-trypsin inhibitor heavy chain (ITIH) genes in multiple human solid tumors: a systematic expression analysis. BMC Cancer *8*, 25.

Honda, H., Fujimoto, M., Serada, S., Urushima, H., Mishima, T., Lee, H., Ohkawara, T., Kohno, N., Hattori, N., Yokoyama, A., et al. (2017). Leucine-rich α-2 glycoprotein promotes lung fibrosis by modulating TGF-β signaling in fibroblasts. Physiological Reports *5*, e13556.

Hu, W., Yen, Y.-T., Singh, S., Kao, C.-L., and Wu-Hsieh, B.A. (2012). SARS-CoV Regulates Immune Function-Related Gene Expression in Human Monocytic Cells. Viral Immunology *25*, 277–288.

Jain, S., Gautam, V., and Naseem, S. (2011). Acute-phase proteins: As diagnostic tool. J. Pharm. Bioallied Sci. *3*, 118–127.

Khatri, N., Sagar, A., Peddada, N., Choudhary, V., Chopra, B.S., Garg, V., Garg, R., and Ashish (2014). Plasma gelsolin levels decrease in diabetic state and increase upon treatment with F-actin depolymerizing versions of gelsolin. J Diabetes Res *2014*, 152075.

Kong, Z., Zhou, C., Chen, L., Ren, A., Zhang, D., Basang, Z., Tan, Z., Kang, J., and Li, B. (2019). Multi-Omics Analysis Reveals Up-Regulation of APR Signaling, LXR/RXR and FXR/RXR Activation Pathways in Holstein Dairy Cows Exposed to High-Altitude Hypoxia. Animals (Basel) *9*, 406.

Kumaraswamy, S.B., Linder, A., Åkesson, P., and Dahlbäck, B. (2012). Decreased plasma concentrations of apolipoprotein M in sepsis and systemic inflammatory response syndromes. Crit. Care *16*, R60.

Lee, P.-S., Waxman, A.B., Cotich, K.L., Chung, S.W., Perrella, M.A., and Stossel, T.P. (2007). Plasma gelsolin is a marker and therapeutic agent in animal sepsis. Crit. Care Med. *35*, 849–855.

Li, G.H., Arora, P.D., Chen, Y., McCulloch, C.A., and Liu, P. (2012). Multifunctional roles of gelsolin in health and diseases. Med. Res. Rev. *32*, 999–1025.

Li, S., Lee, C., Hsu, C., Huang, H., and Su, Y. (2020). IL-6 induces haptoglobin expression through activating STAT3 in human head and neck cancer. J. Oral Pathol. Med. *49*, 49–54.

Macpherson, M.E., Halvorsen, B., Yndestad, A., Ueland, T., Mollnes, T.E., Berge, R.K., Rashidi, A., Otterdal, K., Gregersen, I., Kong, X.Y., et al. (2019). Impaired HDL Function Amplifies Systemic Inflammation in Common Variable Immunodeficiency. Sci. Rep. *9*, 9427.

Naka, T., and Fujimoto, M. (2018). LRG is a novel inflammatory marker clinically useful for the evaluation of disease activity in rheumatoid arthritis and inflammatory bowel disease. Immunol Med *41*, 62–67.

Qin, C., Zhou, L., Hu, Z., Zhang, S., Yang, S., Tao, Y., Xie, C., Ma, K., Shang, K., Wang, W., et al. (2020). Dysregulation of immune response in patients with COVID-19 in Wuhan, China. Clin. Infect. Dis. ciaa248.

Ricklin, D., Hajishengallis, G., Yang, K., and Lambris, J.D. (2010). Complement: a key system for immune surveillance and homeostasis. Nat. Immunol. *11*, 785–797.

Ricklin, D., Reis, E.S., and Lambris, J.D. (2016). Complement in disease: a defence system turning offensive. Nat. Rev. Nephrol. *12*, 383–401.

Ruan, Q., Yang, K., Wang, W., Jiang, L., and Song, J. (2020). Correction to: Clinical predictors of mortality due to COVID-19 based on an analysis of data of 150 patients from Wuhan, China. Intensive Care Med. 1–3.

Sack, G.H. (2018). Serum amyloid A – a review. Molecular Medicine *24*, 1–27.

Shi, H., Han, X., Jiang, N., Cao, Y., Alwalid, O., Gu, J., Fan, Y., and Zheng, C. (2020). Radiological findings from 81 patients with COVID-19 pneumonia in Wuhan, China: a descriptive study. Lancet Infect. Dis. *20*, 425–434.

Shirai, R., Hirano, F., Ohkura, N., Ikeda, K., and Inoue, S. (2009). Up-regulation of the expression of leucine-rich alpha(2)-glycoprotein in hepatocytes by the mediators of acute-phase response. Biochem. Biophys. Res. Commun. *382*, 776–779.

Shive, C.L., Jiang, W., Anthony, D.D., and Lederman, M.M. (2015). Soluble CD14 is a nonspecific marker of monocyte activation. AIDS *29*, 1263–1265.

Silverman, A.M., Nakata, R., Shimada, H., and Sposto, R. (2012). A Galectin-3–Dependent Pathway Upregulates Interleukin-6 in the Microenvironment of Human Neuroblastoma. Cancer Res. *72*, 2228–2238.

Sirniö, P., Väyrynen, J.P., Klintrup, K., Mäkelä, J., Mäkinen, M.J., Karttunen, T.J., and Tuomisto, A. (2017). Decreased serum apolipoprotein A1 levels are associated with poor survival and systemic inflammatory response in colorectal cancer. Sci. Rep. *7*, 5374.

Soeters, P.B., Wolfe, R.R., and Shenkin, A. (2019). Hypoalbuminemia: Pathogenesis and Clinical Significance. JPEN J. Parenter. Enteral Nutr. *43*, 181–193.

Stock, A.-M., Klee, F., Edlund, K., Grinberg, M., Hammad, S., Marchan, R., Cadenas, C., Niggemann, B., Zänker, K.S., Rahnenführer, J., et al. (2015). Gelsolin Is Associated with Longer Metastasis-free Survival and Reduced Cell Migration in Estrogen Receptor-positive Breast Cancer. Anticancer Res. *35*, 5277–5285.

Tani, S., Nagao, K., and Hirayama, A. (2016). Association of systemic inflammation with the serum apolipoprotein A-1 level: A cross-sectional pilot study. J. Cardiol. *68*, 168–177.

Wang, X., Abraham, S., McKenzie, J.A.G., Jeffs, N., Swire, M., Tripathi, V.B., Luhmann, U.F.O., Lange, C.A.K., Zhai, Z., Arthur, H.M., et al. (2013). LRG1 promotes angiogenesis by modulating endothelial TGF-β signalling. Nature *499*, 306–311.

White, R., Giordano, S., and Datta, G. (2017). Role of HDL-Associated Proteins and Lipids in

the Regulation of Inflammation. In Advances in Lipoprotein Research, T. Isbir, ed. (intechopen), p. 53.

Wittmann, J., Dieckow, J., Schröder, H., Hampel, U., Garreis, F., Jacobi, C., Milczarek, A., Hsieh, K.L., Pulli, B., Chen, J.W., et al. (2018). Plasma gelsolin promotes re-epithelialization. Sci. Rep. *8*, 13140.

World Health Organisation (2020). WHO R&D Blueprint novel Coronavirus COVID-19 Therapeutic Trial Synopsis.

Xu, G., Xia, Z., Deng, F., Liu, L., Wang, Q., Yu, Y., Wang, F., Zhu, C., Liu, W., Cheng, Z., et al. (2019). Inducible LGALS3BP/90K activates antiviral innate immune responses by targeting TRAF6 and TRAF3 complex. PLoS Pathog. *15*, e1008002.

Zhou, F., Yu, T., Du, R., Fan, G., Liu, Y., Liu, Z., Xiang, J., Wang, Y., Song, B., Gu, X., et al. (2020). Clinical course and risk factors for mortality of adult inpatients with COVID-19 in Wuhan, China: a retrospective cohort study. Lancet *395*, 1054–1062.
